# *Pseudomonas aeruginosa*-derived DnaJ functions as a novel immunomodulator inducing IFNβ via CME–SGK1–IRF3 axis in macrophages

**DOI:** 10.1038/s41598-025-31281-x

**Published:** 2025-12-03

**Authors:** Jaehoo Lee, Yeji Lee, Yongxin Jin, Weihui Wu, Un-Hwan Ha

**Affiliations:** 1 Department of Biotechnology and Bioinformatics, Korea University, Sejong, 30019 Republic of Korea; 2 Interdisciplinary Graduate Program for Artificial Intelligence Smart Convergence Technology, Korea University, Sejong, 30019 Republic of Korea; 3 State Key Laboratory of Medicinal Chemical Biology, Key Laboratory of Molecular Microbiology and Technology of the Ministry of Education, Department of Microbiology, Nankai University, Tianjin, 300071 China

**Keywords:** CME, DnaJ, IFNβ, IRF3, *Pseudomonas aeruginosa*, SGK1, Diseases, Immunology, Microbiology

## Abstract

Type I interferons (IFNs), particularly IFNβ, play a pivotal role in coordinating innate and adaptive immune responses during microbial infections. *Pseudomonas aeruginosa* (*P. aeruginosa*), a clinically significant opportunistic pathogen, is able to induce IFNβ expression; however, the specific microbial factors responsible for this induction remain poorly characterized. In this study, we identify DnaJ, a heat shock protein 40 (HSP40) homolog derived from *P. aeruginosa*, as a novel microbial inducer of IFNβ expression in macrophages. Among the bacterial HSP homologs tested, DnaJ elicits the most robust IFNβ production via a mechanism dependent on Toll-like receptor 4 (TLR4) and the TRIF–TBK1–IRF3 signaling axis. Mechanistic analysis revealed that clathrin-mediated endocytosis (CME) is required for DnaJ-induced IRF3 activation, and that serum/glucocorticoid regulated kinase 1 (SGK1) functions downstream of CME to promote IRF3 phosphorylation and subsequent IFNβ expression. Consistent with these findings, human HSP40 similarly induced IFNβ expression through the conserved CME–SGK1–IRF3 pathway, indicating that both bacterial and host-derived HSP40 proteins can serve as immune modulators. Collectively, these findings identify *P. aeruginosa* DnaJ as a potent immunomodulatory ligand capable of inducing IFNβ expression. DnaJ may therefore represent a promising candidate for therapeutic modulation of innate immunity or as an adjuvant in antimicrobial immunotherapy.

**Supplementary Information:**

The online version contains supplementary material available at 10.1038/s41598-025-31281-x.

## Introduction

Innate immunity serves as the host’s first line of defense against pathogenic infections, with macrophages playing a critical role by recognizing pathogen-associated molecular patterns (PAMPs) through pattern recognition receptors (PRRs) located on the cell surface or within endosomes^1^. Upon recognition of PAMP, PRRs initiate multiple signaling cascades that lead to the production of proinflammatory cytokines including interferons (IFNs)^2^. Type I IFNs are critical mediators of host defense, orchestrating both innate and adaptive immune responses during microbial infections^2,3^. Particularly, IFNβ is rapidly induced in pathogen-infected cells and exhibits diverse antimicrobial effects, including macrophage activation, enhancement of antigen presentation, and regulation of adaptive immune responses^3^. Furthermore, IFNβ signaling induces the expression of hundreds of IFN-stimulated genes (ISGs), which collectively confer an antiviral state that limits viral replication and spread^2,4^. Interestingly, commensal microbiota has shown to increase systemic type I IFN-mediated antiviral responses^5^, implying that bacteria can influence host susceptibility to secondary viral infection—potentially representing a broadly effective strategy for microbial competition.

*Pseudomonas aeruginosa* (*P. aeruginosa*) is an opportunistic pathogen responsible for a wide range of infections, often involving the synergistic action of multiple virulence and regulatory factors^6^. According to the WHO Bacterial Priority Pathogens List (2024), *P. aeruginosa*, *Escherichia coli* (*E. coli*), *Klebsiella pneumoniae* and *Staphylococcus aureus* pose the greatest threat to public health, as infections caused by multidrug-resistant strains of these bacteria are associated with high mortality rate. Several studies have shown that *P. aeruginosa* infection significantly increases IFNβ expression during host-pathogen interactions^7–9^. However, in cystic fibrosis airway epithelial cells, IFNβ induction is significantly attenuated, resulting in impaired immune surveillance and underscoring the critical role for IFNβ in activating innate immune defenses^10^. Despite its protective functions, IFNβ can exert detrimental effects depending on the pathogen and the stage of infection^11^. In murine models of acute *P. aeruginosa* lung infection, IFNβ promotes the formation of neutrophil extracellular traps (NETs), which facilitate biofilm development and creates a protective niche that enhances bacterial persistence^12^. Additionally, IFNβ has been shown to support the intracellular survival of *P. aeruginosa* within macrophages^9^. These findings suggest that IFNβ may paradoxically contribute to disease severity by fostering an environment conductive to bacterial colonization. Collectively, these observations highlight the need for tightly regulated IFNβ production following infection. While appropriate IFNβ responses are essential for pathogen clearance, *P. aeruginosa* may manipulate this pathway to evade immune responses and promote chronic infection.

*P. aeruginosa* stimulates host immune responses through various PAMPs, including bacterial heat shock protein (HSP) homologs^13,14^. HSPs are highly conserved molecular chaperones found across species, from bacteria to humans^15,16^, and when released into the extracellular environment—such as during cellular stress or damage—they can function as immune activators, acting as either PAMPs or damage-associated molecular patterns (DAMPs)^17,18^. We have previously demonstrated that *P. aeruginosa*-derived HSPs modulate innate immune responses in human macrophages^19–28^. Among these, the HSP40 homolog DnaJ, which is released extracellularly from *P. aeruginosa*^25^, exhibits a range of immunomodulatory functions, including the induction of IL-1β, TLR2, and TLR7 expression^25,27,28^, as well as the suppression of excessive IL-8 production through upregulation of miR-146a^26^. These findings suggest that DnaJ is capable of activating multiple immune signaling pathways and may play a multifaceted role in host-pathogen interactions.

In this study, we identified *P. aeruginosa*-derived DnaJ as a specific inducer of IFNβ expression. The efficiency with which DnaJ stimulates the IFNβ response may represent an important factor in developing therapeutic strategies against microbial infections. Accordingly, we sought to elucidate the mechanism by which DnaJ induces IFNβ expression. Our findings demonstrate that DnaJ induces IFNβ production through a signaling pathway involving clathrin-mediated endocytosis (CME) and the subsequent activation of SGK1 and IRF3 downstream of TLR4. Furthermore, we showed that human HSP40 (hHSP40) also induces IFNβ through a similar mechanism, highlighting an evolutionary conserved role of HSP40 family members in innate immune signaling. The ability to trigger or counteract the IFNβ response is likely critical for the host’s capacity to control microbial infections. These findings provide new insights into the role of bacterial HSPs in IFNβ regulation and may inform the development of novel strategies for modulating antimicrobial immune responses.

## Results

### *P. aeruginosa*-derived DnaJ induces IFNβ expression

**Fig. 1:**
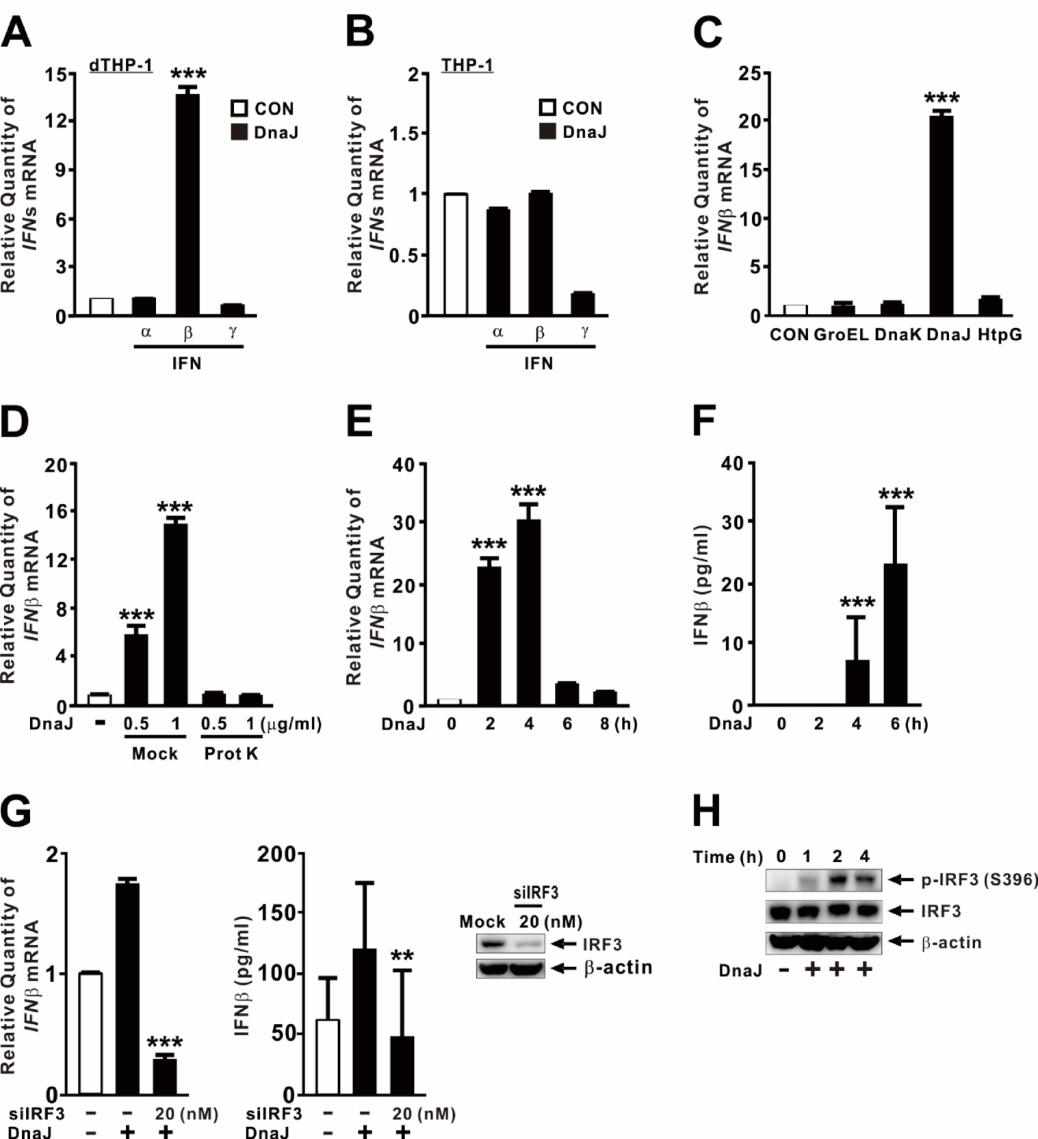
*P. aeruginosa*-derived DnaJ induces IFNβ expression. (**A**, **C**, **D**) dTHP-1 cells were treated for 4 h with (**A**) DnaJ (1 µg/ml), (**C**) recombinant HSP homologs (1 µg/ml), or (**D**) DnaJ at the indicated concentration with or without pretreatment with proteinase K (20 µg/ml, 1 h). (**B**) THP-1 cells were treated for 4 h with DnaJ (1 µg/ml). (**E**, **F**, **H**) Cells were treated with DnaJ (1 µg/ml) for the indicated durations. (**G**) Cells were transfected with IRF3 siRNA (siIRF3) for 48 h, followed by DnaJ treatment (1 µg/ml) for 4 h. Knockdown efficiency is shown in the right panel of (**G**). IFNβ mRNA and protein levels were analyzed by qPCR and ELISA, respectively; protein expression was assessed by immunoblotting. The samples used for immunoblotting were obtained from the same experiment, and the original blots/gels are shown in the Supplementary Figure. Data in (**A**–**G**) are expressed as means ± SD (*n* = 3). Immunoblots are representative of three experiments. ***, *p* < 0.001 vs. CON (**A**–**F**) and DnaJ treatment alone (**G**).

To investigate whether DnaJ regulates type Ⅰ and II IFN expression, we purified recombinant DnaJ protein using a previously established protocol^28^. As a control, an extract from *E. coli* containing an empty vector was processed in parallel using the same purification procedure. As shown in Fig. 1A, DnaJ specifically induced IFNβ expression in macrophages, with minimal effects on IFNα and IFNγ. Notably, DnaJ did not induce IFNβ expression in THP-1 monocytes (Fig. 1B), suggesting a macrophage-specific effect. Building on our previous findings that *P. aeruginosa*-derived HSP homologs (GroEL, DnaK, DnaJ, and HtpG) modulate host innate immune responses in human macrophages^19–28^, we compared the IFNβ-inducing capacities of these homologs. All proteins were purified using the same protocol^19,21,24,28^. Among them, DnaJ exhibited the most potent induction of IFNβ, while the others had minimal effects (Fig. 1C). DnaJ-induced IFNβ expression was both dose- and time-dependent (Fig. 1D and F). A concentration of 1 µg/ml was sufficient to induce IFNβ expression, with mRNA levels peaking at 4 h post-treatment and protein secretion reaching a maximum at 6 h.

To confirm that this effect was directly attributable to the DnaJ protein, we treated DnaJ with proteinase K prior to stimulation. This treatment abolished its ability to induce IFNβ, confirming the specificity of the protein-mediated response (Fig. 1D). Given the essential role of IRF3 in early IFNβ transcription in response to microbial stimuli, we next examined whether IRF3 is involved in DnaJ-mediated IFNβ induction. Knockdown of IRF3 using siRNA (siIRF3) significantly reduced IFNβ expression and protein secretion following DnaJ treatment (Fig. 1G, *left and middle panels*), and successful knockdown was verified by reduced IRF3 protein levels (Fig. 1G, *right panel*). In addition, DnaJ stimulation increased IRF3 phosphorylation in a time-dependent manner, with detectable phosphorylation at 1 h and a peak at 2 h (Fig. 1H). Collectively, these findings demonstrate that *P. aeruginosa*-derived DnaJ specifically induces IFNβ expression in macrophages through activation of IRF3.

### DnaJ-induced IFNβ expression is regulated by TLR4

**Fig. 2:**
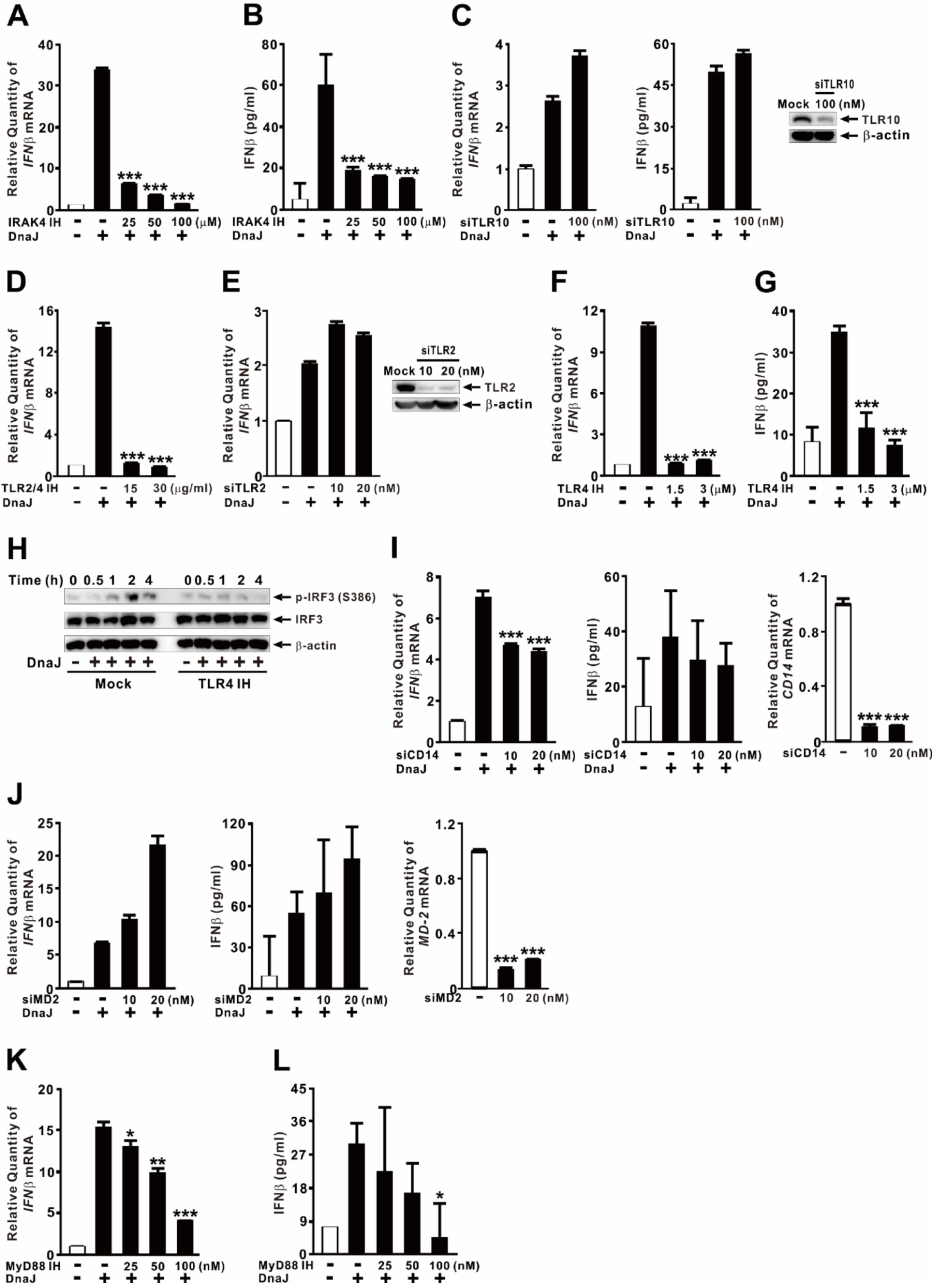
DnaJ-induced IFNβ expression is regulated by TLR4. (**A**, **B**, **D**, **F**–**H**, **K**, **L**) dTHP-1 cells were pretreated for 1 h with: (**A**, **B**) IRAK4 IH (compound 26), (**D**) TLR2/4 IH (OxPAPC), (**F**–**H**) TLR4 IH (CLI-095; 3 µM in **H**), or (**K**, **L**) MyD88 IH (T6167923). (**C**, **E**, **I**, **J**) Cells were transfected with: (**C**) siTLR10, (**E**) siTLR2, (**I**) siCD14, or (**J**) siMD2 for 48 h. Knockdown efficiency is shown in the right panel of (**C**, **E**, **I**, **J**). Following pretreatment or transfection, cells were treated with DnaJ (1 µg/ml) for 4 h (**A**–**G**, **I**–**L**) or for the indicated durations (**H**). IFNβ mRNA was quantified by qPCR; protein levels were analyzed by immunoblotting. The samples used for immunoblotting were obtained from the same experiment, and the original blots/gels are shown in the Supplementary Figure. Data in (**A**–**G**, **I**–**L**) are expressed as means ± SD (*n* = 3). Immunoblots are representative of three experiments. *, *p* < 0.05; **, *p* < 0.01; ***, *p* < 0.001 vs. DnaJ treatment alone. Inhibitor (IH).

To investigate which receptor mediates DnaJ-induced IFNβ expression, we first examined the role of IRAK4, a key adaptor kinase in TLR signaling. As shown in Fig. 2A and B, pretreatment with an IRAK4 inhibitor significantly reduced DnaJ-induced IFNβ expression and protein secretion, suggesting TLR-mediated signaling is involved in this response. We next assessed the role of TLR10, as previous studies implicated TLR10 in DnaJ-mediated modulation of innate immunity^28^. However, knockdown of TLR10 using siRNA (siTLR10) did not affect IFNβ expression and protein secretion following DnaJ treatment (Fig. 2C, *left and middle panels*), and knockdown efficiency was confirmed by TLR10 protein levels (Fig. 2C, *right panel*). In contrast, pretreatment with the TLR2/4 inhibitor OxPAPC significantly suppressed DnaJ-induced IFNβ mRNA expression (Fig. 2D).

To differentiate the contribution of TLR2 and TLR4, we selectively knocked down TLR2 using siRNA (siTLR2). As shown in Fig. 2E (*left panel*), TLR2 knockdown did not alter DnaJ-induced IFNβ expression, indicating that TLR2 is not involved in this pathway. The efficiency of TLR2 knockdown was confirmed at the protein level (Fig. 2E, *right panel*). In contrast, pretreatment with the specific TLR4 inhibitor CLI-095 markedly suppressed DnaJ-induced IFNβ mRNA and protein expression (Fig. 2F and G) and abolished IRF3 phosphorylation (Fig. 2H), indicating that TLR4 is the primary receptor mediating DnaJ recognition and signaling to IRF3. Because TLR4, which uses LPS as a prototypical agonist, requires the co-receptors CD14 and MD2 that directly interact with LPS^29^, we next examined their involvement. Knockdown of CD14 using siRNA (siCD14) partially reduced DnaJ-induced IFNβ expression but did not alter IFNβ protein levels (Fig. 2I). Similarly, MD2 knockdown (siMD2) had no effect on DnaJ-induced IFNβ expression, suggesting that, unlike LPS, DnaJ does not depend on CD14 or MD2 for TLR4-mediated signaling. Knockdown efficiency was confirmed at the mRNA level (Fig. 2I and J, *right panels*). Given the involvement of TLR4, we further investigated the role of the adaptor protein myeloid differentiation primary response 88 (MyD88). Pretreatment with the MyD88 inhibitor T6167923 led to a moderate reduction in DnaJ-induced IFNβ expression and protein secretion (Fig. 2K and L), though the effect was less pronounced than that observed with the IRAK4 inhibitor. This suggests that the MyD88-dependent pathway partially contributes to IFNβ induction, but the TRIF-dependent pathway likely plays a dominant role. To rule out the possibility that the reduction in IFNβ was due to cytotoxicity caused by the chemical inhibitors, we performed LDH release and MTT assays, which showed minimal cytotoxicity (data not shown). Collectively, these results demonstrate that DnaJ induces IFNβ transcription primarily through a TLR4-dependent signaling pathway, culminating in IRF3 phosphorylation and activation.

### DnaJ-induced IFNβ expression is mediated via the TRIF-dependent pathway

**Fig. 3:**
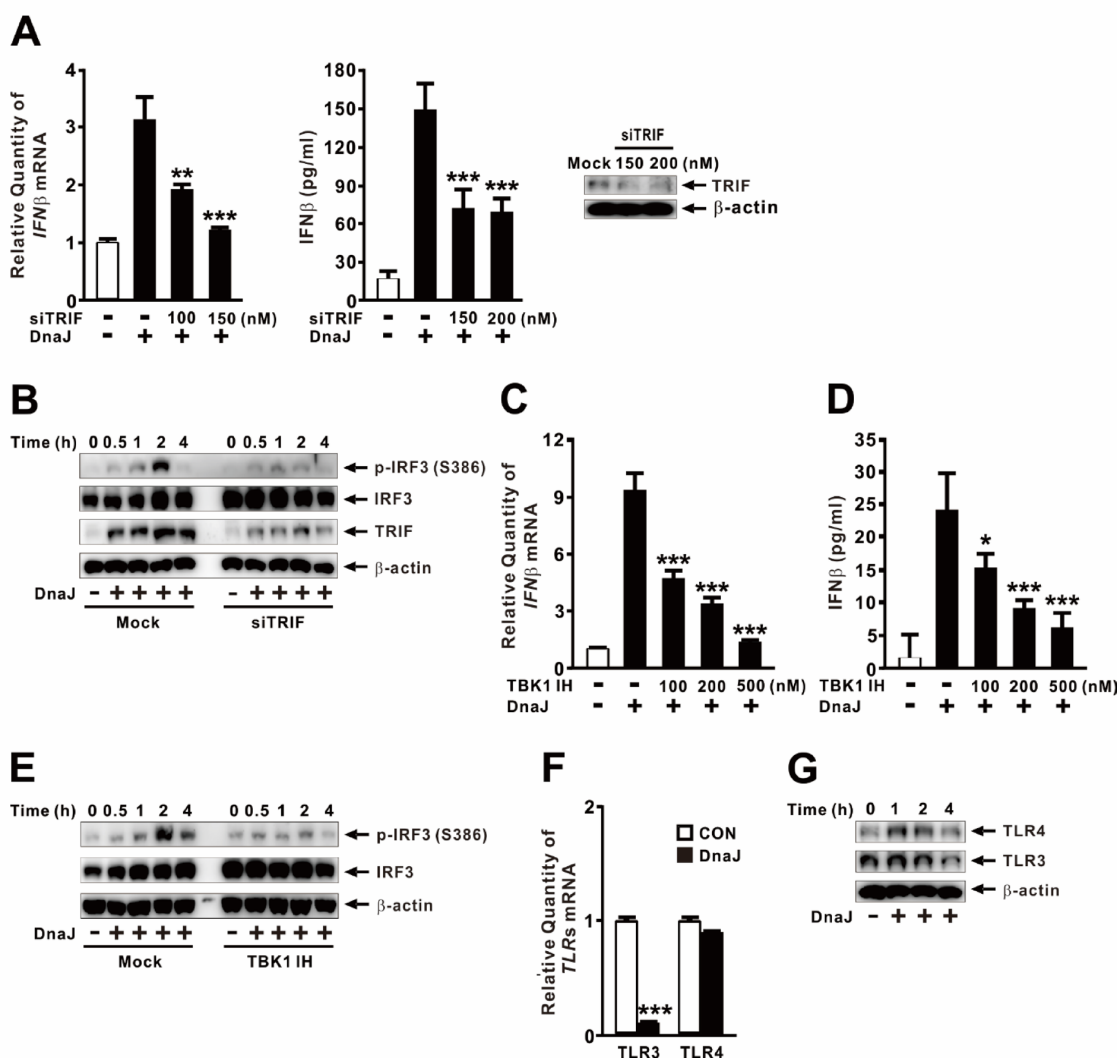
DnaJ-induced IFNβ expression is mediated via the TRIF-dependent pathway. (**A**, **B**) dTHP-1 cells were transfected with siTRIF (150 nM in **B**) for 48 h. Knockdown efficiency is shown in the right panel of (**A**). (**C**–**E**) Cells were pretreated for 1 h with TBK1 IH (MRT67307; 200 nM in **E**). Following transfection or pretreatment, cells were treated with DnaJ (1 µg/ml) for 4 h (**A**, **C**, **D**) or for the indicated durations (**B**, **E**). (**F**, **G**) Cells were treated with DnaJ (1 µg/ml) for 4 h (**F**) or for the indicated durations (**G**). IFNβ mRNA and protein levels were analyzed by qPCR and ELISA, respectively; protein expression was assessed by immunoblotting. The samples used for immunoblotting were obtained from the same experiment, and the original blots/gels are shown in the Supplementary Figure. Data in (**A**, **C**, **D**, **F**) are expressed as means ± SD (*n* = 3). Immunoblots are representative of three experiments. *, *p* < 0.05; **, *p* < 0.01; ***, *p* < 0.001 vs. DnaJ treatment alone (**A**, **C**, **D**) or CON (**F**).

Given that TLR4 can signal through adaptor proteins other than MyD88, we investigated the role of TRIF (toll-interleukin 1 (IL-1) receptor homology (TIR) domain-containing adaptor inducing IFN-β), an alternative adaptor involved in TLR4 signaling. As shown in Fig. 3A, knockdown of TRIF using siRNA (siTRIF) significantly reduced DnaJ-induced IFNβ expression in a dose-dependent manner (*left panel*). This inhibitory effect was further confirmed at the protein level: TRIF knockdown markedly decreased IFNβ secretion in response to DnaJ treatment (*middle panel*). Knockdown efficiency was validated by measuring TRIF protein levels (*right panel*). To further assess the role of TRIF in DnaJ-mediated IRF3 activation, we examined the phosphorylation status of IRF3 following TRIF knockdown. As shown in Fig. 3B, DnaJ treatment triggered a time-dependent increase in IRF3 phosphorylation in control cells, peaking at 2 h post-treatment. In contrast, TRIF knockdown significantly impaired IRF3 phosphorylation, indicating that TRIF is essential for DnaJ-mediated IRF3 activation. Knockdown efficiency was confirmed by TRIF protein measurement.

Since TRIF-dependent signaling typically activates TANK-binding kinase 1 (TBK1), which directly phosphorylates IRF3, we next examined the involvement of TBK1. Pretreatment with the TBK1-specific inhibitor MRT67307 significantly suppressed DnaJ-induced IFNβ expression and protein secretion in a dose-dependent manner (Fig. 3C and D). To rule out the possibility that the reduction in IFNβ was due to cytotoxicity caused by the chemical inhibitor, we performed LDH release and MTT assays, which showed minimal cytotoxicity (data not shown). Consistently, MRT67307 also markedly reduced IRF3 phosphorylation over time following DnaJ treatment (Fig. 3E). These results confirm that the TRIF-TBK1 axis is critical for IRF3 activation and subsequent IFNβ induction in response to DnaJ. Given that TRIF is classically associated with TLR3 and TLR4 signaling^30^, and that IRF3 is activated by TBK1 downstream of both receptors^31^, we examined whether DnaJ alters the expression of these TLRs. Interestingly, DnaJ significantly reduced TLR3 mRNA expression while having minimal impact on TLR4 (Fig. 3F). DnaJ treatment was further shown to gradually reduce TLR3 production while simultaneously increasing TLR4 production at the early stage (Fig. 3G), suggesting that TRIF-mediated IFNβ induction in this context is primarily mediated through TLR4. Taken together, these findings demonstrate that DnaJ induces IFNβ expression through a TRIF-dependent signaling pathway involving TBK1-mediated phosphorylation of IRF3.

### DnaJ-mediated IFNβ induction requires clathrin-mediated endocytosis

**Fig. 4:**
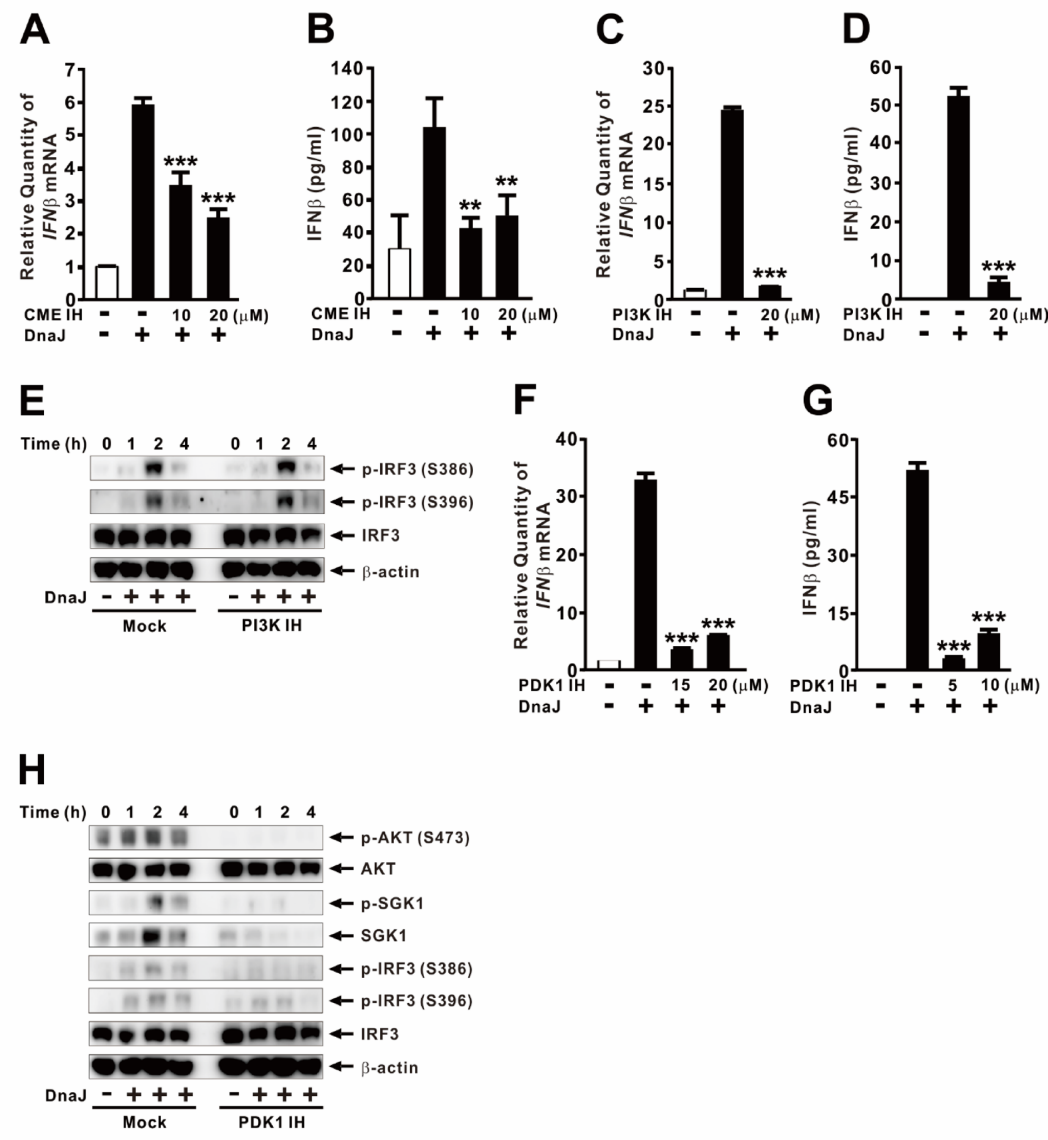
DnaJ-mediated IFNβ induction requires clathrin-mediated endocytosis. dTHP-1 cells were pretreated for 1 h with: (**A**, **B**) clathrin-mediated endocytosis (CME) IH (Pitstop2), (**C**–**E**) PI3K IH (LY294002; 20 µM in **E**), (**F**–**H**) PDK1 IH (GSK2334470; 20 µM in **H**). Cells were then treated with DnaJ (1 µg/ml) for 4 h (**A**–**D**, **F**, **G**) or for the indicated times (**E**, **H**). IFNβ mRNA and protein levels were analyzed by qPCR and ELISA, respectively; protein expression was assessed by immunoblotting. The samples used for immunoblotting were obtained from the same experiment, and the original blots/gels are shown in the Supplementary Figure. Data in (**A**–**D**, **F**, **G**) are expressed as means ± SD (*n* = 3). Immunoblots are representative of three experiments. **, *p* < 0.01; ***, *p* < 0.001 vs. DnaJ treatment alone.

TLR4 activates TRIF-dependent signaling following internalization into endosomes via CME, a process essential for type I IFN induction during infection^32^. To evaluate the role of CME in DnaJ-induced IFNβ expression, we examined the effect of Pitstop2, a specific CME inhibitor. As shown in Fig. 4A, pretreatment with Pitstop2 significantly reduced IFNβ expression in a dose-dependent manner. This inhibitory effect was also confirmed at the protein level (Fig. 4B), supporting the conclusion that CME is required for DnaJ-mediated signaling leading to IFNβ induction. Since PI3K signaling is known to regulate endocytosis and TLR4 trafficking^33^, we next assessed its involvement using the PI3K-specific inhibitor LY294002. Pretreatment with LY294002 markedly suppressed DnaJ-induced IFNβ expression (Fig. 4C) and protein secretion (Fig. 4D), indicating that PI3K activity contributes to this response. However, LY294002 did not affect IRF3 phosphorylation at either Ser386—crucial for IRF3 activation—or Ser396—which stabilizes the active IRF3 conformation and enhances transcriptional activity (Fig. 4E)^34^.

Given that PI3K signaling recruits phosphoinositide-dependent kinase 1 (PDK1), we next examined the role of PDK1 in DnaJ-induced IFNβ expression. Pretreatment with the PDK1 inhibitor GSK2334470 significantly reduced both IFNβ mRNA expression and protein secretion (Fig. 4F and G). Moreover, GSK2334470 reduced IRF3 phosphorylation at both Ser386 and Ser396, and the inhibition correlated with decreased phosphorylation of AKT and SGK1, confirming the effectiveness of the inhibitor (Fig. 4H). Interestingly, endogenous SGK1 levels were altered by treatment with DnaJ. These findings indicate that DnaJ-induced IRF3 phosphorylation and IFNβ expression occur through a PI3K-independent but PDK1-dependent signaling pathway. To rule out the possibility that the reduction in IFNβ was due to cytotoxicity caused by the chemical inhibitors, we performed LDH release and MTT assays, which showed minimal cytotoxicity (data not shown)^35–37^. Collectively, the data demonstrate that DnaJ triggers IFNβ production via a mechanism involving CME-dependent internalization and subsequent activation of PDK1-mediated signaling.

### DnaJ induces IFNβ expression via SGK1-IRF3 signaling

**Fig. 5:**
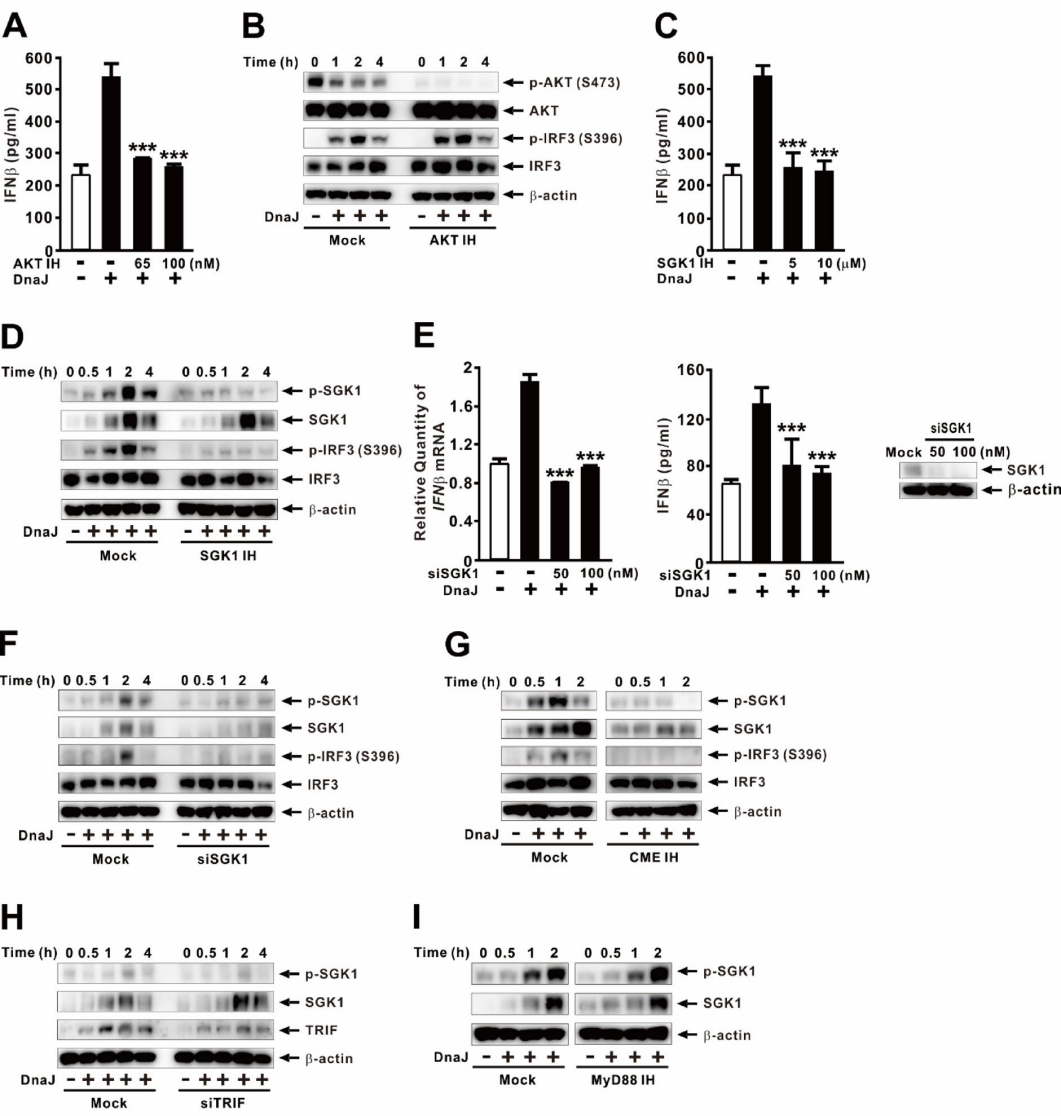
DnaJ induces IFNβ expression via SGK1-IRF3 signaling. (**A**–**D**, **G**, **I**) dTHP-1 cells were pretreated for 1 h with: (**A**, **B**) AKT IH (MK-2206; 100 nM in **B**), (**C**, **D**) SGK1 IH (GSK650394; 10 µM in **D**), (**G**) CME IH (Pitstop2; 20 µM), or (**I**) MyD88 IH (T6167923; 100 nM). (**E**, **F**, **H**) Cells were transfected for 48 h with: (**E**, **F**) siSGK1 (100 nM in **F**), or (**H**) siTRIF (150 nM). Knockdown efficiency is shown in the right panel of (**E**). Following pretreatment or transfection, cells were treated with DnaJ (1 µg/ml) for 4 h (**A**, **C**, **E**) or for the indicated times (**B**, **D**, **F**–**I**). IFNβ mRNA and protein levels were analyzed by qPCR and ELISA; protein levels were analyzed by immunoblotting. The samples used for immunoblotting were obtained from the same experiment, and the original blots/gels are shown in the Supplementary Figure. Data in (**A**, **C**, **E**) are expressed as means ± SD (*n* = 3). Immunoblots are representative of three experiments. ***, *p* < 0.001 vs. DnaJ treatment alone (**A**, **C**, **E**).

Given that PDK1 activates AKT, a kinase involved in diverse cellular processes, we first evaluated the role of AKT in DnaJ-induced IFNβ expression. Pretreatment with the AKT inhibitor MK-2206 significantly reduced IFNβ protein secretion in response to DnaJ (Fig. 5A). However, while MK-2206 effectively abolished DnaJ-induced phosphorylation of AKT at Ser473, it had no effect on IRF3 phosphorylation (Fig. 5B), consistent with the findings from PI3K inhibition. These results suggest that AKT is not directly involved in IRF3 activation. We next focused on SGK1, another downstream effector of PDK1 that can function independently of AKT^38,39^. Pretreatment with the SGK1 inhibitor GSK650394 significantly reduced DnaJ-induced IFNβ secretion. Time-course analysis revealed that DnaJ induced phosphorylation of both SGK1 and IRF3 in a time-dependent manner, with maximal phosphorylation observed at 2 h post-treatment (Fig. 5D). Notably, SGK1 inhibition with GSK650394 significantly suppressed phosphorylation of both SGK1 and IRF3, suggesting that SGK1 activation is essential for IRF3 phosphorylation and subsequent IFNβ induction. To further validate the role of SGK1, we performed siRNA-mediated knockdown of SGK1. As shown in Fig. 5E (*left and middle panels*), knockdown of SGK1 significantly reduced IFNβ expression and protein secretion following DnaJ treatment, with knockdown efficiency confirmed by SGK1 protein levels (Fig. 5E, *right panel*). A time-course analysis in SGK1-deficient cells further demonstrated that both SGK1 and IRF3 phosphorylation were markedly reduced upon DnaJ stimulation (Fig. 5F), reinforcing the requirement of SGK1 for IRF3 activation and IFNβ production.

To determine whether SGK1 activation depends on endocytosis, we pretreated cells with a CME inhibitor. As shown in Fig. 5G, inhibition of CME significantly suppressed DnaJ-induced phosphorylation of both SGK1 and IRF3. Interestingly, endogenous SGK1 levels were also altered by treatment with the inhibitor. To assess the relationship between TRIF and the SGK1, we examined SGK1 phosphorylation in TRIF knockdown cells. As shown in Fig. 5H, TRIF knockdown did not significantly affect SGK1 production and phosphorylation, suggesting that SGK1 acts upstream of or independently from TRIF. Knockdown efficiency was confirmed by TRIF protein measurement. Furthermore, pretreatment with a MyD88 inhibitor did not alter SGK1 production and phosphorylation (Fig. 5I), indicating that MyD88 is not involved in SGK1 activation. To rule out the possibility that the reduction in IFNβ was due to cytotoxicity caused by the chemical inhibitors, we performed LDH release and MTT assays, which showed minimal cytotoxicity (data not shown). Collectively, these results demonstrate that DnaJ induces IFNβ expression via a CME-dependent activation of SGK1, which in turn drives IRF3 phosphorylation and activation.

### Human HSP40 induces IFNβ expression via CME-dependent SGK1-IRF3 signaling, similar to *P. aeruginosa*-derived DnaJ

**Fig. 6:**
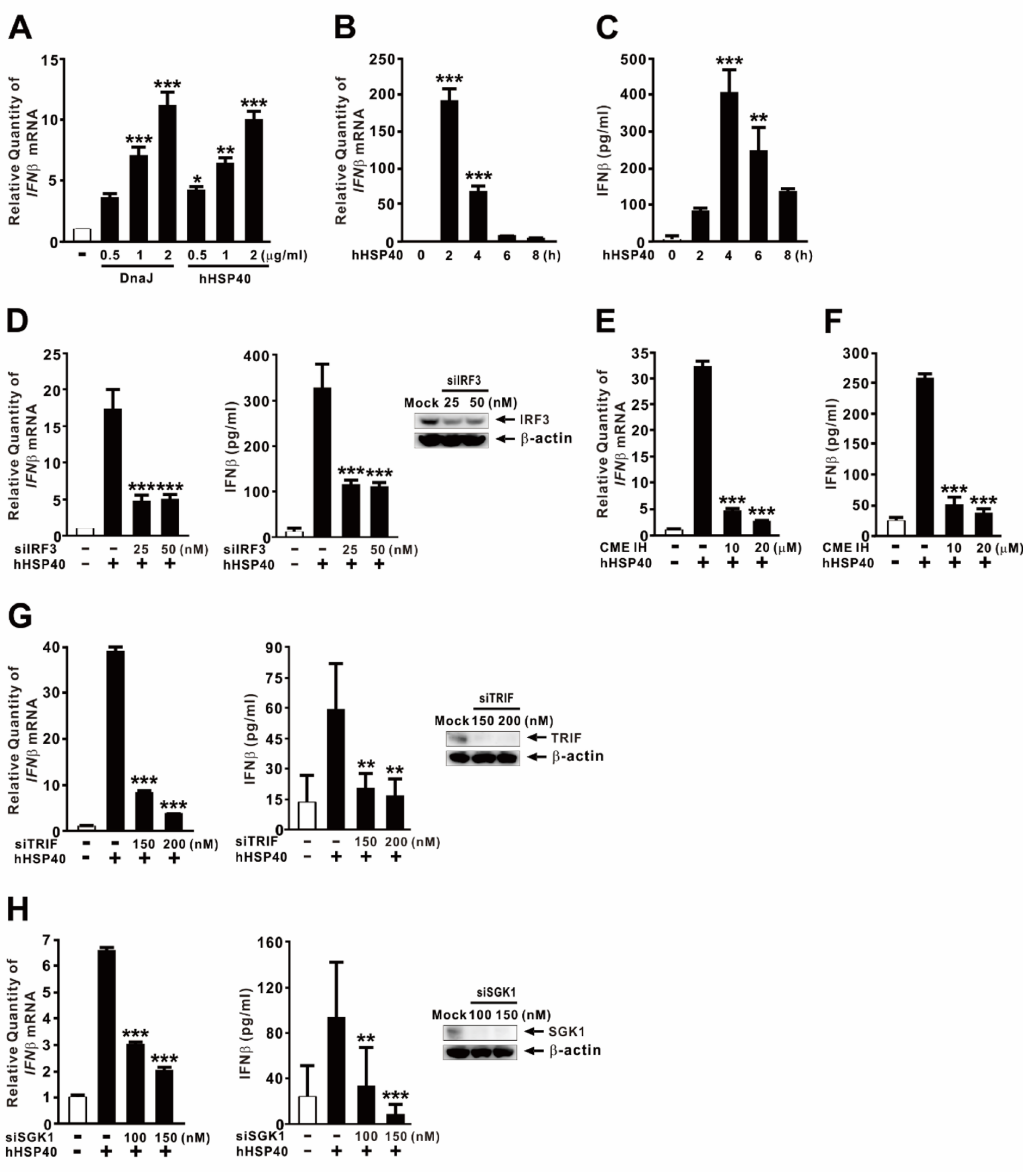
Human HSP40 induces IFNβ expression via CME-dependent SGK1-IRF3 signaling, similar to *P. aeruginosa*-derived DnaJ. (**A**–**C**) dTHP-1 cells were treated with either DnaJ or hHSP40 at the indicated concentration for 4 h (**A**) or with 1 µg/ml for the indicated durations (**B**, **C**). (**D**, **G**, **H)** Cells were transfected for 48 h with: (**D**) siIRF3, (**G**) siTRIF, or (**H**) siSGK1. Knockdown efficiency is shown in the right panels of (**D**, **G**, **H**). (**E**, **F**) Cells were pretreated with CME IH (Pitstop2) for 1 h. Following transfection or pretreatment, cells were treated with hHSP40 (1 µg/ml) for 4 h. mRNA and protein levels were measured by qPCR and ELISA, respectively; protein levels were analyzed by immunoblotting. The samples used for immunoblotting were obtained from the same experiment, and the original blots/gels are shown in the Supplementary Figure. Data are expressed as means ± SD (*n* = 3). Immunoblots are representative of three experiments. **, *p* < 0.01; ***, *p* < 0.001 vs. CON (**A**–**C**) or hHSP40 treatment alone (**D**–**H**).

Given the structural similarities between bacterial DnaJ and hHSP40^40^, we investigated whether hHSP40 could induce IFNβ expression through mechanisms similar to those employed by *P. aeruginosa*-derived DnaJ. Recombinant hHSP40 protein was expressed and purified using the sample protocol applied to DnaJ. Both DnaJ and hHSP40 induced IFNβ expression in a dose-dependent manner (Fig. 6A), with peak expression observed 2 h post-treatment (Fig. 6B). This effect was further confirmed at the protein level (Fig. 6C). To determine whether hHSP40 utilizes the same signaling pathway as DnaJ, we first examined the role of IRF3. Knockdown of IRF3 significantly reduced hHSP40-induced IFNβ expression and protein secretion (Fig. 6D, *left and middle panels*), and successful silencing was confirmed by immunoblotting (Fig. 6D, *right panel*). We next assessed the involvement of endocytosis. Pretreatment with a CME inhibitor markedly suppressed hHSP40-induced IFNβ expression (Fig. 6E) and protein secretion (Fig. 6F), mirroring the effects observed with DnaJ. To rule out the possibility that the reduction in IFNβ was due to cytotoxicity caused by the chemical inhibitor, we performed LDH release and MTT assays, which showed minimal cytotoxicity (data not shown). Finally, we evaluated the roles of TRIF and SGK1 in hHSP40-mediated signaling. Knockdown of either TRIF (Fig. 6G, *left and middle panels*) or SGK1 (Fig. 6H, *left and middle panels*) significantly diminished hHSP40-induced IFNβ expression and protein secretion. Knockdown efficiencies were verified by quantifying TRIF and SGK1 protein levels, respectively (Fig. 6G and H, *right panels*). Collectively, these results demonstrate that hHSP40 acts as a DAMP, inducing IFNβ expression through a conserved CME-dependent SGK1-IRF3 signaling pathway. These findings reveal an evolutionarily conserved role for HSP40 family members in the regulation of innate immune responses.

## Discussion

In this study, we identify DnaJ, a *P. aeruginosa*-derived homolog of HSP40, as a novel microbial inducer of IFNβ expression in macrophages. Among the bacterial HSP homologs tested, DnaJ exhibited the strongest capacity to induce IFNβ, suggesting a unique immunomodulatory role distinct from other HSP family members. Although HSPs are a conserved group of molecular chaperones found in both prokaryotic and eukaryotic cells^15,16^, accumulating evidence suggests that extracellular HSPs can function as danger signals, activating innate immune responses under conditions of cellular stress^18^. In particular, extracellular eukaryotic HSPs have been shown to initiate immune responses that contribute to the control of infections and inflammatory diseases^41,42^; for example, HSP60 has been reported to modulate inflammation when released extracellularly^43^. Consistent with these findings, we observed that hHSP40 similarly induces IFNβ expression in macrophages, mirroring the activity of *P. aeruginosa*-derived DnaJ. Given the importance of IFNβ in antimicrobial defense, it is plausible that the innate immune system has evolved to recognize bacterial HSP homologs as immunostimulatory signals.

To ensure that the observed IFNβ induction by DnaJ was not due to endotoxin contamination, DnaJ was purified from *E. coli* under endotoxin-free conditions. Residual lipopolysaccharide (LPS) was removed using Triton X-114 phase separation, as described previously^44,45^, and the final LPS concentration was confirmed by a LAL assay to be below 0.05 EU/mg of protein—an amount insufficient to trigger IFNβ expression. Given that LPS is known to induce type I and type II IFN production in macrophages^46,47^ and can activate IFNβ expression in THP-1 monocytes^48^, it was essential to eliminate any confounding effects of LPS. Importantly, DnaJ selectively induced IFNβ expression in macrophages but not in monocytes. Other bacterial HSP homologs (GroEL, DnaK, HtpG), purified using the same procedure, failed to induce IFNβ, further supporting the specificity of the response to DnaJ. Additionally, proteinase K treatment abolished the IFNβ-inducing activity and knockdown of CD14 and MD2 did not alter the DnaJ-mediated IFNβ expression, verifying that the effect was mediated by the DnaJ protein itself.

Mechanistically, DnaJ-induced IFNβ expression is mediated through the activation of IRF3, which requires upstream signaling via TLR4 and the adaptor protein TRIF. IRF3 is a key transcription factor involved in IFNβ induction downstream of both the TLR3-TRIF and TLR4-TRIF/MyD88 pathways^30,31^. DnaJ treatment resulted in a downregulation of TLR3, which may act as a negative feedback mechanism to limit excessive IFNβ production, further supporting TLR4 as the primary receptor in this context. Interestingly, DnaJ also induced differential regulation of TLR3 and TLR7, with a notable upregulation of TLR7 expression^25^. However, since TLR7 signals through a MyD88-dependent but TRIF-independent pathway^49^, it is unlikely to mediate the observed IRF3 activation. In addition to TLR4, other receptors such as CD40, CD91, and TLR10 have been proposed to participate in DnaJ signaling^27,28^. Among these, TLR10 and CD40 did not influence IFNβ induction, while CD91 showed a modest positive effect (data not shown). Nevertheless, CD91 knockdown had minimal impact on SGK1 or IRF3 activation, suggesting that CD91 is not essential for DnaJ-induced IFNβ production. Although IRF7, which interacts with IRF3, is known to contribute to the positive feedback regulation of IFNβ downstream of TLR4-TRIF/MyD88 signaling^50–52^, our data indicate that IRF7 is not involved in responses mediated by either DnaJ or hHSP40 (data not shown). Collectively, these findings suggest that DnaJ is primarily recognized by TLR4 and induces IFNβ expression in macrophages via IRF3-dependent signaling.

Moreover, CME is required for DnaJ-induced IRF3 activation and subsequent IFNβ expression, consistent with previous studies showing that TRIF-dependent TLR4 signaling relies on endosomal internalization^32,53^. We further demonstrated that while AKT signaling contributes to IFNβ induction, it is dispensable for IRF3 activation, indicating that it functions outside the canonical TLR4-TRIF-TBK1-IRF3 axis. This contrasts with a previous report in which AKT was shown to act downstream of TBK1 and to promote IRF3 activation and IFNβ expression in response to poly(I: C) and LPS, agonists of TLR3 and TLR4, respectively^54^. The discrepancy may be attributed to differences in stimuli, highlighting the unique signaling properties of DnaJ, which is recognized by multiple receptors including CD40, CD91, and TLR10. In addition, we found that inhibition of NF-κB suppressed DnaJ-induced IFNβ expression (data not shown), suggesting potential positive crosstalk between AKT and NF-κB, as previously reported^55,56^. Instead of AKT, we identified SGK1 as a critical mediator of this response. Inhibition or knockdown of SGK1 significantly reduced both IRF3 phosphorylation and IFNβ expression, consistent with previous findings showing that DnaJ activates TLR2 signaling via the SGK1 pathway^28^.

Interestingly, endogenous SGK1 levels were altered by treatment with DnaJ and chemical inhibitors, consistent with previous reports indicating that SGK1 transcription can be rapidly induced by various stimuli, while phosphorylation-dependent activation of pre-existing SGK1 protein occurs concurrently^35–37^. Moreover, SGK1 production and activation was not affected by inhibition of TRIF or MyD88, suggesting that SGK1 functions independently of these adaptor proteins or may act upstream of them. In contrast, SGK1 production and activation was abrogated by CME inhibition, indicating that SGK1 operates within a CME-dependent signaling axis that ultimately leads to IRF3 activation. Notably, hHSP40 also induced IFNβ expression via this CME-SGK1-IRF3 pathway, suggesting that both microbial and host-derived HSP40s serve as immunomodulatory ligands, potentially acting as DAMPs or PAMPs depending on the context. These findings support the existence of an evolutionarily conserved mechanism by which HSP40 family members regulate type I IFN responses.

**Fig. 7:**
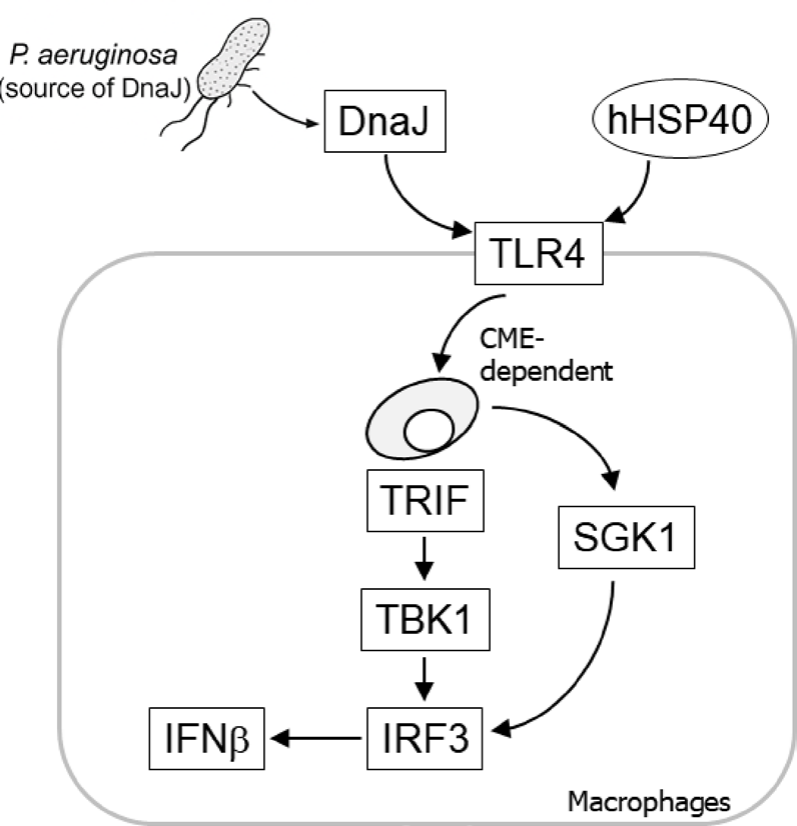
A schematic illustration depicting the proposed mechanism underlying DnaJ-mediated induction of IFNβ production.

TLR4 is unique among TLRs in that it signals through both TRIF- and MyD88-dependent pathways^30,57^. Our findings support the notion that the TLR4-TRIF-TBK1-IRF3 axis serves as the primary pathway for DnaJ-induced IFNβ expression, consistent with previous reports in the context of *P. aeruginosa* infection^8^. However, inhibition of IRAK4—a kinase that integrates TLR4-driven MyD88 and TRIF signaling^57^—as well as pharmacological inhibition of TLR4 using CLI-095, which blocks recruitment of both MyD88 and TRIF^58^, resulted in a marked reduction of IFNβ levels. Similarly, selective inhibition of MyD88 partially reduced IFNβ expression. While the MyD88 pathway is classically associated with NF-κB-mediated proinflammatory cytokine production^57^, the IFNβ enhancer is also regulated by the cooperative binding of NF-κB, IRF3, and ATF-2/c-Jun^59^. In addition to the contributions of NF-κB and IRF3, we found that JNK and p38 MAP kinases also promoted IFNβ expression, with JNK playing a more prominent role in IRF3 activation. In contrast, ERK did not appear to be involved (data not shown). Although AKT has previously been reported to inhibit JNK and p38 MAPK through suppression of apoptosis signal-regulated kinase 1 (ASK1) in L929 fibroblasts^60^, our data indicate that in macrophages, both AKT and these MAPKs are positive regulators of IFNβ induction. These differences likely reflect cell type-specific regulatory mechanisms. Additionally, while MyD88 has been shown to negatively regulate TLR3-mediated IFNβ production^61^, this is unlikely to be relevant in our system, as DnaJ treatment downregulated TLR3 expression. Taken together, our results suggest that DnaJ-induced IFNβ expression is primarily mediated through the canonical TLR4-TRIF-IRF3 pathway (Fig. 7), but is also modulated by a parallel TLR4-MyD88-PI3K signaling axis, which was previously reported^62^. This dual-pathway engagement likely serves to fine-tune the magnitude and timing of IFNβ responses during the early phase of innate immune activation.

As the first line of defense, the innate immune system relies on cytokines such as type I IFNs to initiate protective responses against invading pathogens. Although recombinant IFNs are used clinically, their therapeutic efficacy is often limited by a short half-life and systemic toxicity. Consequently, strategies that promote endogenous production of type I IFNs have emerged as attractive alternatives for enhancing host defense. Synthetic TLR ligands, for instance, have been proposed as next-generation adjuvants for vaccines and cancer immunotherapy^63^. In this context, we hypothesized that bacterial HSPs—which are structurally homologous to eukaryotic DAMPs and are secreted rather than membrane-bound—may serve as innate immune modulators^19–27^. As demonstrated in this study, *P. aeruginosa*-derived DnaJ acts as a potent immunomodulatory ligand that promotes CME of TLR4, leading to robust IFNβ induction and enhancement of innate immune responses. Notably, DnaJ also upregulated TLR7 expression^25^, a receptor involved in antiviral immunity, suggesting a potential role in enhancing macrophage antiviral functions. Collectively, these findings support the potential application of DnaJ as an immunomodulatory agent, either alone or in combination with antiviral therapies.

## Methods

### Reagents

Proteinase K, LY294002 (PI3K inhibitor), GSK2334470 (PDK1 inhibitor), and Compound 26 (IRAK4 inhibitor) were purchased from Thermo Fisher Scientific (Waltham, MA, USA), Cell Signaling Technology (Danvers, MA, USA), APExBio (Houston, TX, USA), Merck Millipore (Burlington, MA, USA), respectively. OxPAPC (TLR2/4 inhibitor) and CLI-095 (TLR4 inhibitor) were purchased from InvivoGen (San Diego, CA, USA). T6167923 (MyD88 inhibitor), MRT67307 (TBK1 inhibitor), and MK-2206 (AKT inhibitor) were purchased from MedChemExpress (Monmouth Junction, NJ, USA). Pitstop2 (CME inhibitor) and GSK650394 (SGK1 inhibitor) were purchased from Selleckchem (Houston, TX, USA).

### Cell culture

Media described below were supplemented with 10% heat-inactivated fetal bovine serum (FBS; HyClone, Rockford, IL, USA), penicillin (100 units/ml) and streptomycin (0.1 mg/ml). Human monocyte-derived THP-1 cells were maintained in Roswell Park Memorial Institute-1640 medium (RPMI-1640; HyClone). Differentiation into macrophage-like cells was induced by treatment with 50 nM phorbol-12-myristate-13-acetate (PMA) for 48 h, followed by a 24 h rest period. These differentiated cells are referred to as dTHP-1 cells. Cells were maintained at 37 °C in a humidified incubator with 5% CO_2_.

### Construction of the plasmids to express His-tagged DnaJ and human HSP40

The coding regions for the *DnaJ* (PA4760) and *hHSP40* loci were amplified from genomic DNA derived from *P. aeruginosa* strain PAK and from THP-1 complementary DNA (cDNA), respectively, using polymerase chain reaction (PCR) with the following primers: *His*-tagged *DnaJ* plasmid, 5’-G*GAATTC*GATGGCGAAACGAGACT-3’ and 5’-CAC*AAGCTT*CGGCTGTCAGGCCGC-3’ (which contain restriction enzyme recognition sites for *Eco*RI and *Hin*dIII); *His*-tagged *hHSP40* plasmid, 5’-CG*GGATCC*GATGGGTAAAGACTACTACC-3’ and 5’-AA*CTGCAG*GTCCAGAAATCCTTGAGC-3’ (which contain restriction enzyme recognition sites for BamHⅠ and PstⅠ). The resultant 1.2 kb and 1.082 kb PCR products were cloned into the pETDuet-1 vector (Novagen, Germany) using the restriction sites incorporated into the primer sets. The resultant plasmids, designated pJW18001 and pDK22002, respectively, were transformed into *E. coli* BL21 (DE3) cells (Invitrogen, Carlsbad, CA, USA).

###  Purification of DnaJ and hHSP40 recombinant proteins

DnaJ and hHSP40 were purified using the Ni-NTA purification system (Thermo Fisher Scientific). Briefly, the bacterial cell suspension was sonicated on ice 600 times at 150 watts for 1 s at 5-sec intervals using a Digital Sonifier (Branson Ultrasonics, Danbury, CT, USA). Residual intact cells were removed by centrifugation at 20,000 g for 30 min at 4 °C. The bacterial lysate was incubated with Ni-NTA resin (Thermo Fisher Scientific) at 4 °C for 1 h, and then washed sequentially with increasing concentrations of imidazole (20, 40, and 60 mM) in purification buffer (20 mM Tris-HCl, 300 mM NaCl, pH 7.0). The bound protein was eluted with 200 mM imidazole in the same buffer. Phase separation treatment with Triton X-114 was used to remove the residual endotoxin^45^. The concentration of the remaining endotoxin was < 0.05 EU/mg protein, which was determined using the Limulus amebocyte lysate (LAL) Chromogenic Endotoxin Quantitation Kit (Pierce Thermo, Rockford, IL, USA). Protein concentrations were determined by the Bradford method using a Protein Assay Kit (Bio-Rad. Hercules, CA, USA) with bovine serum albumin as the standard protein. The purified protein was stored at -80 ℃ at 500 µg/ml. The same purification protocol was used for *E. coli* BL21 (DE3) cells harboring a pETDuet-1 vector to obtain a control extract. The control extract was used to evaluate the effect of DnaJ and hHSP40 throughout the study.

### Real-time quantitative PCR (qPCR)

The primer sequences used in this study are listed in Table 1. For primer validation, a melt curve analysis was conducted after qPCR to ensure a single peak, indicating a single amplicon. Total RNA was extracted using TRIzol^®^ Reagent (Invitrogen, Carlsbad, CA, USA), following the manufacturer’s instructions. To eliminate genomic DNA contamination, cDNA was synthesized from total RNA using the ReverTra Ace qPCR RT Master Mix with genomic DNA (gDNA) Remover (Toyobo, Japan), which is designed to efficiently remove gDNA through DNase I treatment before starting the reverse transcription process. qPCR was performed using SYBR Green PCR Master Mix (KAPA Biosystems, Woburn, MA, USA) on the CFX96 Real-Time PCR System (Bio-Rad, Hercules, CA, USA). The Thermal cycling conditions were as follows: stage 1, 50 °C for 2 min and 95 °C for 10 min; stage 2, 95 °C for 15 s and 60 °C for 1 min, repeated for 40 cycles. Relative mRNA quantities were calculated using the comparative CT method and normalized against the corresponding levels of human GAPDH.

**Table 1:** qPCR primer sequences.

Genes	Forward (5’ to 3’)	Reverse (5’ to 3’)
*IFNα*	TGGCACAGATGAGGAGAA	AGGACAGGGATGGTTTCA
*IFNβ*	TCCCTGAGGAGATTAAGCAGCT	GGAGCATCTCATAGATGGTCAATG
*IFNγ*	CAGGTCATTCAGATGTAGCG	GCTTTTCAGAGTCATCTCG
*TLR3*	GATCTGTCTCATAATGGCTTG	GACAGATTCCGAATGCTTGTG
*TLR4*	TGGATACGTTTCCTTATAAG	GAAATGGAGGCACCCCTTC
*GAPDH*	CCCTCCAAAATCAAGTGG	CCATCCACAGTCTTCTGG

### Immunoblotting analysis

Cells were lysed on ice for 10 min using a mammalian lysis buffer containing 20 mM Tris-HCl (pH 7.4), 50 mM NaCl, 50 mM sodium pyrophosphate, 30 mM sodium fluoride, 5 µM zinc chloride, 2 mM iodoacetic acid, 1% Triton X-100, 1 mM phenylmethylsulfonyl fluoride, and 0.1 mM sodium orthovanadate (Sigma-Aldrich). Lysates were centrifuged at 20,000×*g* for 20 min at 4 °C, and the resulting supernatant-containing the total cytosolic protein-was collected. Protein concentration was measured using the bicinchoninic acid (BCA) Protein Assay Kit (Thermo Fisher Scientific, Waltham, MA, USA). Approximately 15 µg of each protein sample was separated on an 8% SDS-PAGE gel and transferred to a 0.45 μm polyvinylidene difluoride (PVDF) membrane. Membranes were blocked with 5% bovine serum albumin for 2 h at room temperature, followed by overnight incubation at 4 °C with primary antibodies against IRF3, p-IRF3 (S386), p-IRF3 (S396), TLR2, TLR3, TLR4, TLR10, TRIF, AKT, p-AKT (S473), SGK1, p-SGK1, and β-actin (Cell Signaling Technology, Danvers, MA, USA). After washing, membranes were incubated with horseradish peroxidase (HRP)-conjugated anti-rabbit secondary antibodies for 2 h at room temperature. Protein detection was performed using SuperSignal West Femto Maximum Sensitivity Substrate (Thermo Scientific) and visualized on an Amersham ImageQuant-800 (Cytiva, Marlborough, MA, USA).

### siRNA transfection

IRF3 siRNA (siIRF3; #sc-35710), siTLR2 (#sc-40256), siCD14 (#sc-29248), and siMD2 (#sc-35889) were purchased from Santa Cruz Biotechnology (Dallas, TX, USA). siTLR10, siTRIF, and siSGK1 were purchased from Bioneer (Daejeon-si, Korea). dTHP-1 macrophage cells (5 × 10^5^ cells) were transfected with siRNA or a negative control siRNA-A (#sc-37007) at 20–200 nM using Lipofectamine RNAiMax (Invitrogen). After 48 h of transfection, cells were cultured in RPMI-1640 medium with 5% FBS for an additional 72 h to achieve 80% confluence. qPCR was performed after 24 h of incubation to confirm siRNA knockdown efficiency, either by measuring mRNA levels via qPCR or protein levels via immunoblotting.

### Enzyme-linked immunosorbent assay (ELISA)

The amount of IFNβ released into the supernatant was measured using the human IFNβ ELISA kit (R&D Systems, Minneapolis, MN, USA) following the manufacturer’s instructions. The concentration of IFNβ was determined using a standard curve generated with recombinant IFNβ.

### Statistical analysis

Results are expressed as the mean ± standard deviation (SD). Student’s *t*-test or one-way ANOVA, followed by Tukey’s post-hoc multiple range test with GraphPad Instat software (version 3.06; San Diego, CA, USA), was used for statistical analysis. A *p*-value < 0.05 was considered statistically significant.

## Supplementary Information

Below is the link to the electronic supplementary material.

Supplementary Material 1

Supplementary Material 2

Supplementary Material 3

## Data Availability

The datasets generated during and/or analyzed during the current study are available from the corresponding author upon reasonable request.

## Abbreviations

CME: Clathrin-mediated endocytosis
DAMPs: Damage-associated molecular patterns
HSP40: Heat shock protein 40
LAL: Limulus amebocyte lysate
PAMPs: Pathogen-associated molecular patterns
MyD88: Myeloid differentiation primary response 88
PRRs: Pattern recognition receptors
SGK1: Serum/glucocorticoid regulated kinase 1
TBK1: TANK-binding kinase 1
PDK1: Phosphoinositide-dependent kinase 1
TRIF: Toll-interleukin 1 (IL-1) receptor homology (TIR) domain-containing adaptor inducing IFN-β
IFNs: Type I interferons
