# Supplementary figures and images for: Pseudomonas aeruginosa-derived DnaJ functions as a novel immunomodulator inducing IFNβ via CME–SGK1–IRF3 axis in macrophages
Source: Sci Rep. 2025 Dec 3;16:1386. doi: 10.1038/s41598-025-31281-x (PMC12796343; doi:10.1038/s41598-025-31281-x)

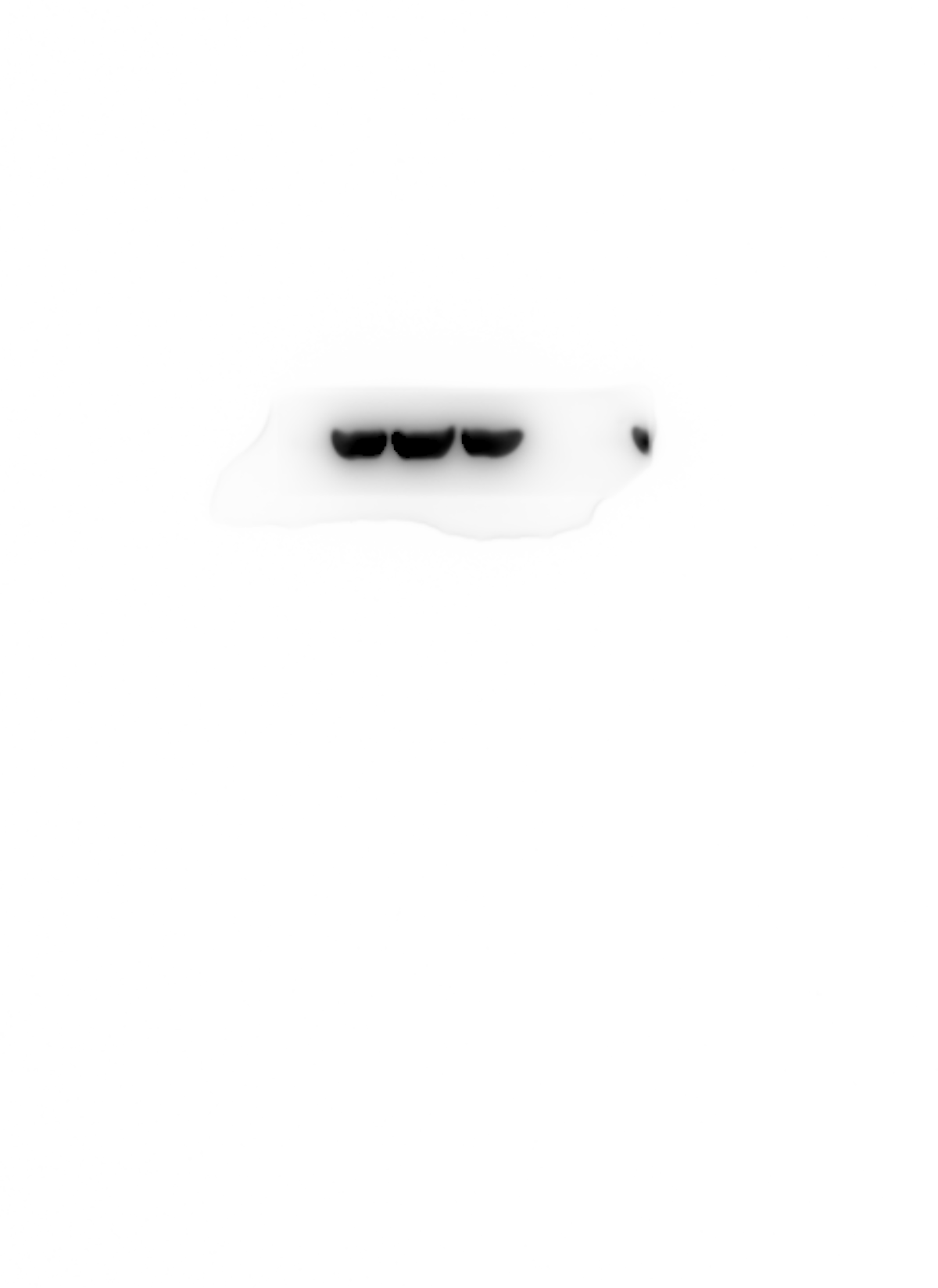

Supplement: Supplementary file 1 — Supplementary Material 1 [file 41598_2025_31281_MOESM1_ESM.zip › Fig1_R1/Fig.1G b-actin.tif]

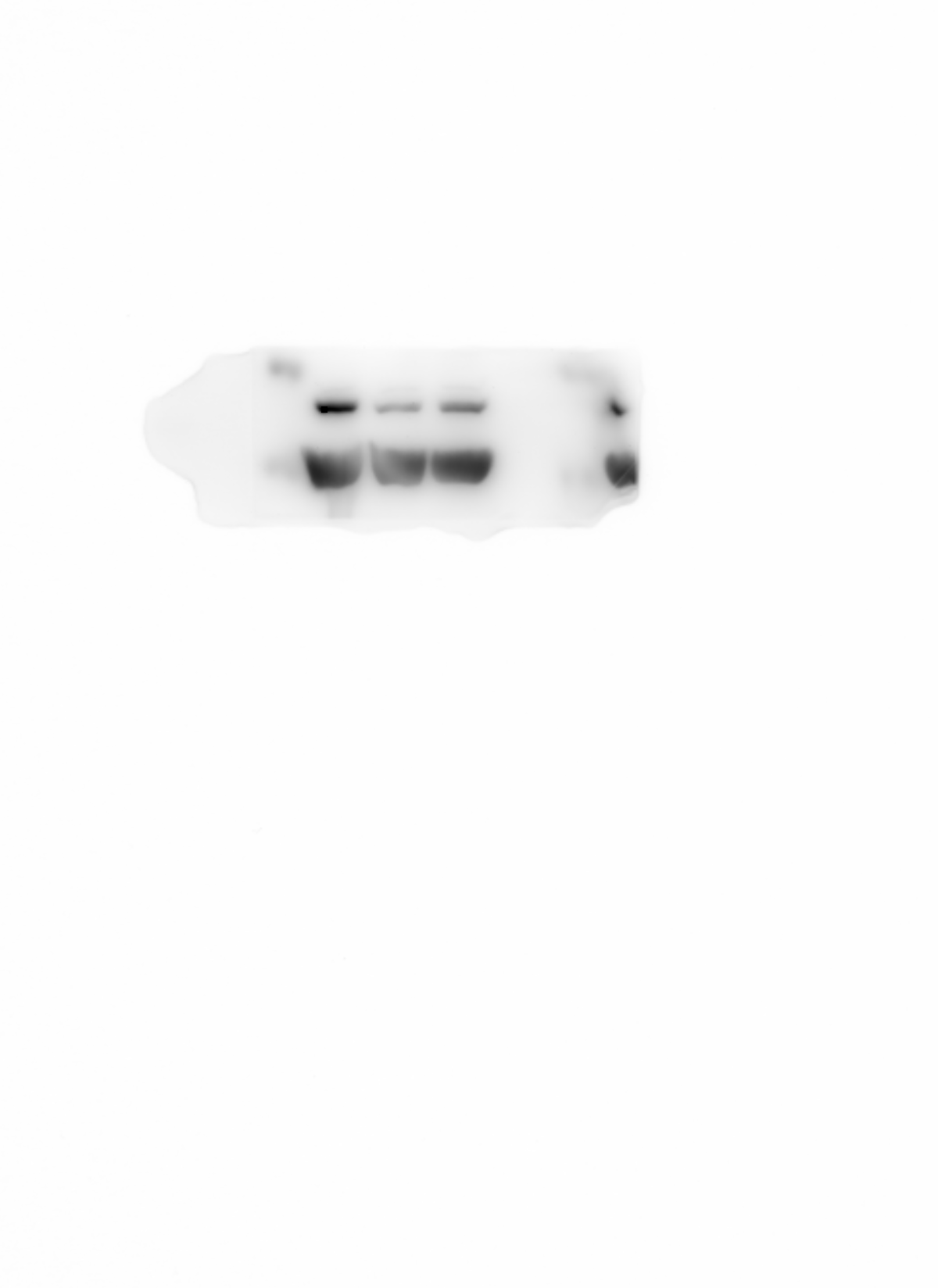

Supplement: Supplementary file 1 — Supplementary Material 1 [file 41598_2025_31281_MOESM1_ESM.zip › Fig1_R1/Fig.1G IRF3.tif]

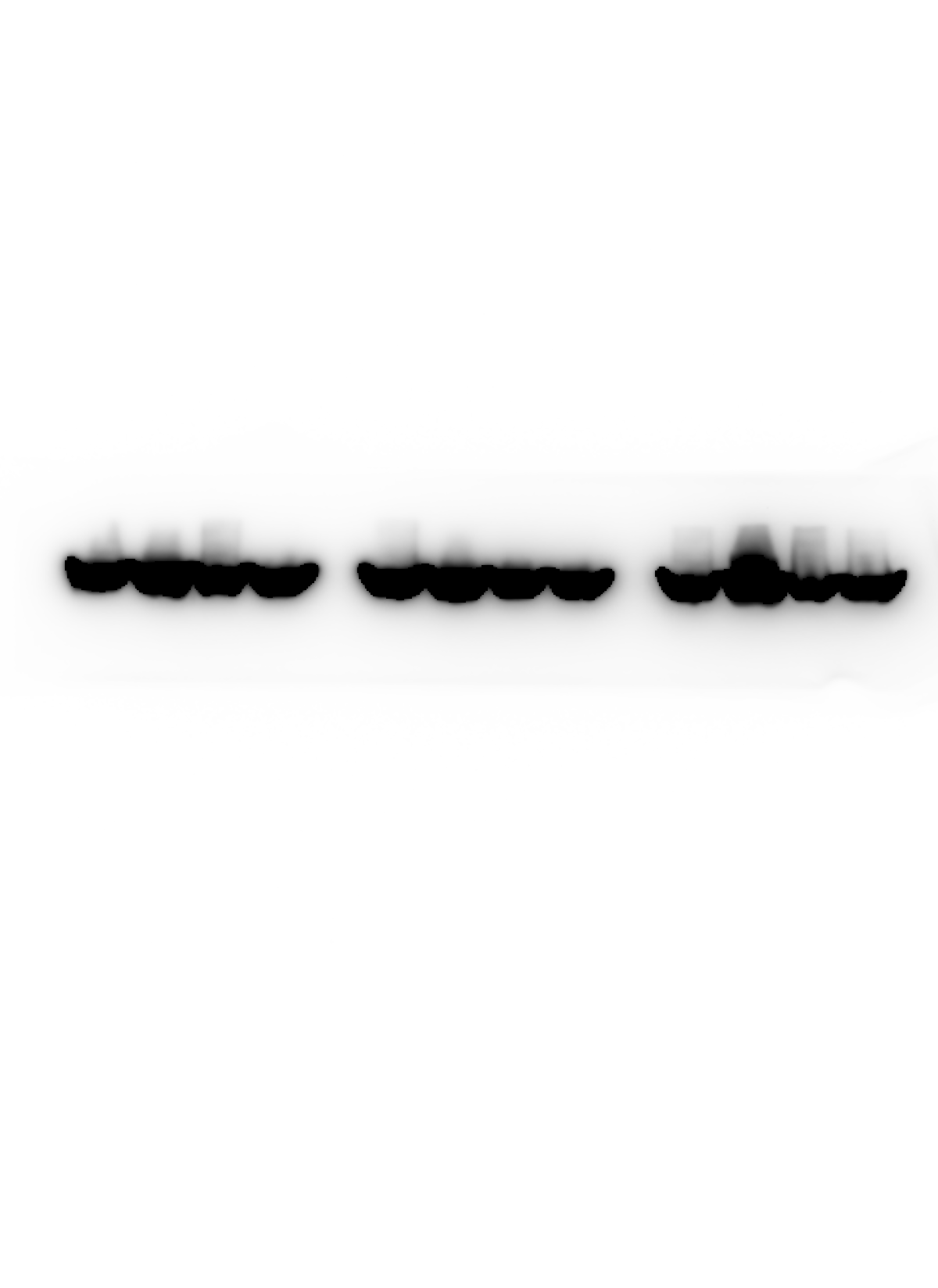

Supplement: Supplementary file 1 — Supplementary Material 1 [file 41598_2025_31281_MOESM1_ESM.zip › Fig1_R1/Fig.1H b-actin (Mock).tif]

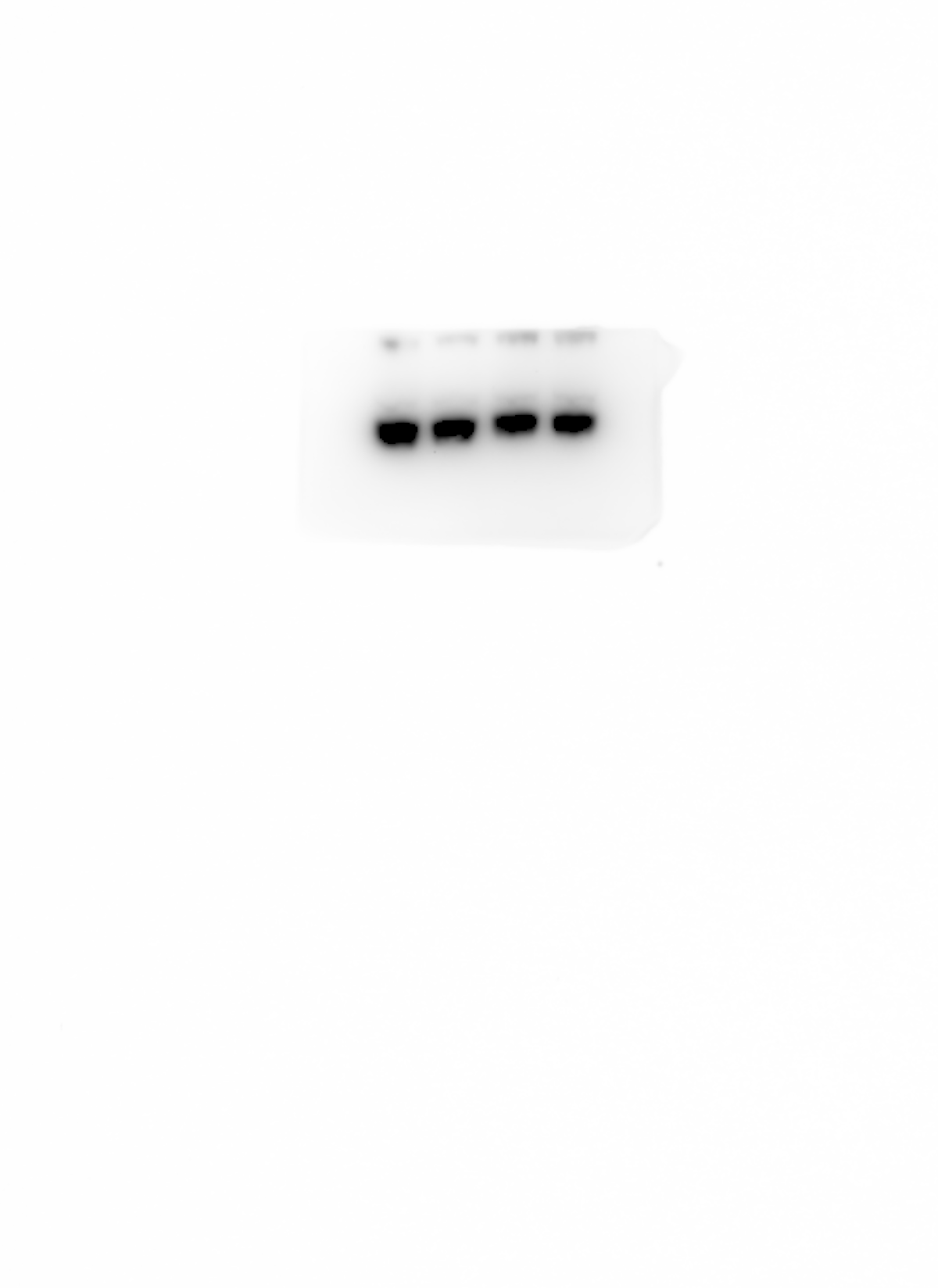

Supplement: Supplementary file 1 — Supplementary Material 1 [file 41598_2025_31281_MOESM1_ESM.zip › Fig1_R1/Fig.1H IRF3.tif]

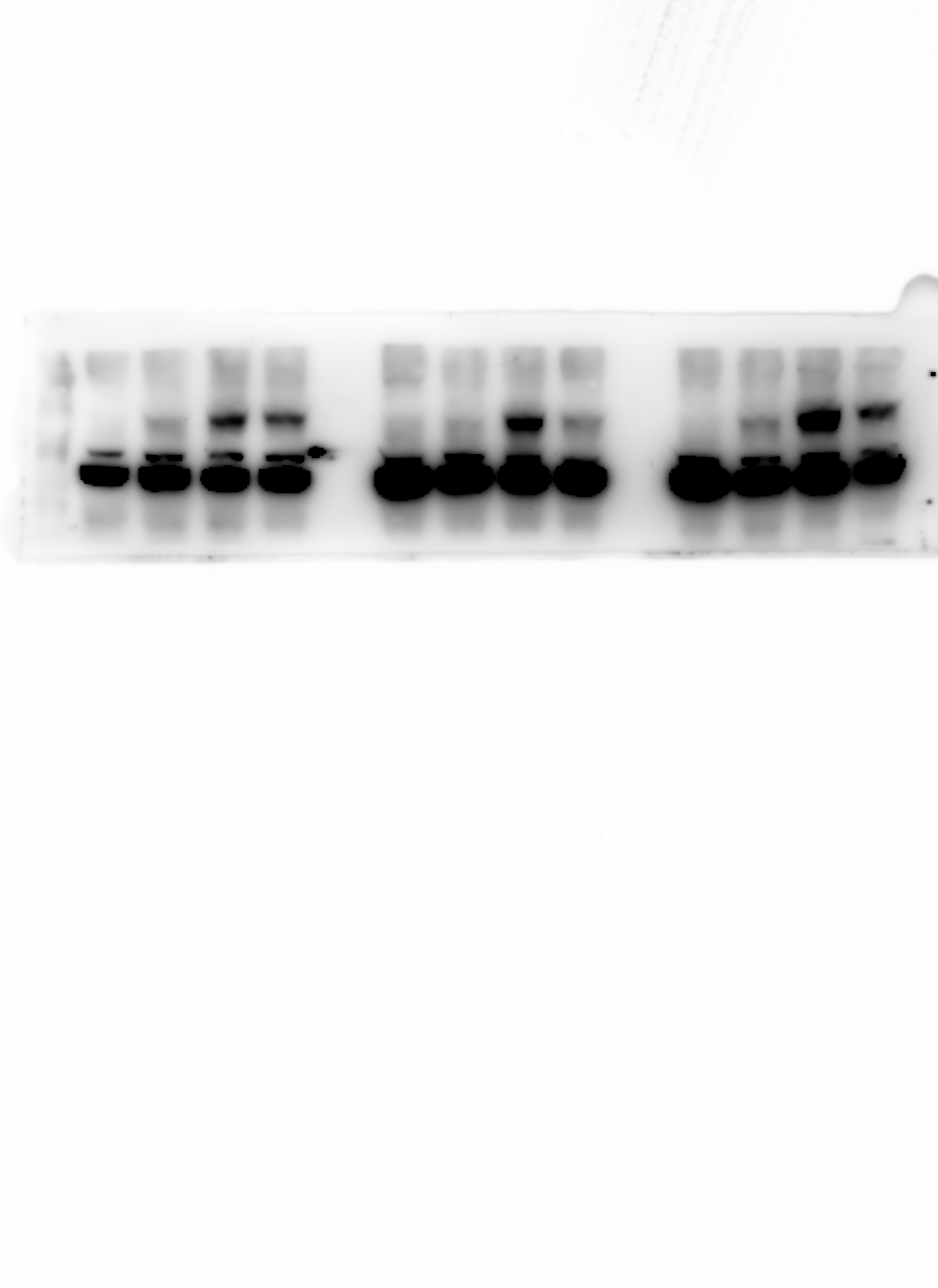

Supplement: Supplementary file 1 — Supplementary Material 1 [file 41598_2025_31281_MOESM1_ESM.zip › Fig1_R1/Fig.1H p-IRF3(S396) (Mock).tif]

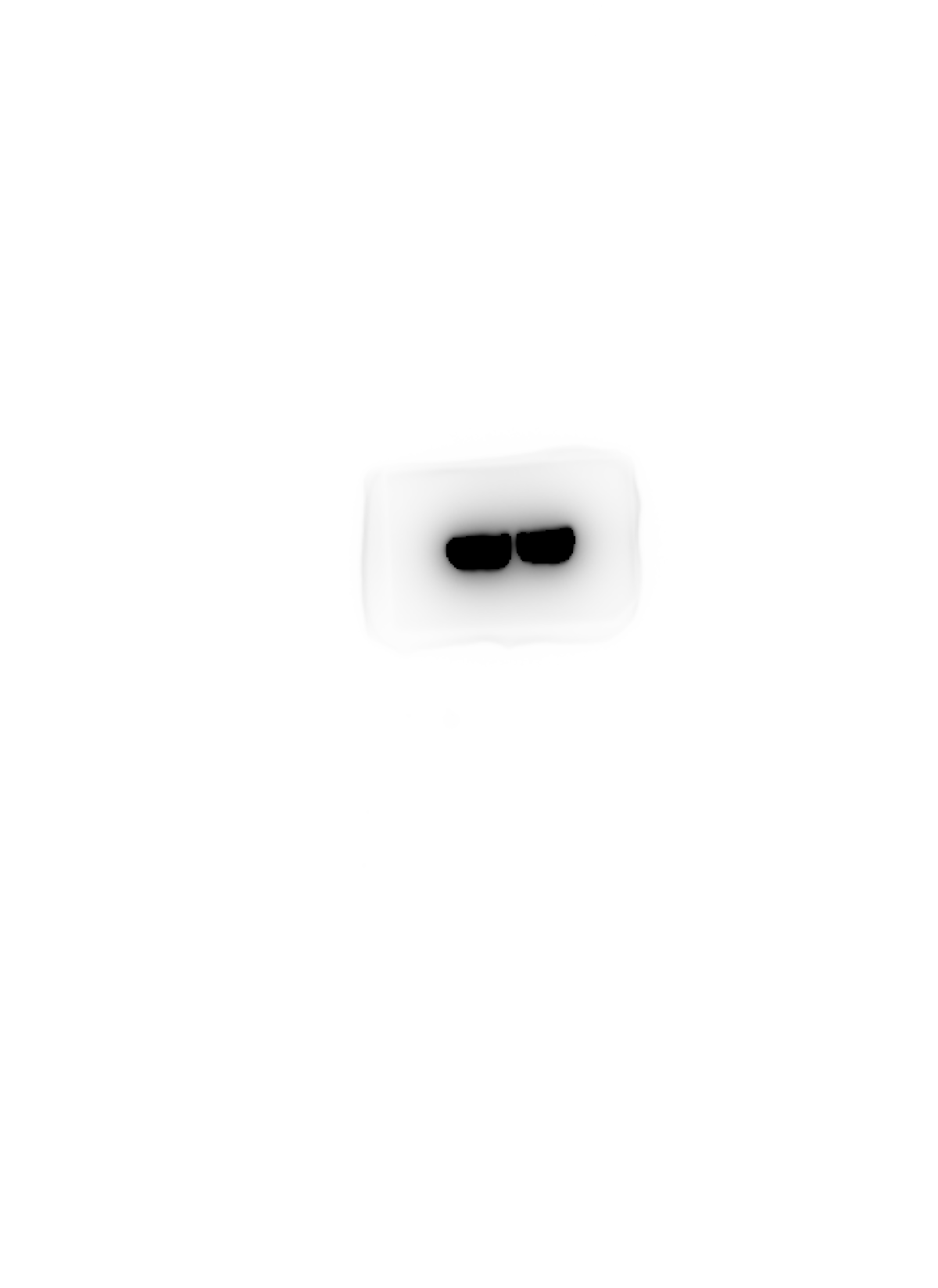

Supplement: Supplementary file 1 — Supplementary Material 1 [file 41598_2025_31281_MOESM1_ESM.zip › Fig2_R1/Fig.2C b-actin.tif]

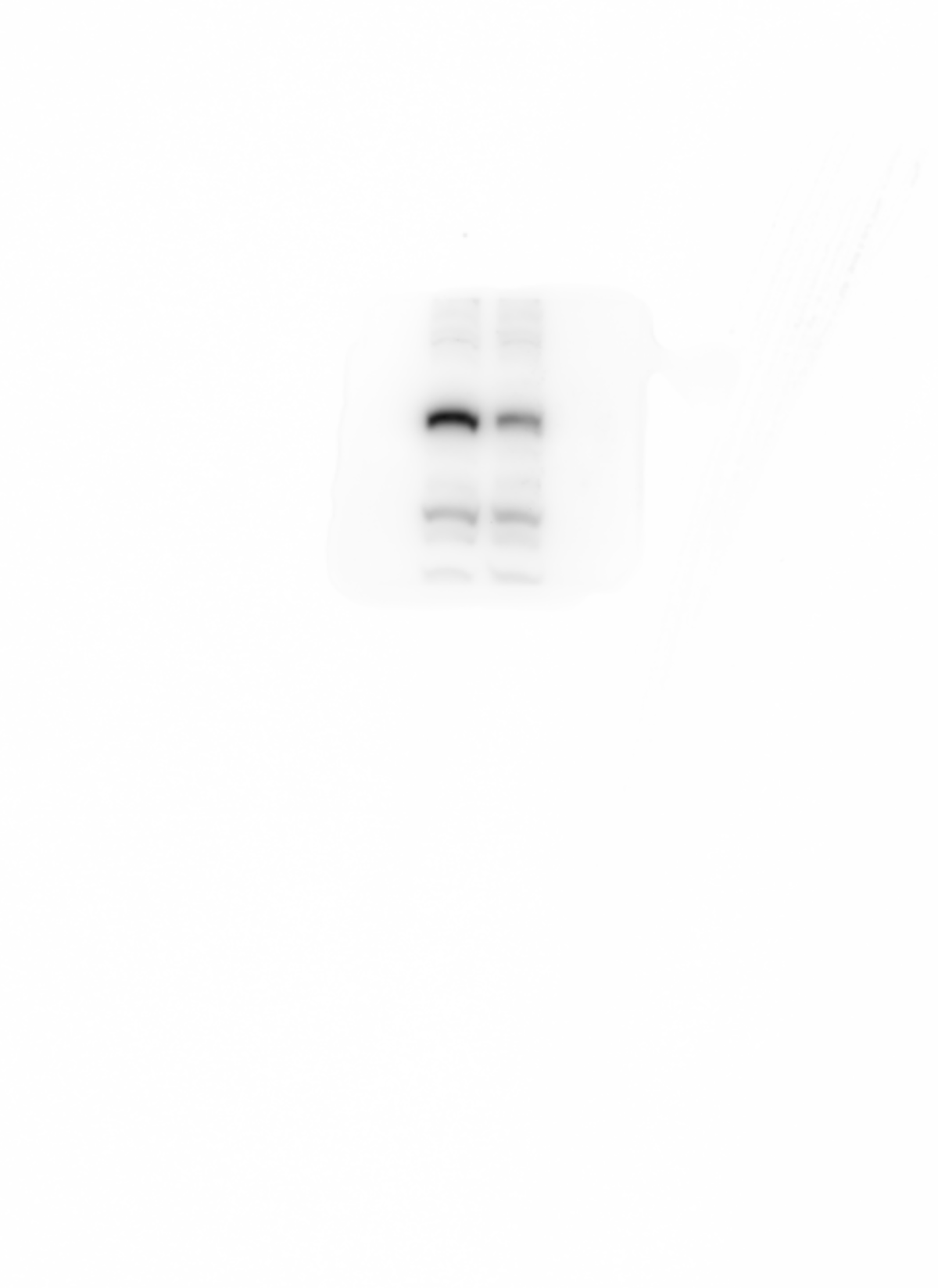

Supplement: Supplementary file 1 — Supplementary Material 1 [file 41598_2025_31281_MOESM1_ESM.zip › Fig2_R1/Fig.2C TLR10.tif]

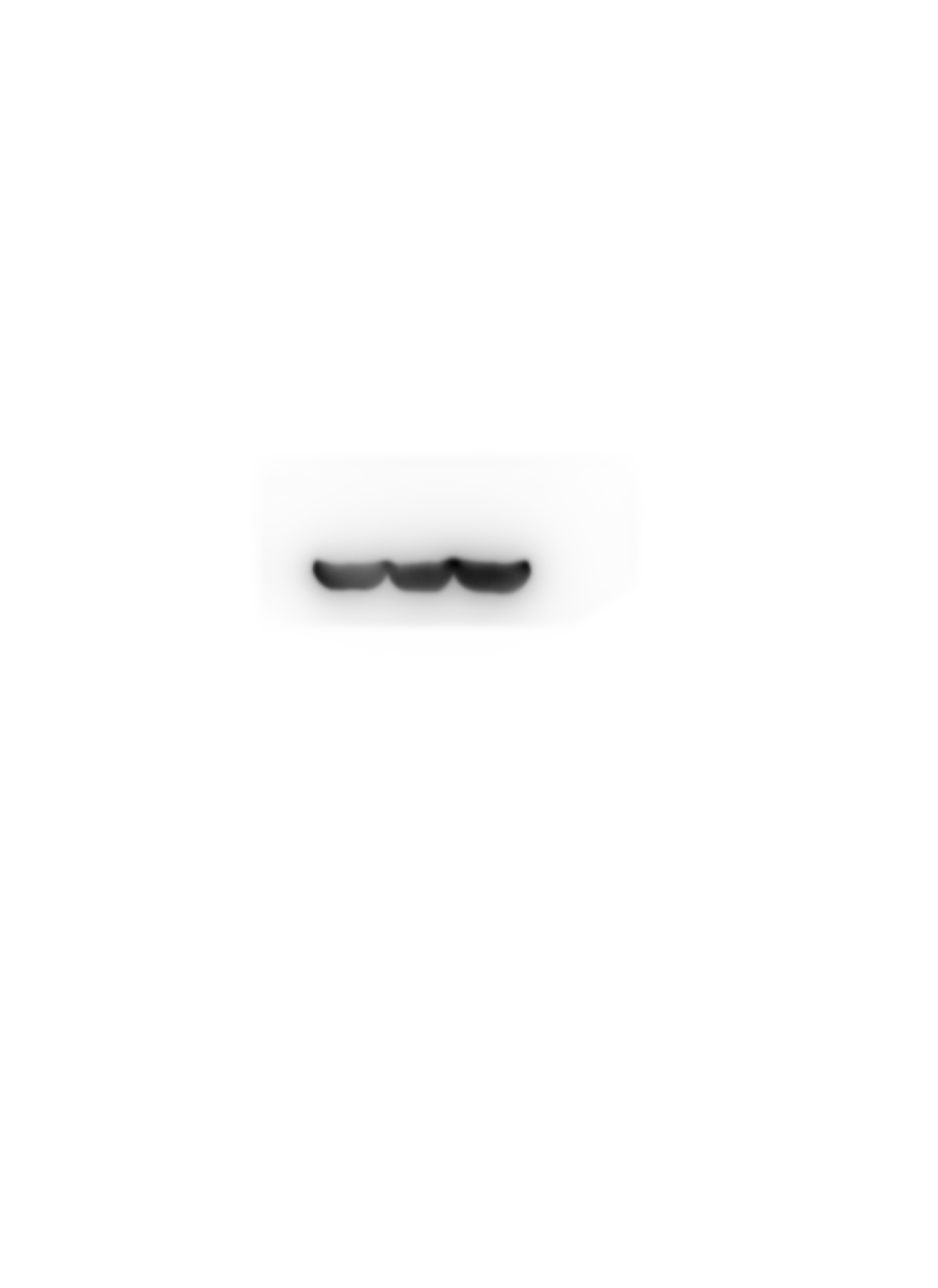

Supplement: Supplementary file 1 — Supplementary Material 1 [file 41598_2025_31281_MOESM1_ESM.zip › Fig2_R1/Fig.2E b-actin.tif]

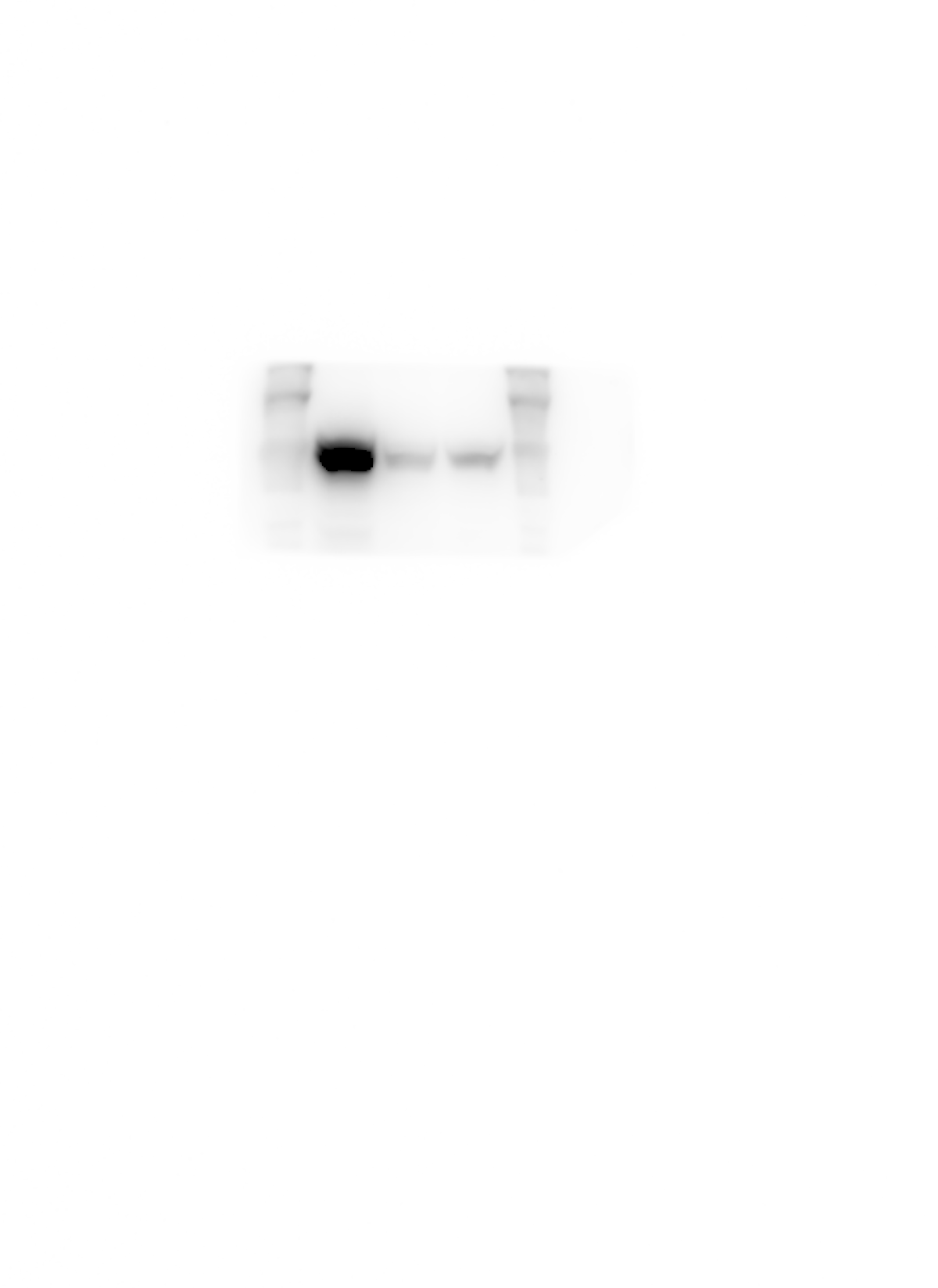

Supplement: Supplementary file 1 — Supplementary Material 1 [file 41598_2025_31281_MOESM1_ESM.zip › Fig2_R1/Fig.2E TLR2.tif]

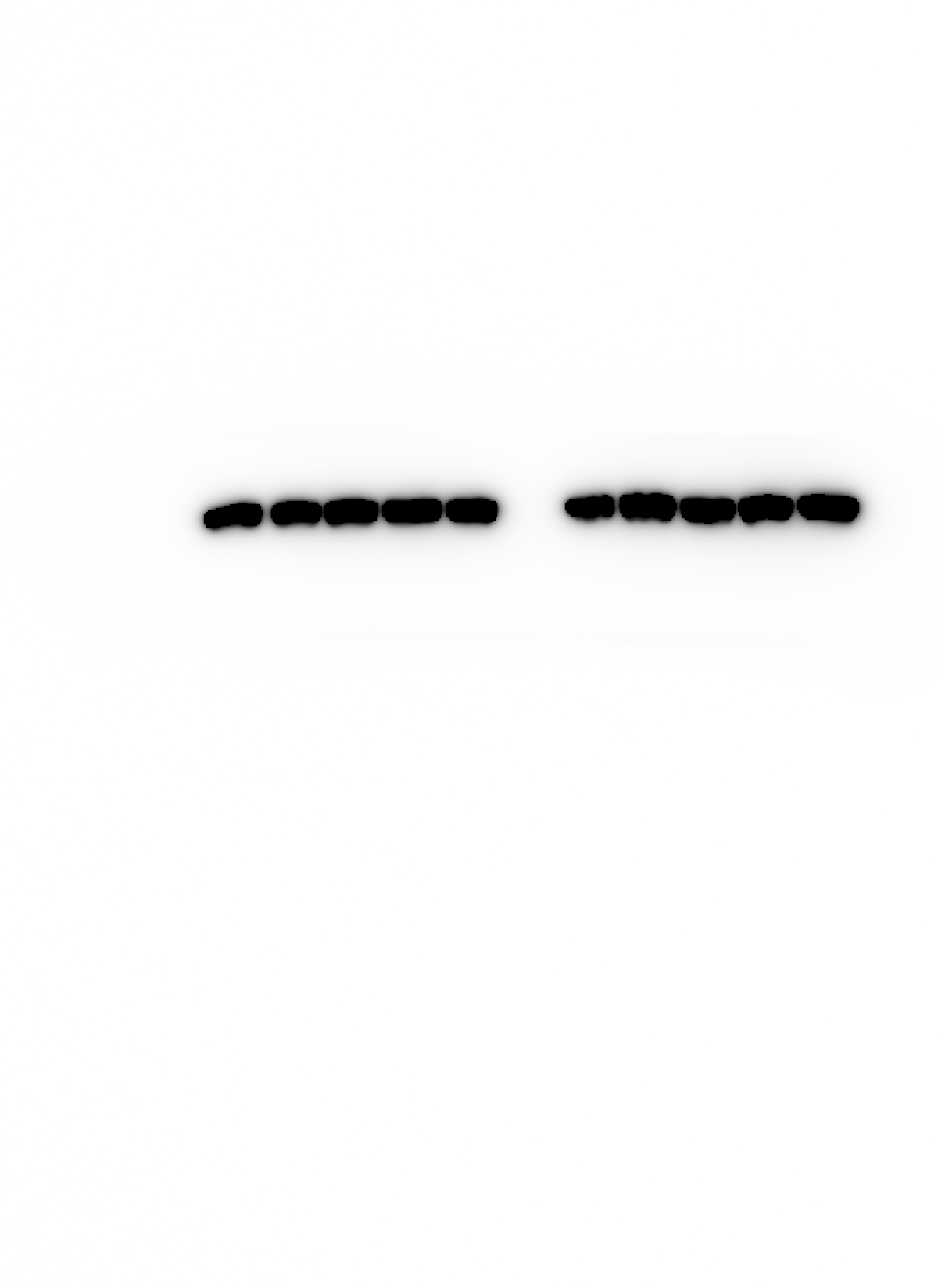

Supplement: Supplementary file 1 — Supplementary Material 1 [file 41598_2025_31281_MOESM1_ESM.zip › Fig2_R1/Fig.2H b-actin.tif]

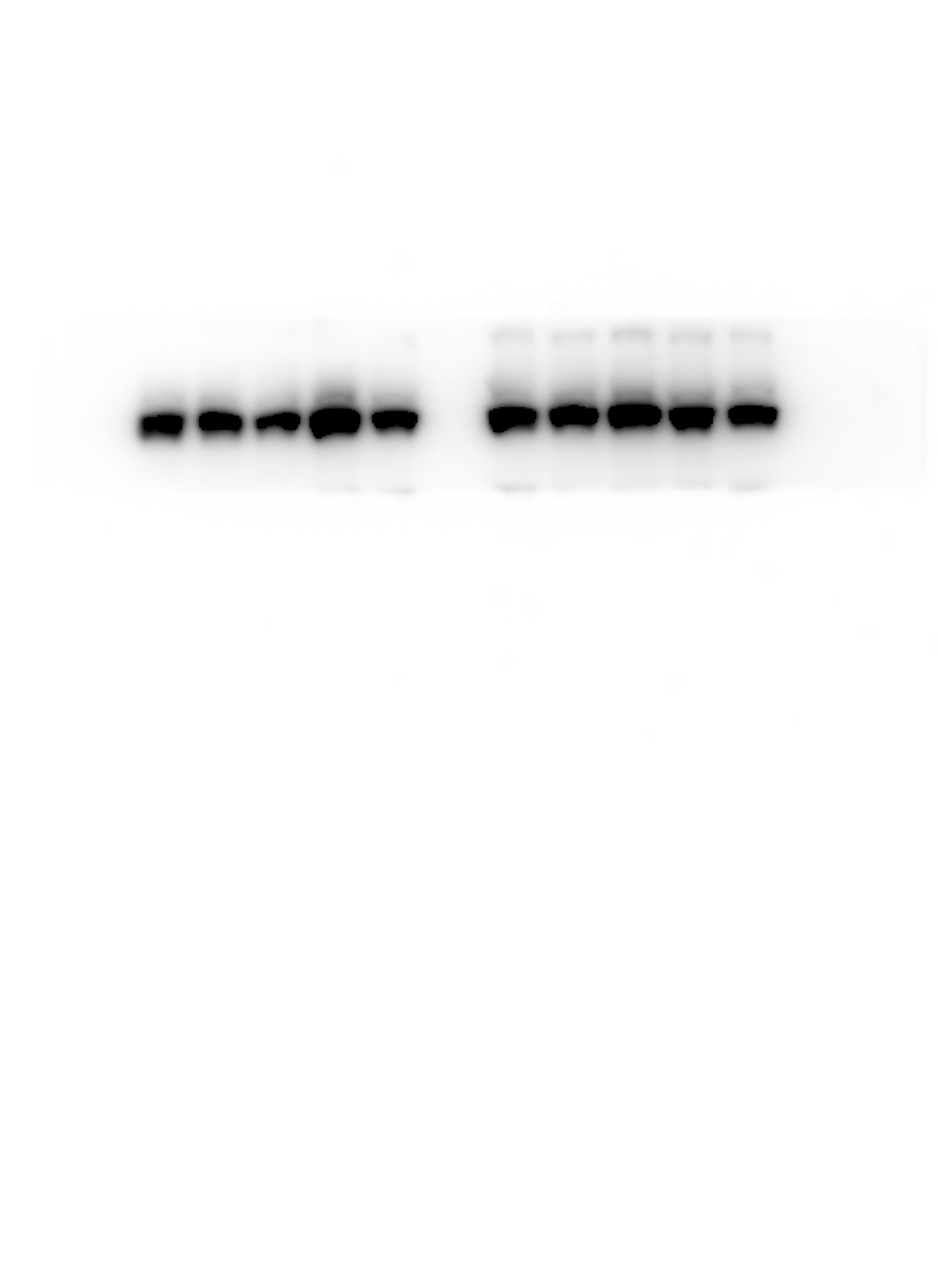

Supplement: Supplementary file 1 — Supplementary Material 1 [file 41598_2025_31281_MOESM1_ESM.zip › Fig2_R1/Fig.2H IRF3.tif]

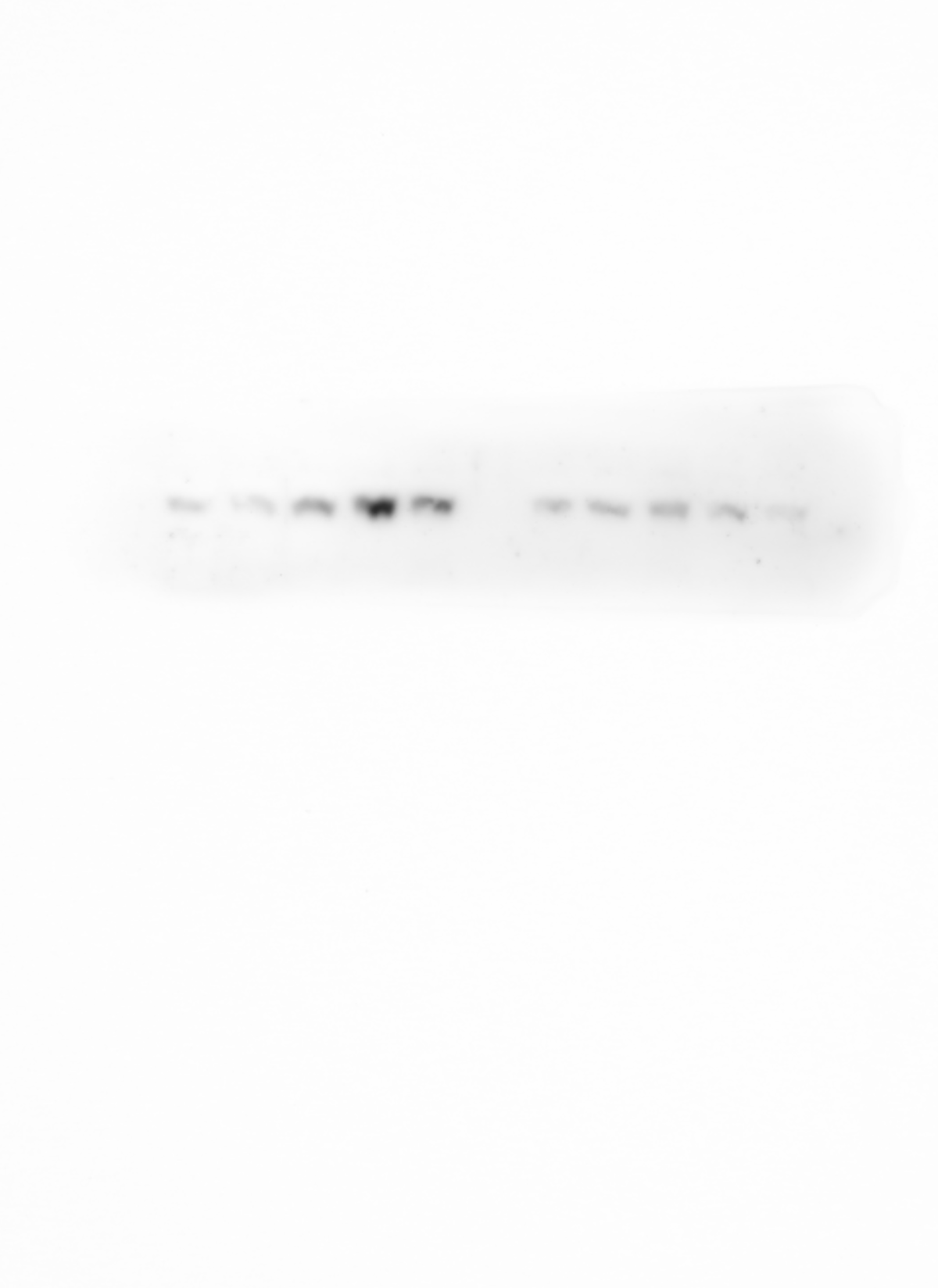

Supplement: Supplementary file 1 — Supplementary Material 1 [file 41598_2025_31281_MOESM1_ESM.zip › Fig2_R1/Fig.2H p-IRF3(S386).tif]

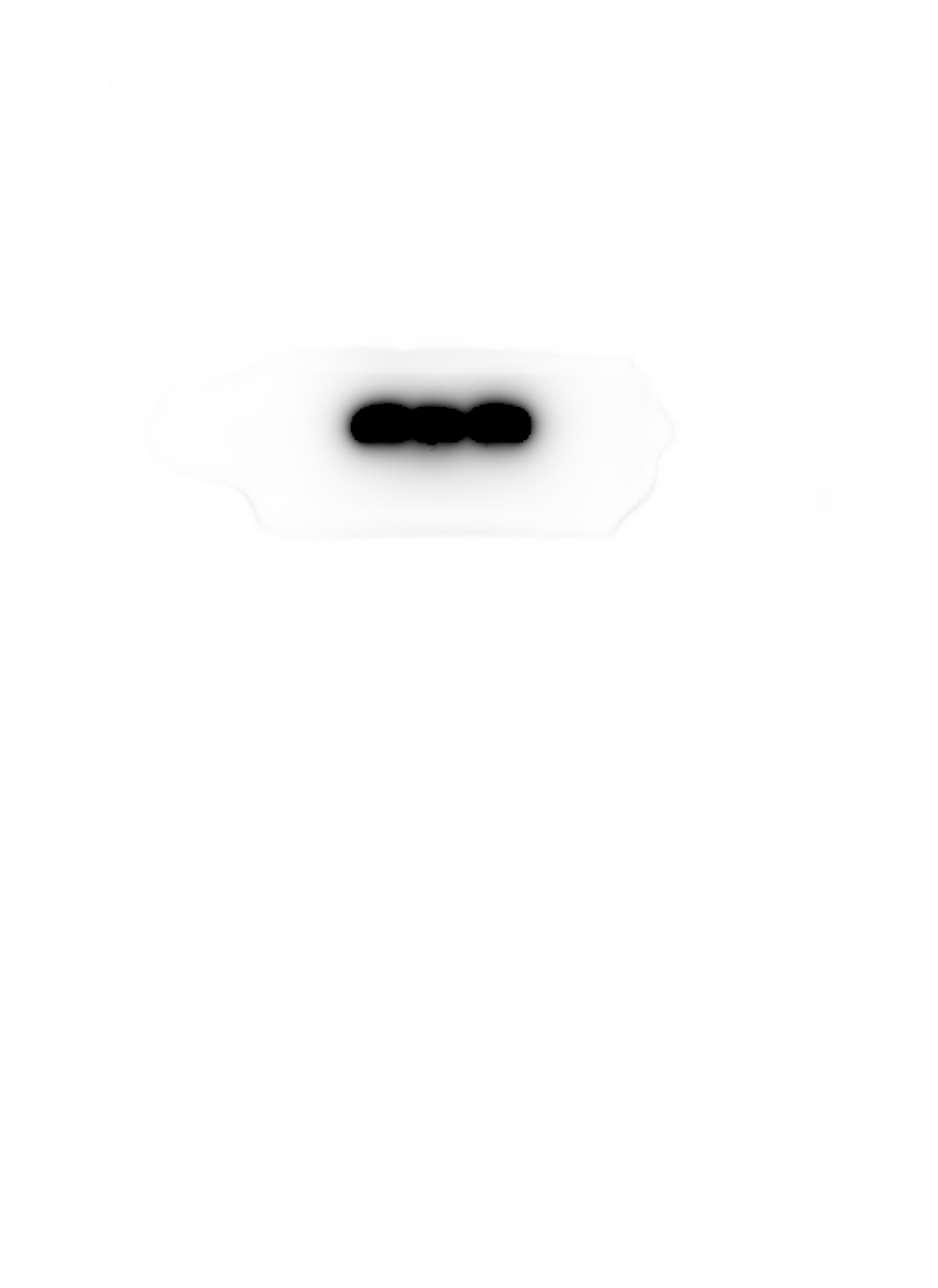

Supplement: Supplementary file 1 — Supplementary Material 1 [file 41598_2025_31281_MOESM1_ESM.zip › Fig3_R1/Fig.3A b-actin.tif]

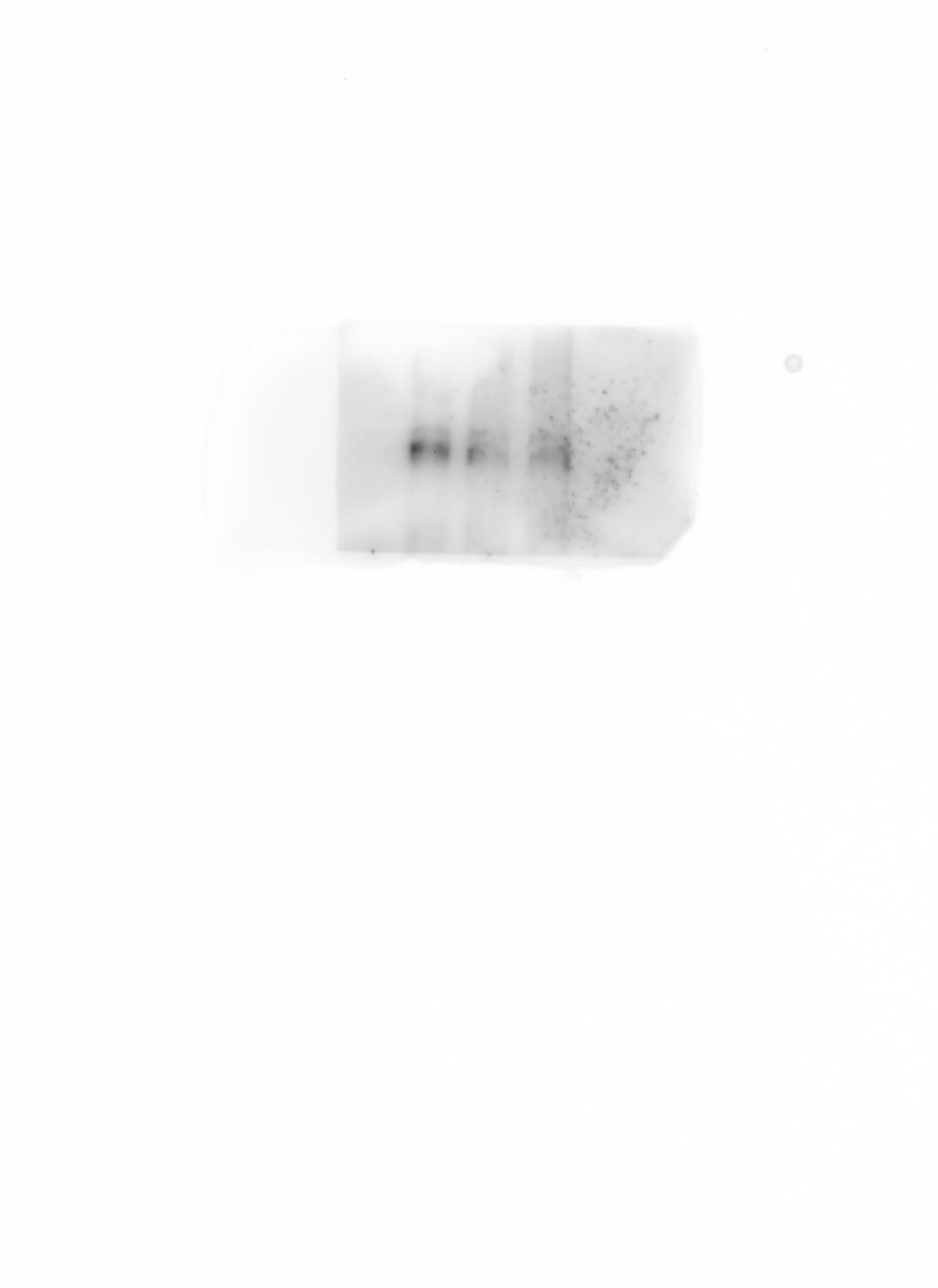

Supplement: Supplementary file 1 — Supplementary Material 1 [file 41598_2025_31281_MOESM1_ESM.zip › Fig3_R1/Fig.3A TRIF.tif]

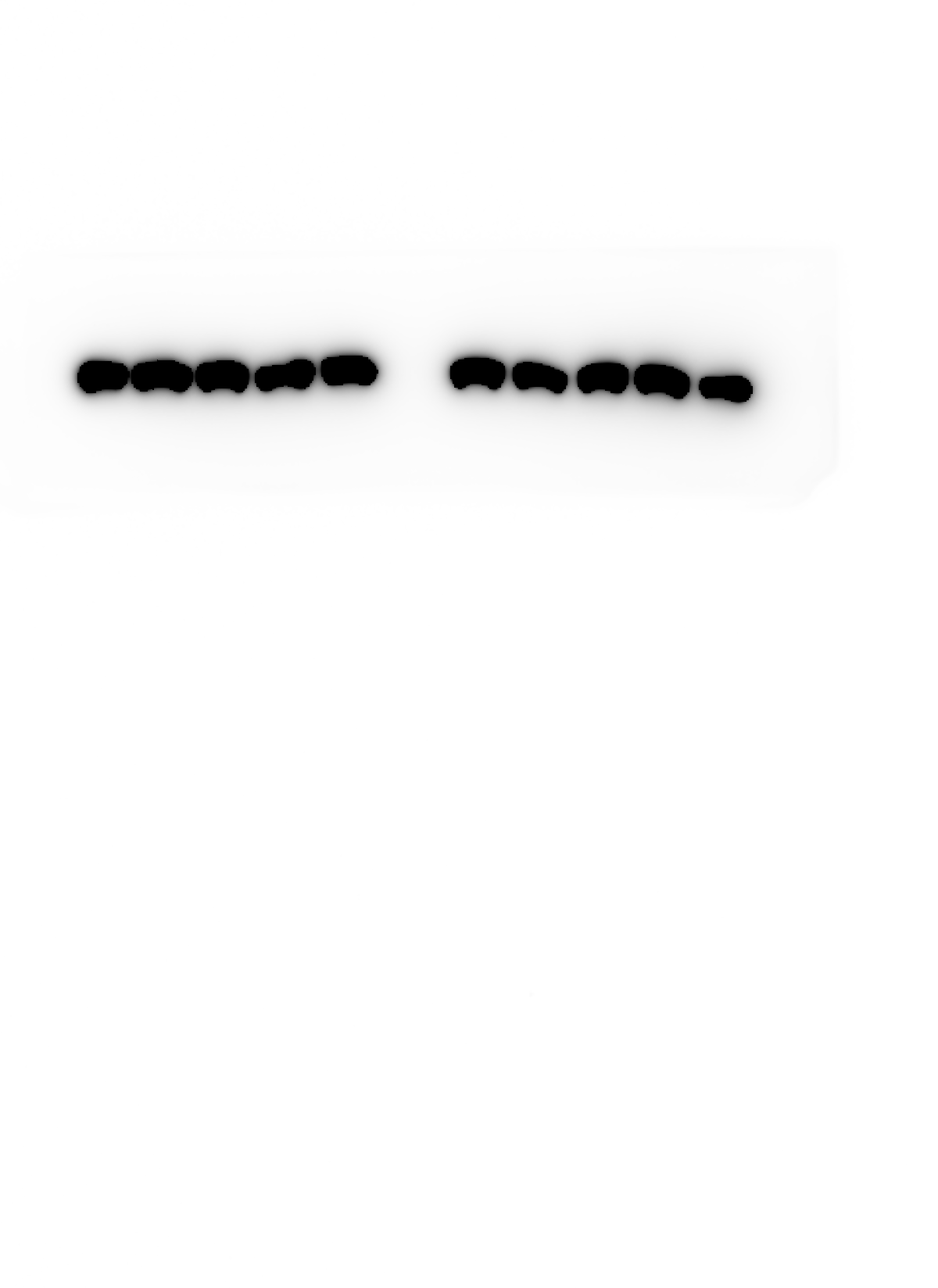

Supplement: Supplementary file 1 — Supplementary Material 1 [file 41598_2025_31281_MOESM1_ESM.zip › Fig3_R1/Fig.3B b-actin.tif]

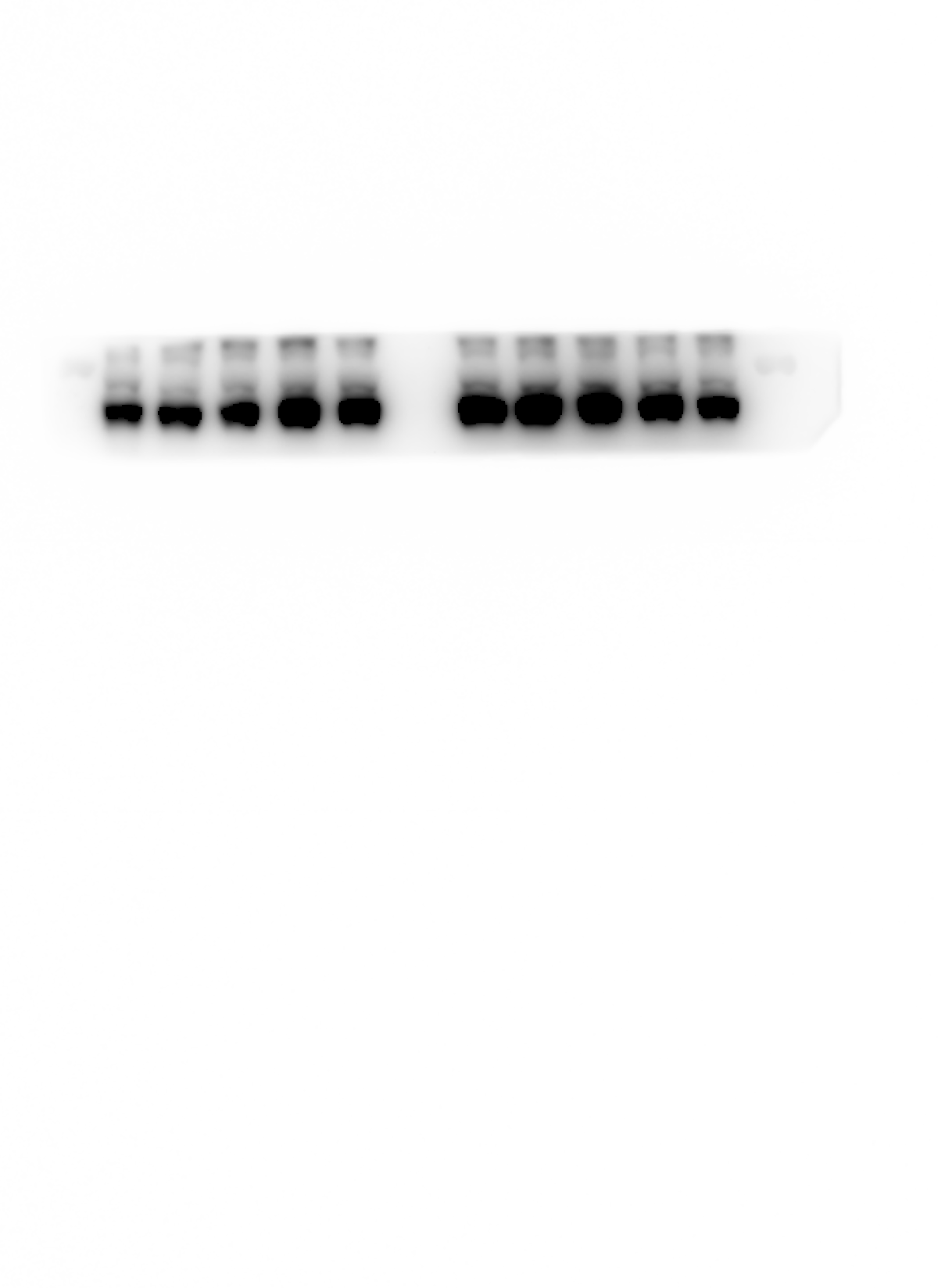

Supplement: Supplementary file 1 — Supplementary Material 1 [file 41598_2025_31281_MOESM1_ESM.zip › Fig3_R1/Fig.3B IRF3.tif]

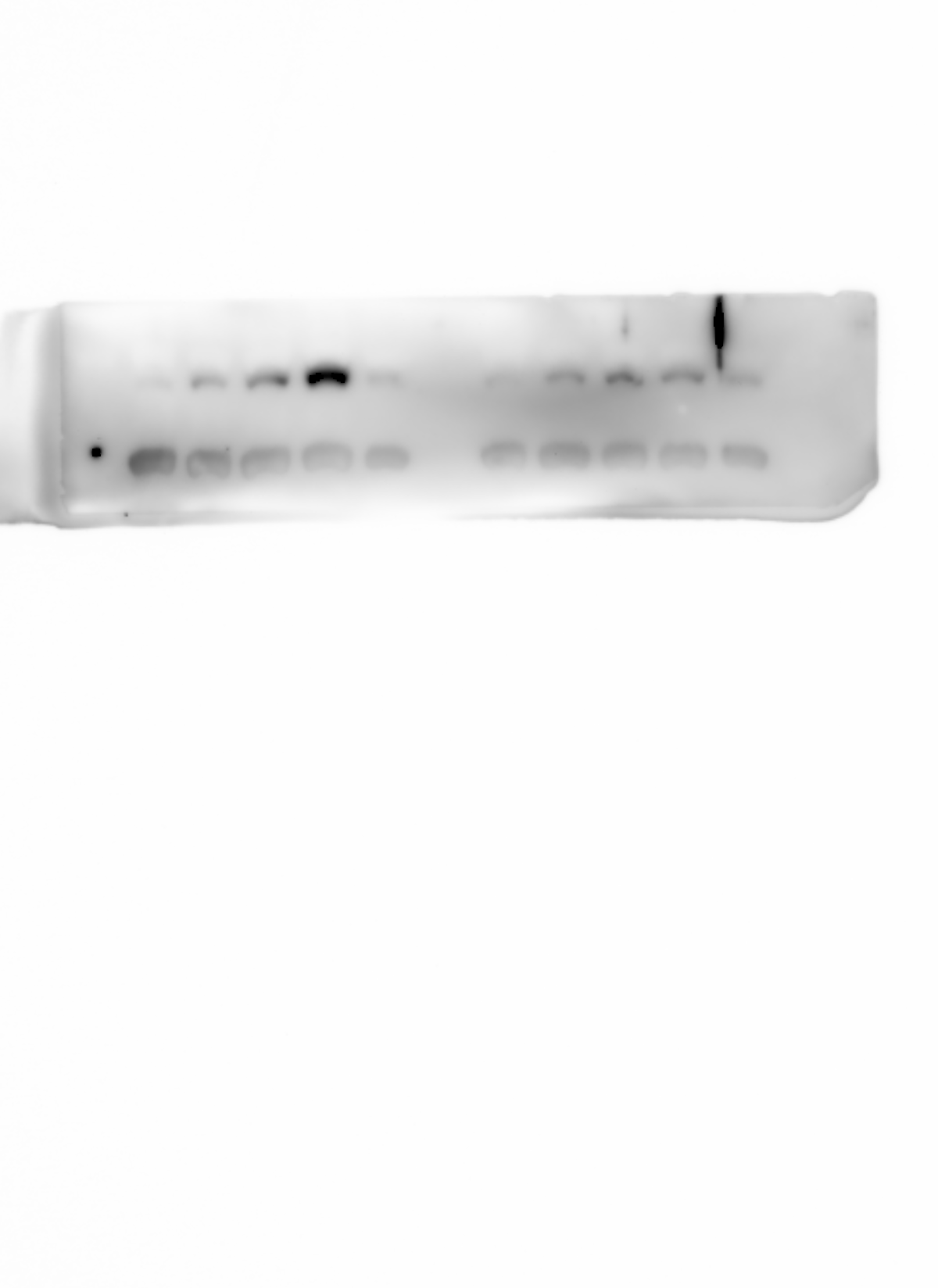

Supplement: Supplementary file 1 — Supplementary Material 1 [file 41598_2025_31281_MOESM1_ESM.zip › Fig3_R1/Fig.3B p-IRF3(S386).tif]

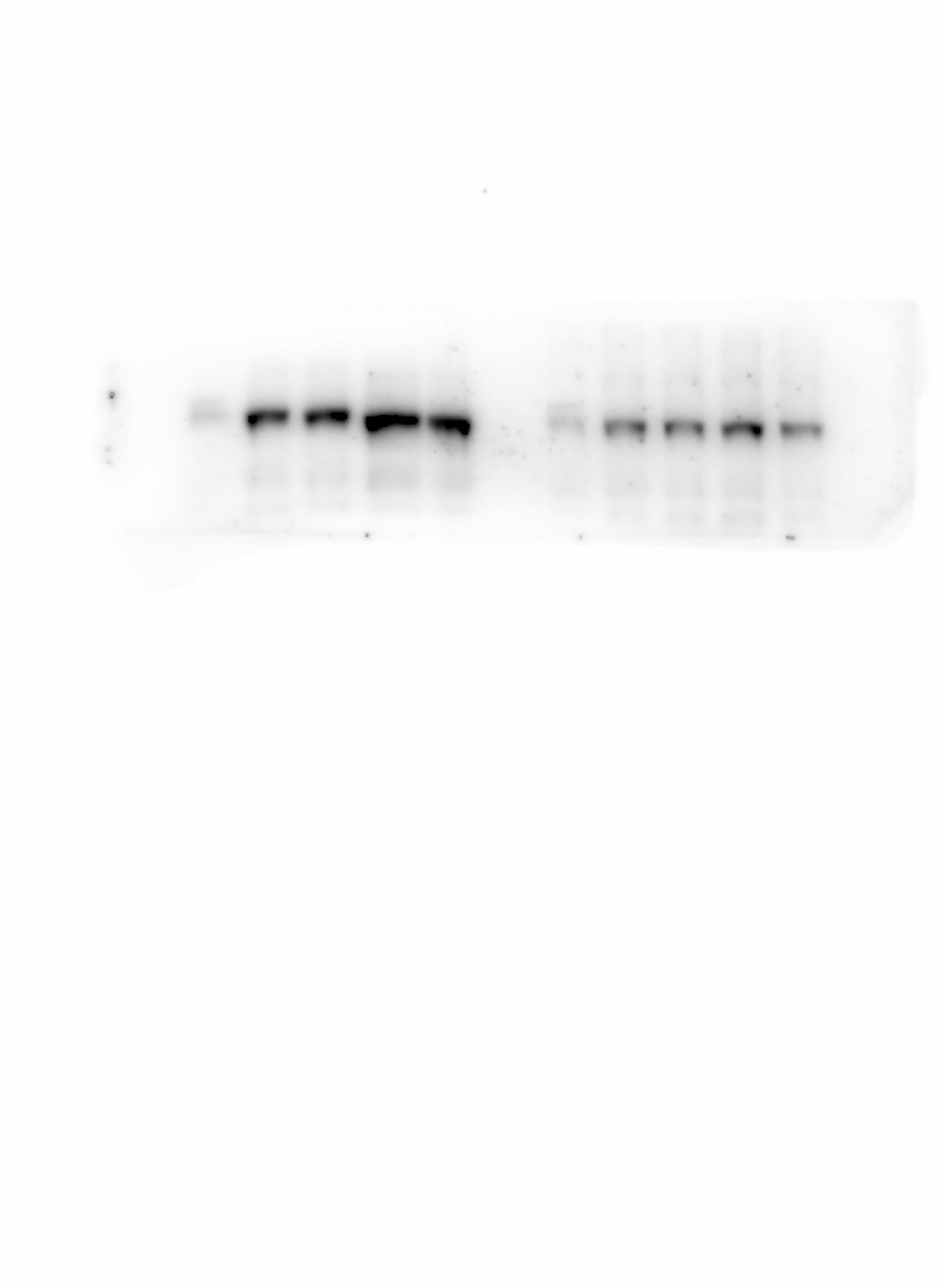

Supplement: Supplementary file 1 — Supplementary Material 1 [file 41598_2025_31281_MOESM1_ESM.zip › Fig3_R1/Fig.3B TRIF.tif]

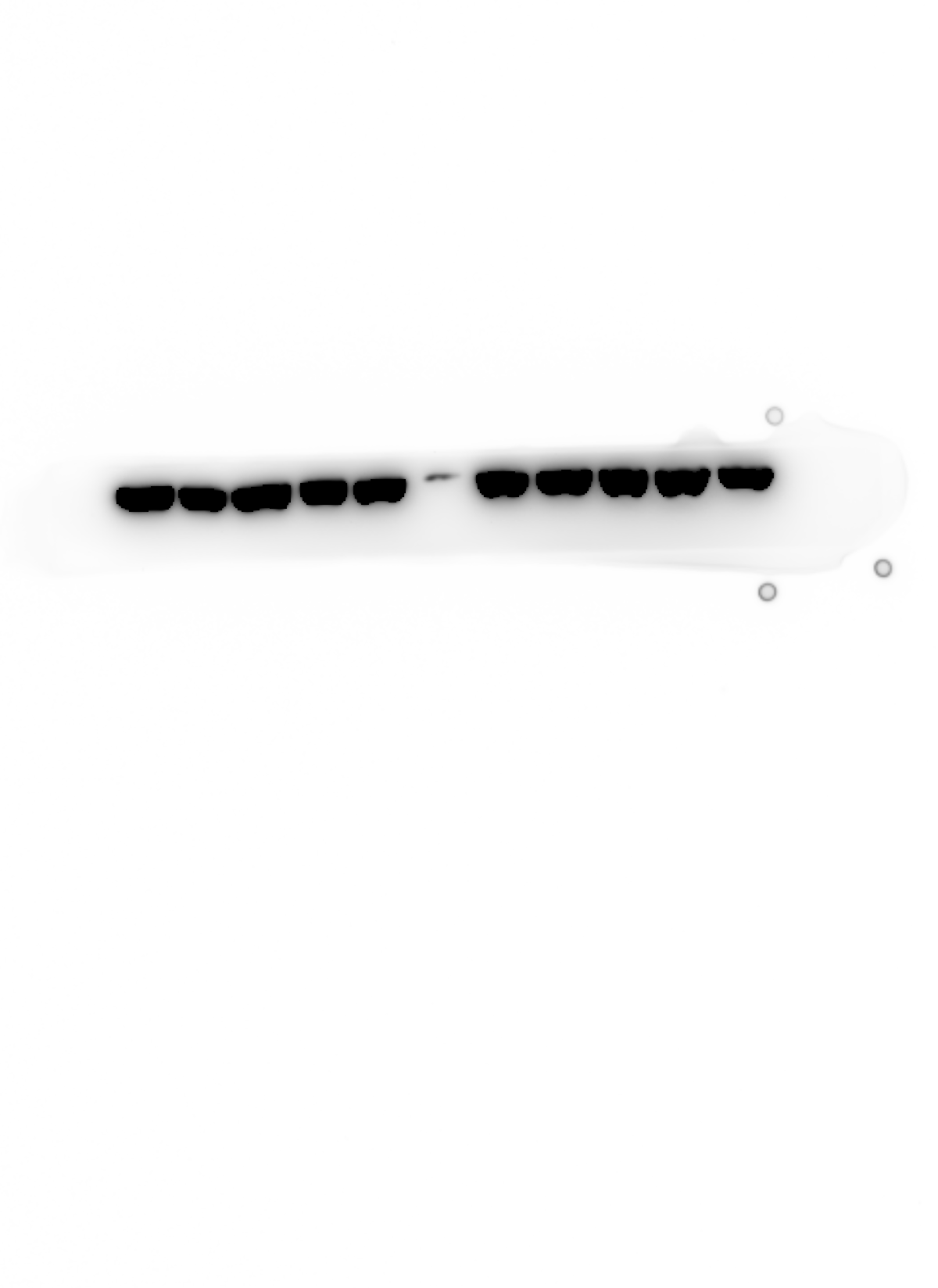

Supplement: Supplementary file 1 — Supplementary Material 1 [file 41598_2025_31281_MOESM1_ESM.zip › Fig3_R1/Fig.3E b-actin.tif]

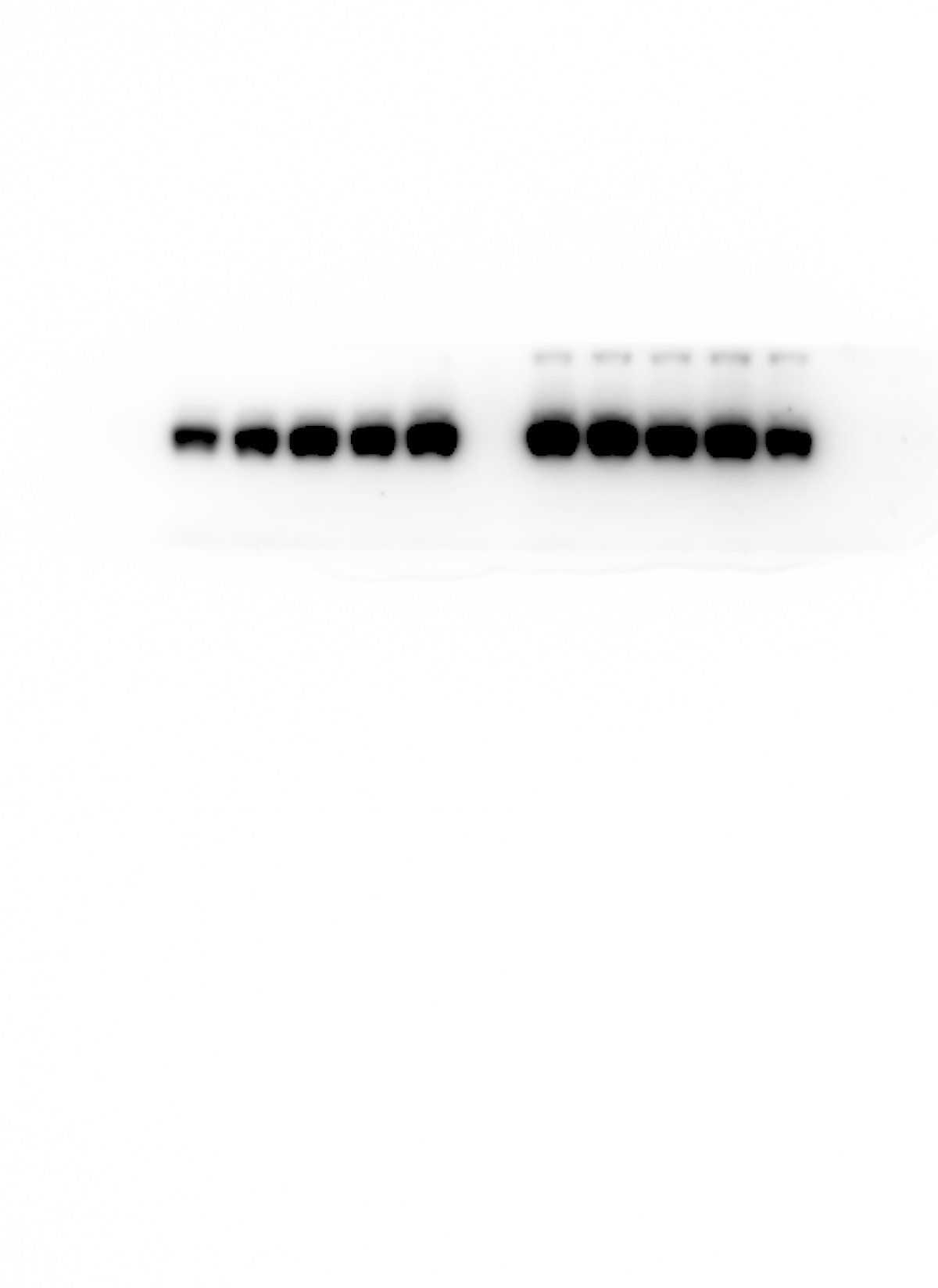

Supplement: Supplementary file 1 — Supplementary Material 1 [file 41598_2025_31281_MOESM1_ESM.zip › Fig3_R1/Fig.3E IRF3.tif]

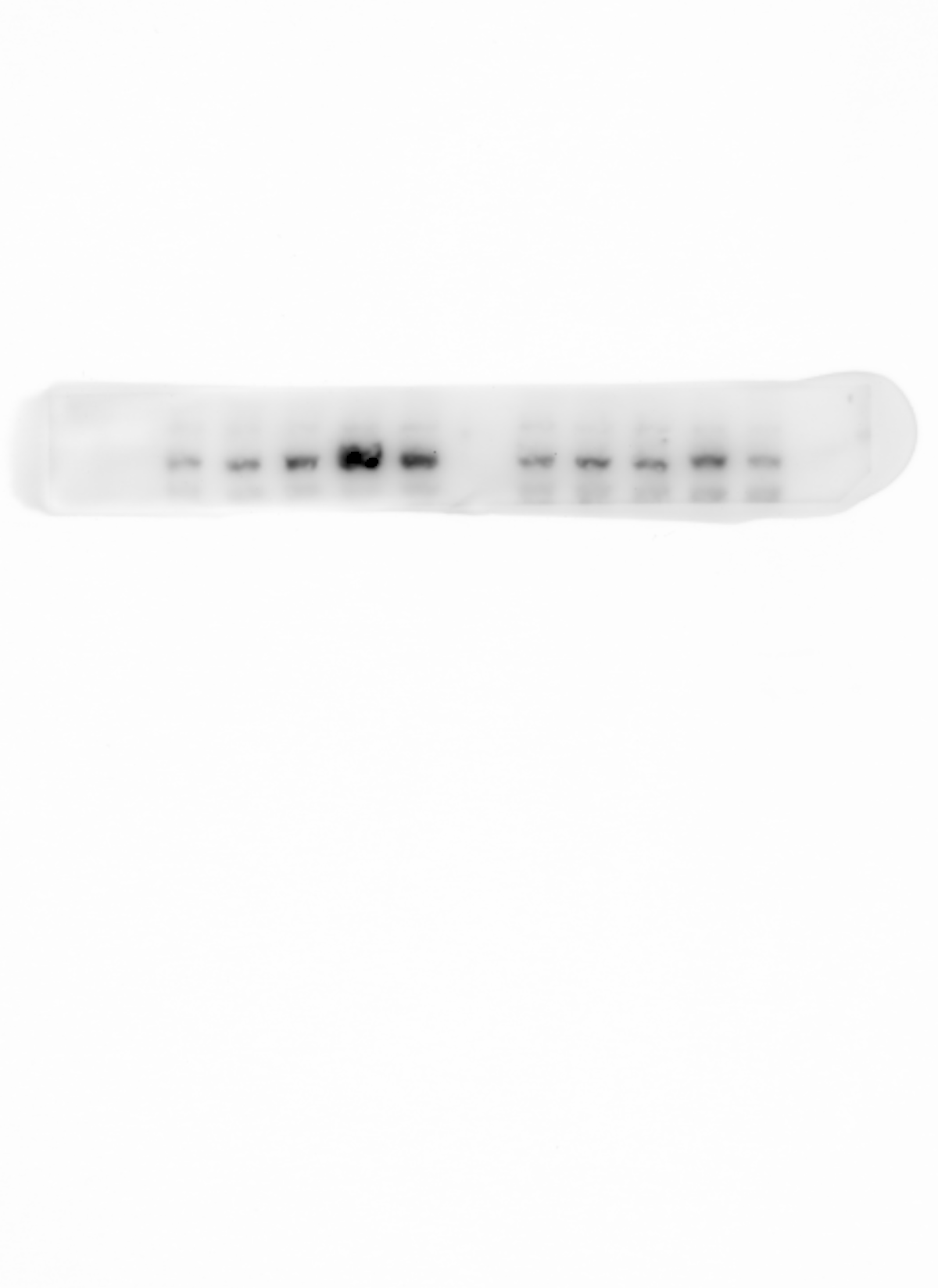

Supplement: Supplementary file 1 — Supplementary Material 1 [file 41598_2025_31281_MOESM1_ESM.zip › Fig3_R1/Fig.3E p-IRF3(S36).tif]

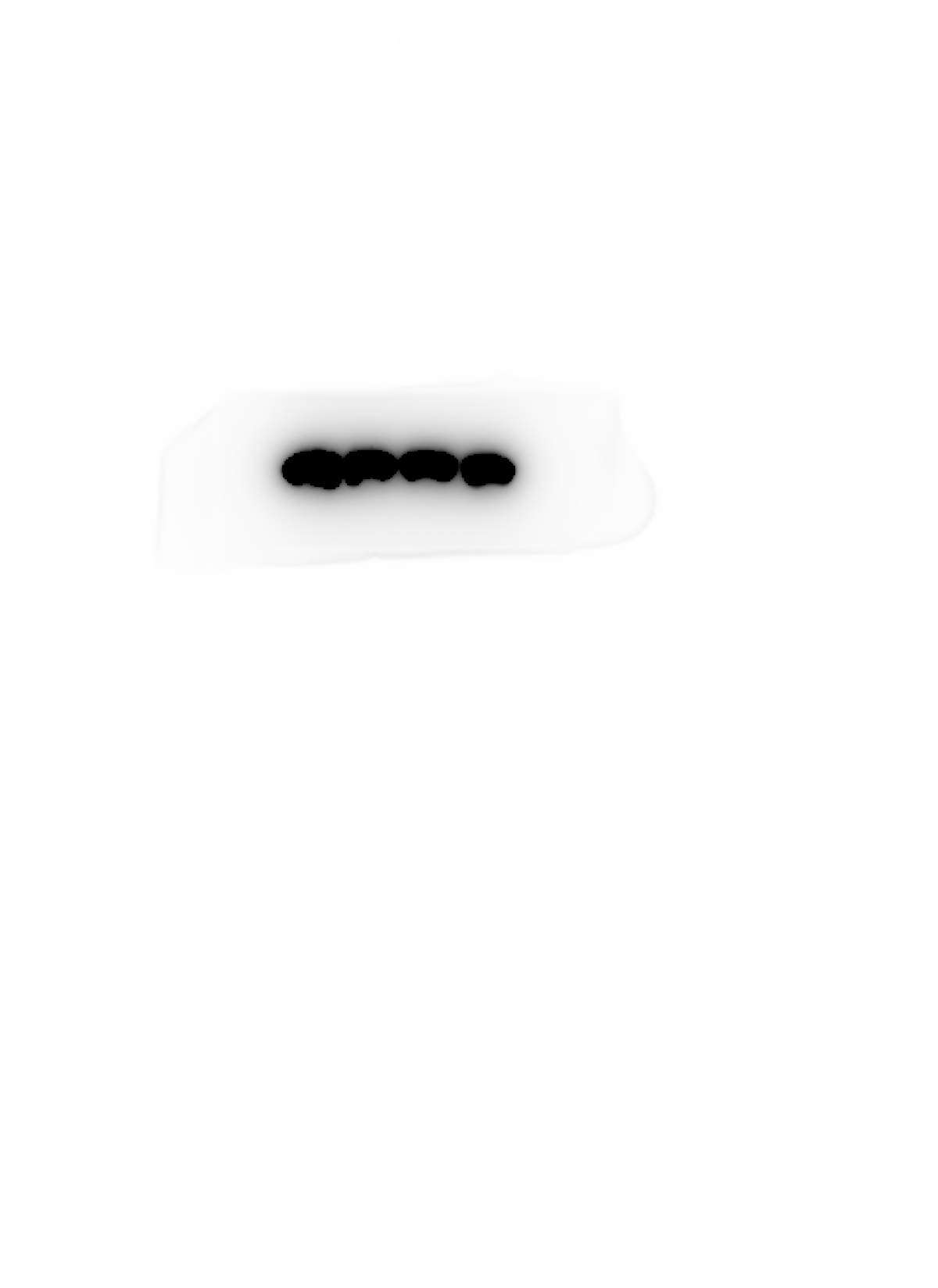

Supplement: Supplementary file 1 — Supplementary Material 1 [file 41598_2025_31281_MOESM1_ESM.zip › Fig3_R1/Fig.3G b-actin.tif]

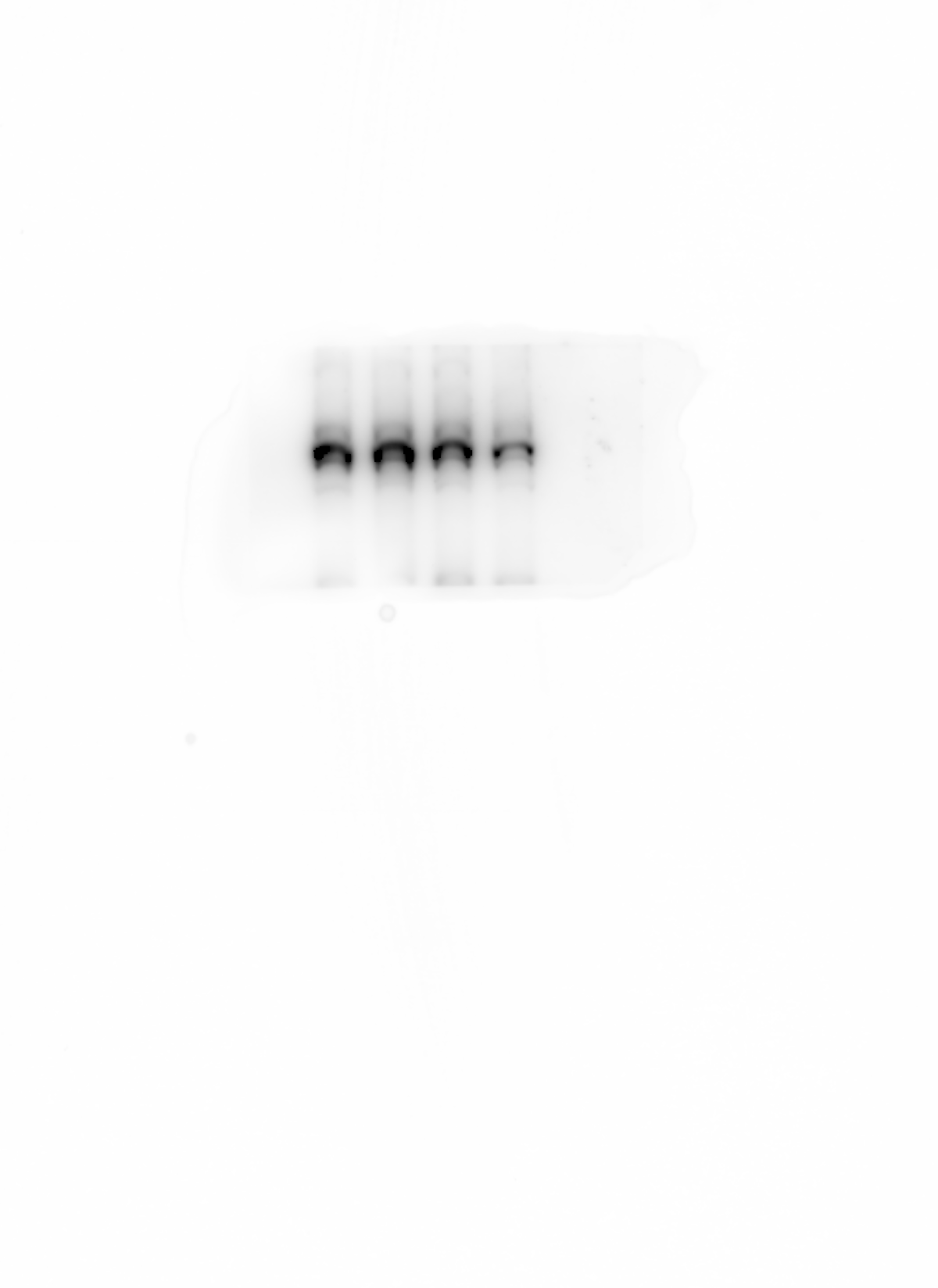

Supplement: Supplementary file 1 — Supplementary Material 1 [file 41598_2025_31281_MOESM1_ESM.zip › Fig3_R1/Fig.3G TLR3.tif]

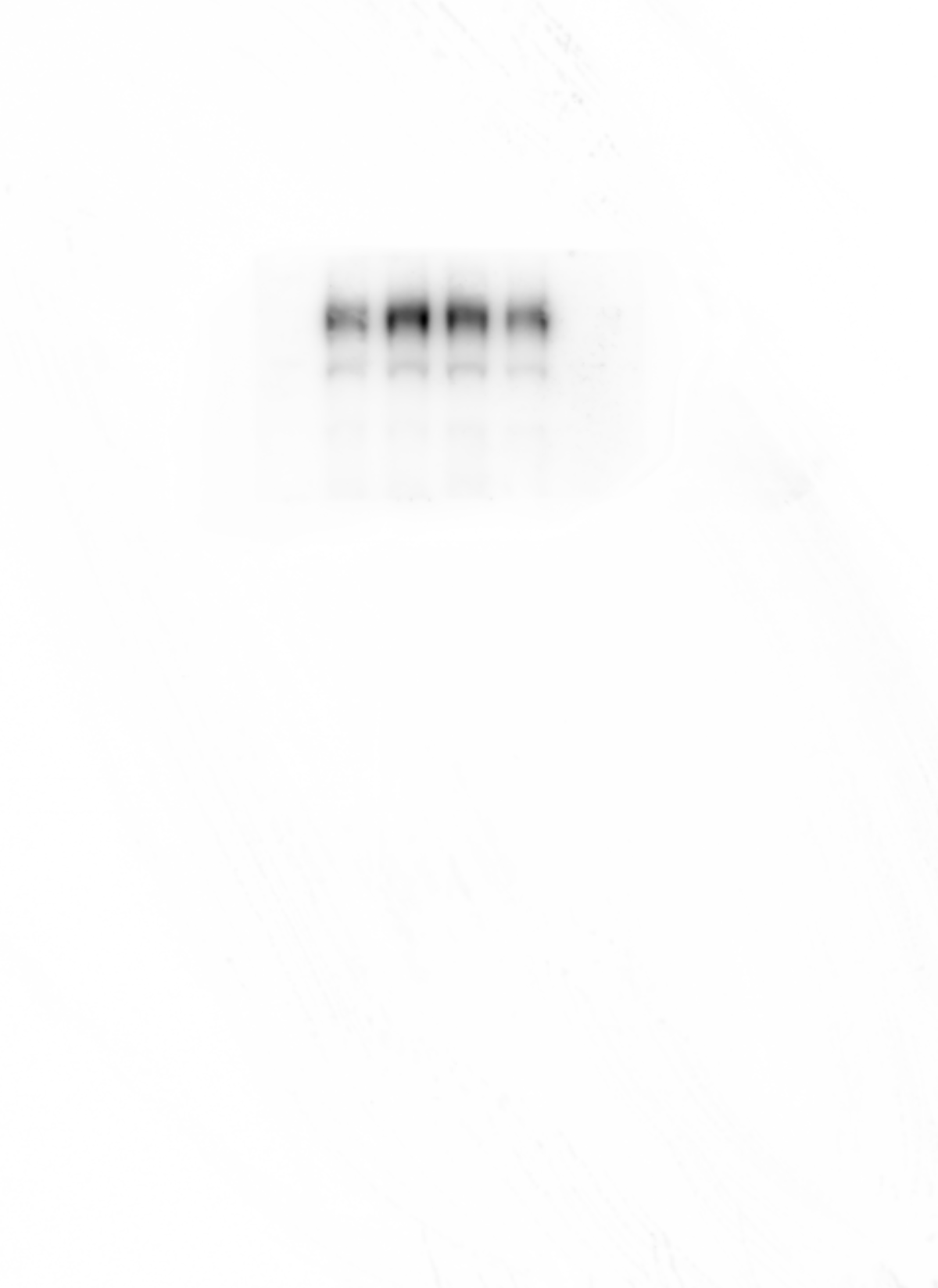

Supplement: Supplementary file 1 — Supplementary Material 1 [file 41598_2025_31281_MOESM1_ESM.zip › Fig3_R1/Fig.3G TLR4.tif]

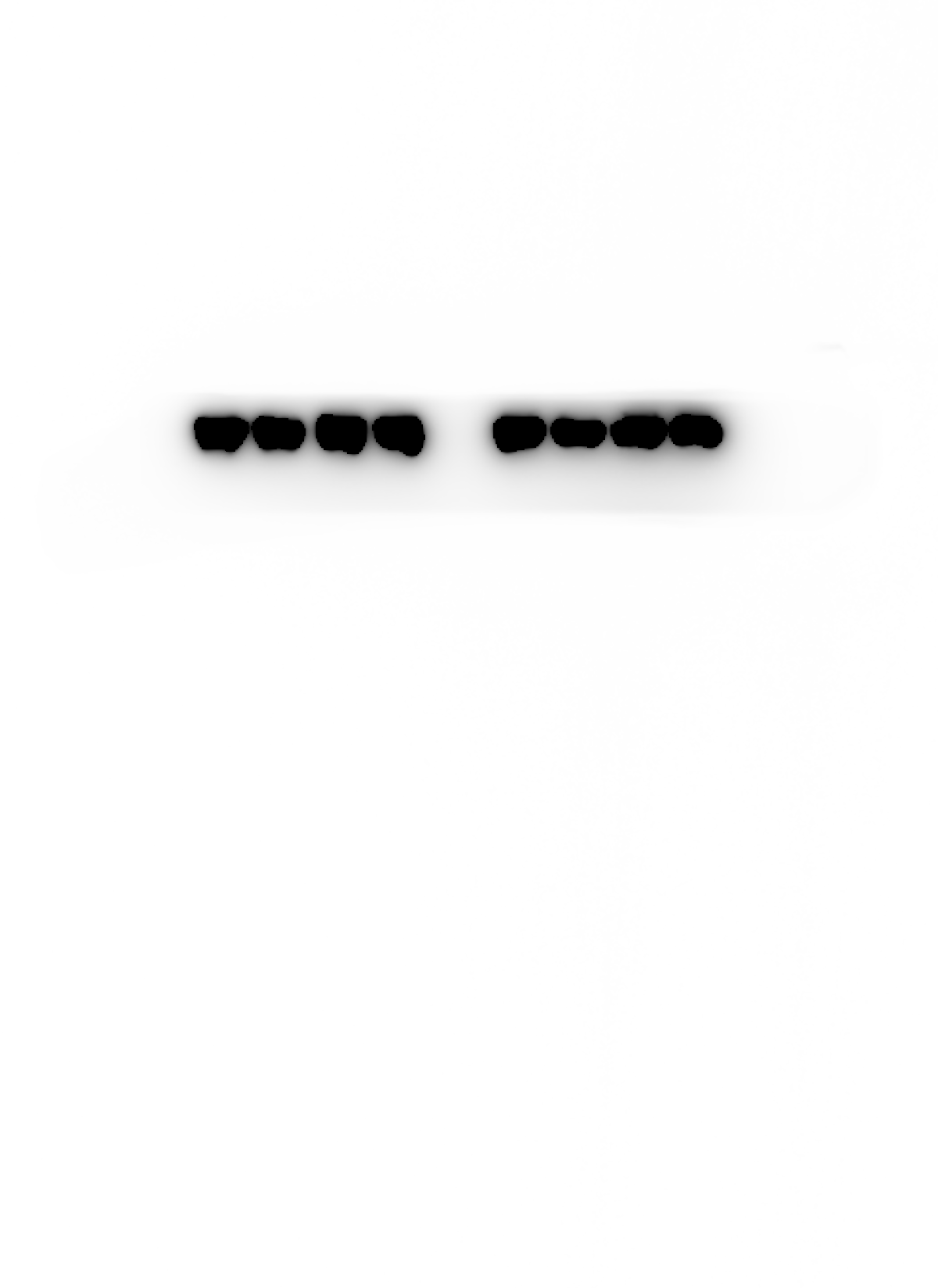

Supplement: Supplementary file 2 — Supplementary Material 2 [file 41598_2025_31281_MOESM2_ESM.zip › Fig4_R1/Fig.4E b-actin.tif]

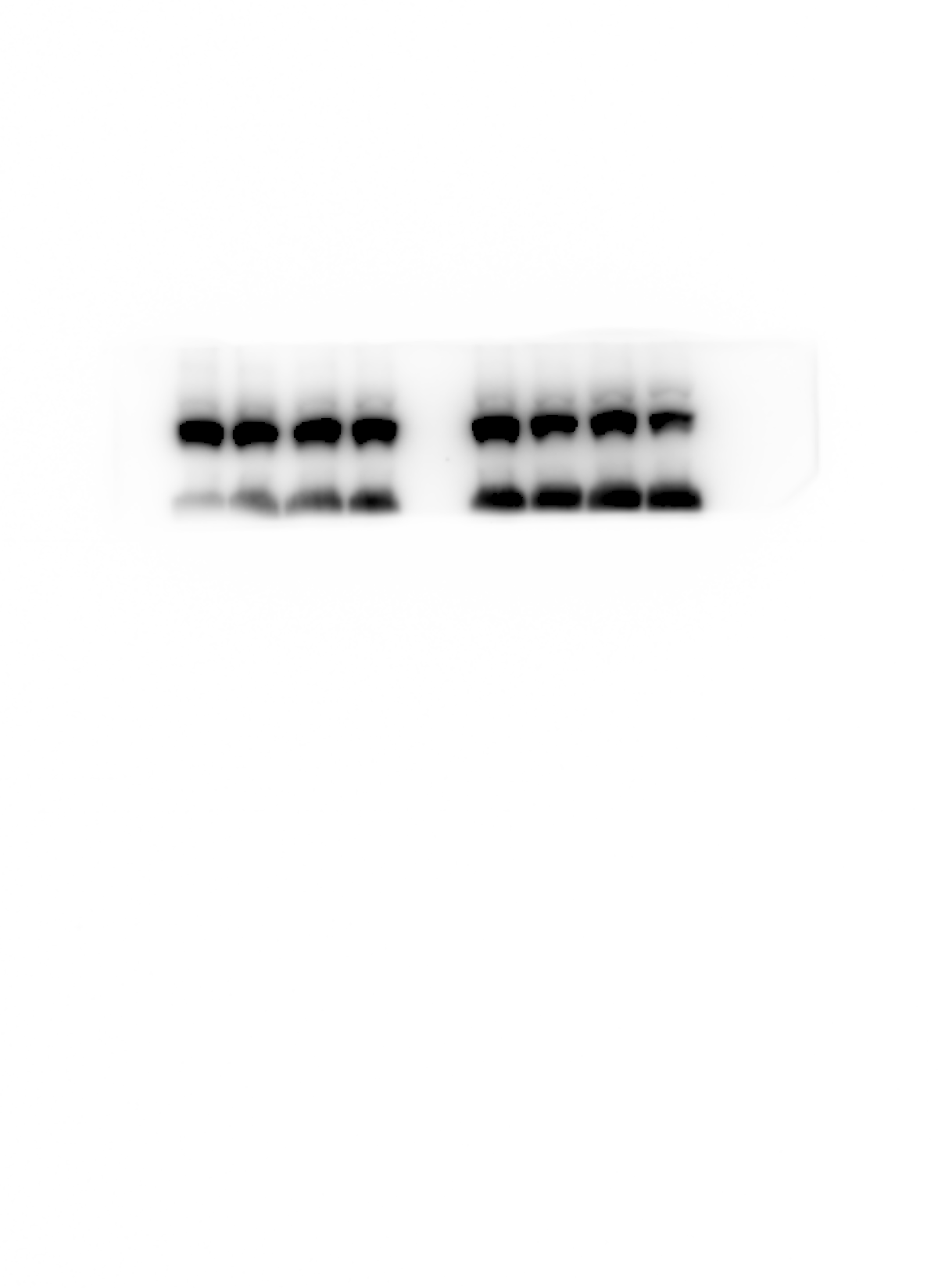

Supplement: Supplementary file 2 — Supplementary Material 2 [file 41598_2025_31281_MOESM2_ESM.zip › Fig4_R1/Fig.4E IRF3.tif]

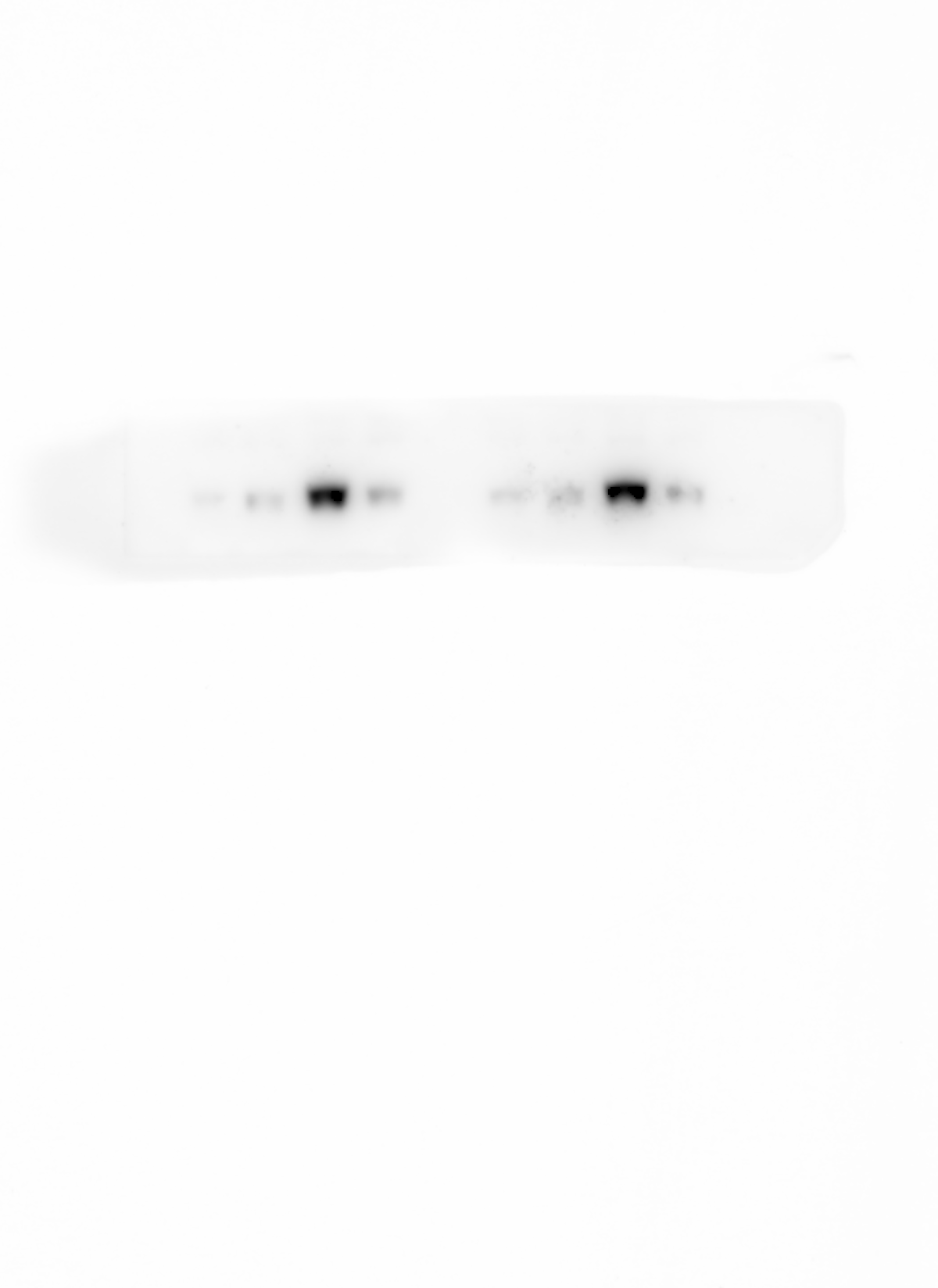

Supplement: Supplementary file 2 — Supplementary Material 2 [file 41598_2025_31281_MOESM2_ESM.zip › Fig4_R1/Fig.4E p-IRF3(S386).tif]

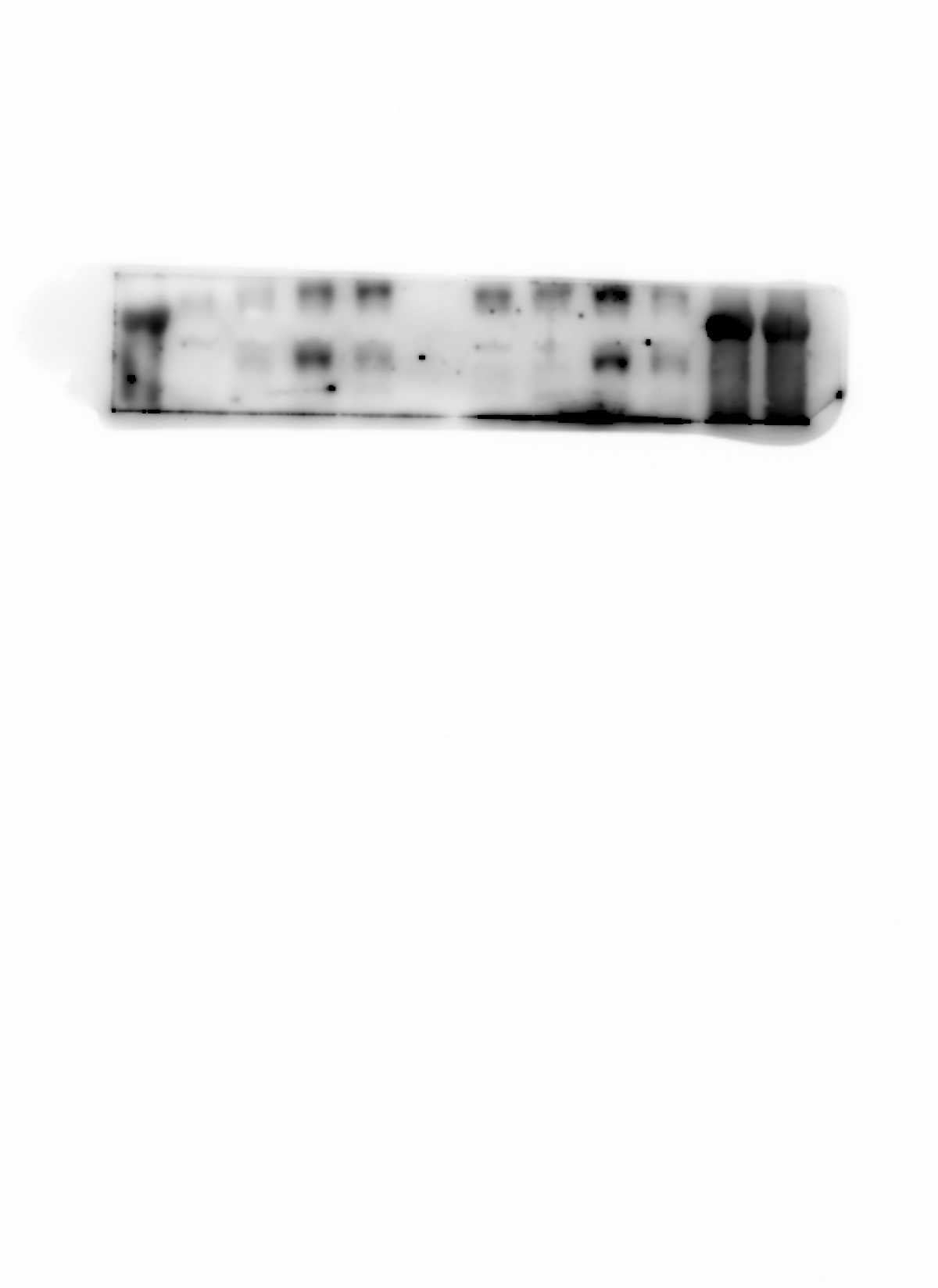

Supplement: Supplementary file 2 — Supplementary Material 2 [file 41598_2025_31281_MOESM2_ESM.zip › Fig4_R1/Fig.4E p-IRF3(S396).tif]

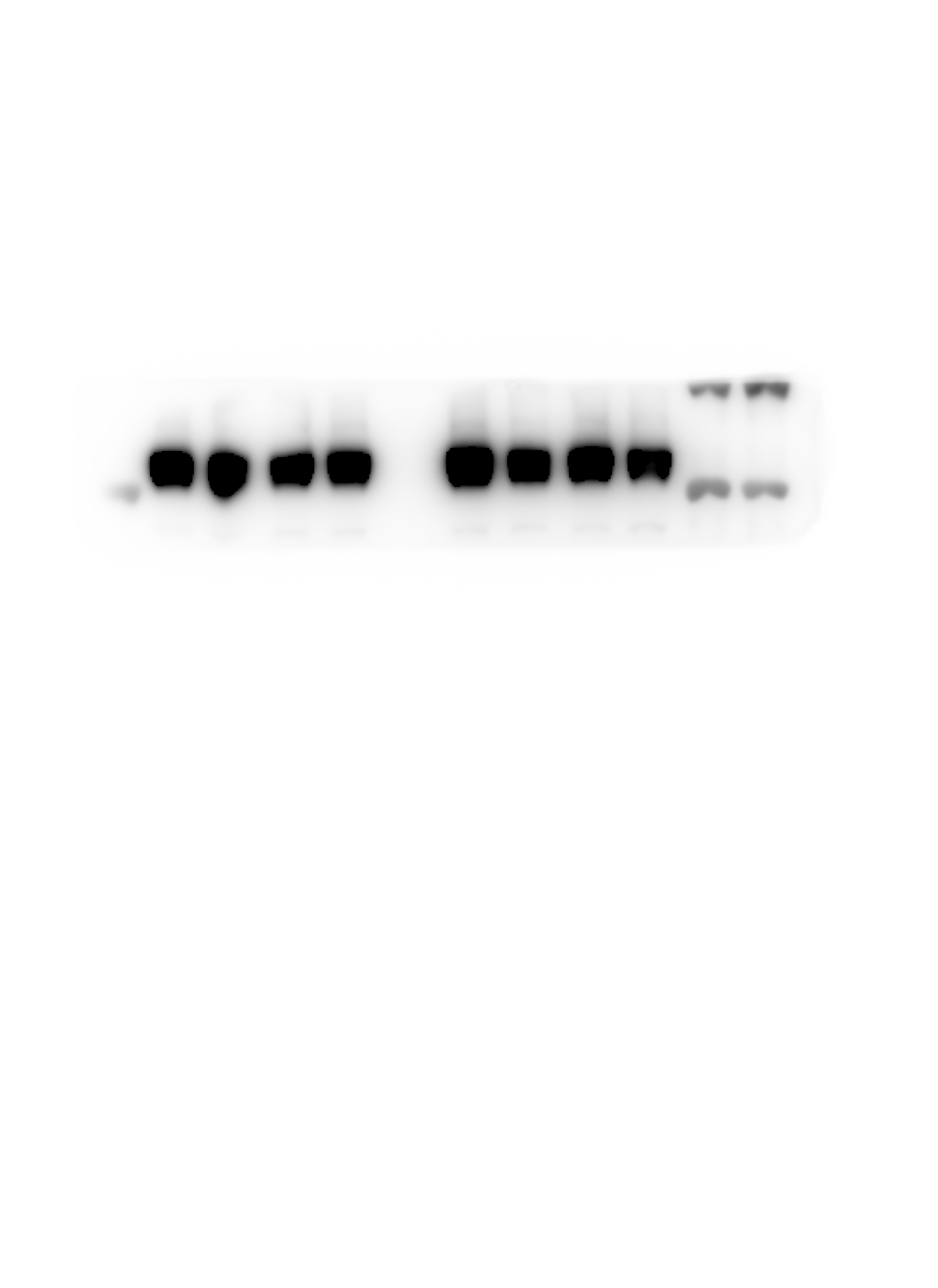

Supplement: Supplementary file 2 — Supplementary Material 2 [file 41598_2025_31281_MOESM2_ESM.zip › Fig4_R1/Fig.4H AKT.tif]

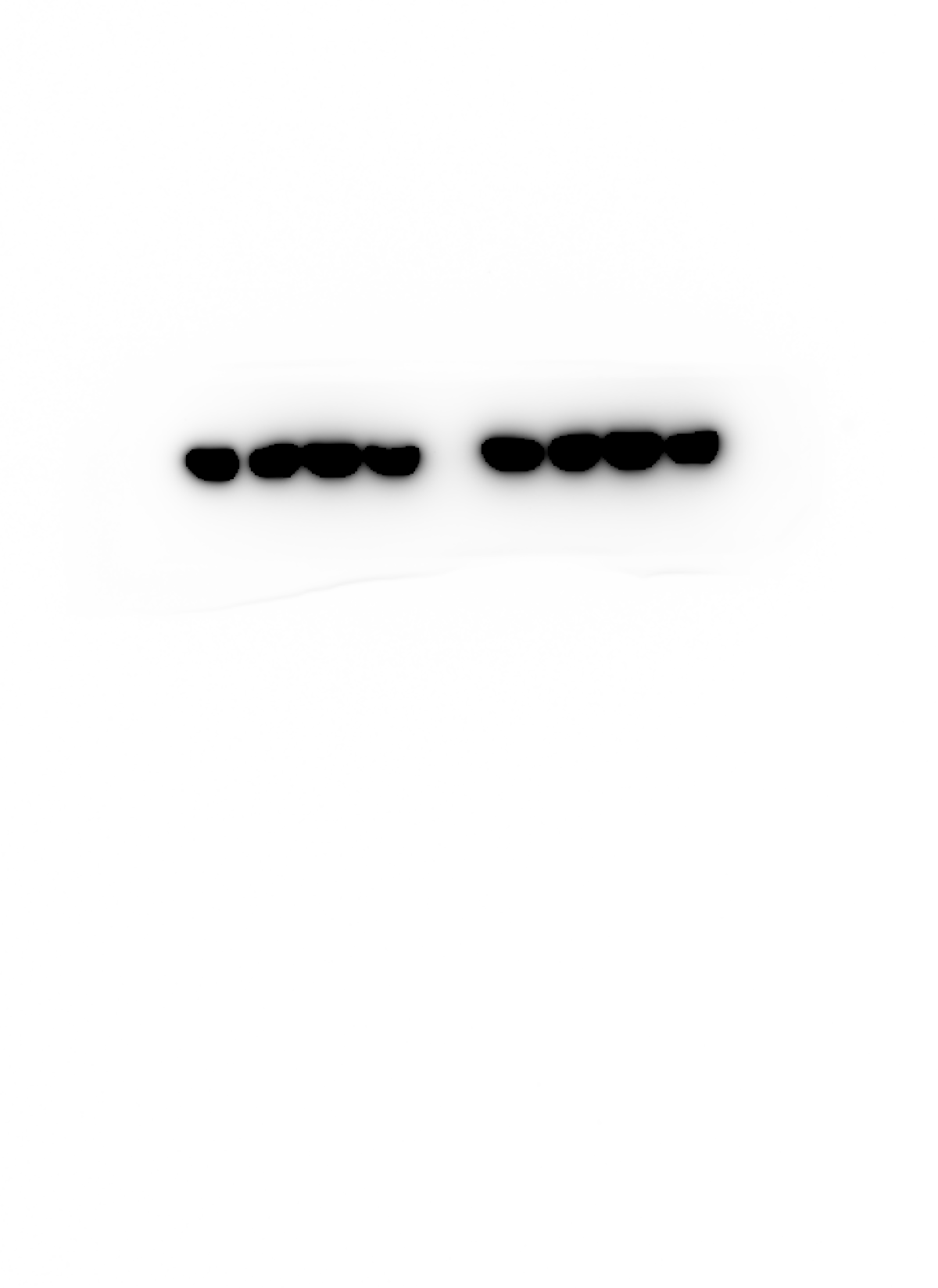

Supplement: Supplementary file 2 — Supplementary Material 2 [file 41598_2025_31281_MOESM2_ESM.zip › Fig4_R1/Fig.4H b-actin.tif]

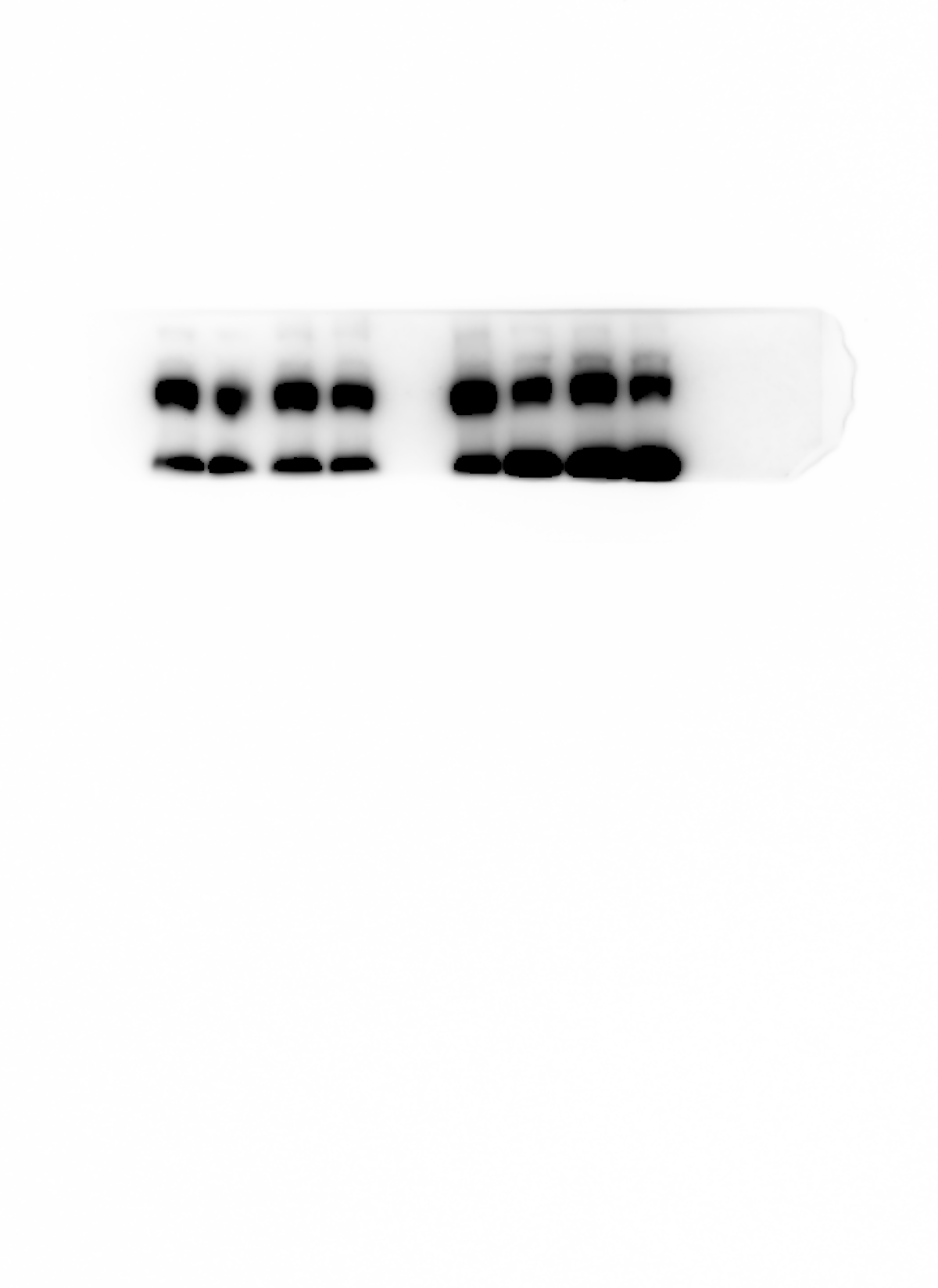

Supplement: Supplementary file 2 — Supplementary Material 2 [file 41598_2025_31281_MOESM2_ESM.zip › Fig4_R1/Fig.4H IRF3.tif]

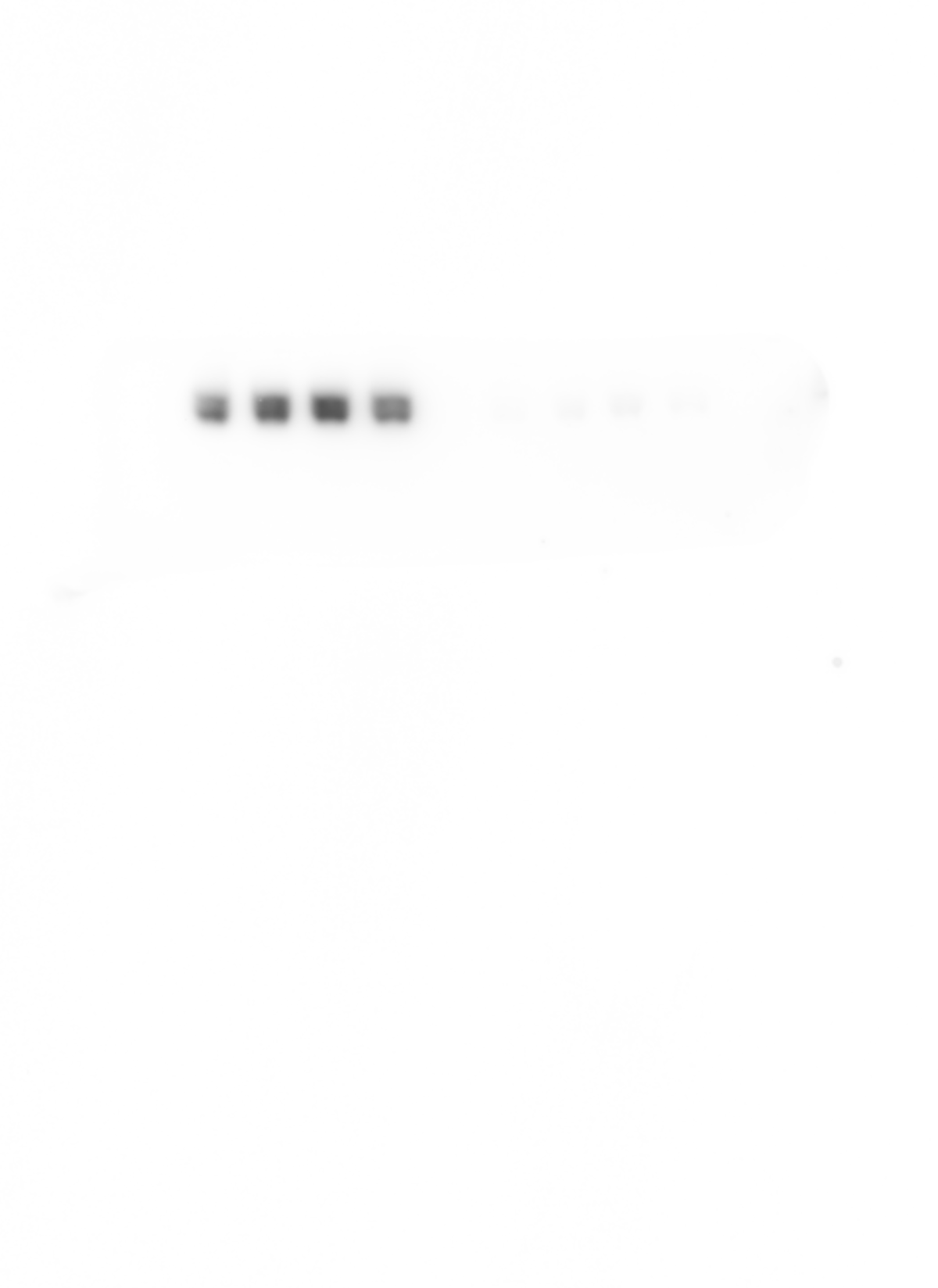

Supplement: Supplementary file 2 — Supplementary Material 2 [file 41598_2025_31281_MOESM2_ESM.zip › Fig4_R1/Fig.4H p-Akt(S473).tif]

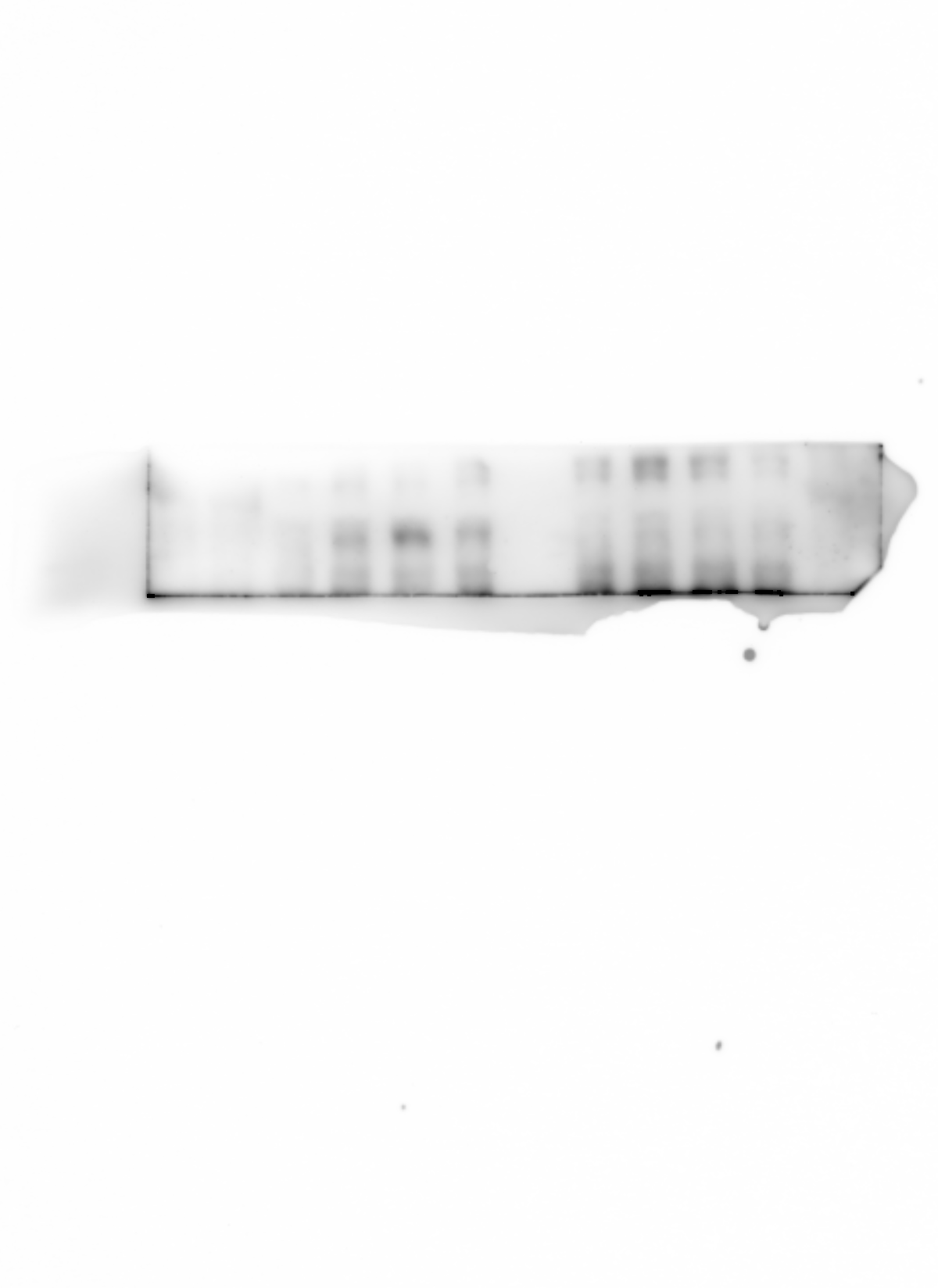

Supplement: Supplementary file 2 — Supplementary Material 2 [file 41598_2025_31281_MOESM2_ESM.zip › Fig4_R1/Fig.4H p-IRF3(S386).tif]

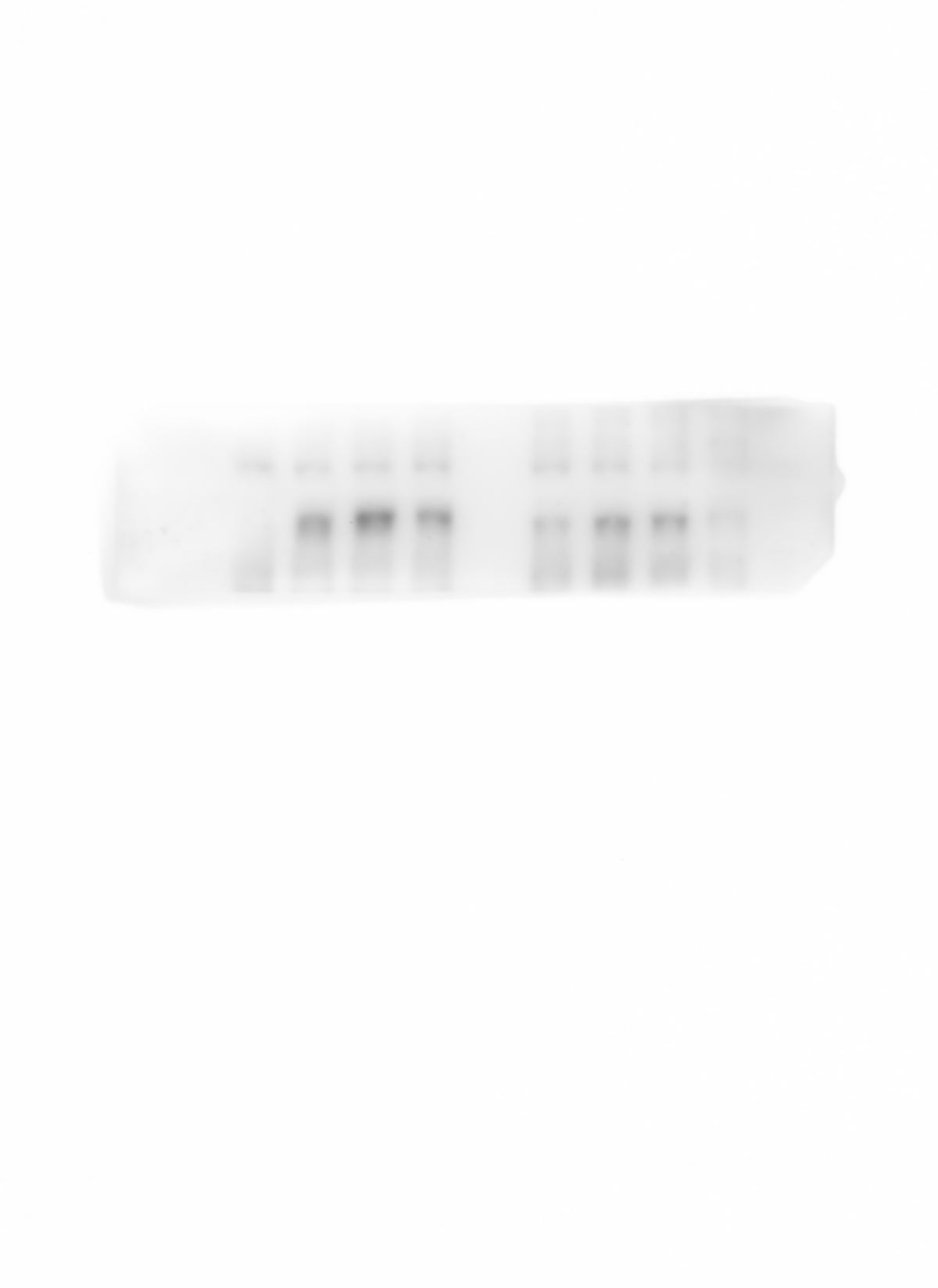

Supplement: Supplementary file 2 — Supplementary Material 2 [file 41598_2025_31281_MOESM2_ESM.zip › Fig4_R1/Fig.4H p-IRF3(S396).tif]

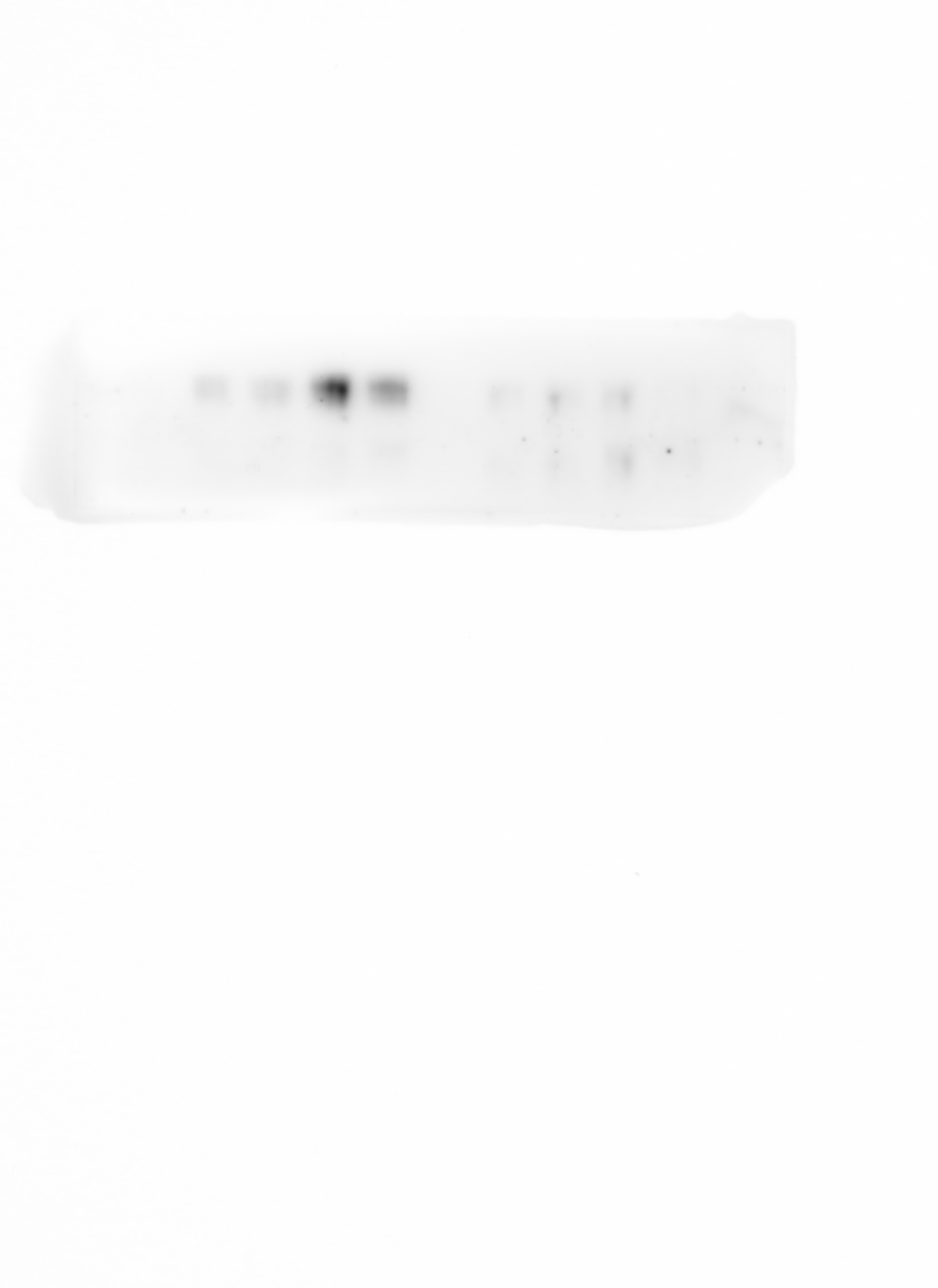

Supplement: Supplementary file 2 — Supplementary Material 2 [file 41598_2025_31281_MOESM2_ESM.zip › Fig4_R1/Fig.4H p-SGK1.tif]

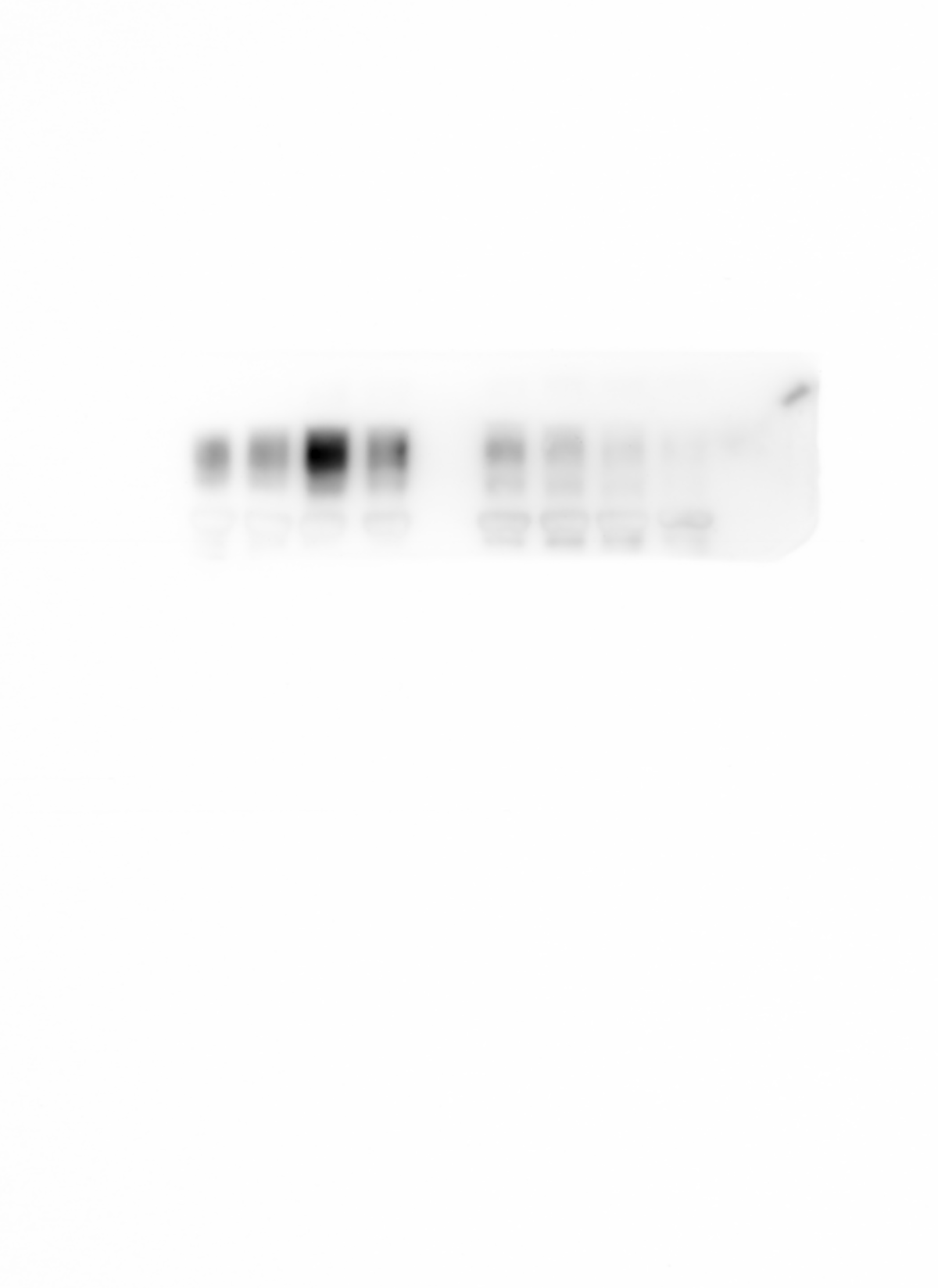

Supplement: Supplementary file 2 — Supplementary Material 2 [file 41598_2025_31281_MOESM2_ESM.zip › Fig4_R1/Fig.4H SGK1.tif]

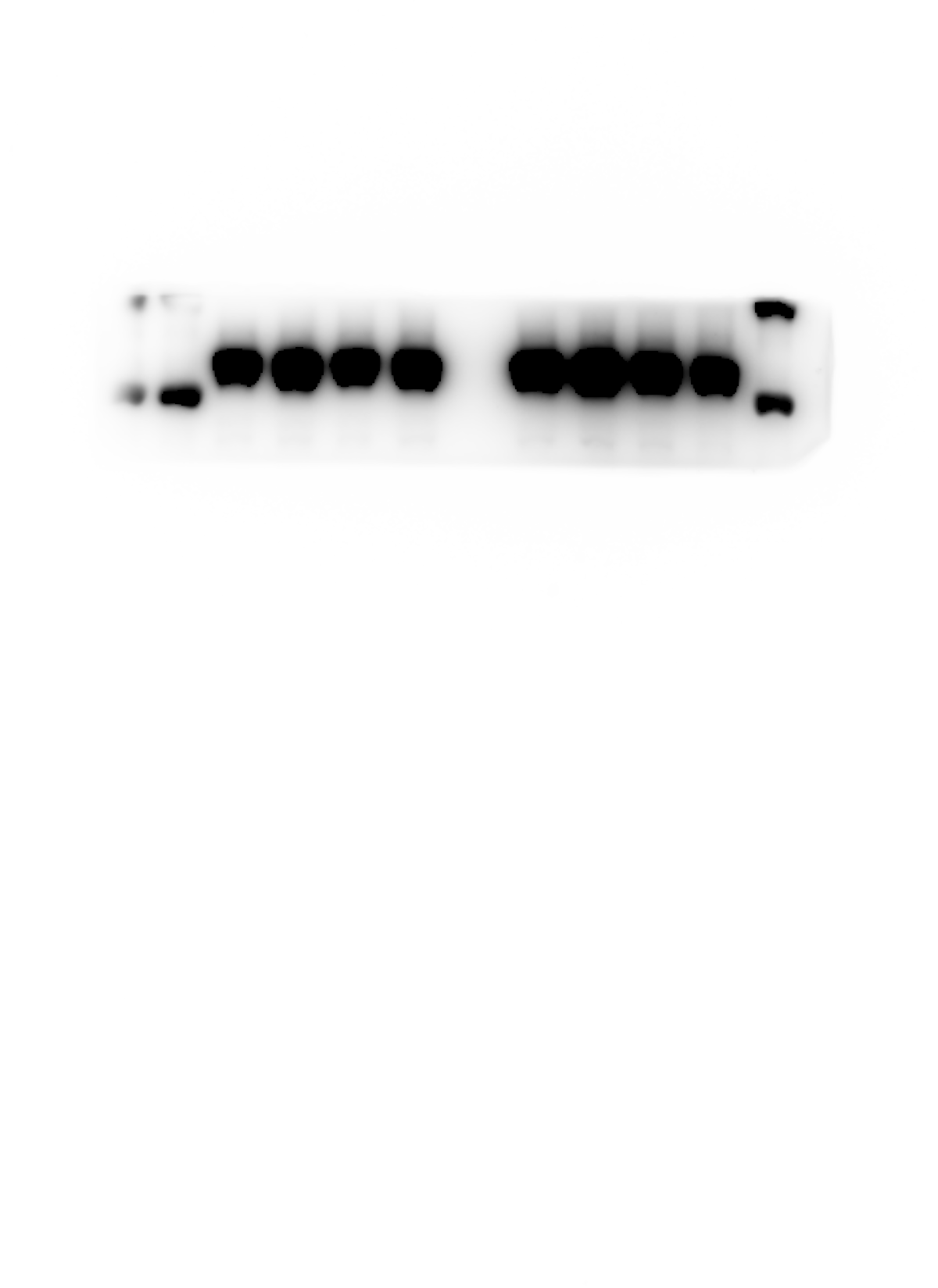

Supplement: Supplementary file 2 — Supplementary Material 2 [file 41598_2025_31281_MOESM2_ESM.zip › Fig5_R1/Fig.5B AKT.tif]

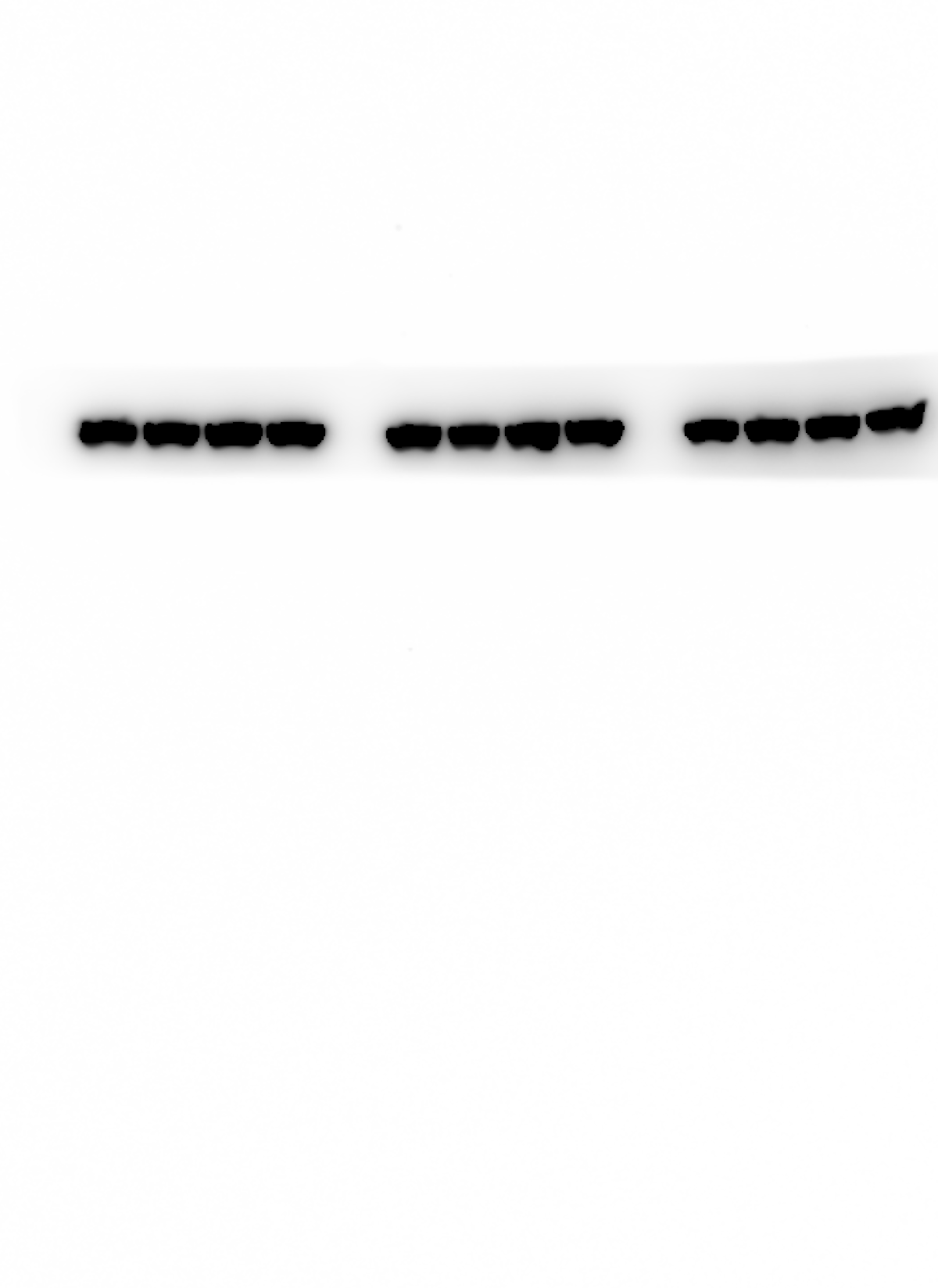

Supplement: Supplementary file 2 — Supplementary Material 2 [file 41598_2025_31281_MOESM2_ESM.zip › Fig5_R1/Fig.5B b-actin.tif]

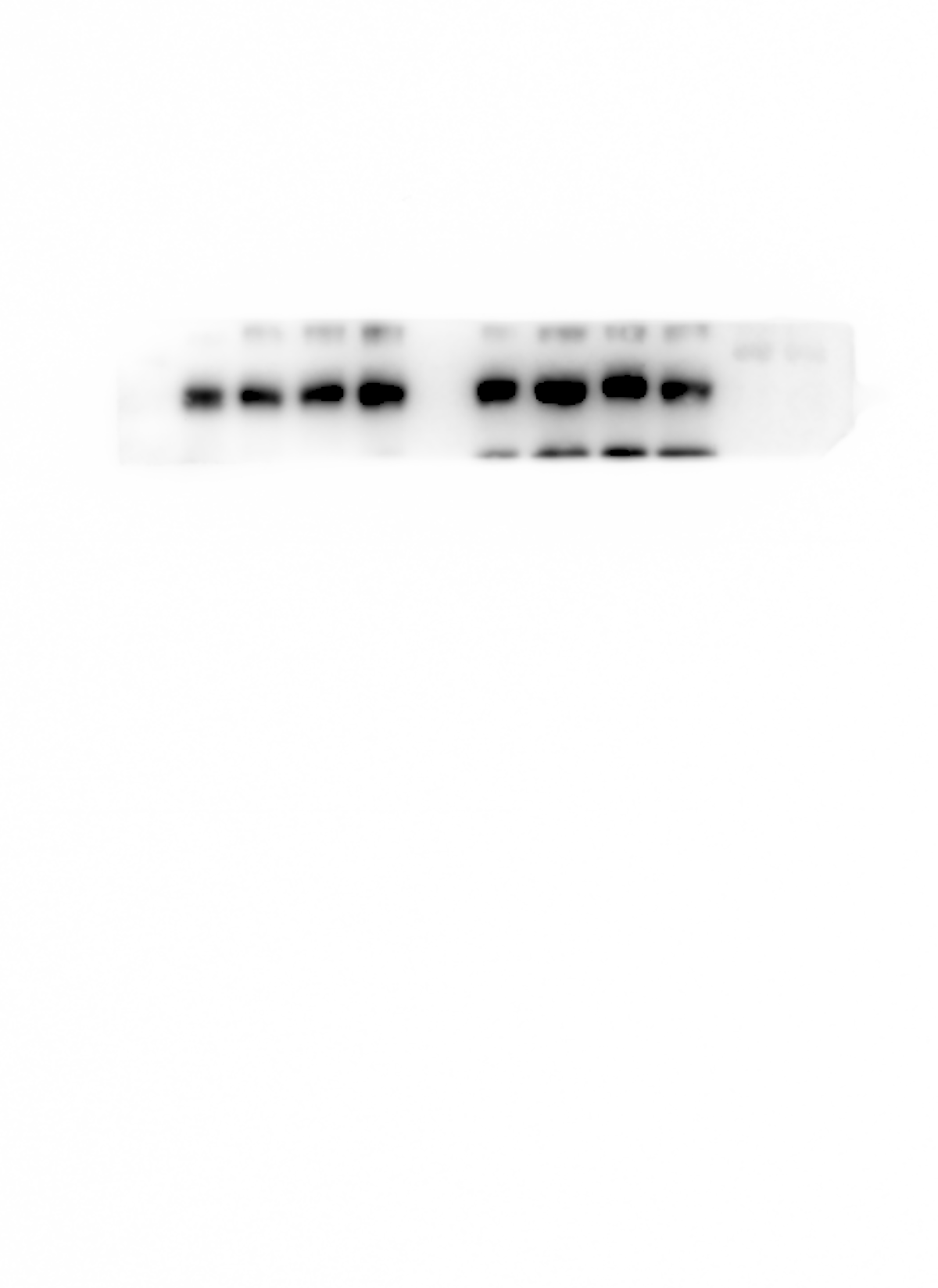

Supplement: Supplementary file 2 — Supplementary Material 2 [file 41598_2025_31281_MOESM2_ESM.zip › Fig5_R1/Fig.5B IRF3.tif]

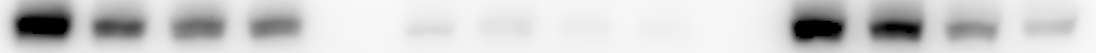

Supplement: Supplementary file 2 — Supplementary Material 2 [file 41598_2025_31281_MOESM2_ESM.zip › Fig5_R1/Fig.5B p-AKT(S473).tif]

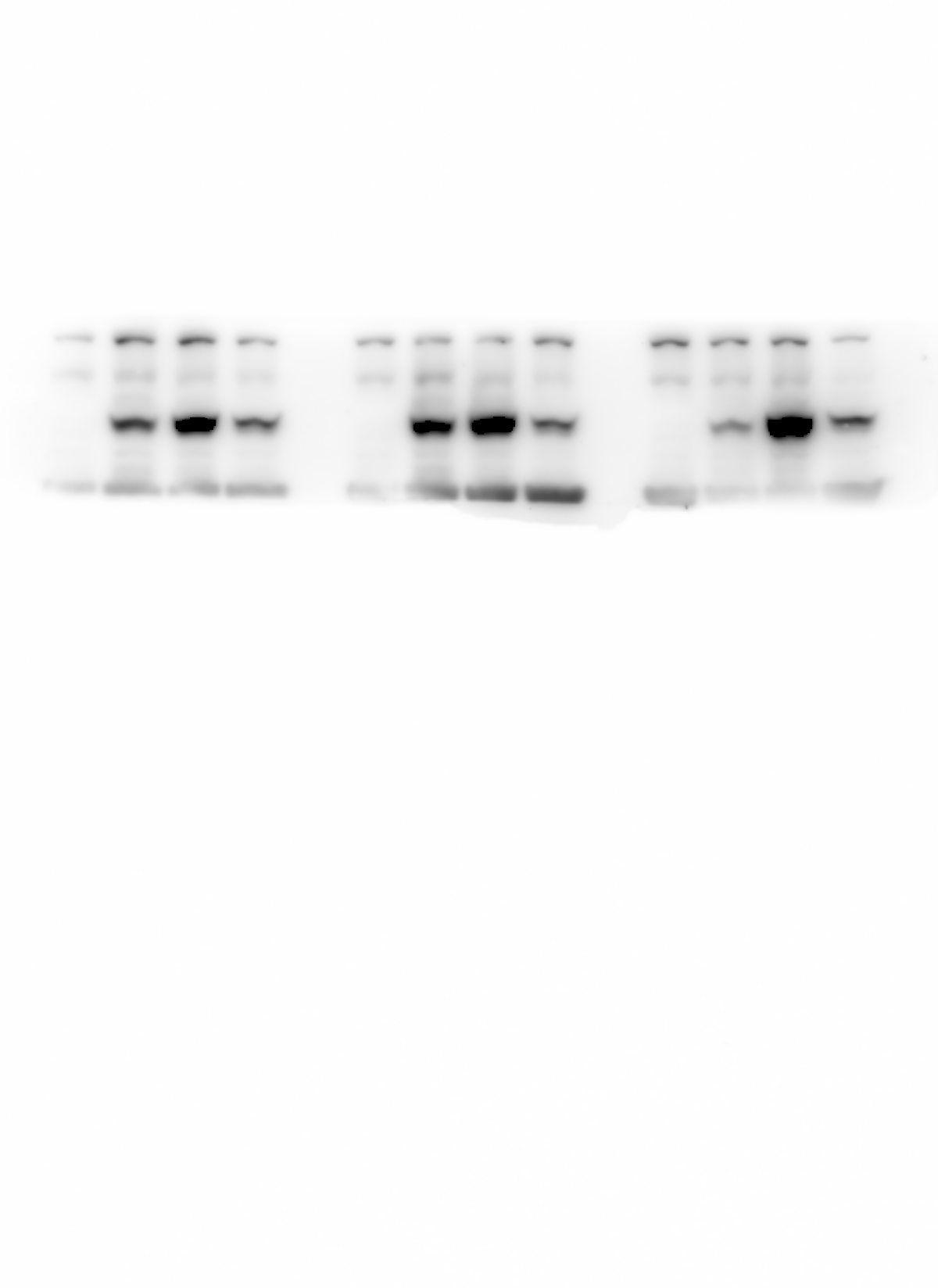

Supplement: Supplementary file 2 — Supplementary Material 2 [file 41598_2025_31281_MOESM2_ESM.zip › Fig5_R1/Fig.5B p-IRF3(S396).tif]

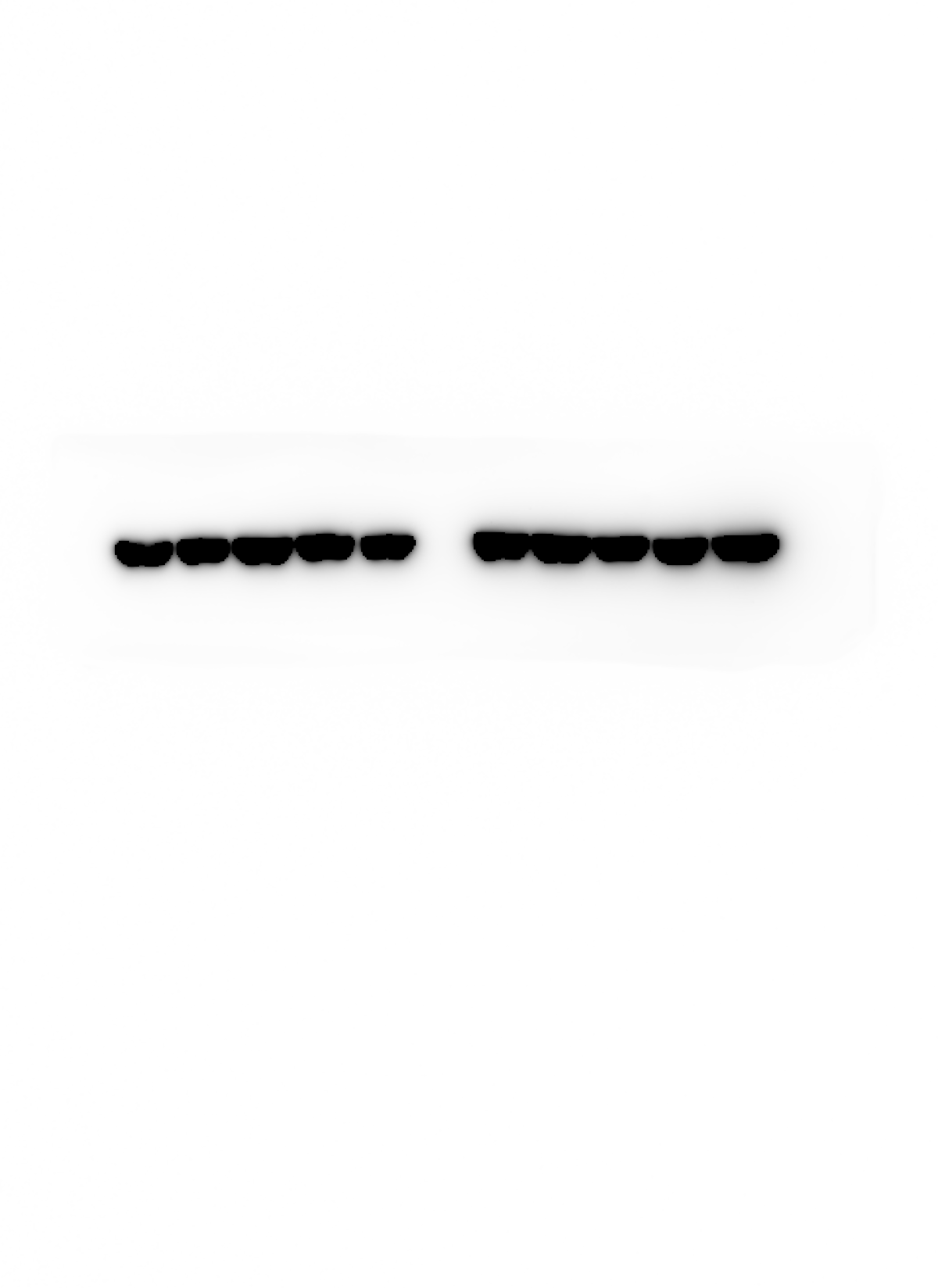

Supplement: Supplementary file 2 — Supplementary Material 2 [file 41598_2025_31281_MOESM2_ESM.zip › Fig5_R1/Fig.5D b-actin.tif]

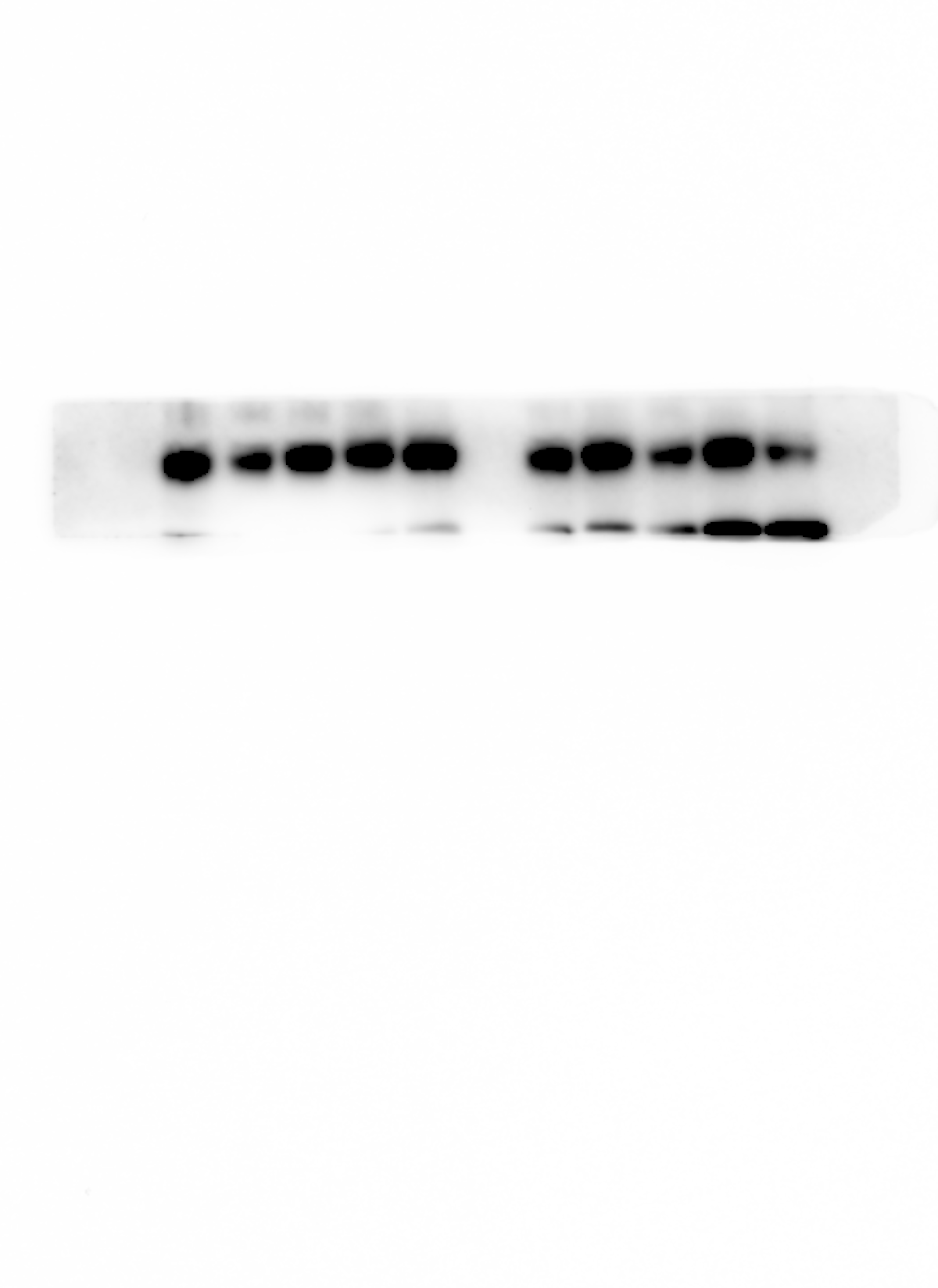

Supplement: Supplementary file 2 — Supplementary Material 2 [file 41598_2025_31281_MOESM2_ESM.zip › Fig5_R1/Fig.5D IRF3.tif]

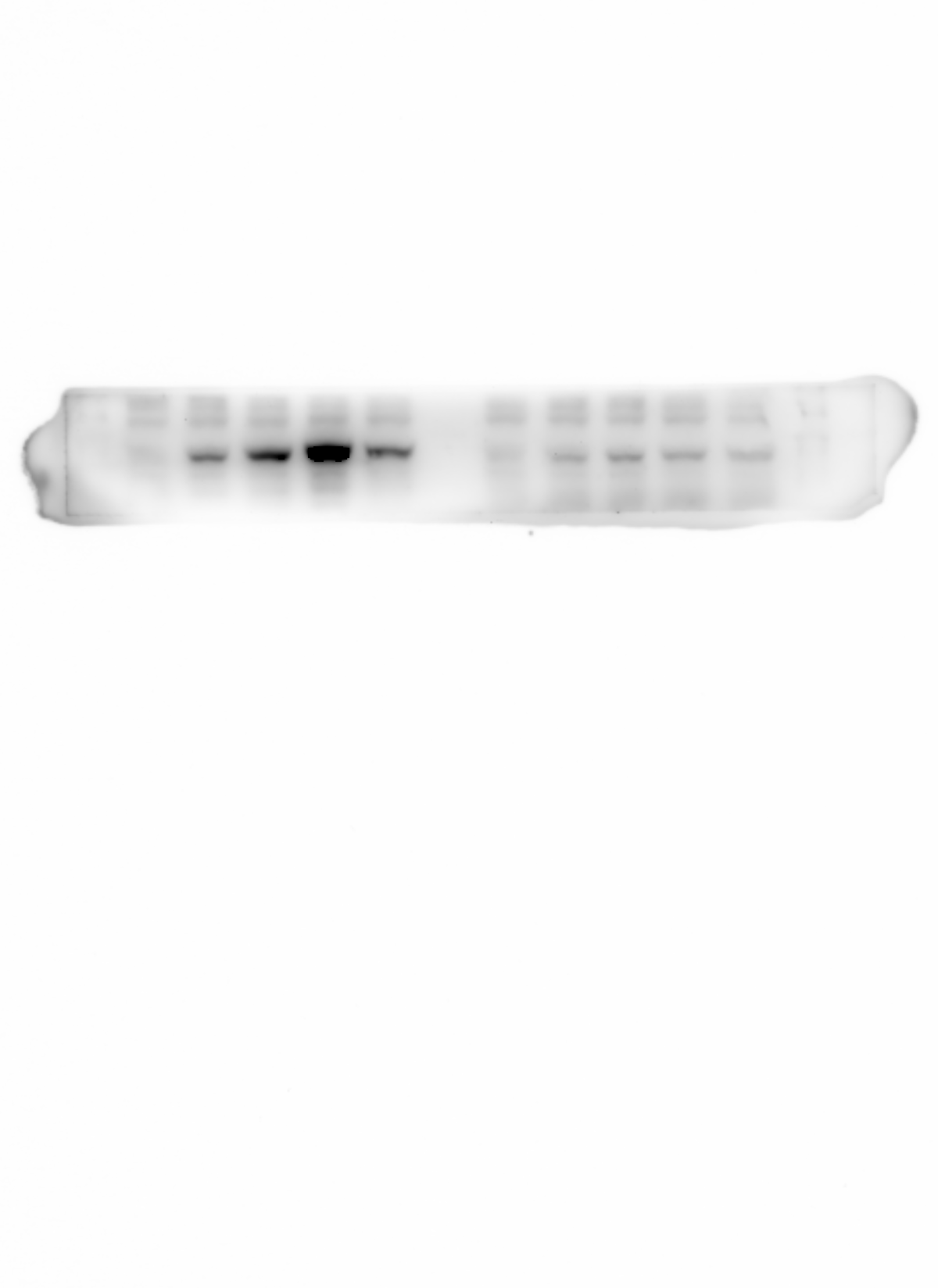

Supplement: Supplementary file 2 — Supplementary Material 2 [file 41598_2025_31281_MOESM2_ESM.zip › Fig5_R1/Fig.5D p-IRF3(S396).tif]

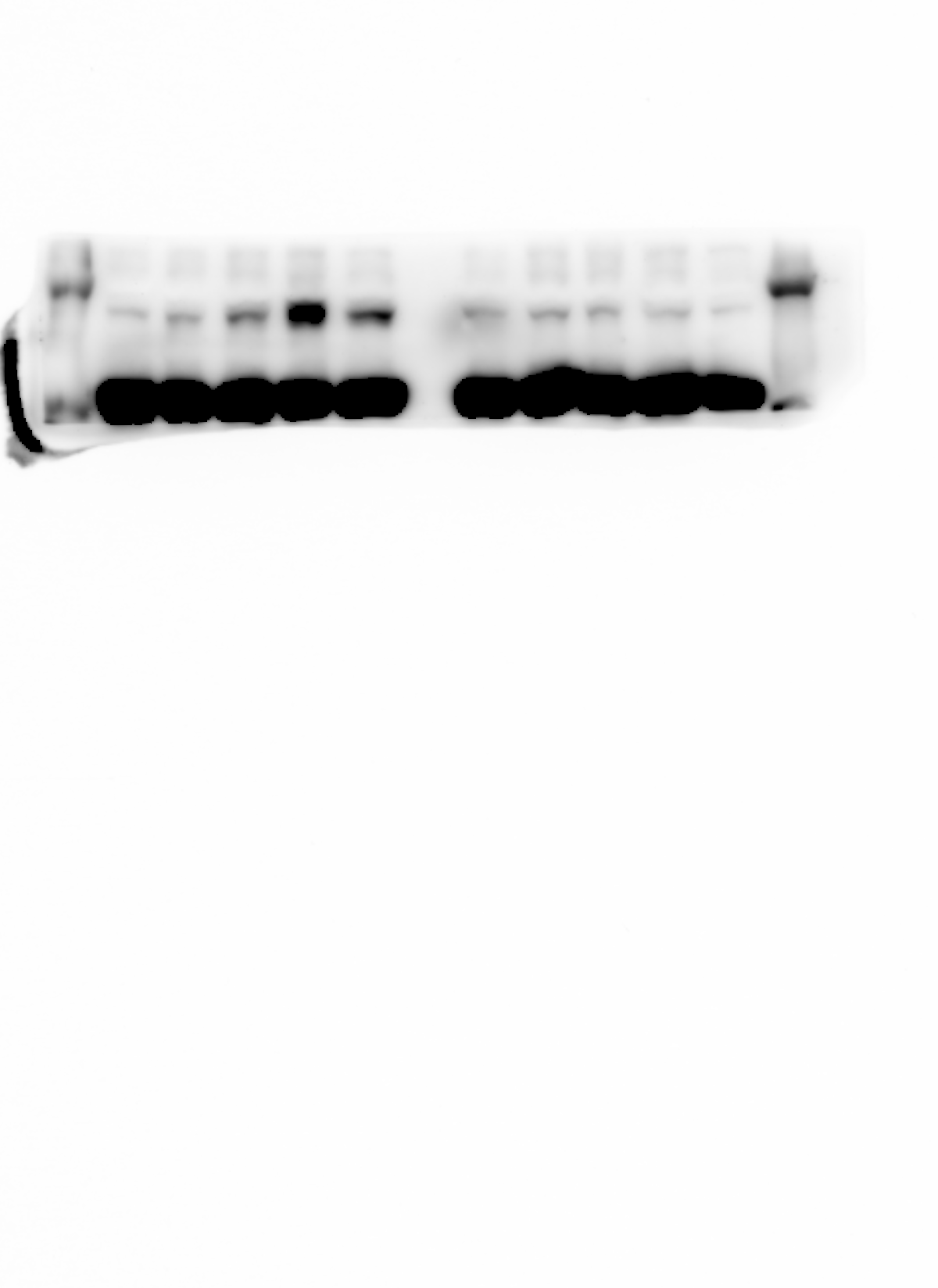

Supplement: Supplementary file 2 — Supplementary Material 2 [file 41598_2025_31281_MOESM2_ESM.zip › Fig5_R1/Fig.5D p-SGK1.tif]

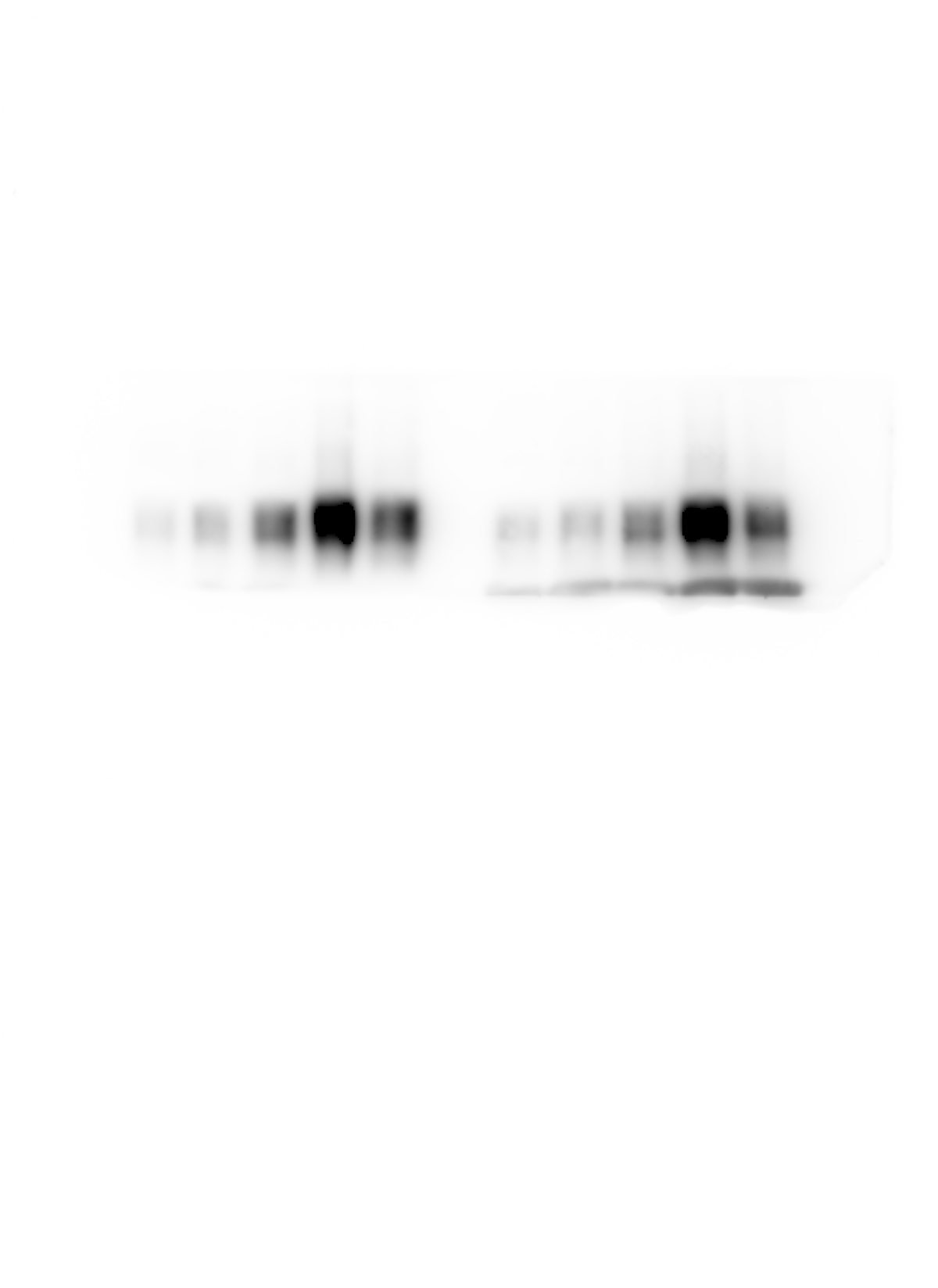

Supplement: Supplementary file 2 — Supplementary Material 2 [file 41598_2025_31281_MOESM2_ESM.zip › Fig5_R1/Fig.5D SGK1.tif]

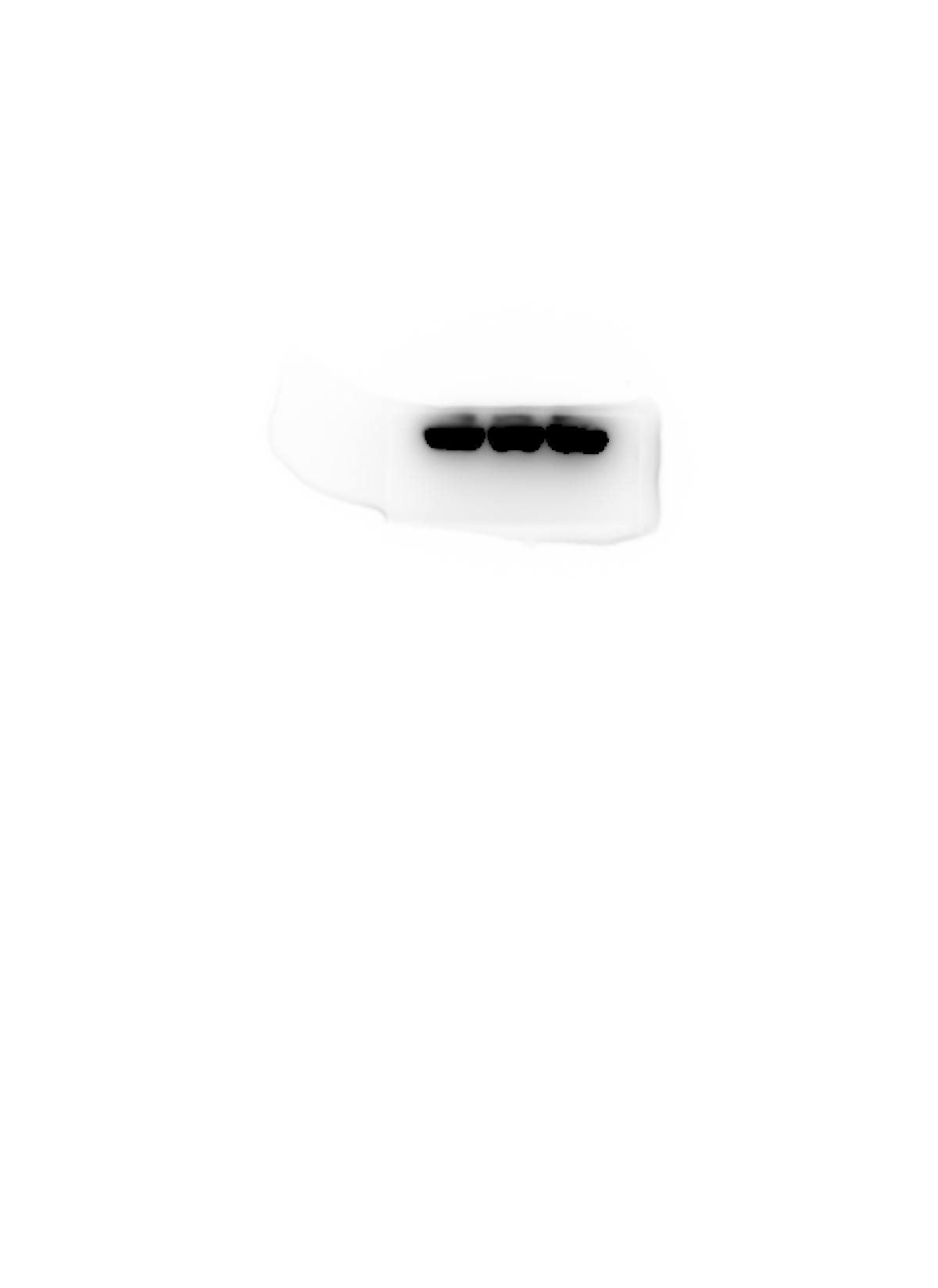

Supplement: Supplementary file 2 — Supplementary Material 2 [file 41598_2025_31281_MOESM2_ESM.zip › Fig5_R1/Fig.5E b-actin.tif]

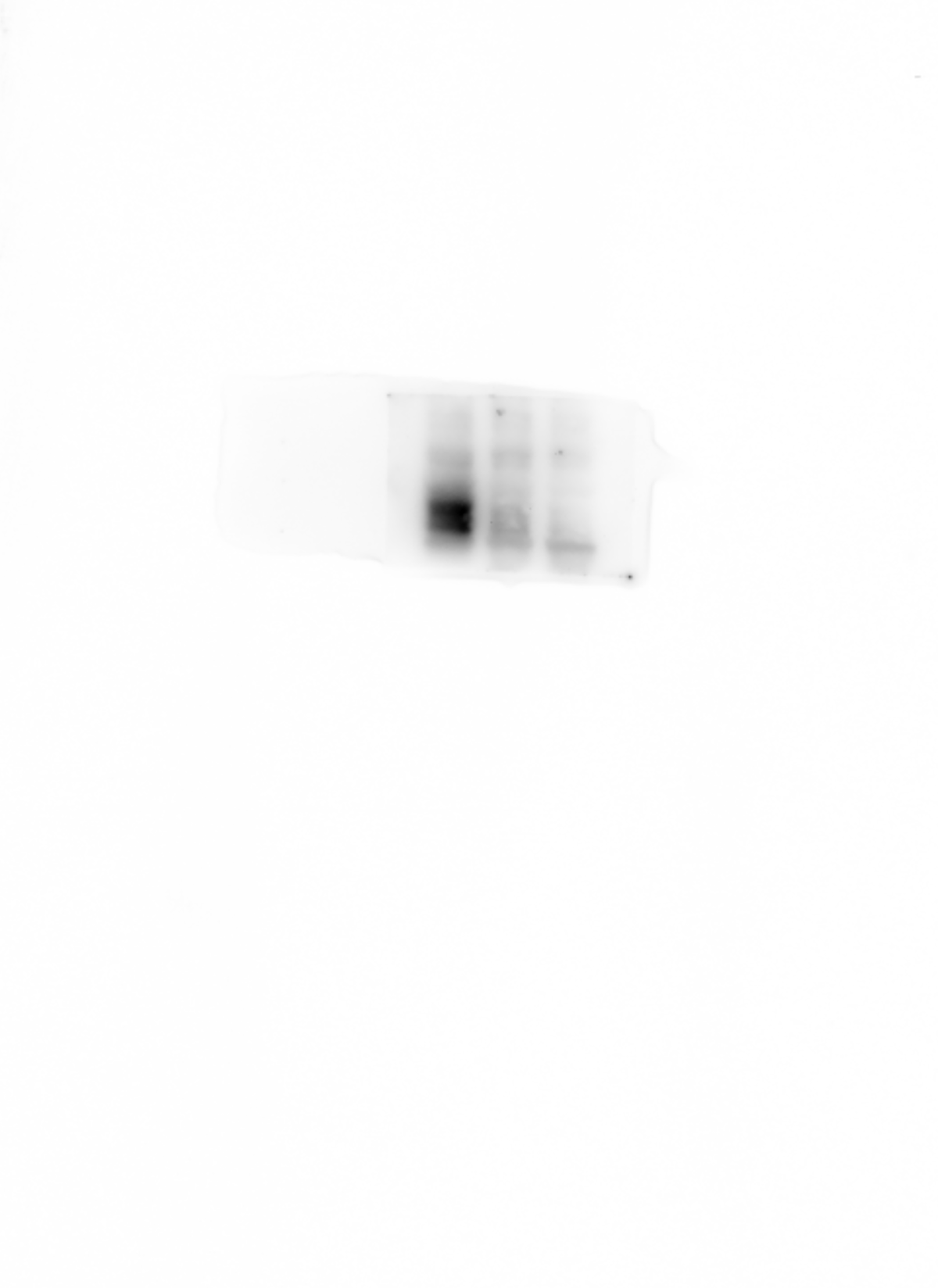

Supplement: Supplementary file 2 — Supplementary Material 2 [file 41598_2025_31281_MOESM2_ESM.zip › Fig5_R1/Fig.5E SGK1.tif]

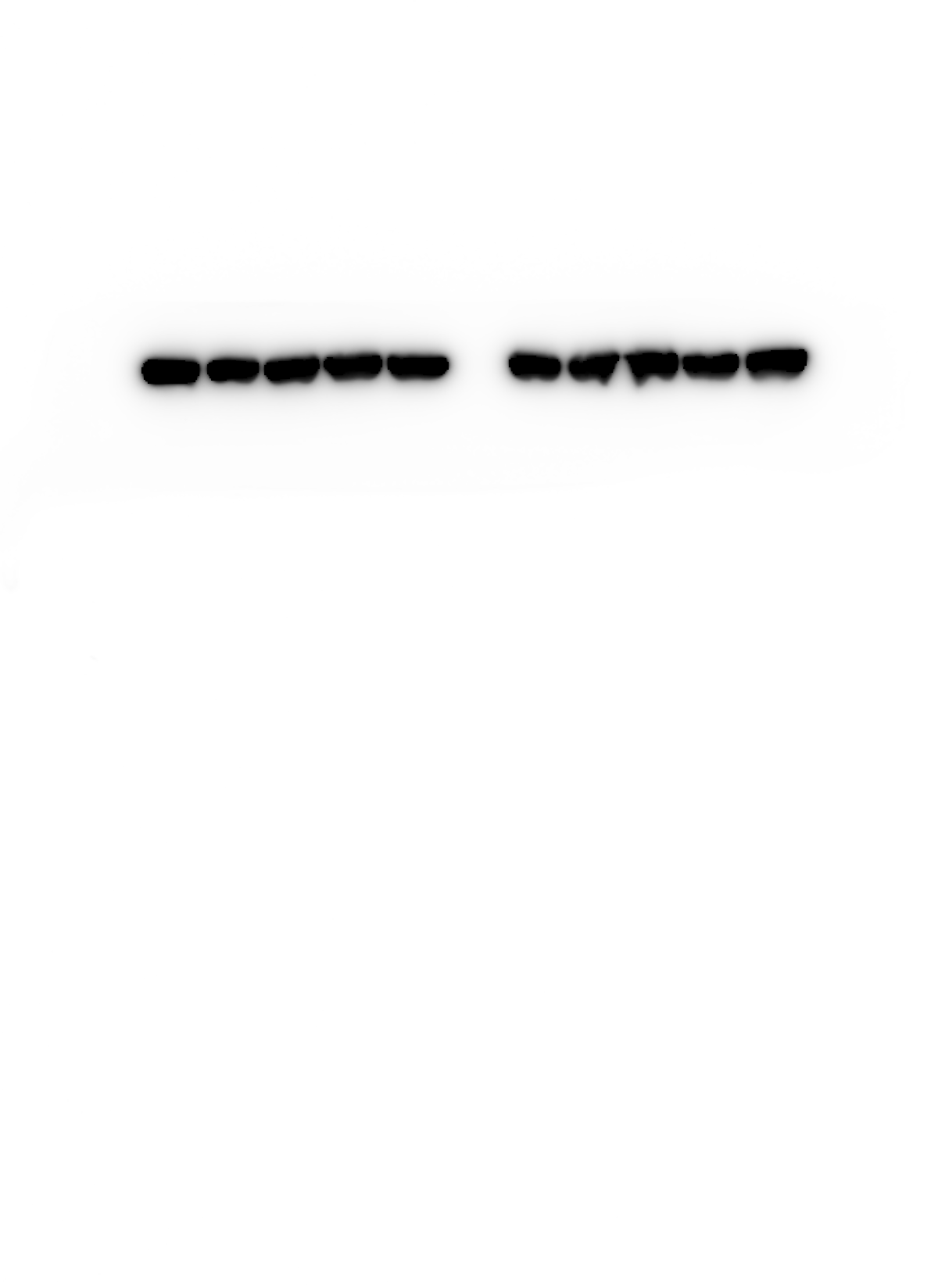

Supplement: Supplementary file 2 — Supplementary Material 2 [file 41598_2025_31281_MOESM2_ESM.zip › Fig5_R1/Fig.5F b-actin.tif]

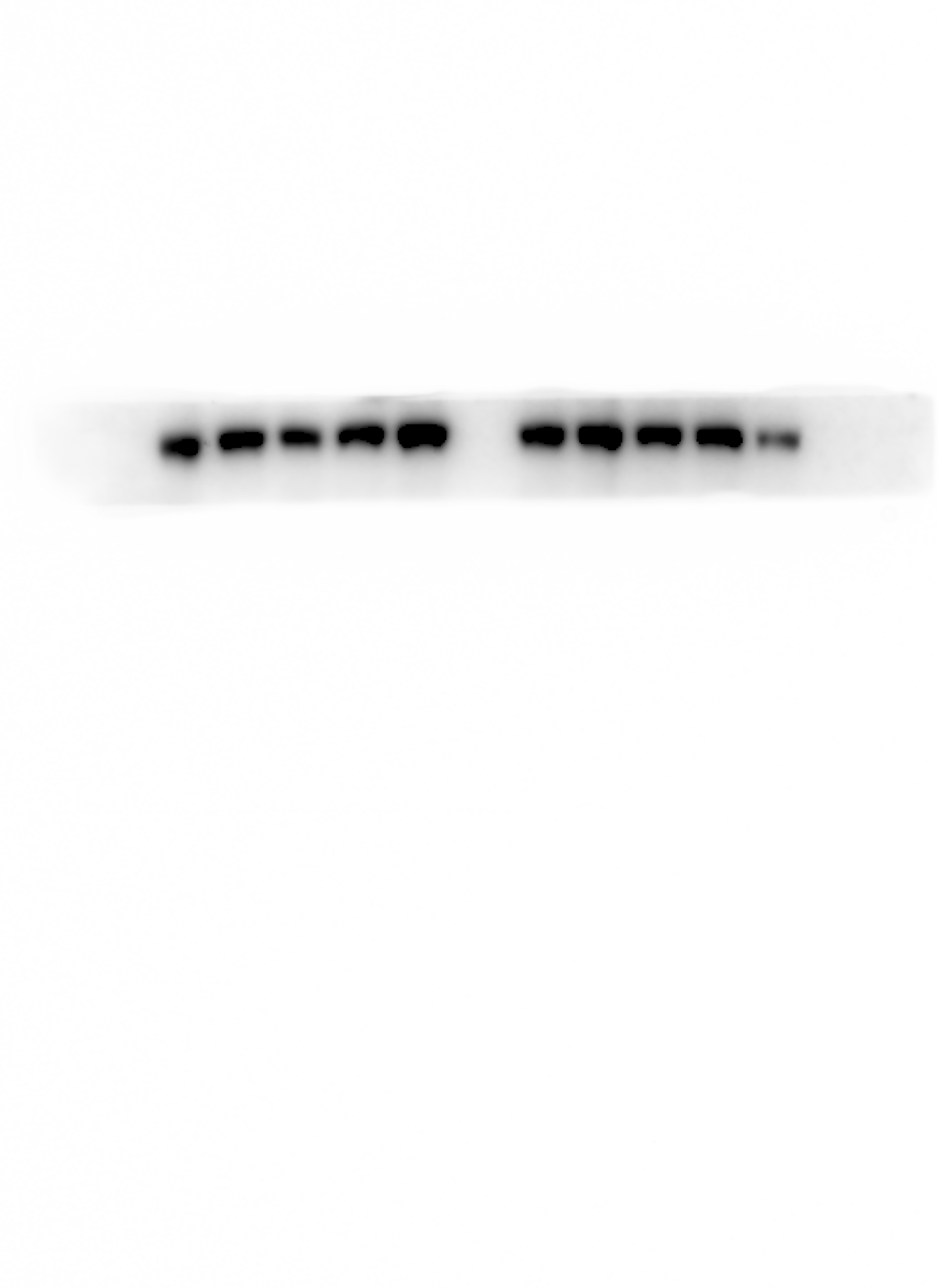

Supplement: Supplementary file 2 — Supplementary Material 2 [file 41598_2025_31281_MOESM2_ESM.zip › Fig5_R1/Fig.5F IRF3.tif]

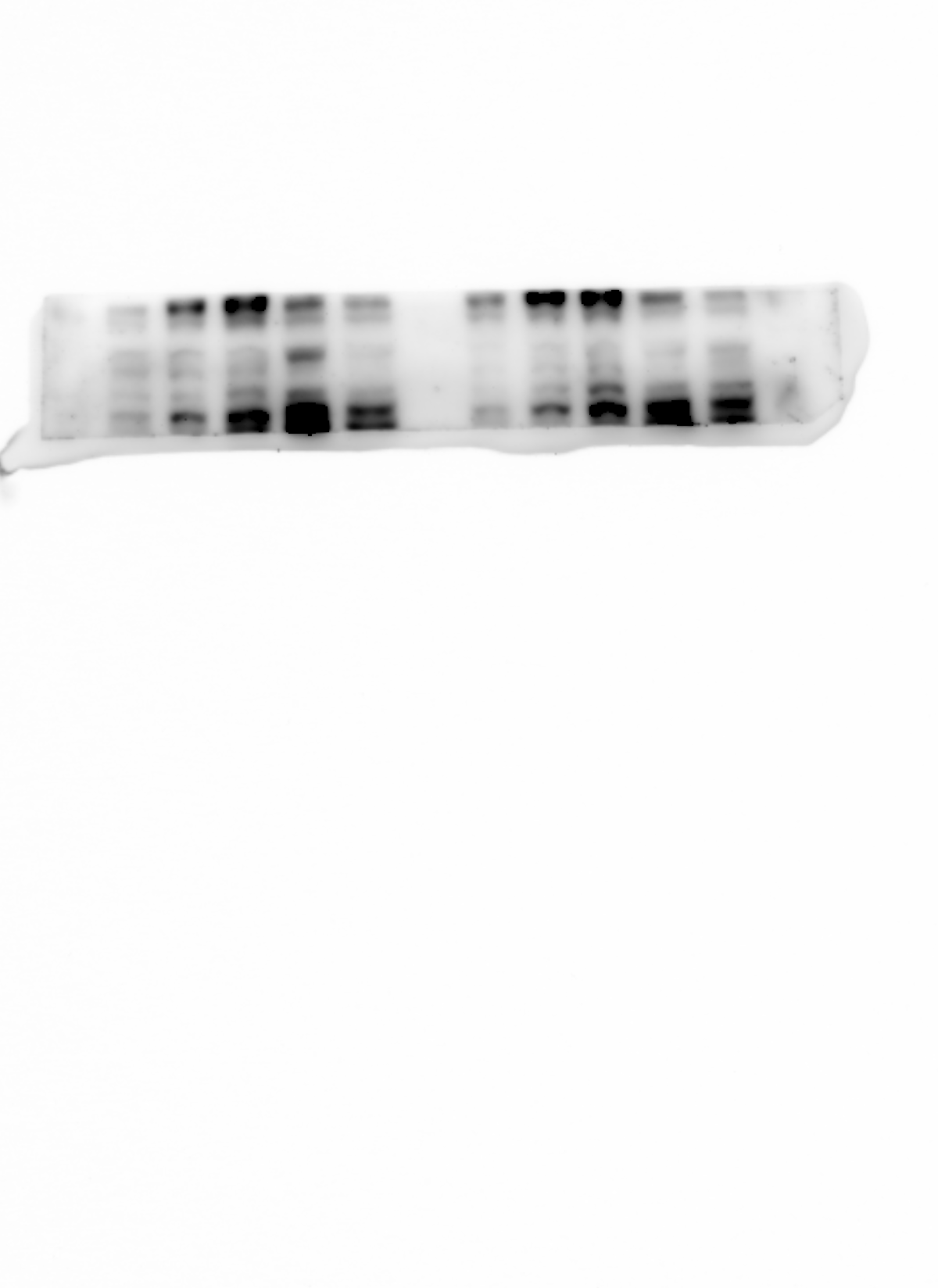

Supplement: Supplementary file 2 — Supplementary Material 2 [file 41598_2025_31281_MOESM2_ESM.zip › Fig5_R1/Fig.5F p-IRF3(S396).tif]

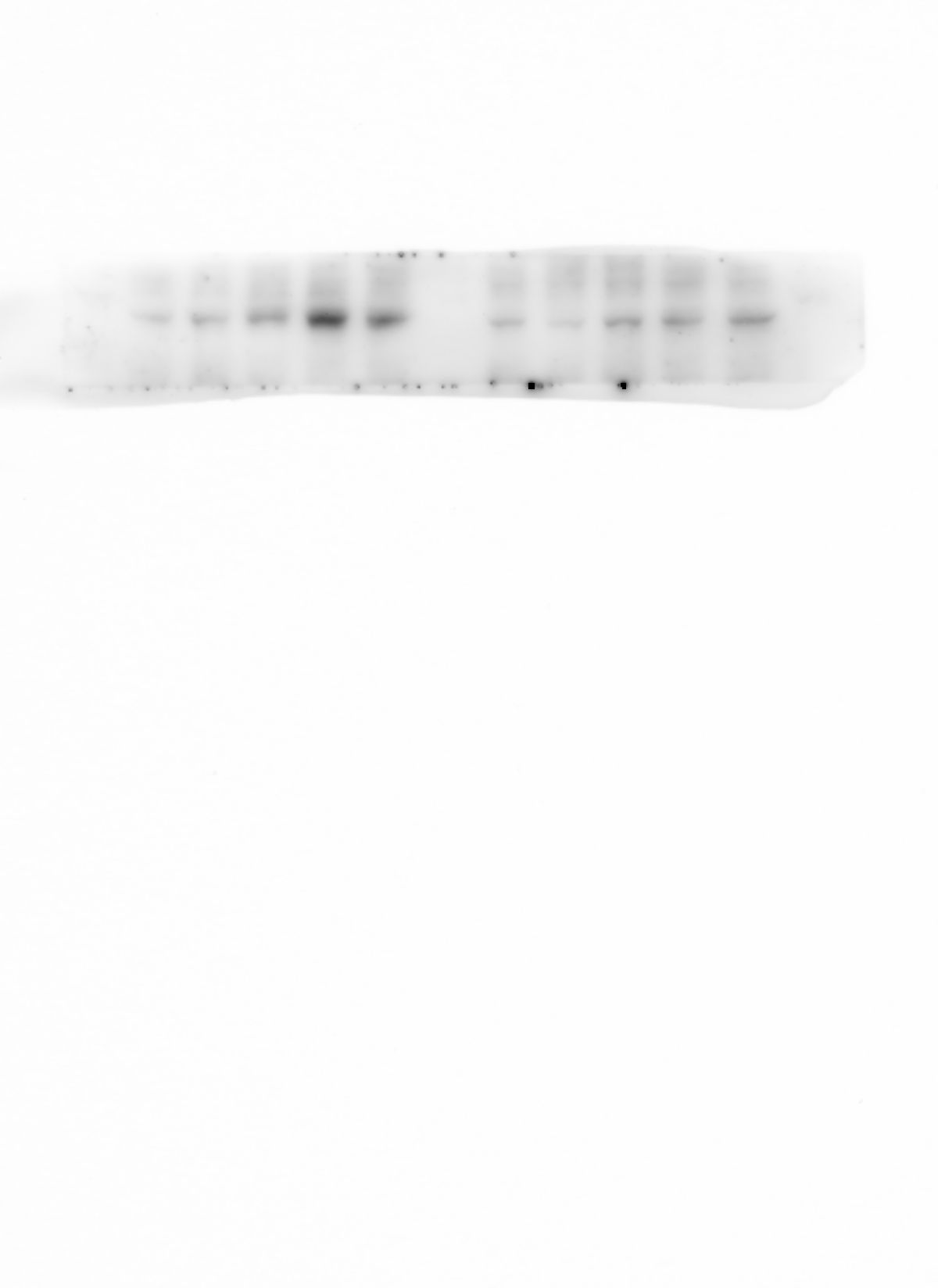

Supplement: Supplementary file 2 — Supplementary Material 2 [file 41598_2025_31281_MOESM2_ESM.zip › Fig5_R1/Fig.5F p-SGK1.tif]

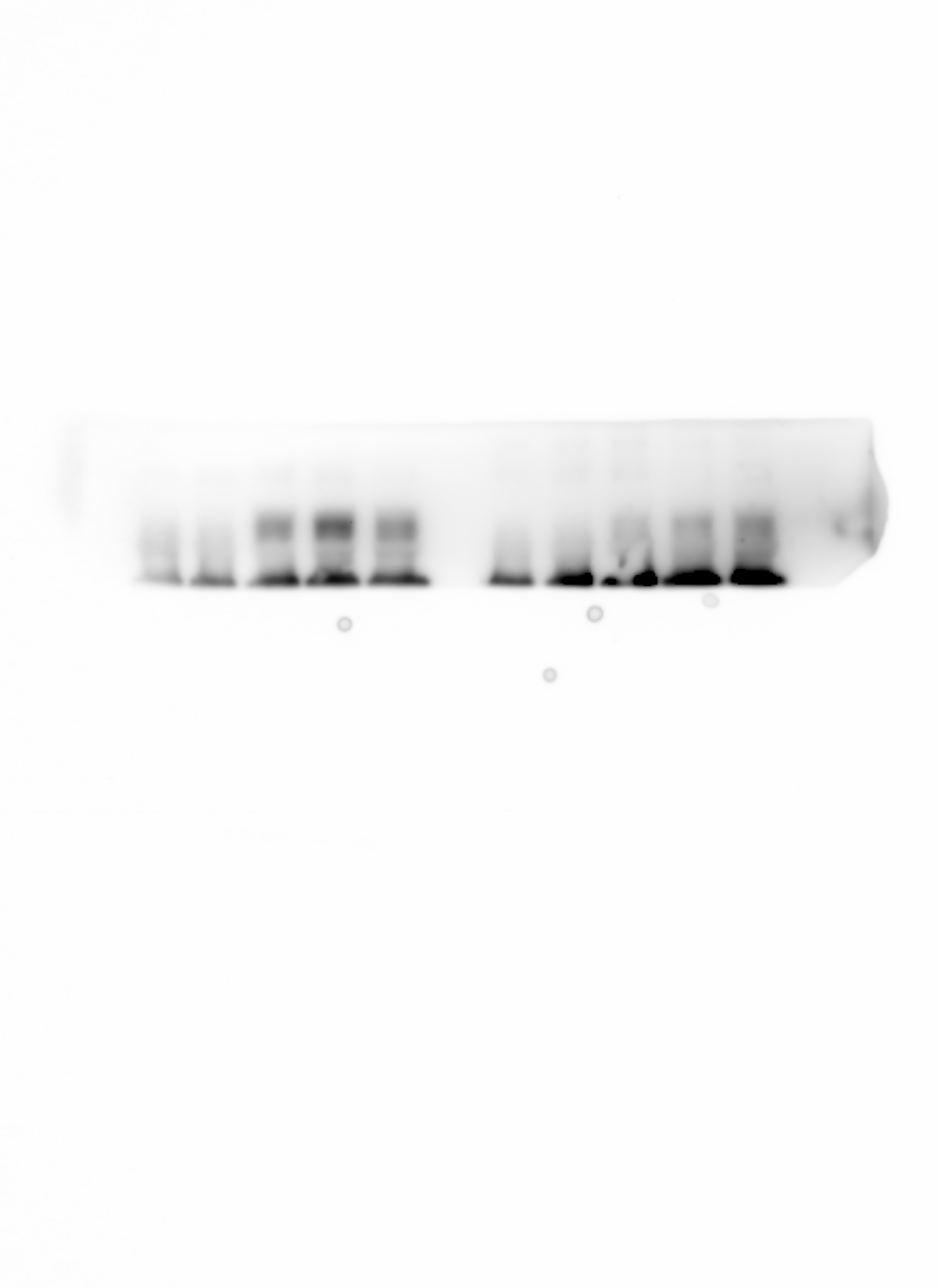

Supplement: Supplementary file 2 — Supplementary Material 2 [file 41598_2025_31281_MOESM2_ESM.zip › Fig5_R1/Fig.5F SGK1.tif]

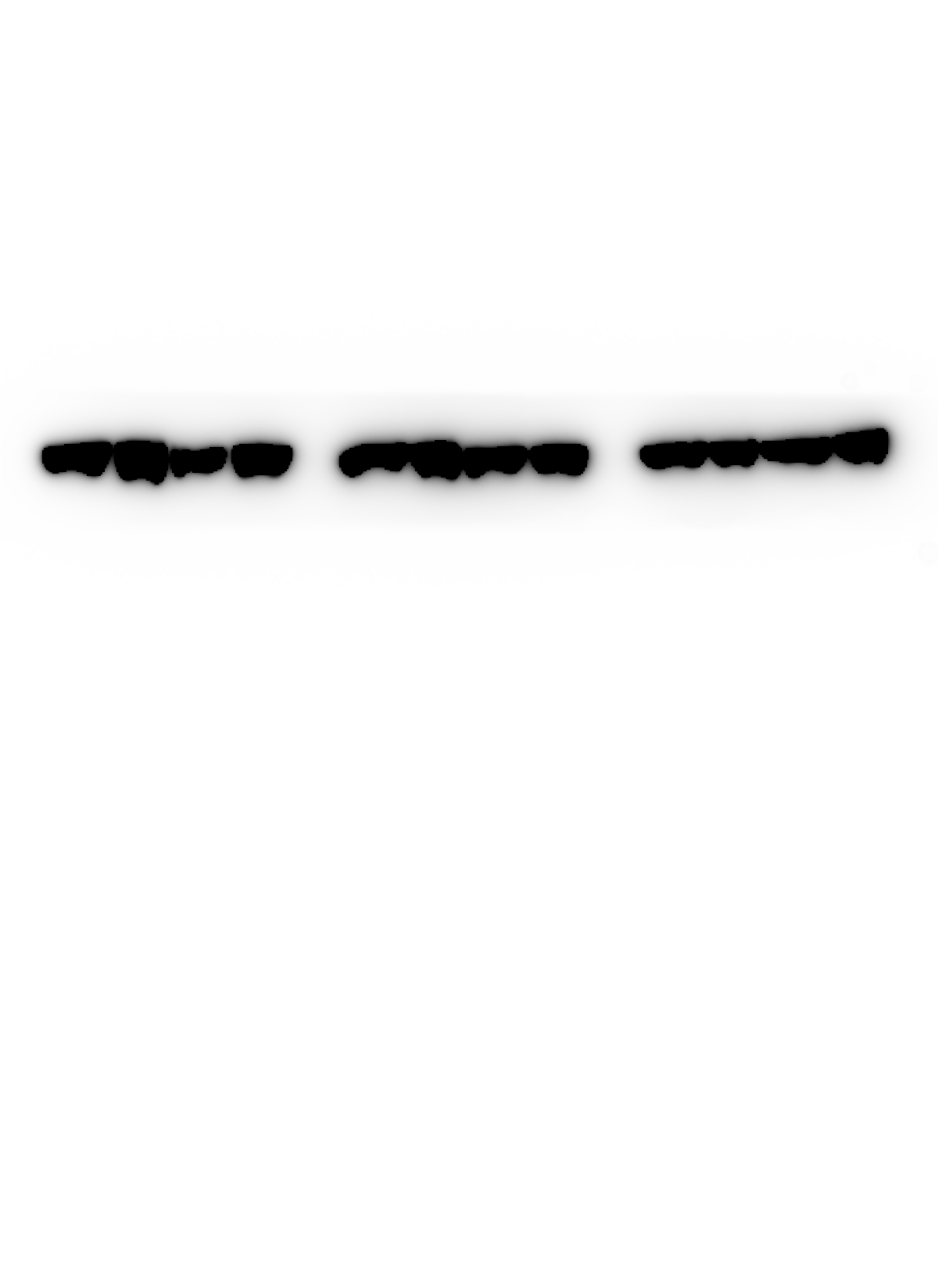

Supplement: Supplementary file 2 — Supplementary Material 2 [file 41598_2025_31281_MOESM2_ESM.zip › Fig5_R1/Fig.5G b-actin (Mock-CIE-CME).tif]

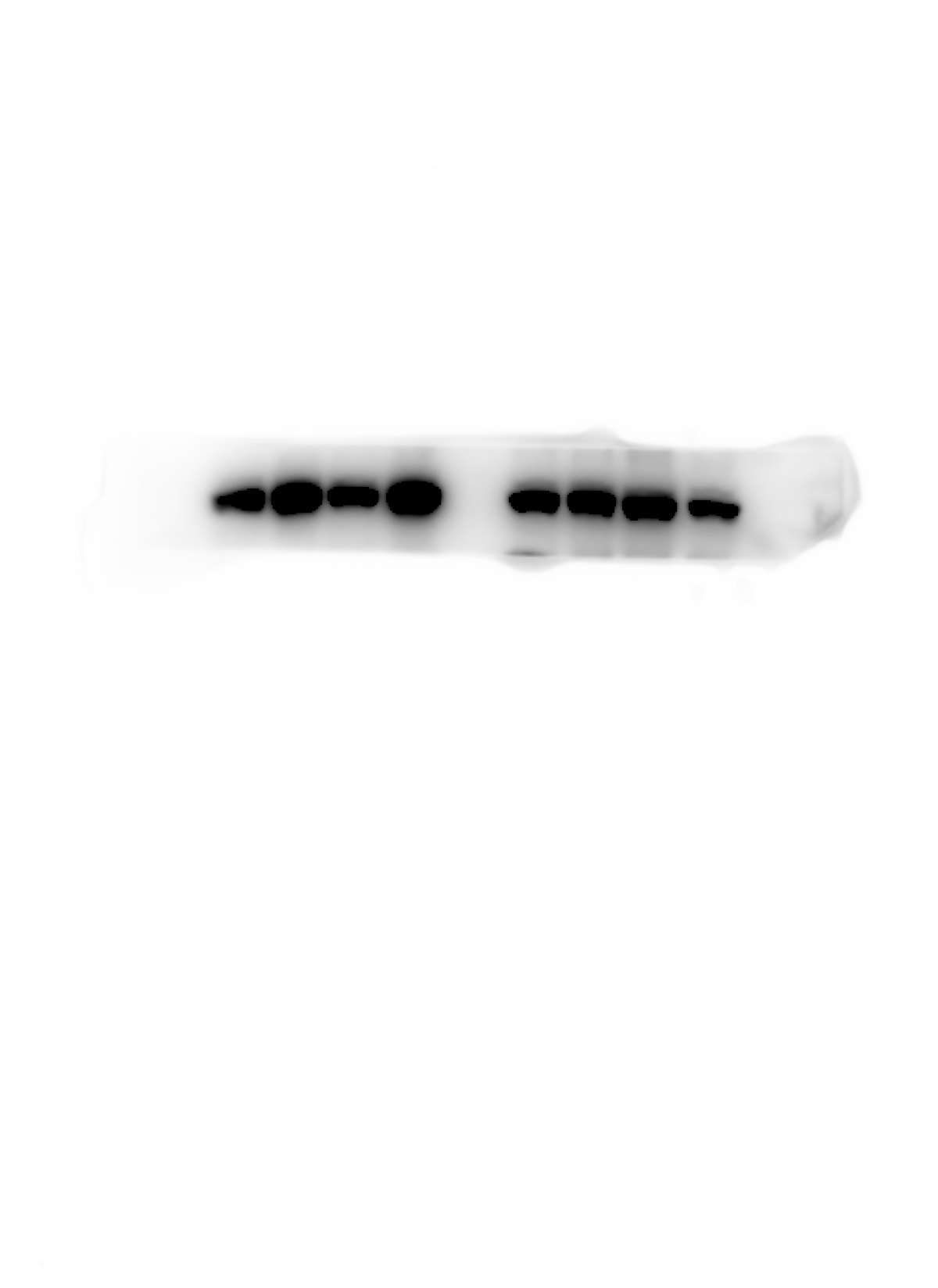

Supplement: Supplementary file 2 — Supplementary Material 2 [file 41598_2025_31281_MOESM2_ESM.zip › Fig5_R1/Fig.5G IRF3.tif]

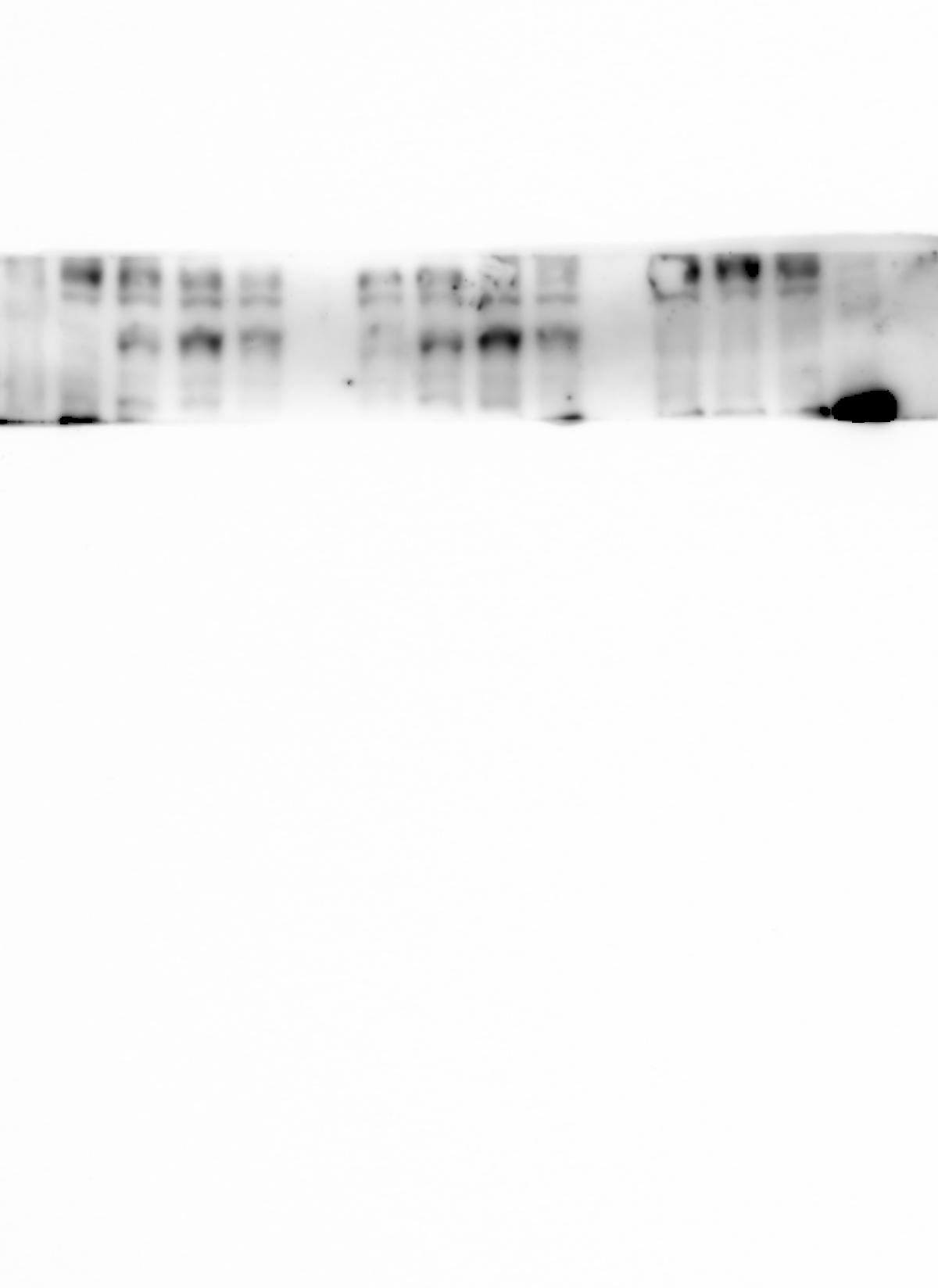

Supplement: Supplementary file 2 — Supplementary Material 2 [file 41598_2025_31281_MOESM2_ESM.zip › Fig5_R1/Fig.5G p-IRF3(S396) (Mock-CIE-CME).tif]

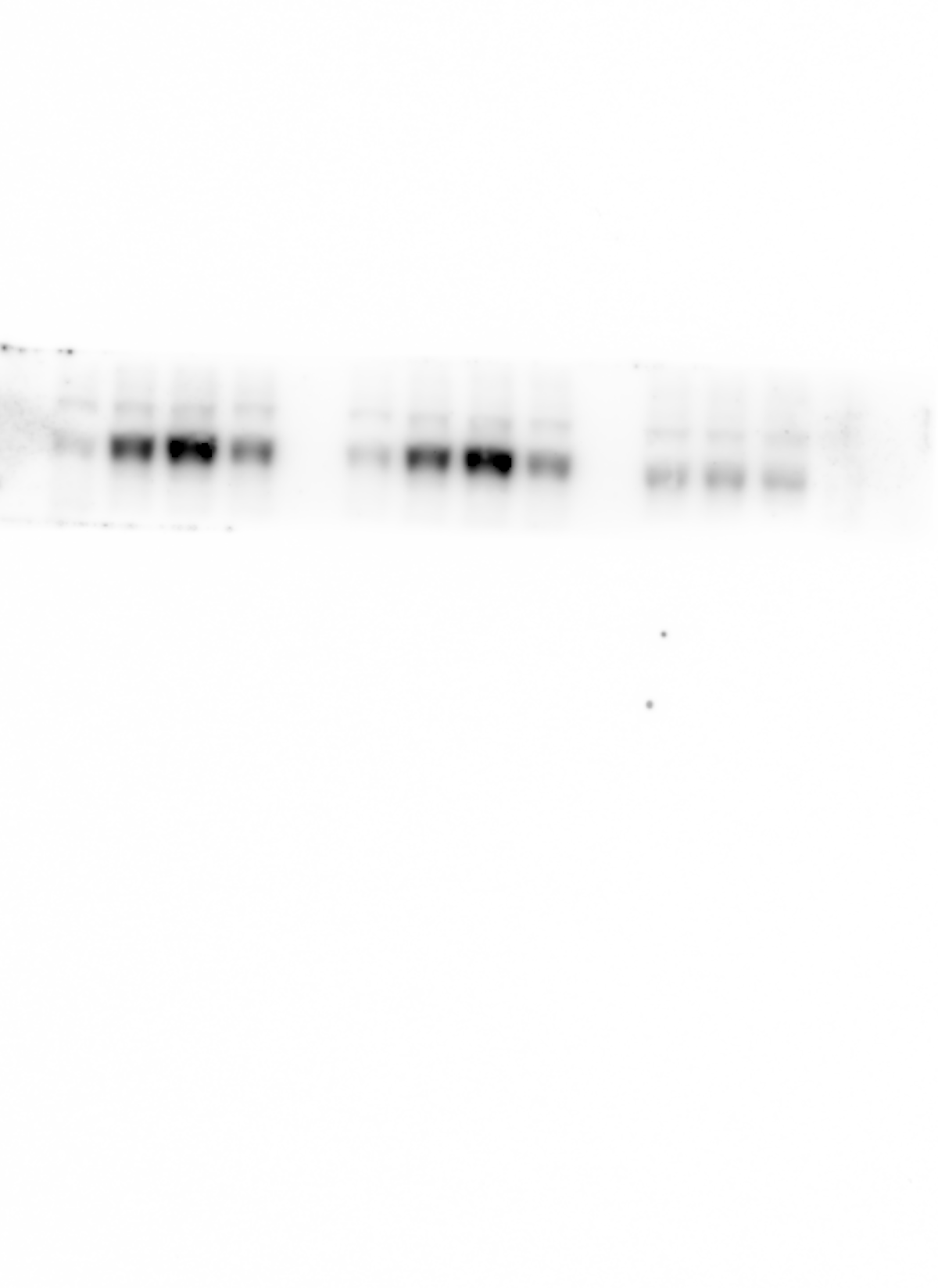

Supplement: Supplementary file 2 — Supplementary Material 2 [file 41598_2025_31281_MOESM2_ESM.zip › Fig5_R1/Fig.5G p-SGK1 (Mock-CIE-CME).tif]

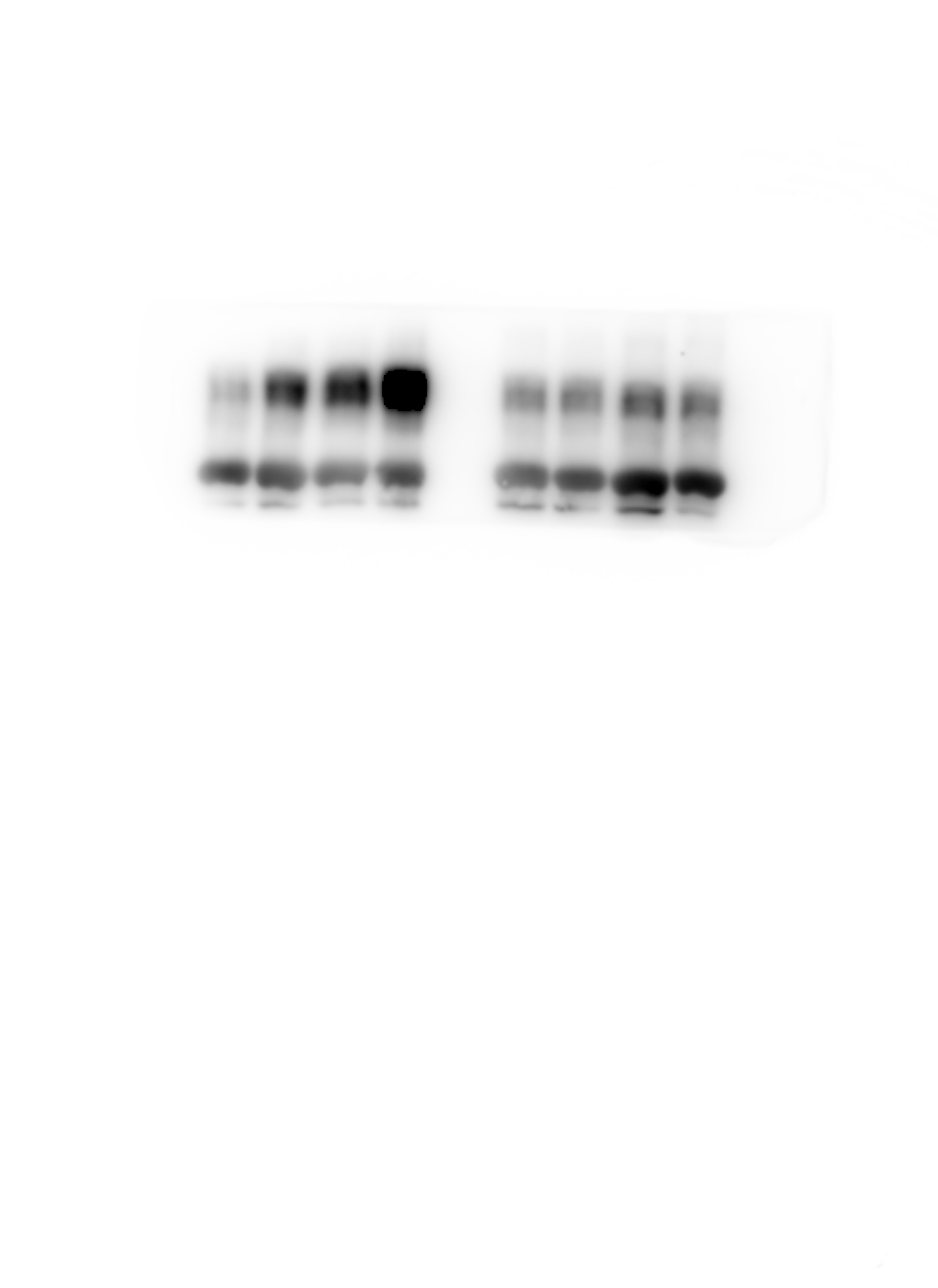

Supplement: Supplementary file 2 — Supplementary Material 2 [file 41598_2025_31281_MOESM2_ESM.zip › Fig5_R1/Fig.5G SGK1.tif]

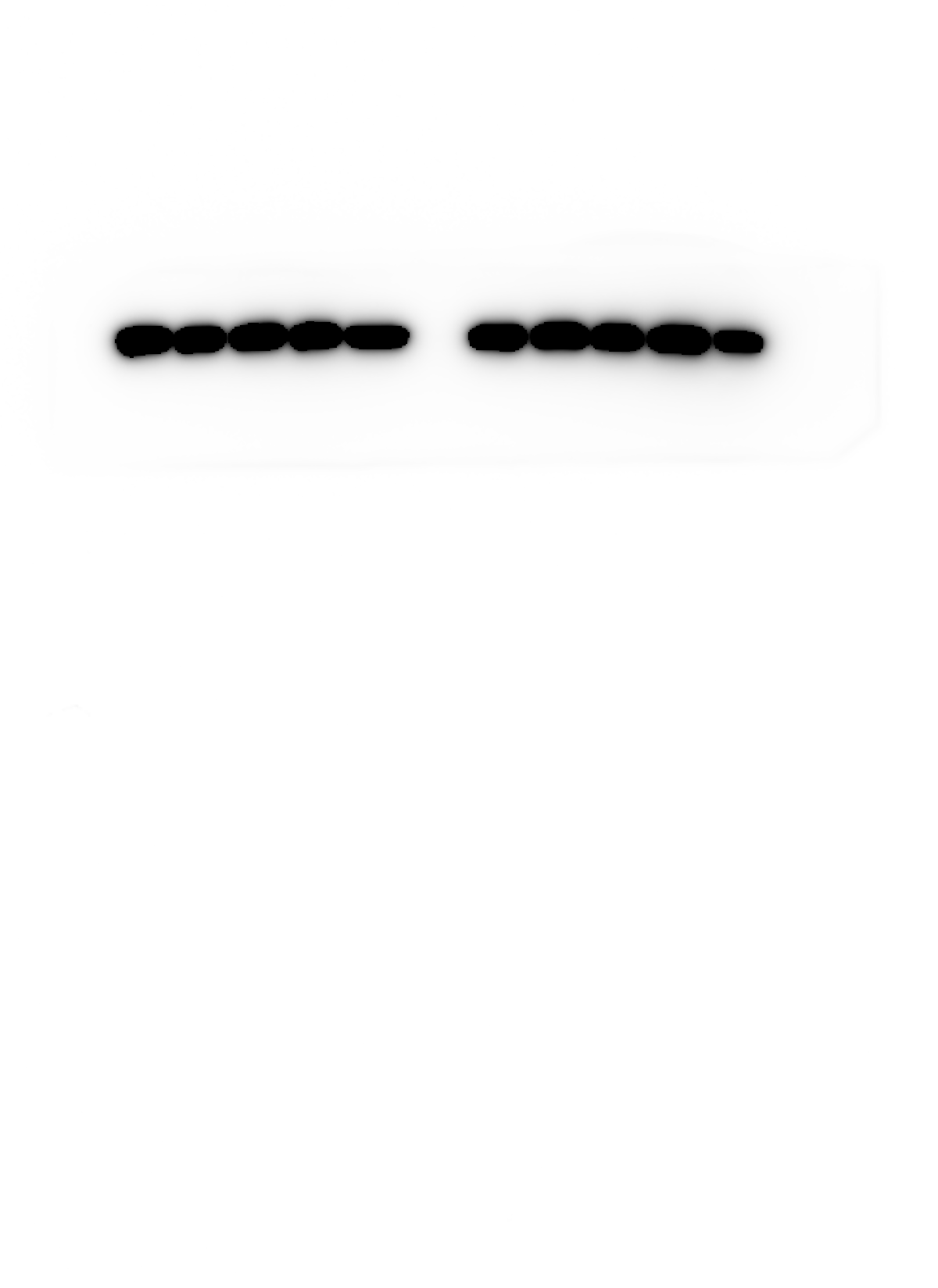

Supplement: Supplementary file 2 — Supplementary Material 2 [file 41598_2025_31281_MOESM2_ESM.zip › Fig5_R1/Fig.5H b-actin.tif]

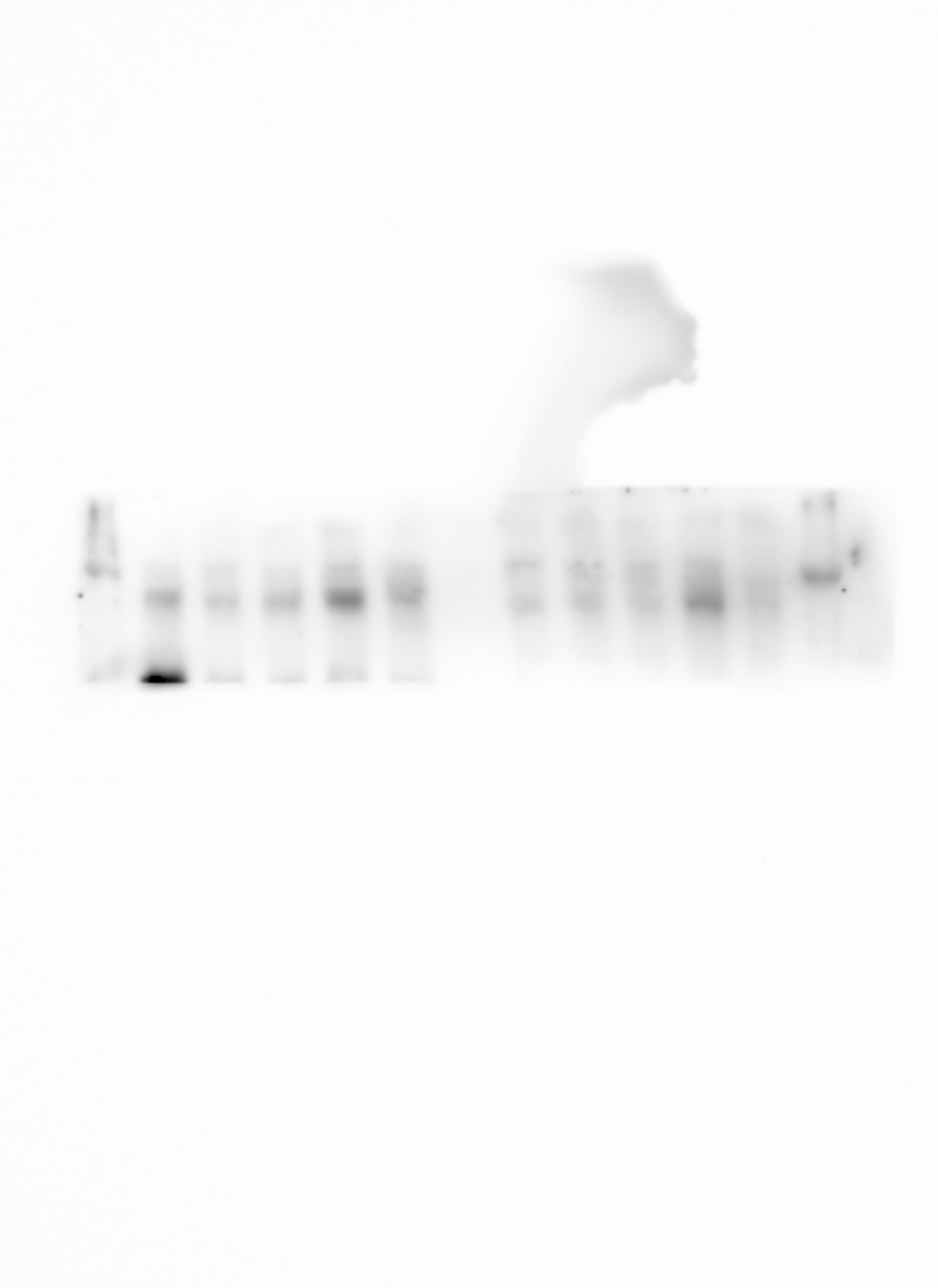

Supplement: Supplementary file 2 — Supplementary Material 2 [file 41598_2025_31281_MOESM2_ESM.zip › Fig5_R1/Fig.5H p-SGK1.tif]

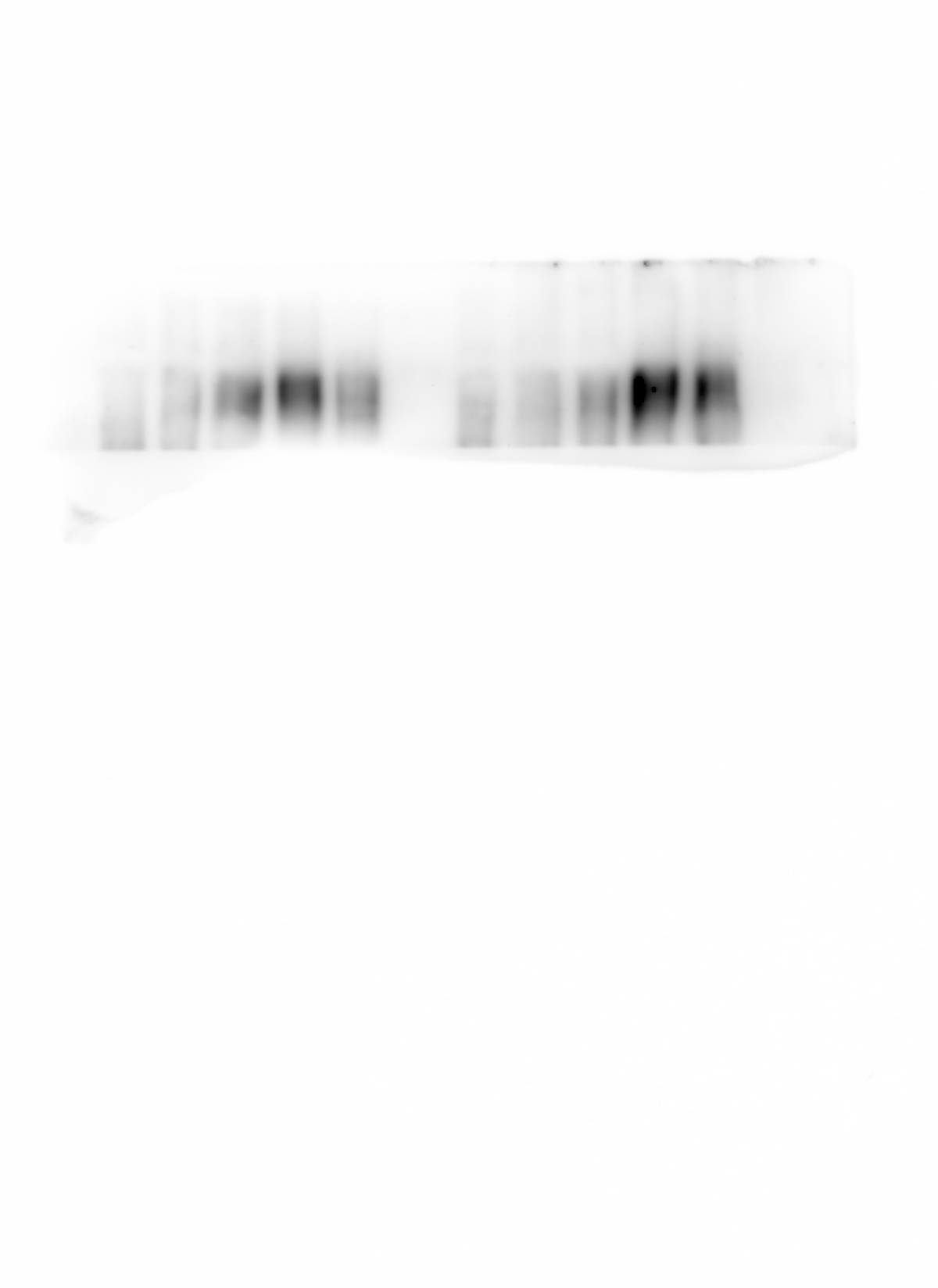

Supplement: Supplementary file 2 — Supplementary Material 2 [file 41598_2025_31281_MOESM2_ESM.zip › Fig5_R1/Fig.5H SGK1.tif]

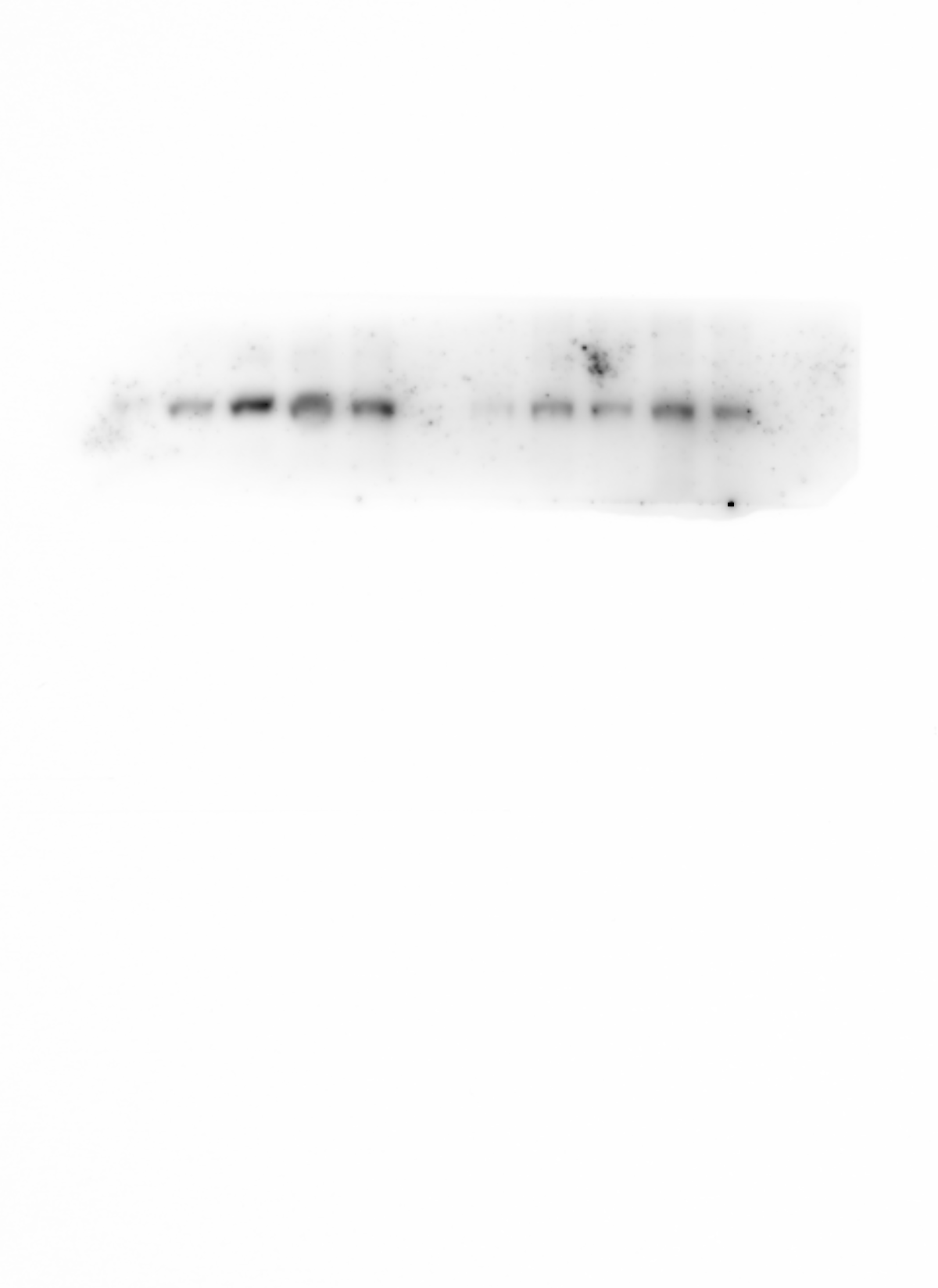

Supplement: Supplementary file 2 — Supplementary Material 2 [file 41598_2025_31281_MOESM2_ESM.zip › Fig5_R1/Fig.5H TRIF.tif]

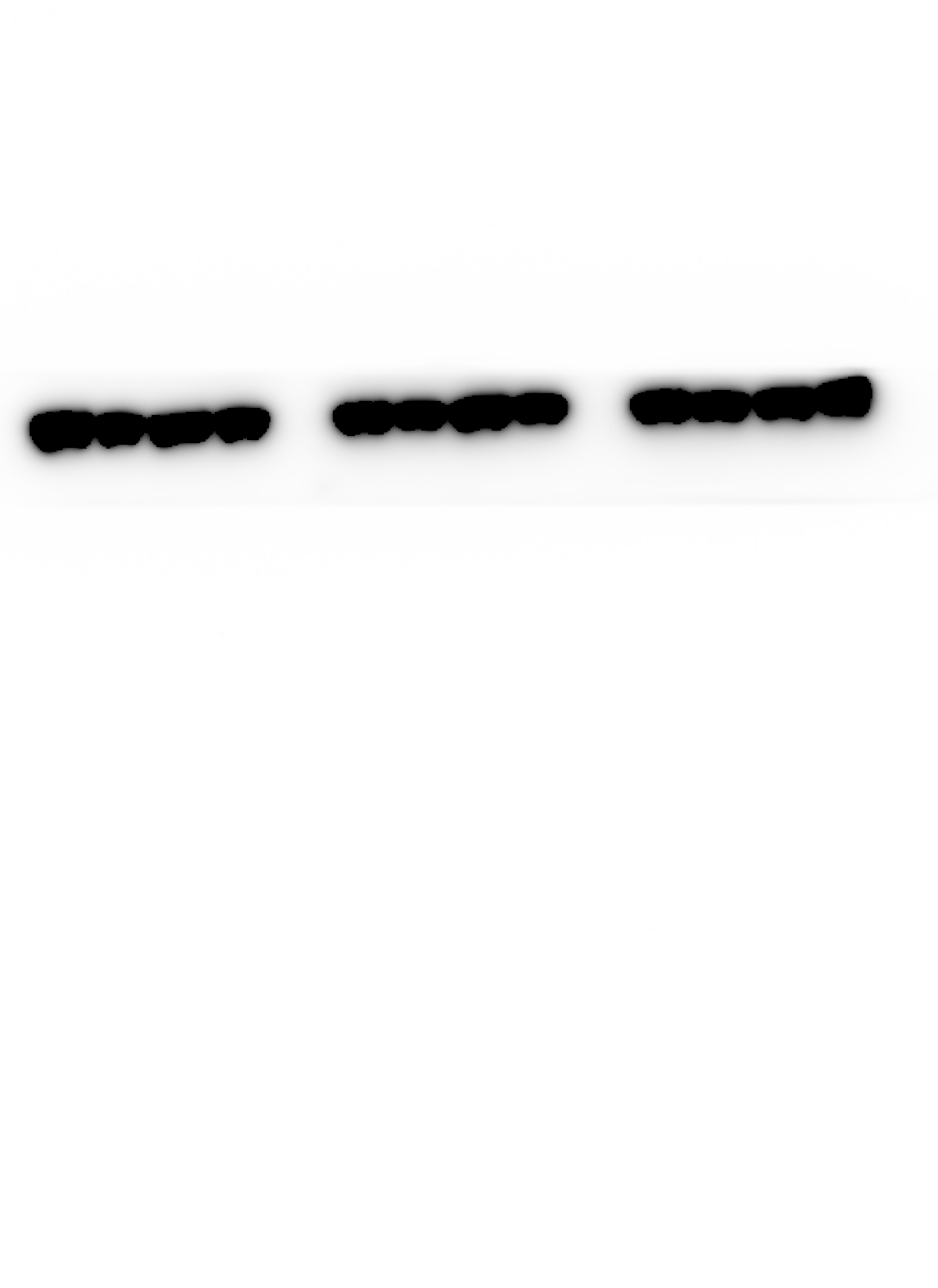

Supplement: Supplementary file 2 — Supplementary Material 2 [file 41598_2025_31281_MOESM2_ESM.zip › Fig5_R1/Fig.5I b-actin (Mock-Myd 50-Myd 100).tif]

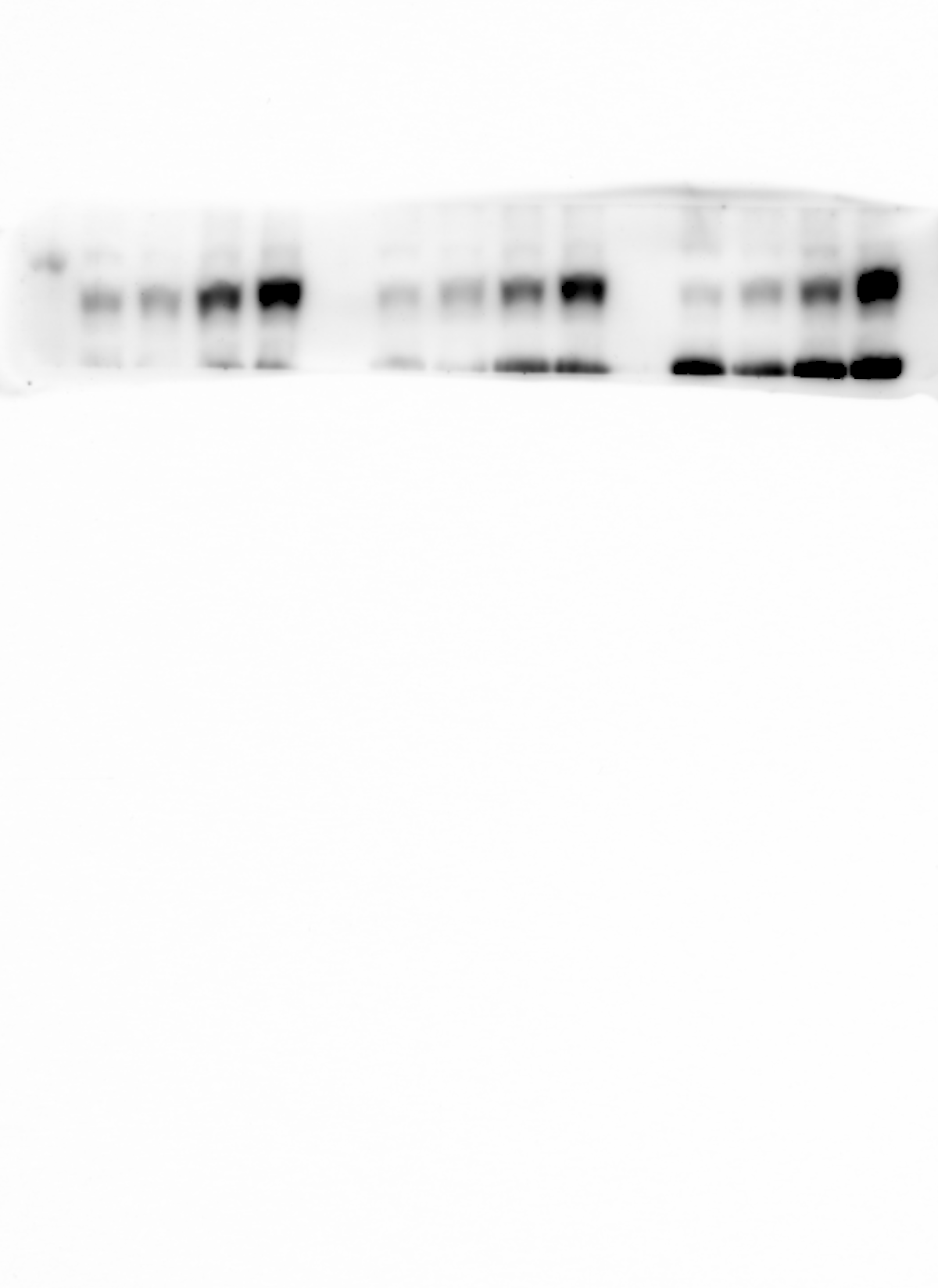

Supplement: Supplementary file 2 — Supplementary Material 2 [file 41598_2025_31281_MOESM2_ESM.zip › Fig5_R1/Fig.5I p-SGK1 (Mock-Myd 50-Myd 100).tif]

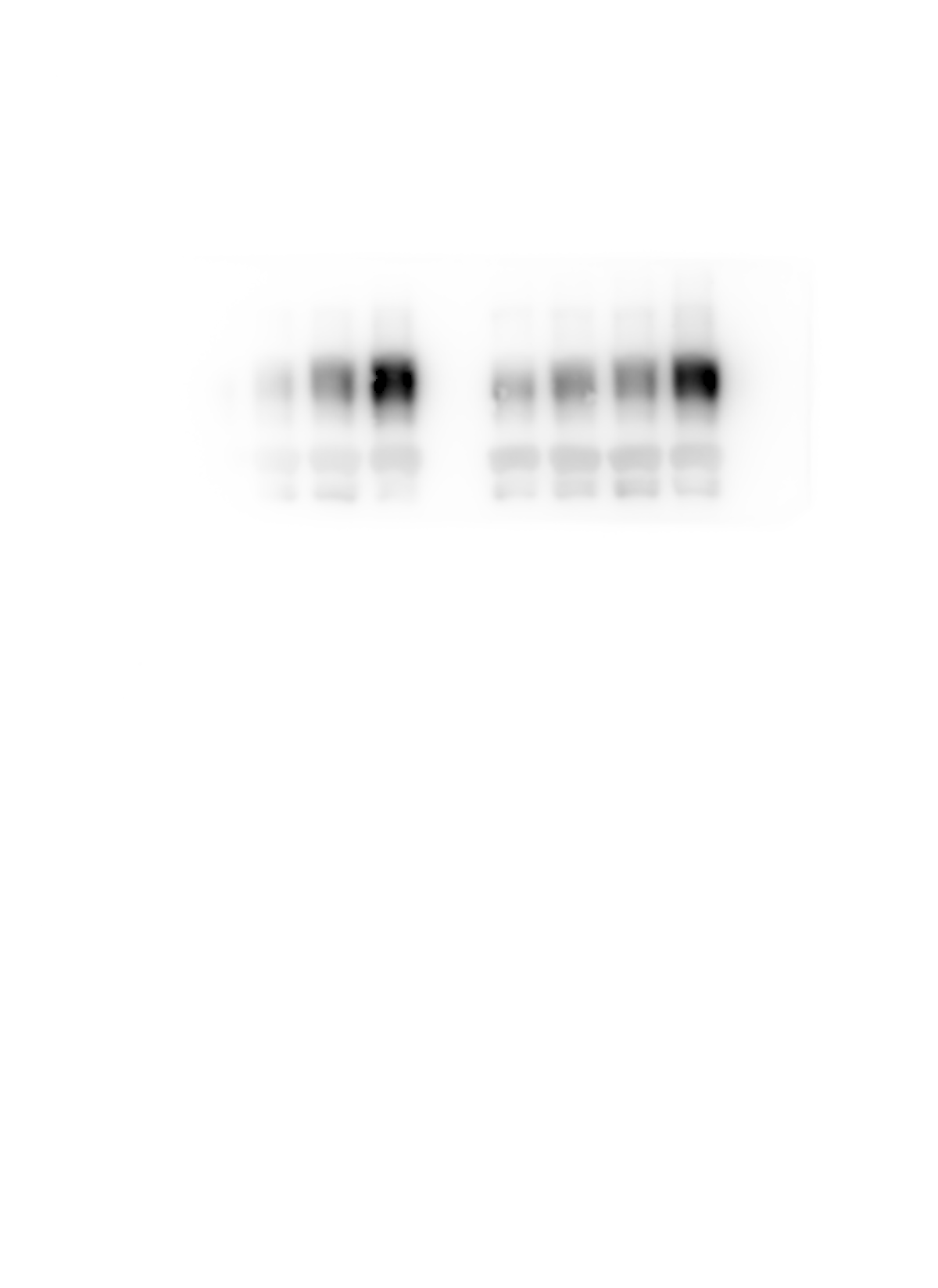

Supplement: Supplementary file 2 — Supplementary Material 2 [file 41598_2025_31281_MOESM2_ESM.zip › Fig5_R1/Fig.5I SGK1.tif]

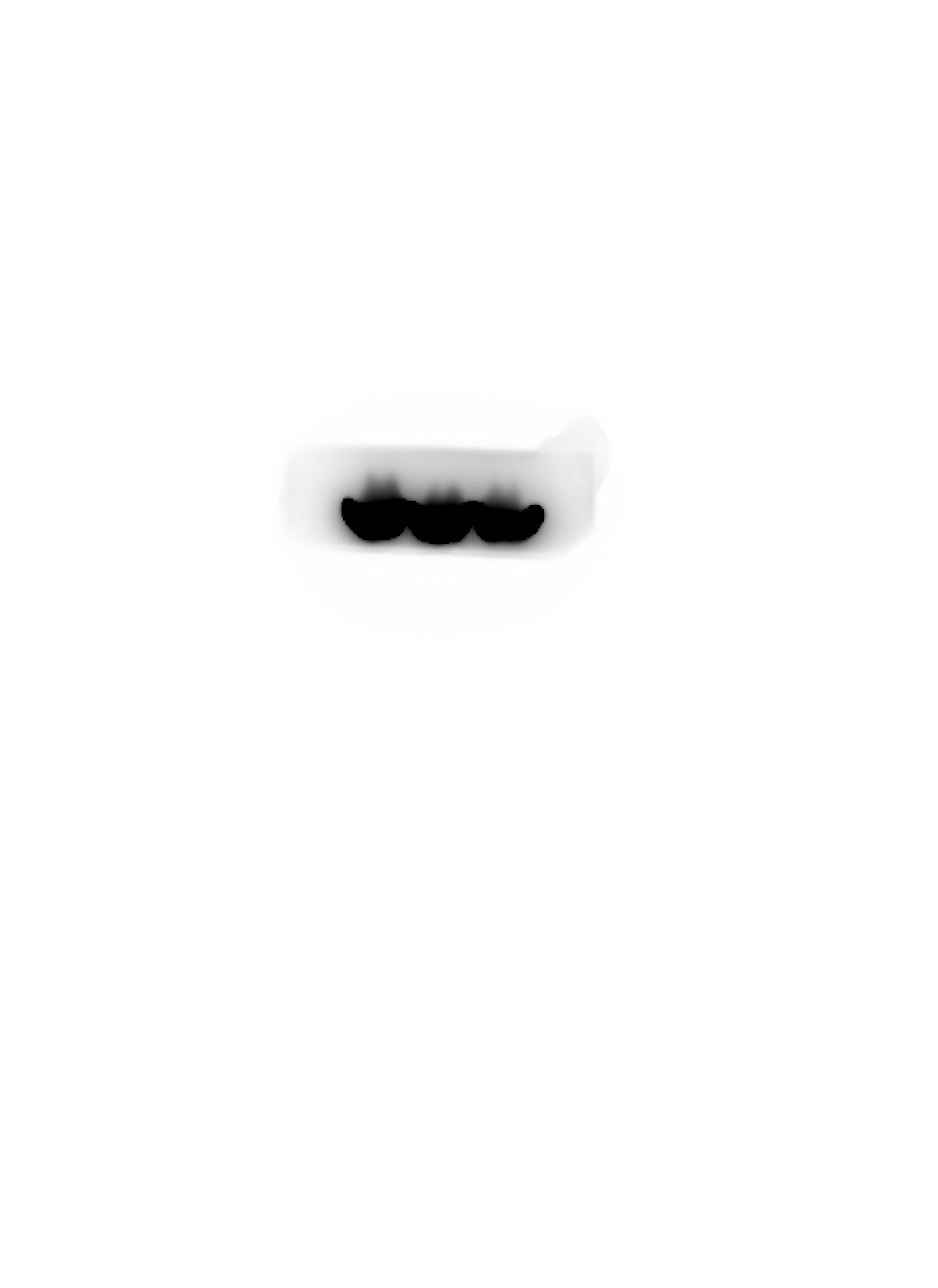

Supplement: Supplementary file 2 — Supplementary Material 2 [file 41598_2025_31281_MOESM2_ESM.zip › Fig6_R1/Fig.6C b-actin.tif]

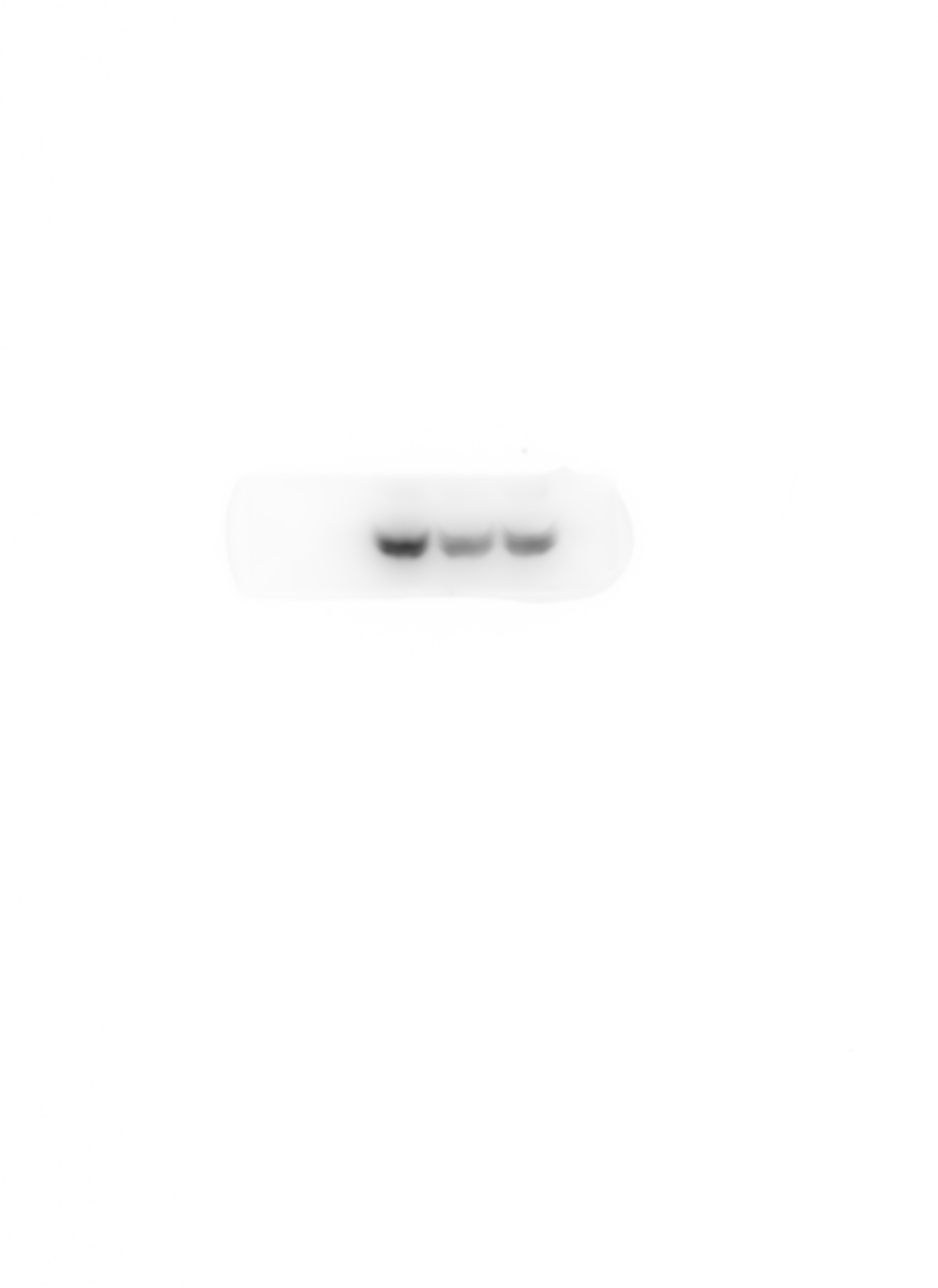

Supplement: Supplementary file 2 — Supplementary Material 2 [file 41598_2025_31281_MOESM2_ESM.zip › Fig6_R1/Fig.6C IRF3.tif]

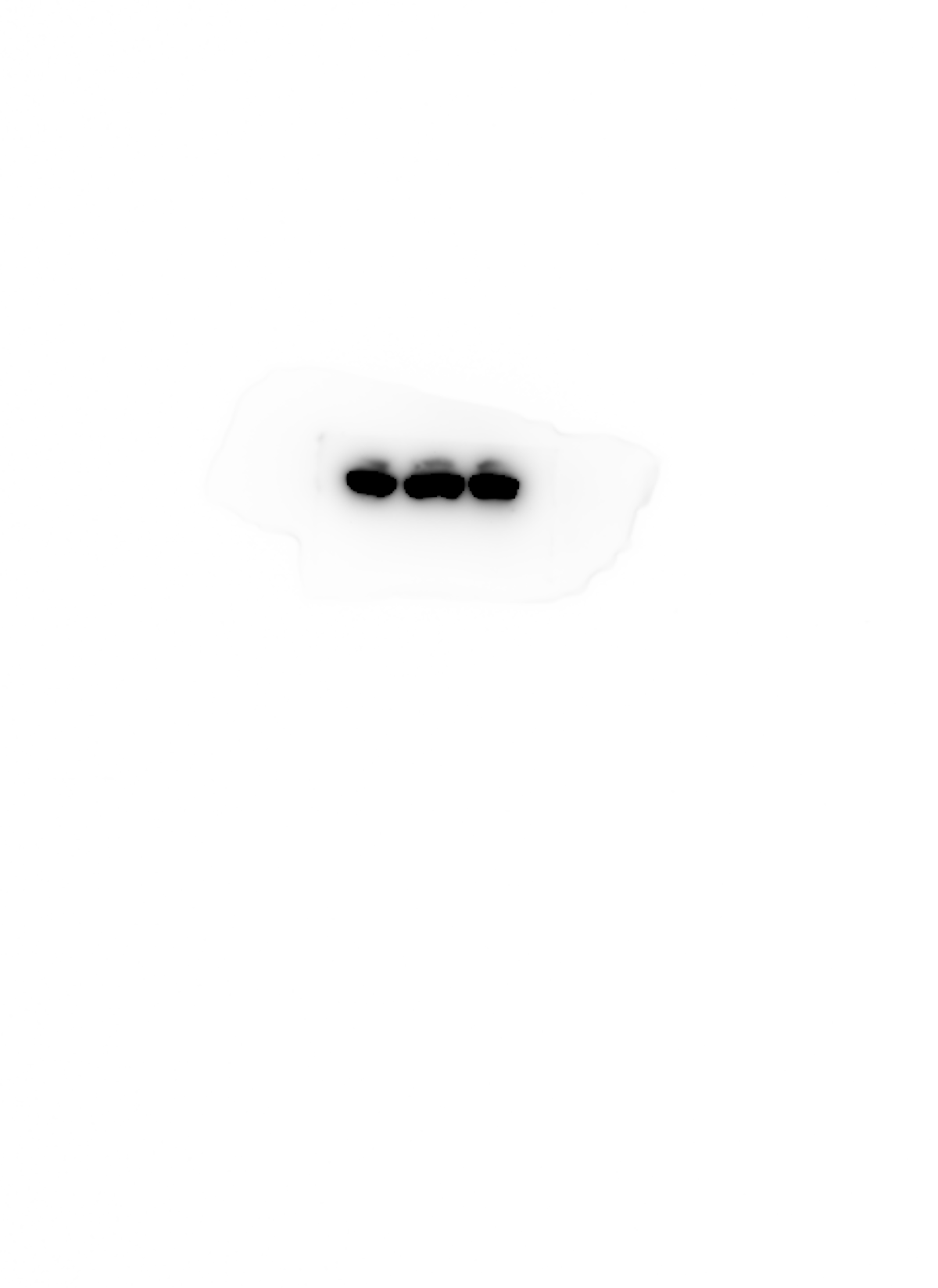

Supplement: Supplementary file 2 — Supplementary Material 2 [file 41598_2025_31281_MOESM2_ESM.zip › Fig6_R1/Fig.6G b-actin.tif]

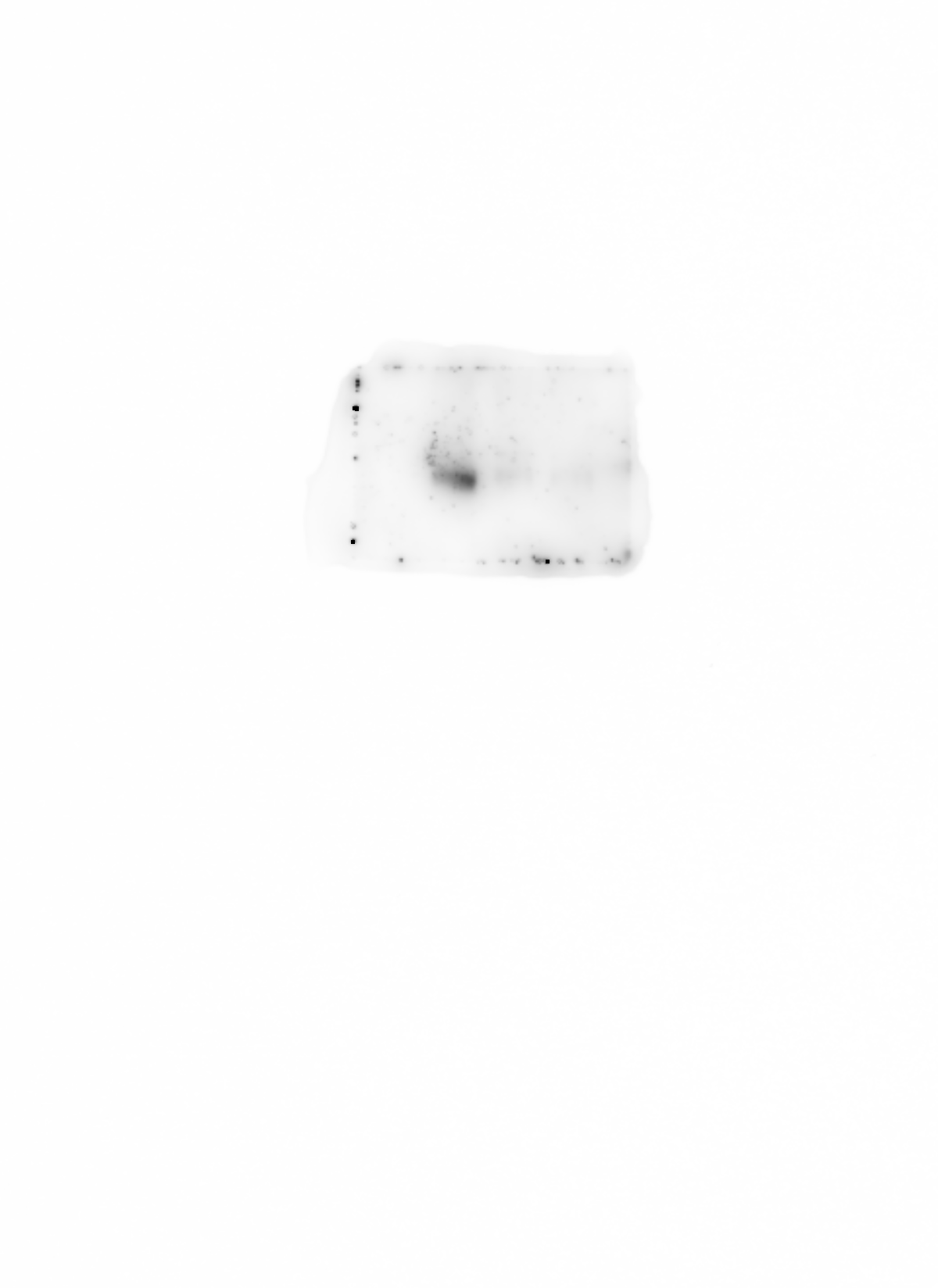

Supplement: Supplementary file 2 — Supplementary Material 2 [file 41598_2025_31281_MOESM2_ESM.zip › Fig6_R1/Fig.6G TRIF.tif]

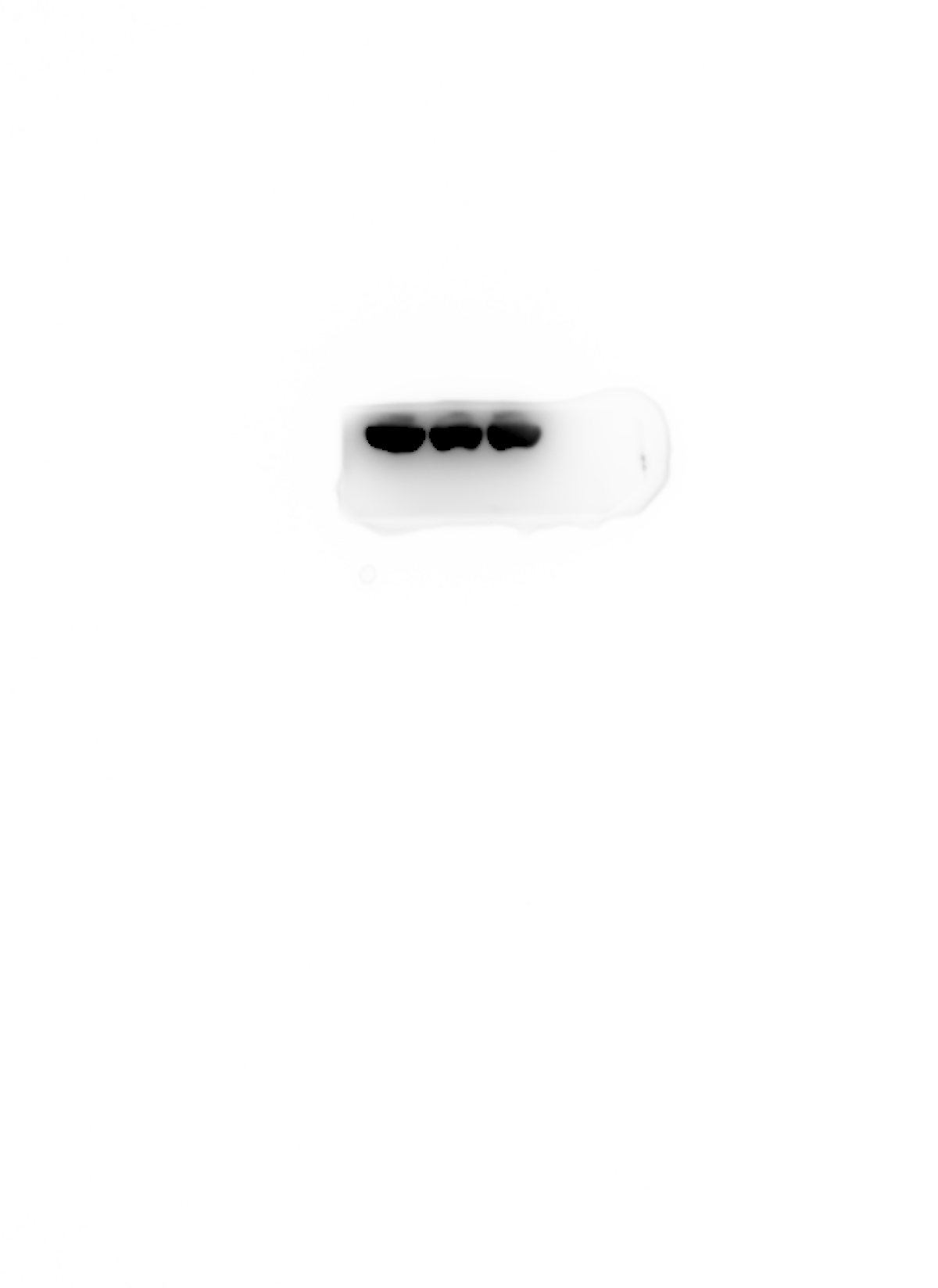

Supplement: Supplementary file 2 — Supplementary Material 2 [file 41598_2025_31281_MOESM2_ESM.zip › Fig6_R1/Fig.6H b-actin.tif]

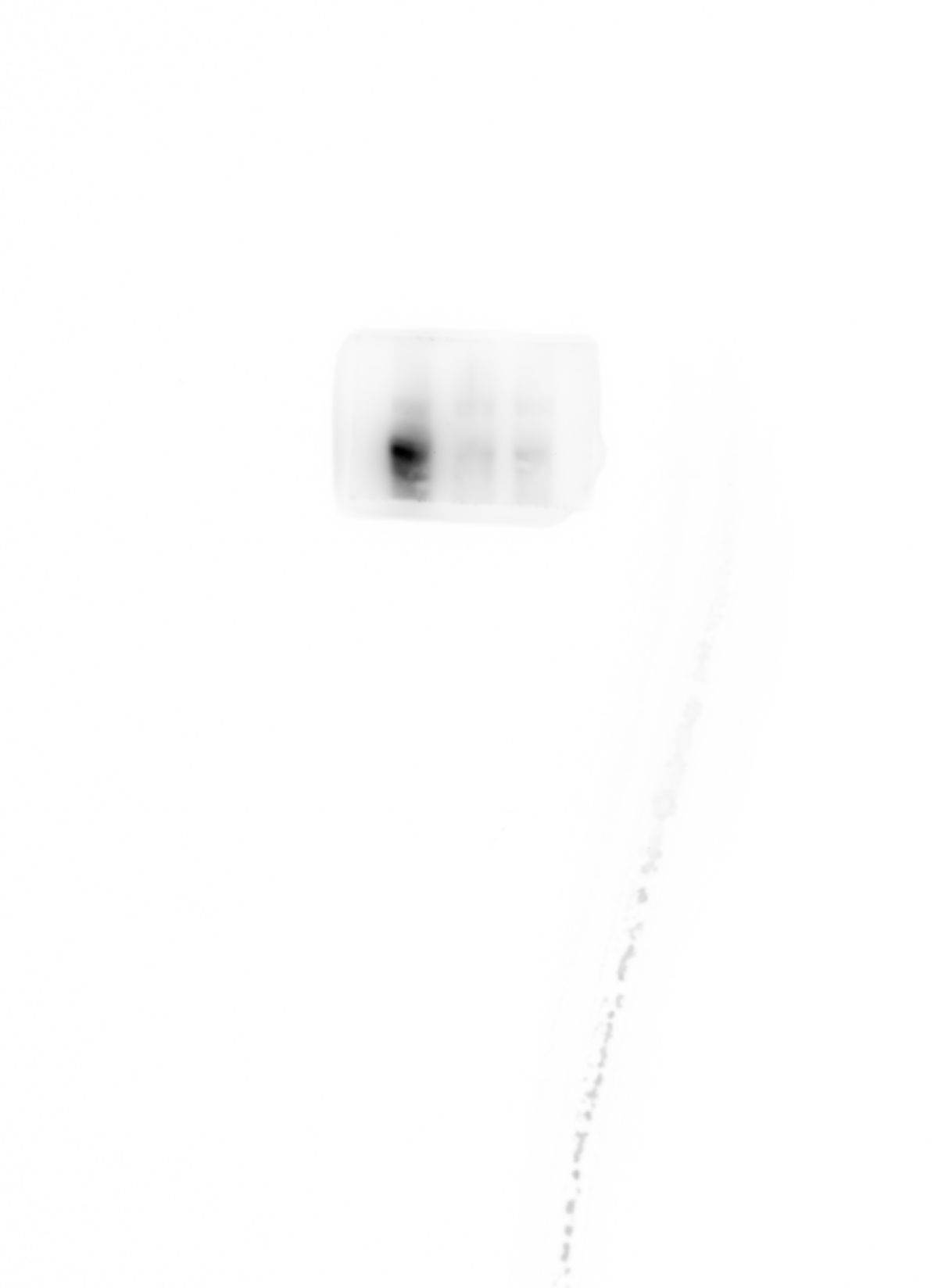

Supplement: Supplementary file 2 — Supplementary Material 2 [file 41598_2025_31281_MOESM2_ESM.zip › Fig6_R1/Fig.6H SGK1.tif]
