# Supplementary material for: Pseudomonas aeruginosa-derived DnaJ functions as a novel immunomodulator inducing IFNβ via CME–SGK1–IRF3 axis in macrophages
Source: Sci Rep. 2025 Dec 3;16:1386. doi: 10.1038/s41598-025-31281-x (PMC12796343; doi:10.1038/s41598-025-31281-x)
Supplement: Supplementary file 3 — Supplementary Material 3 [file 41598_2025_31281_MOESM3_ESM.pptx]

## Slide 1
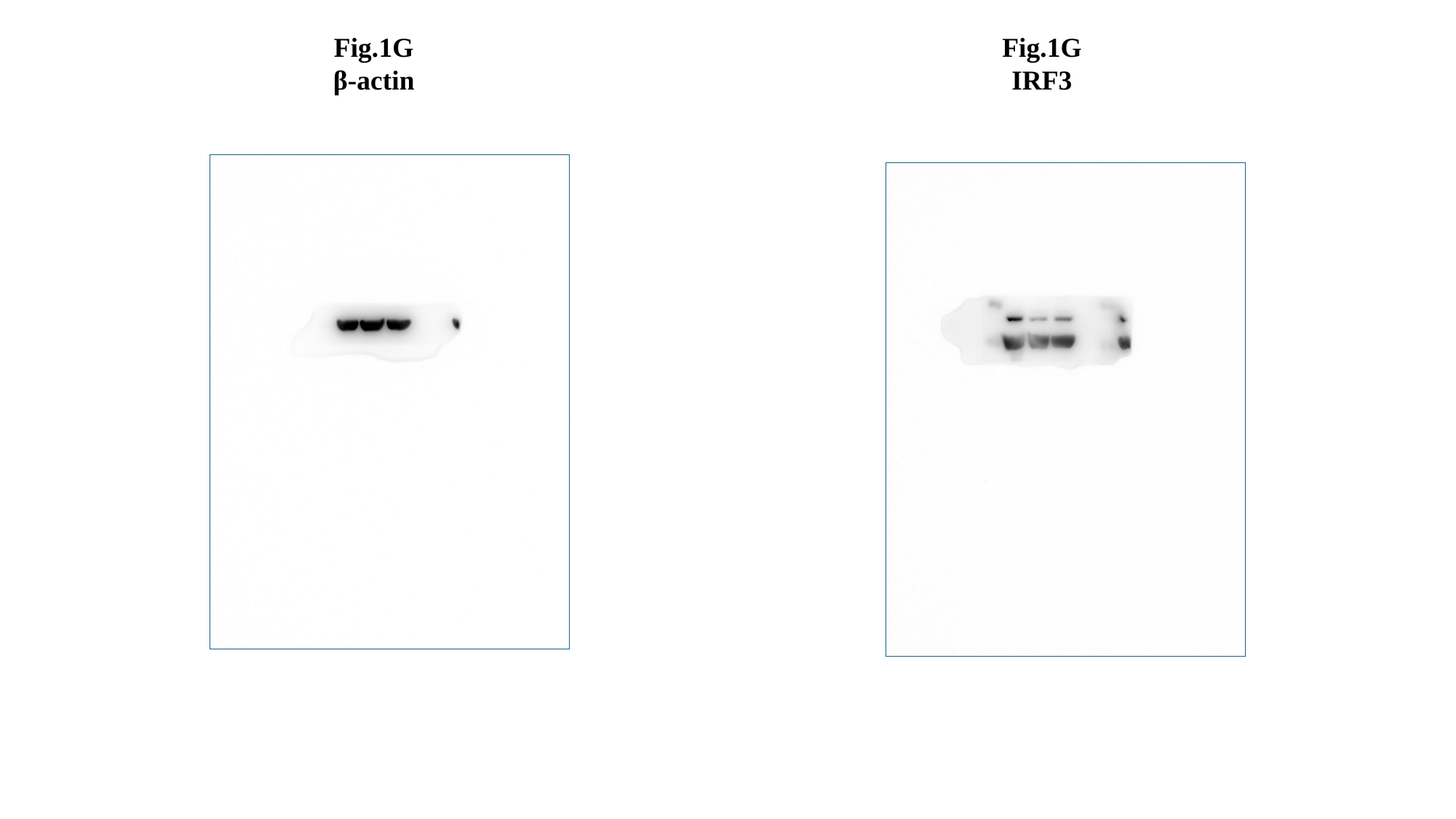

Fig.1G
β-actin
Fig.1G
IRF3

## Slide 2
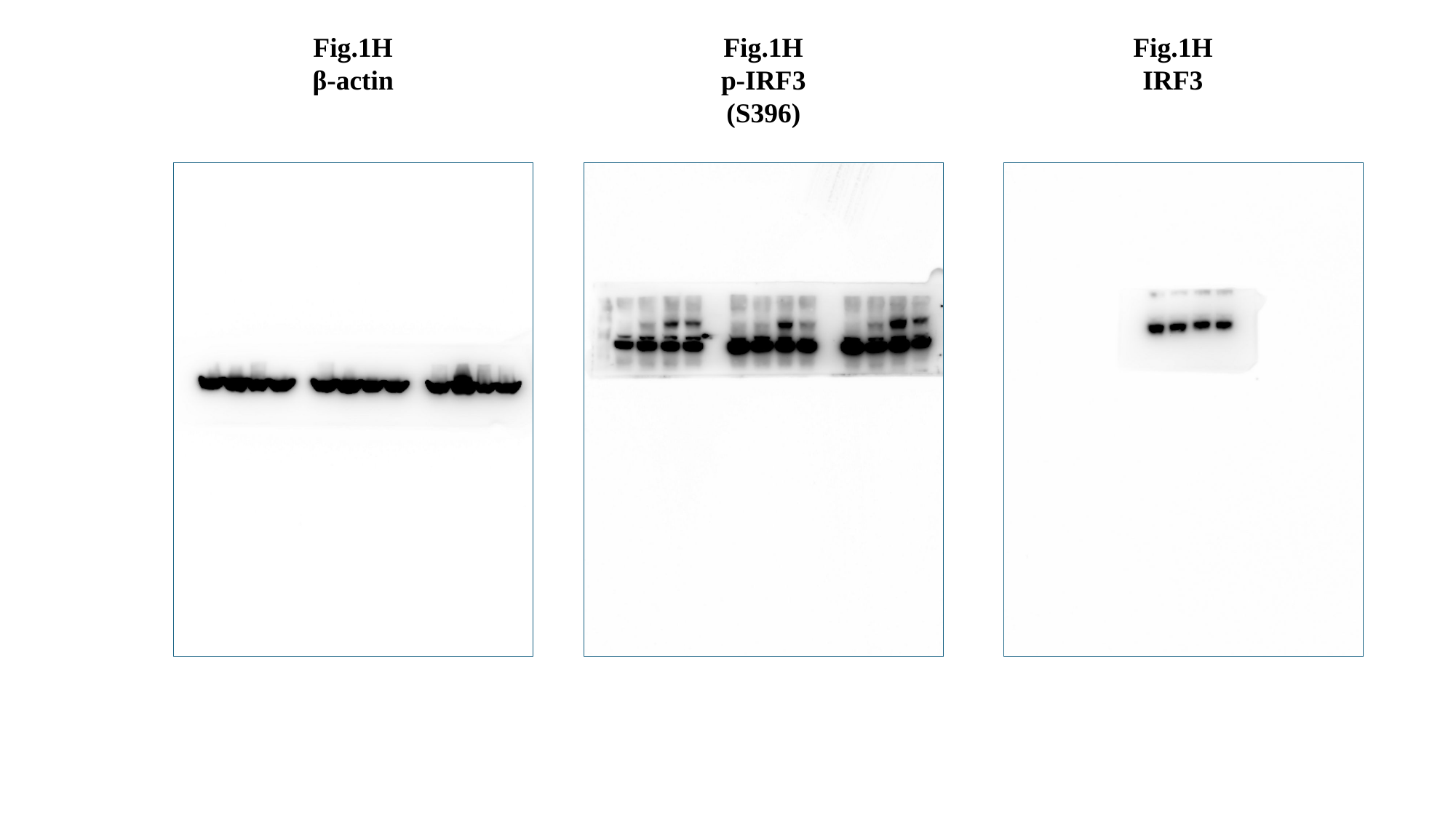

Fig.1H
β-actin
Fig.1H
p-IRF3 (S396)
Fig.1H
IRF3

## Slide 3
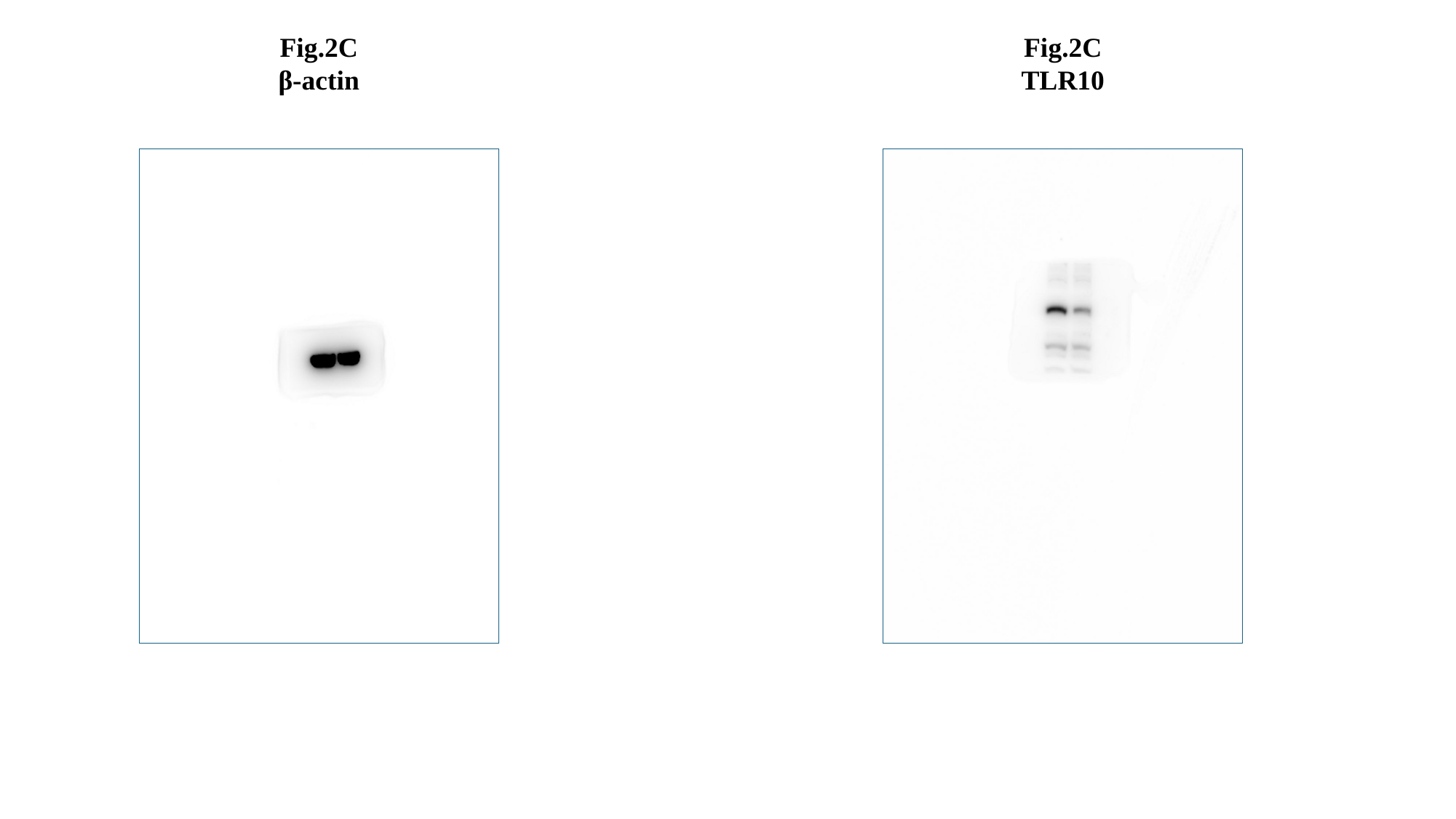

Fig.2C
β-actin
Fig.2C
TLR10

## Slide 4
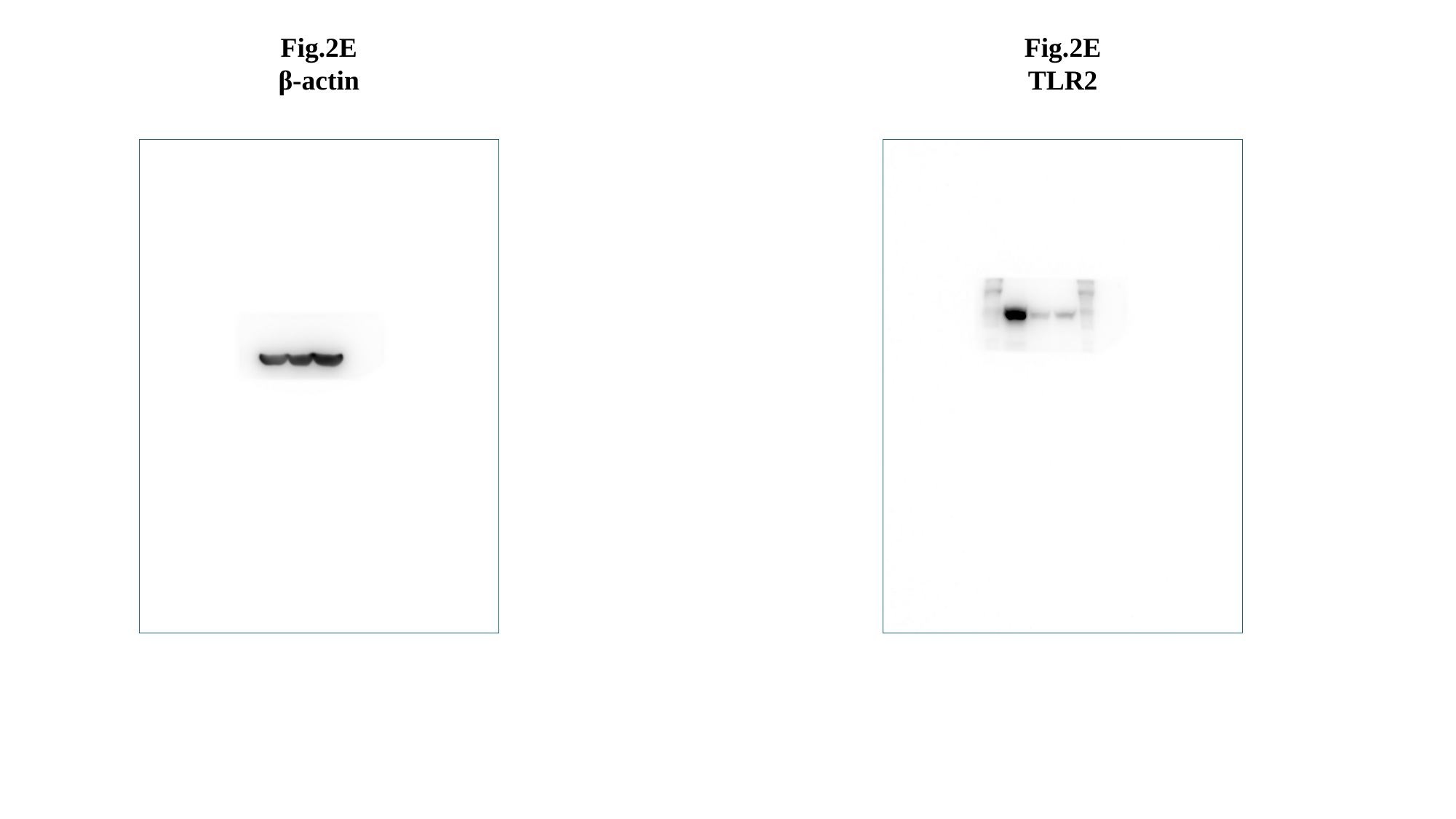

Fig.2E
β-actin
Fig.2E
TLR2

## Slide 5
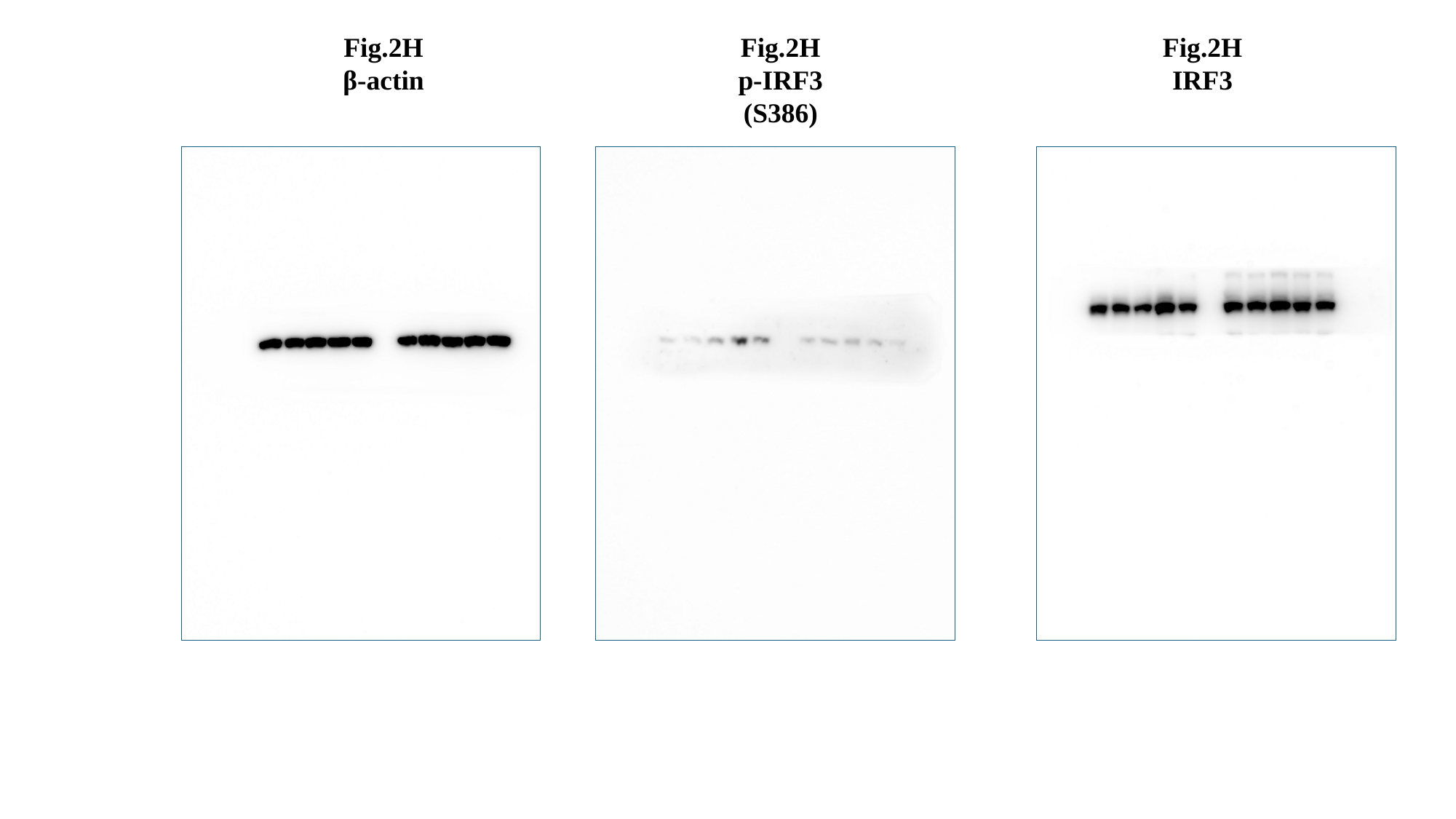

Fig.2H
p-IRF3 (S386)
Fig.2H
IRF3
Fig.2H
β-actin

## Slide 6
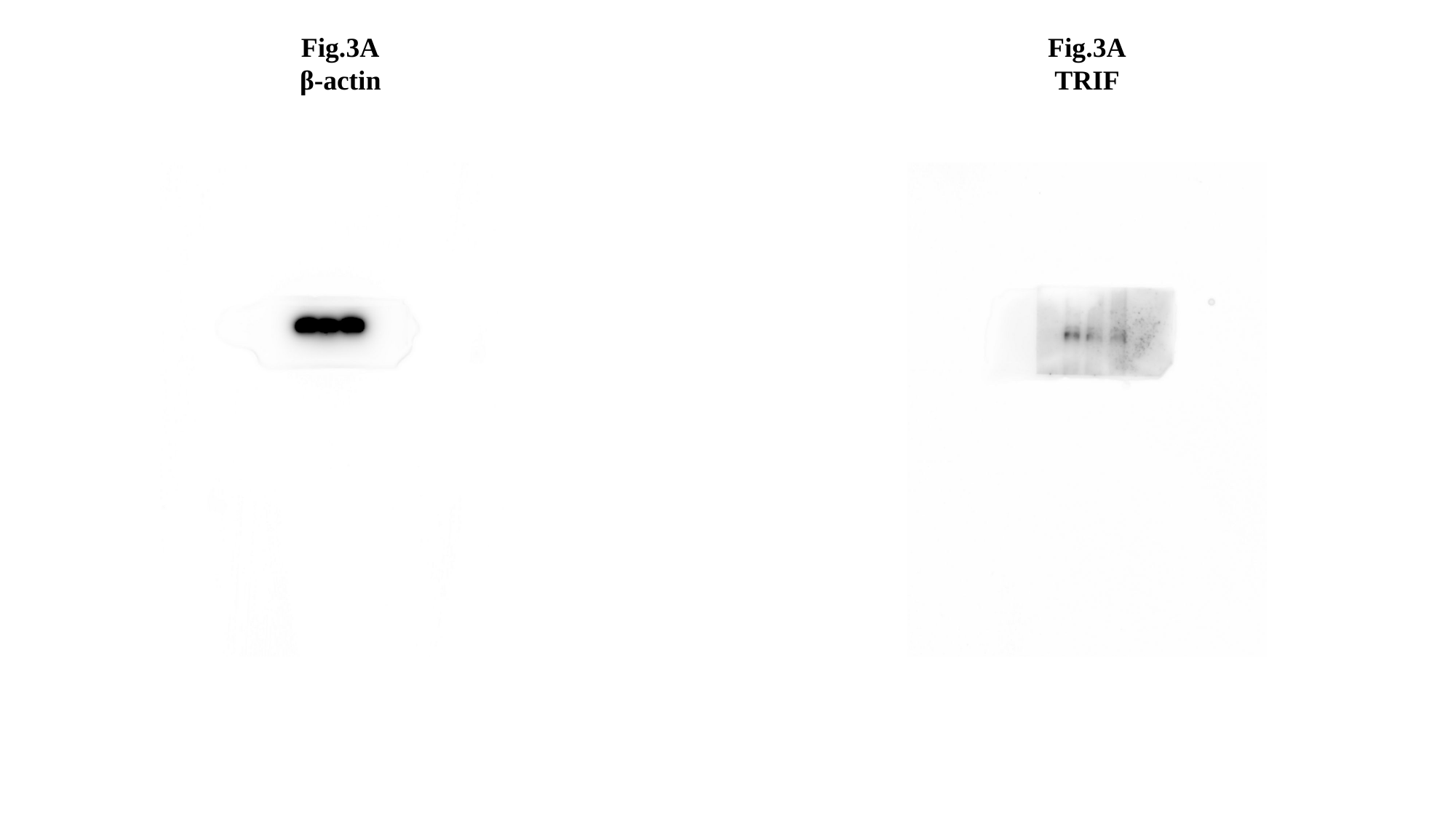

Fig.3A
β-actin
Fig.3A
TRIF

## Slide 7
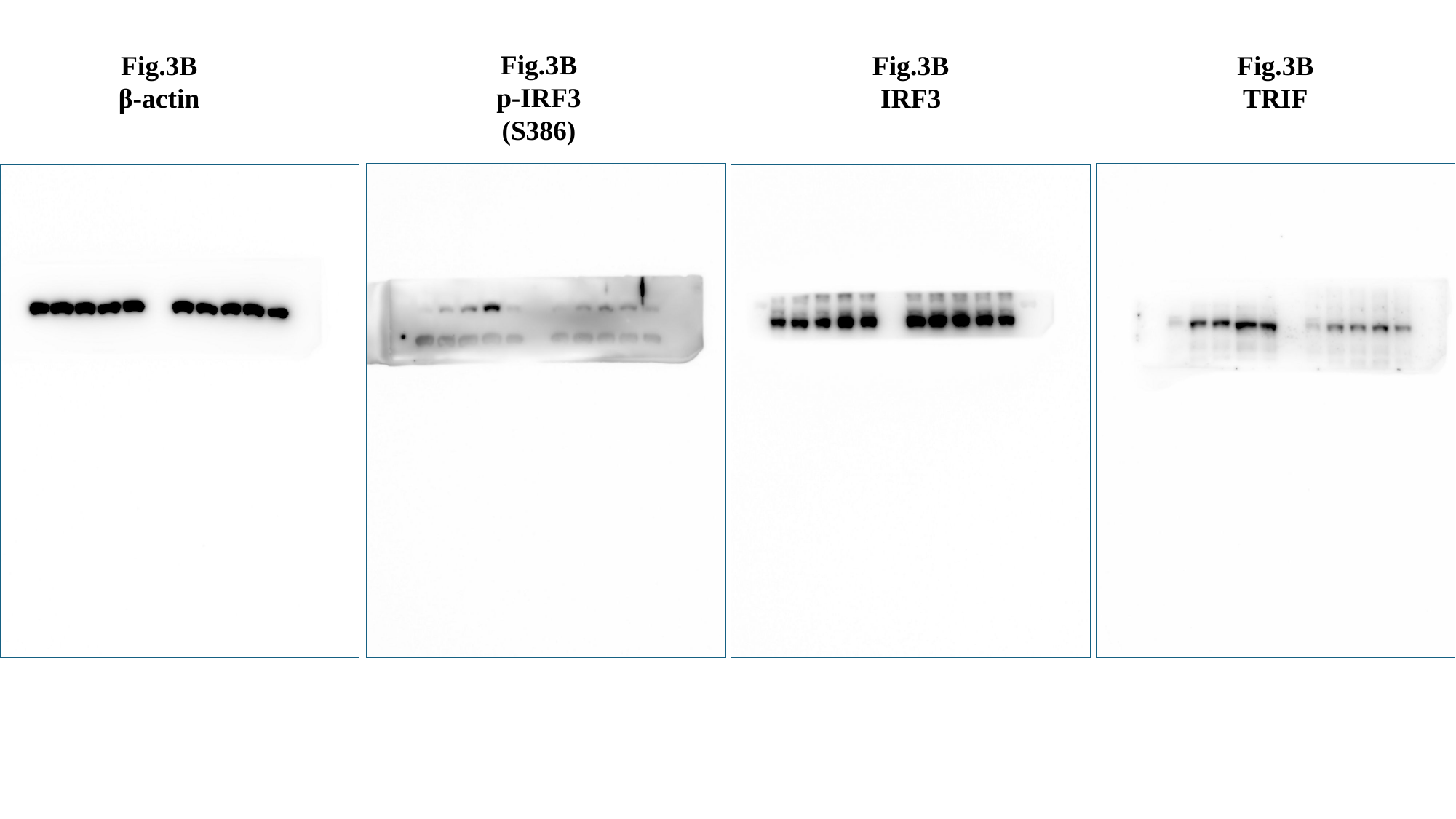

Fig.3B
p-IRF3 (S386)
Fig.3B
β-actin
Fig.3B
TRIF
Fig.3B
IRF3

## Slide 8
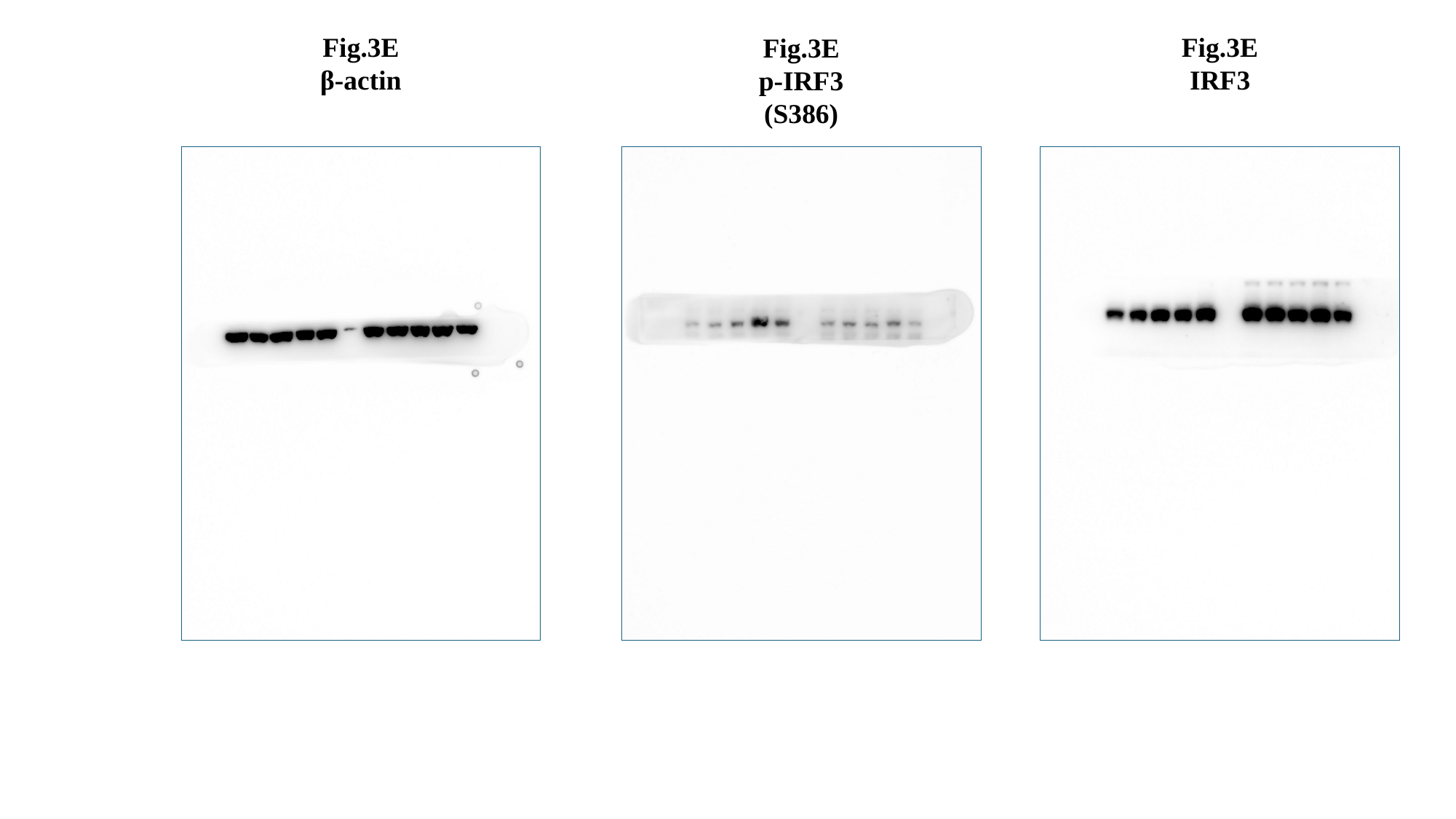

Fig.3E
β-actin
Fig.3E
IRF3
Fig.3E
p-IRF3 (S386)

## Slide 9
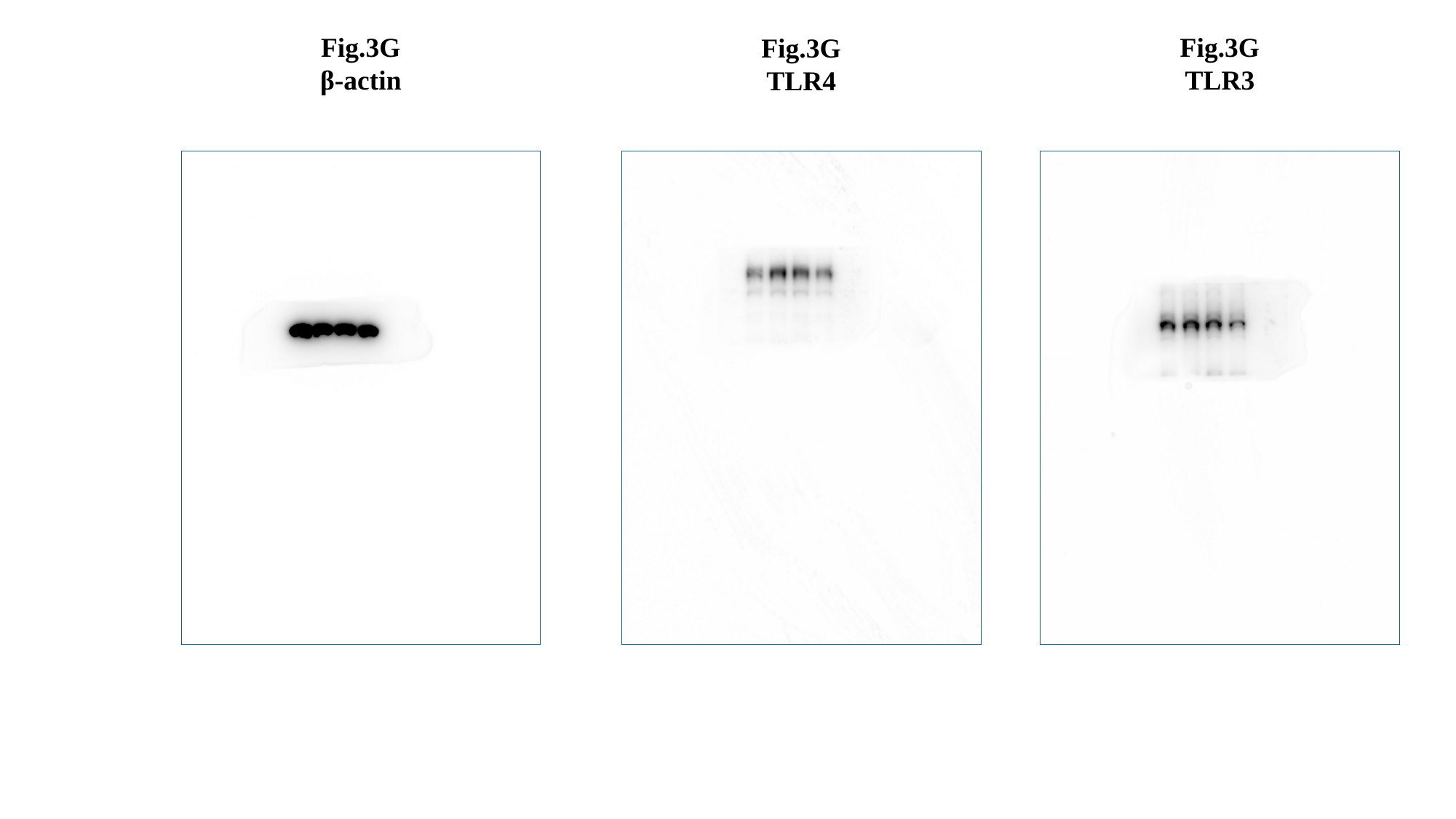

Fig.3G
β-actin
Fig.3G
TLR3
Fig.3G
TLR4

## Slide 10
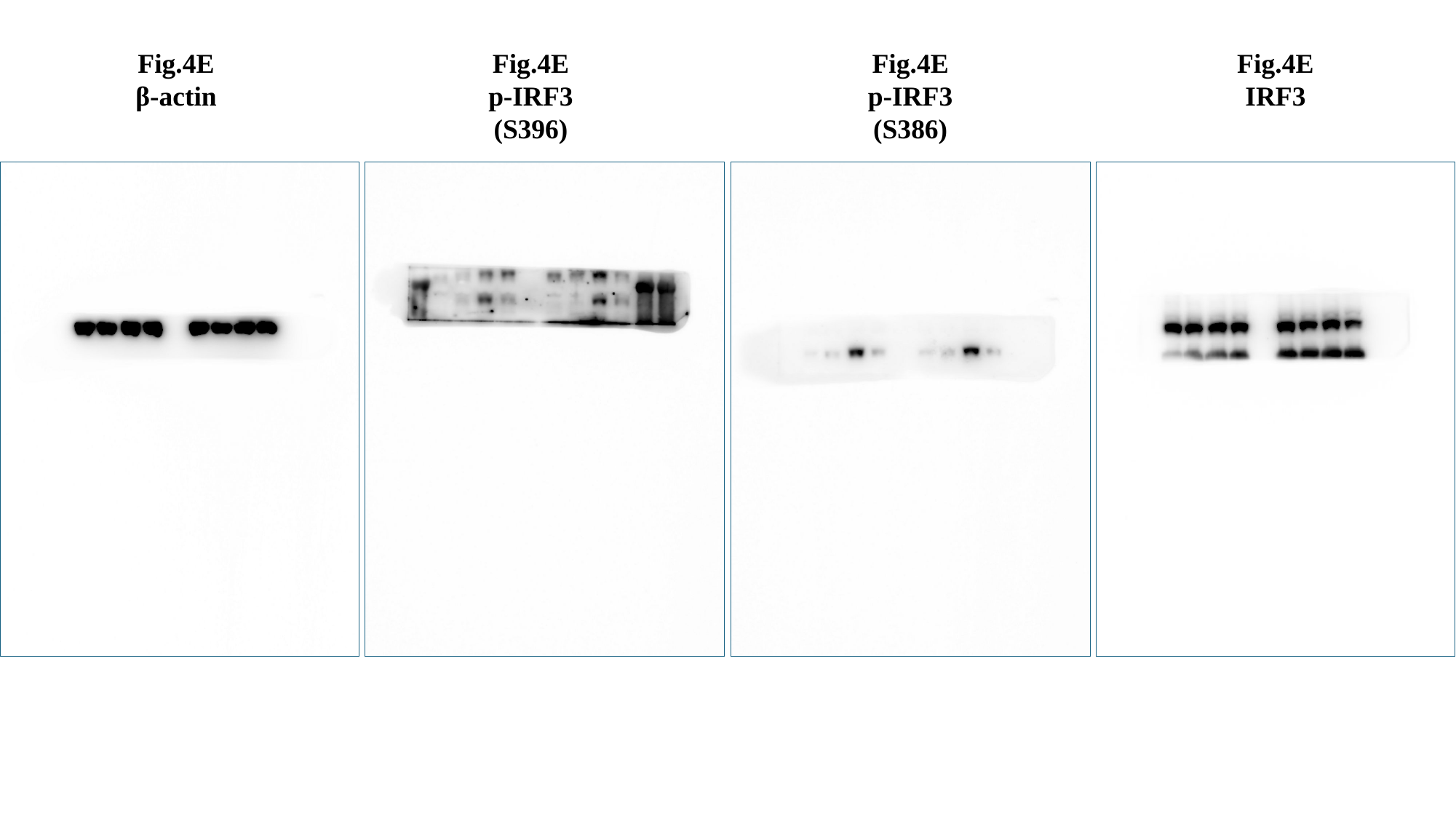

Fig.4E
IRF3
Fig.4E
β-actin
Fig.4E
p-IRF3 (S396)
Fig.4E
p-IRF3 (S386)

## Slide 11
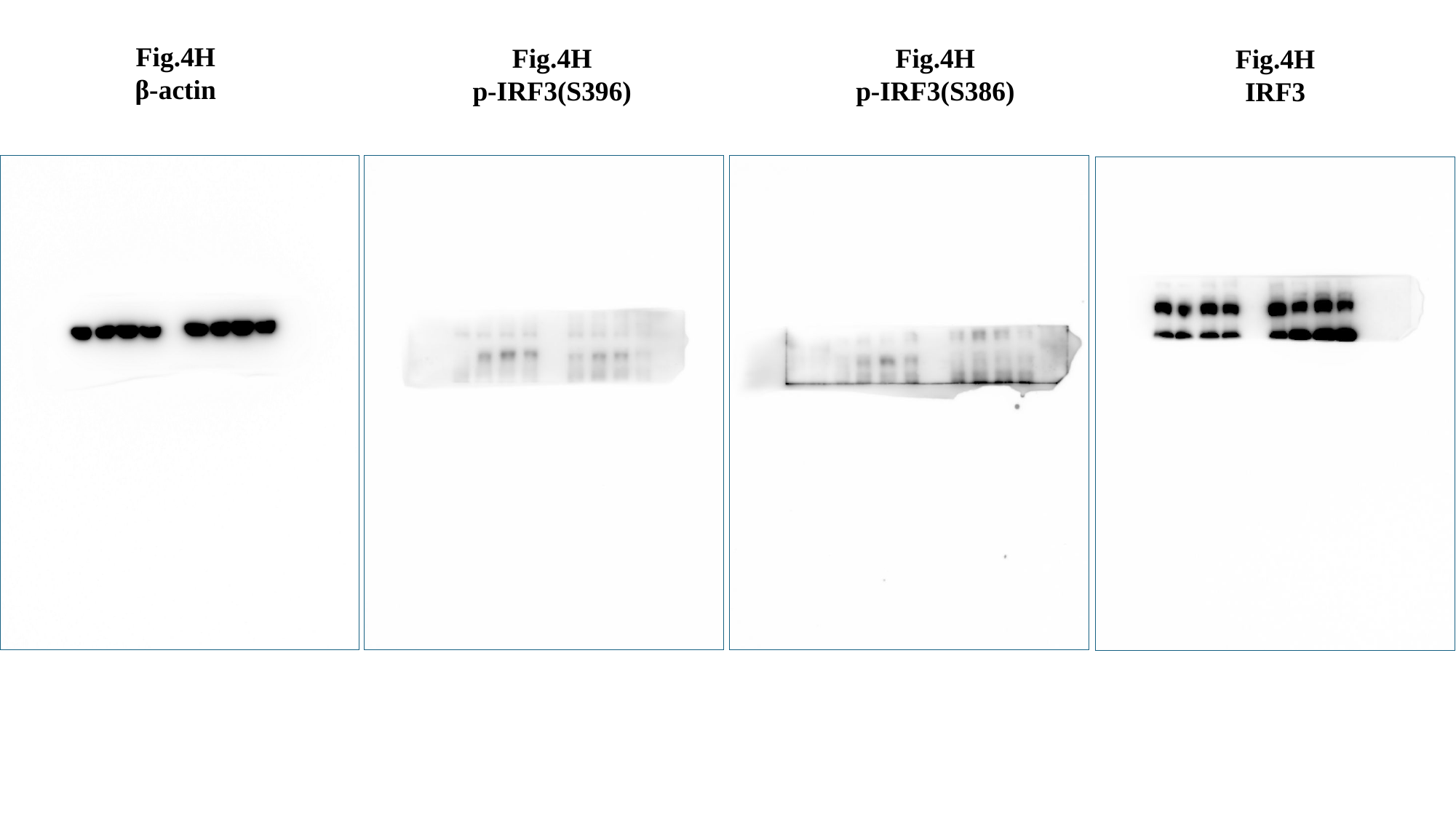

Fig.4H
β-actin
Fig.4H
p-IRF3(S396)
Fig.4H
p-IRF3(S386)
Fig.4H
IRF3

## Slide 12
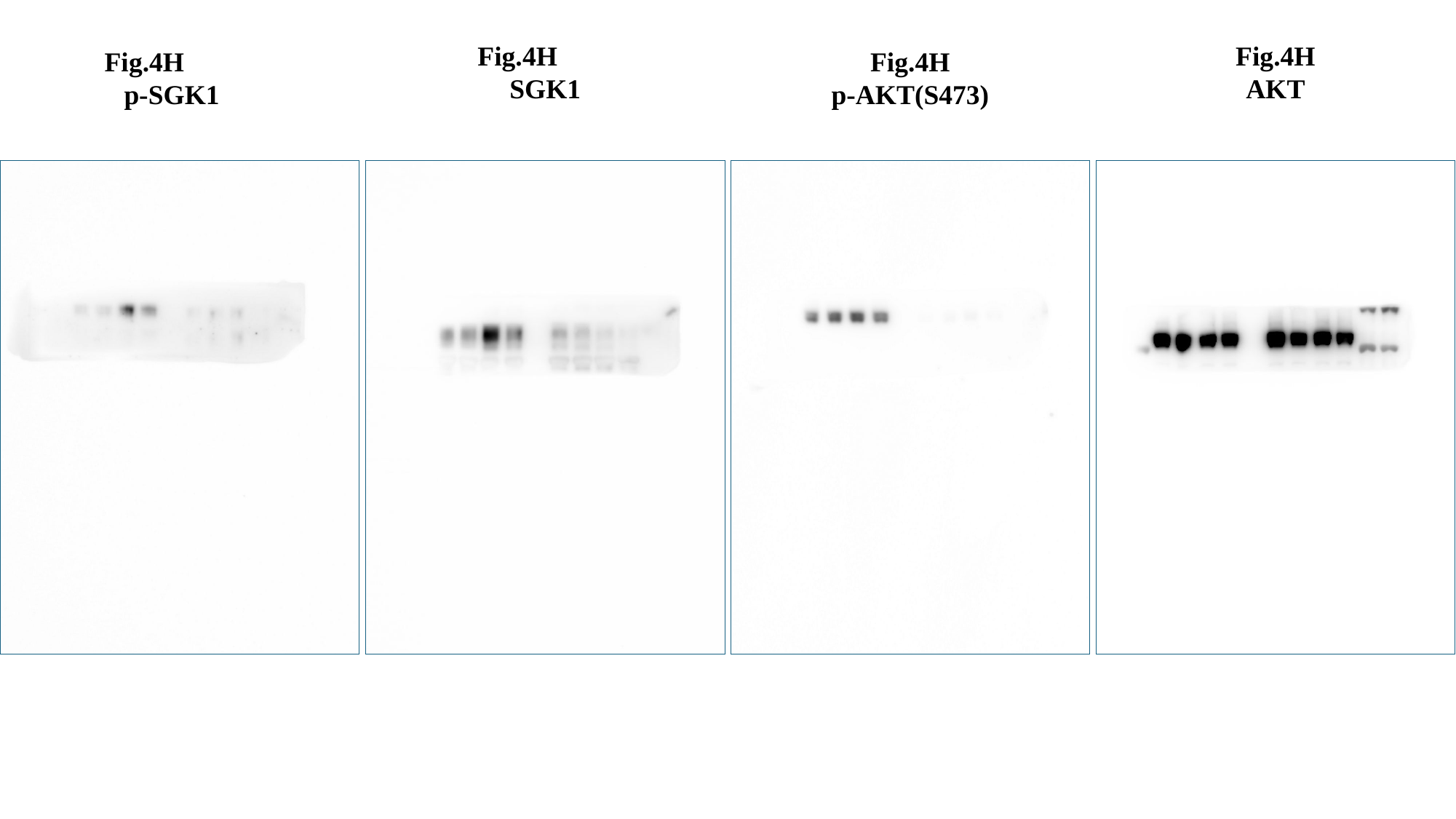

Fig.4H SGK1
Fig.4H
AKT
Fig.4H p-SGK1
Fig.4H
p-AKT(S473)

## Slide 13
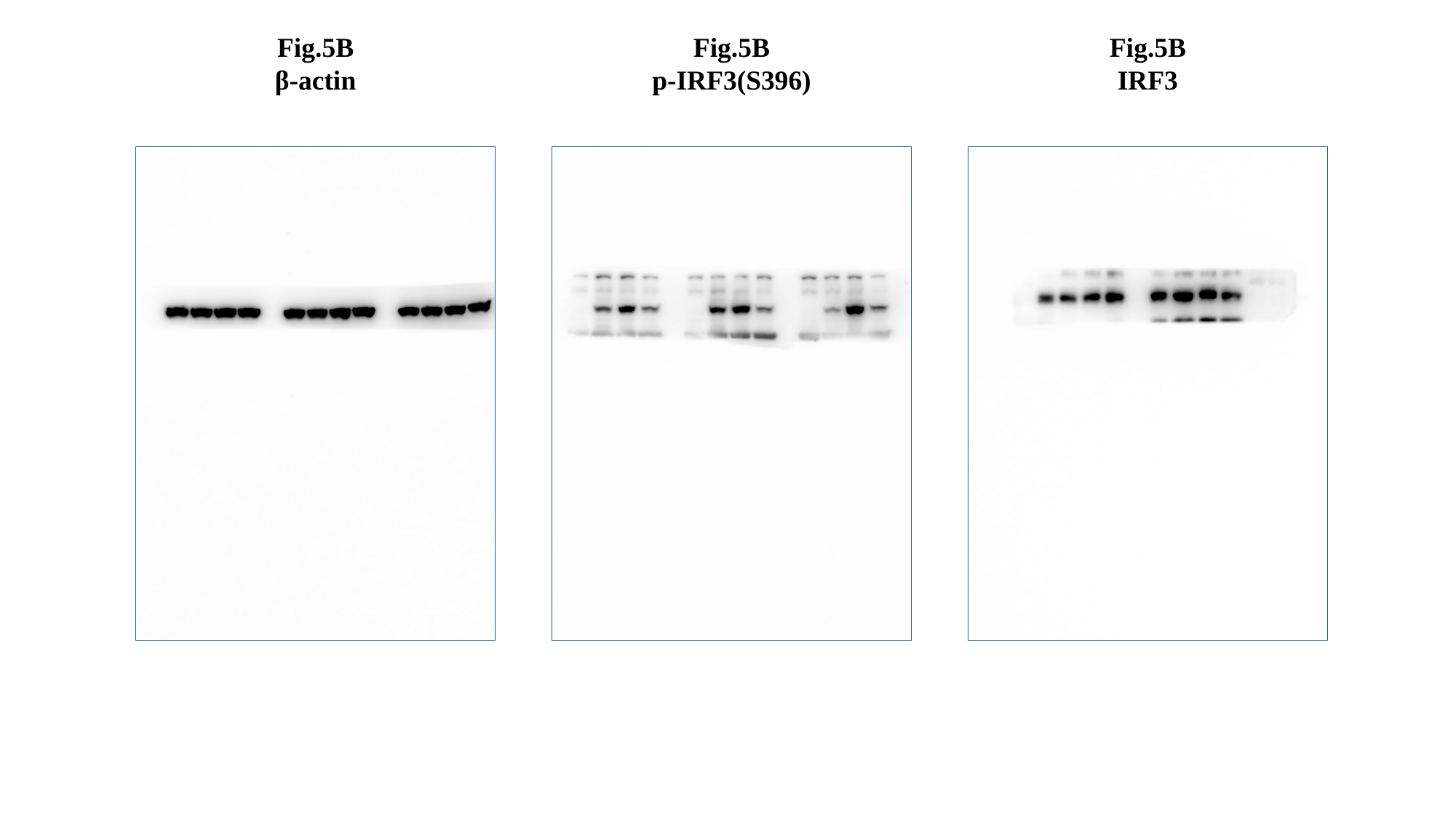

Fig.5B
β-actin
Fig.5B
p-IRF3(S396)
Fig.5B
IRF3

## Slide 14
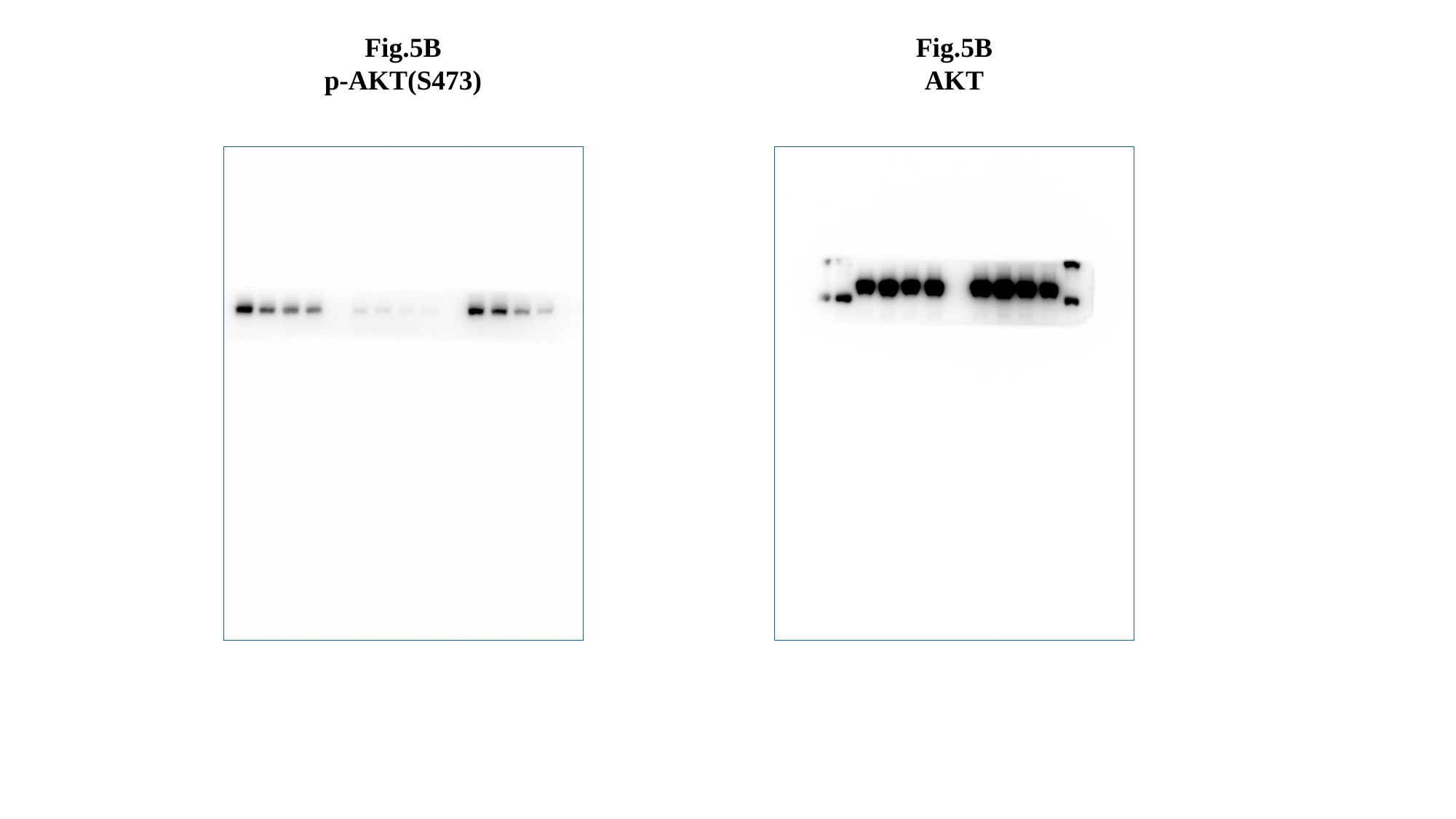

Fig.5B
p-AKT(S473)
Fig.5B
AKT

## Slide 15
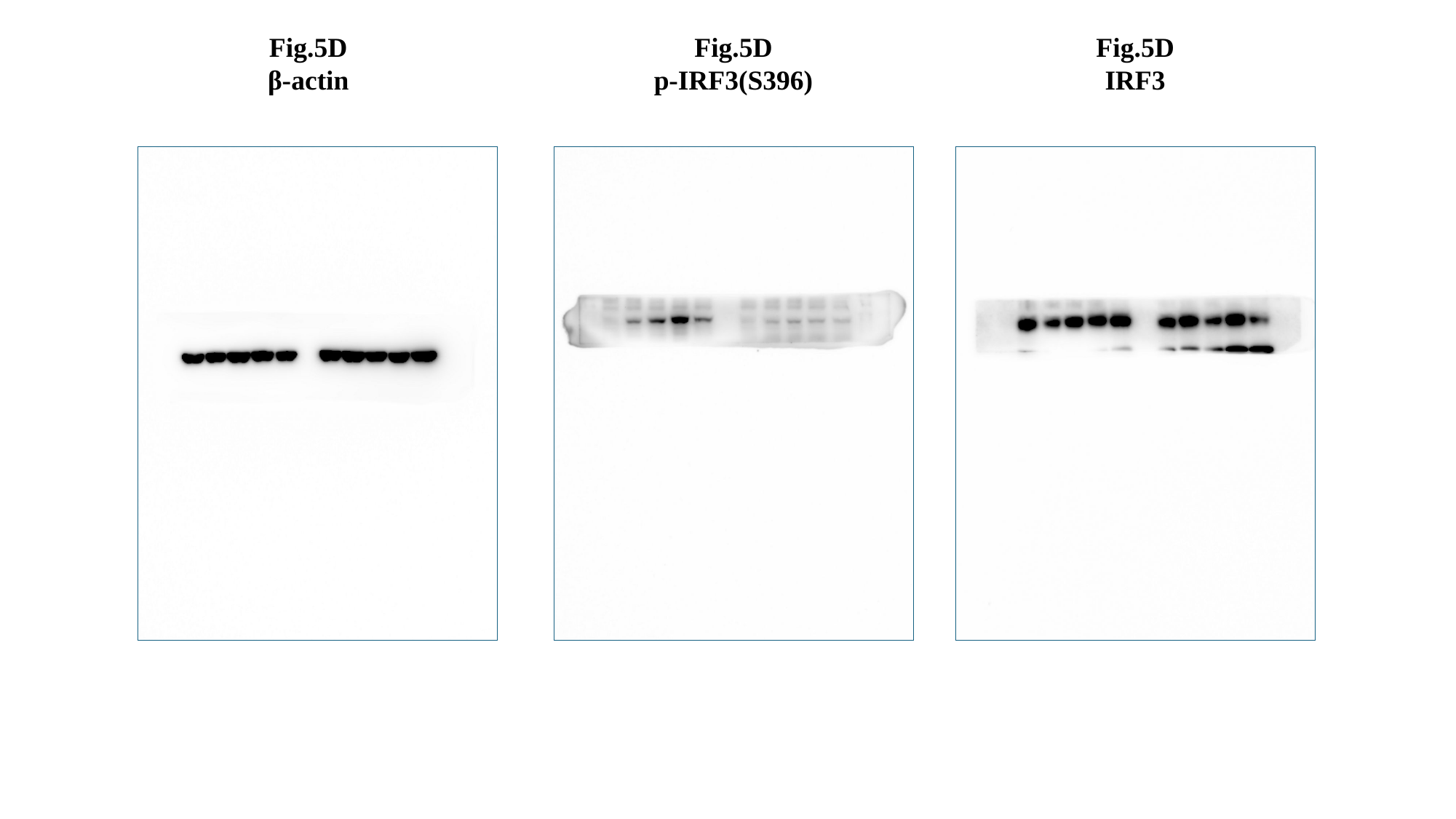

Fig.5D
β-actin
Fig.5D
p-IRF3(S396)
Fig.5D
IRF3

## Slide 16
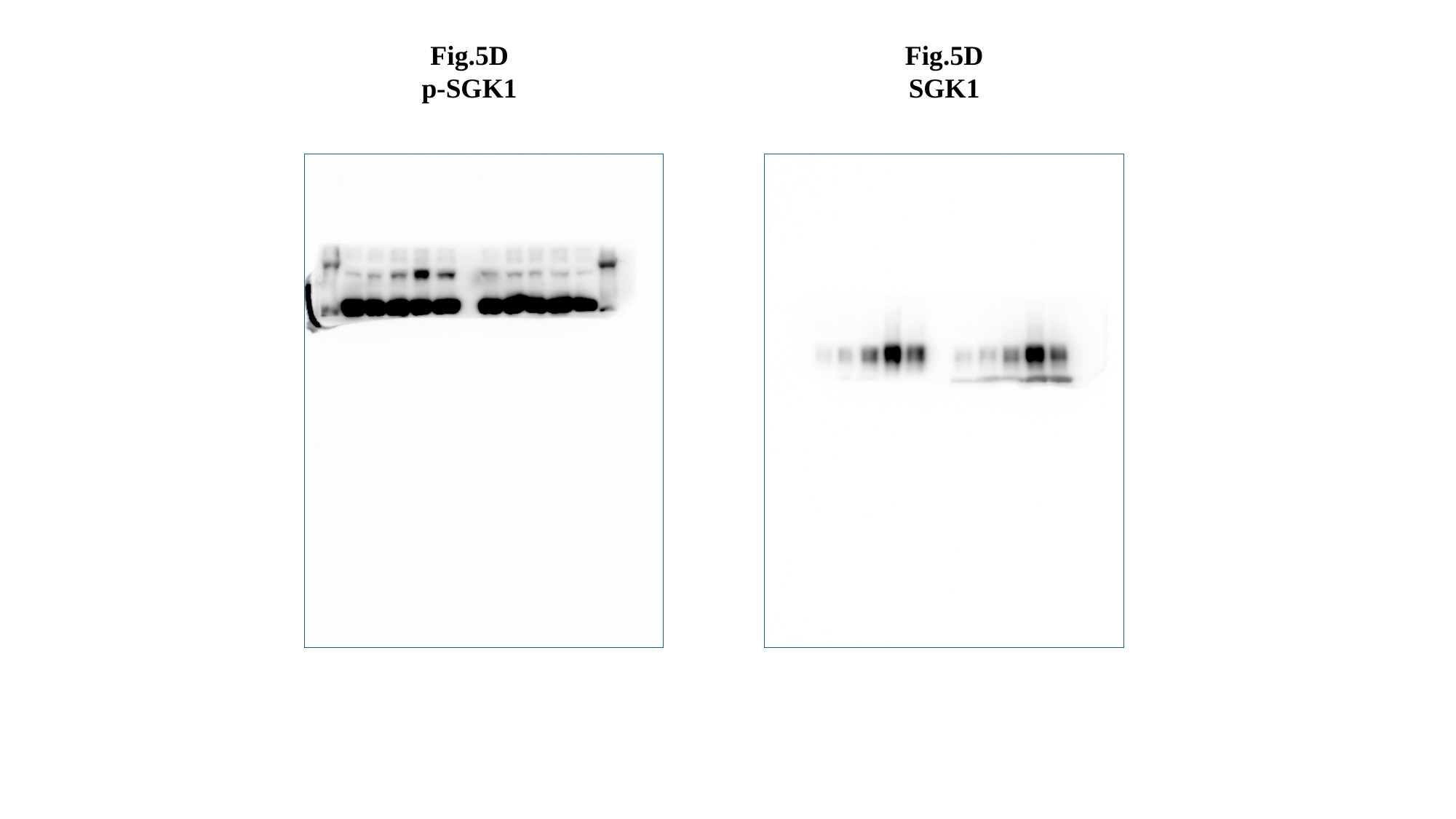

Fig.5D
p-SGK1
Fig.5D
SGK1

## Slide 17
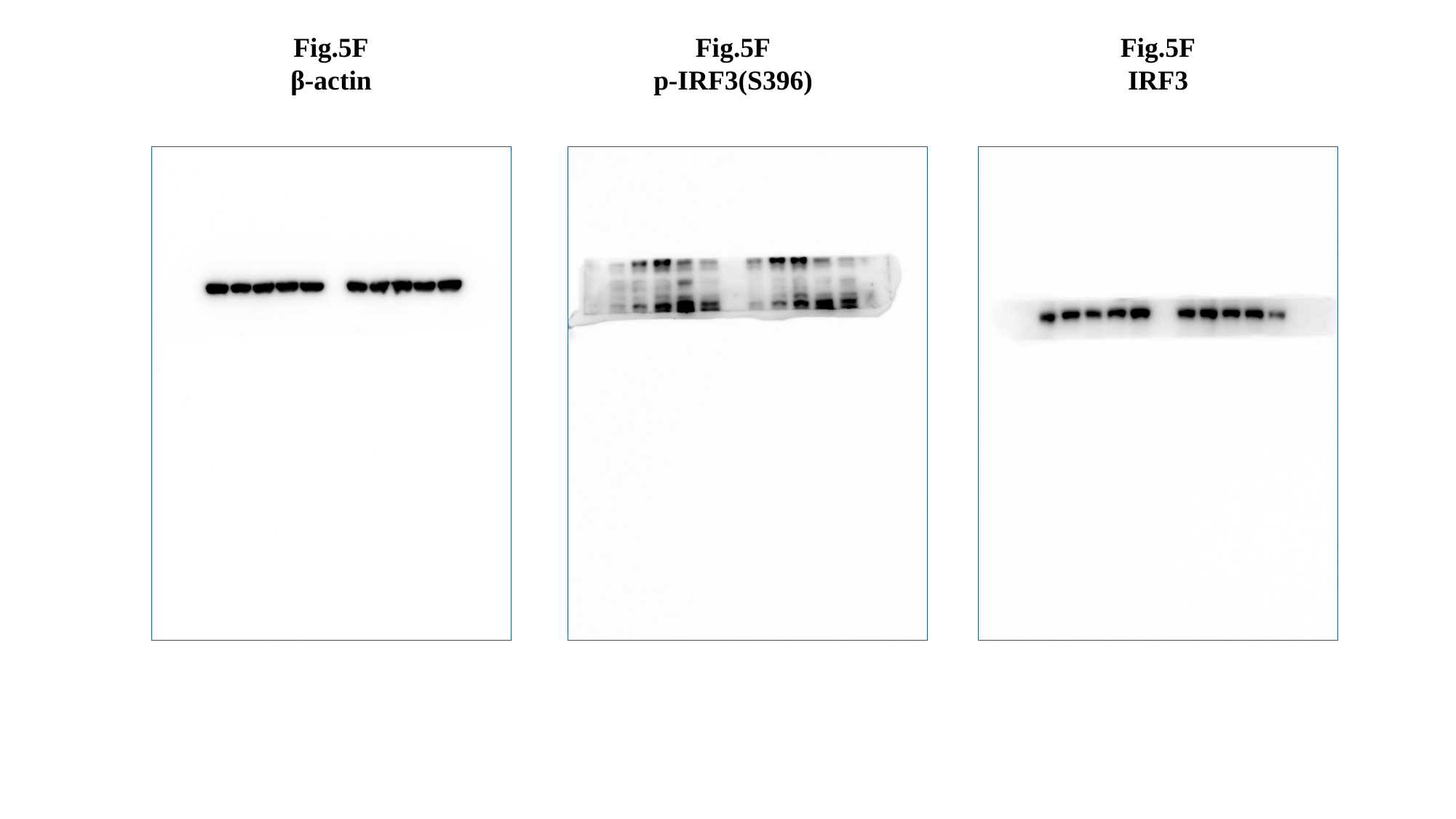

Fig.5F
β-actin
Fig.5F
p-IRF3(S396)
Fig.5F
IRF3

## Slide 18
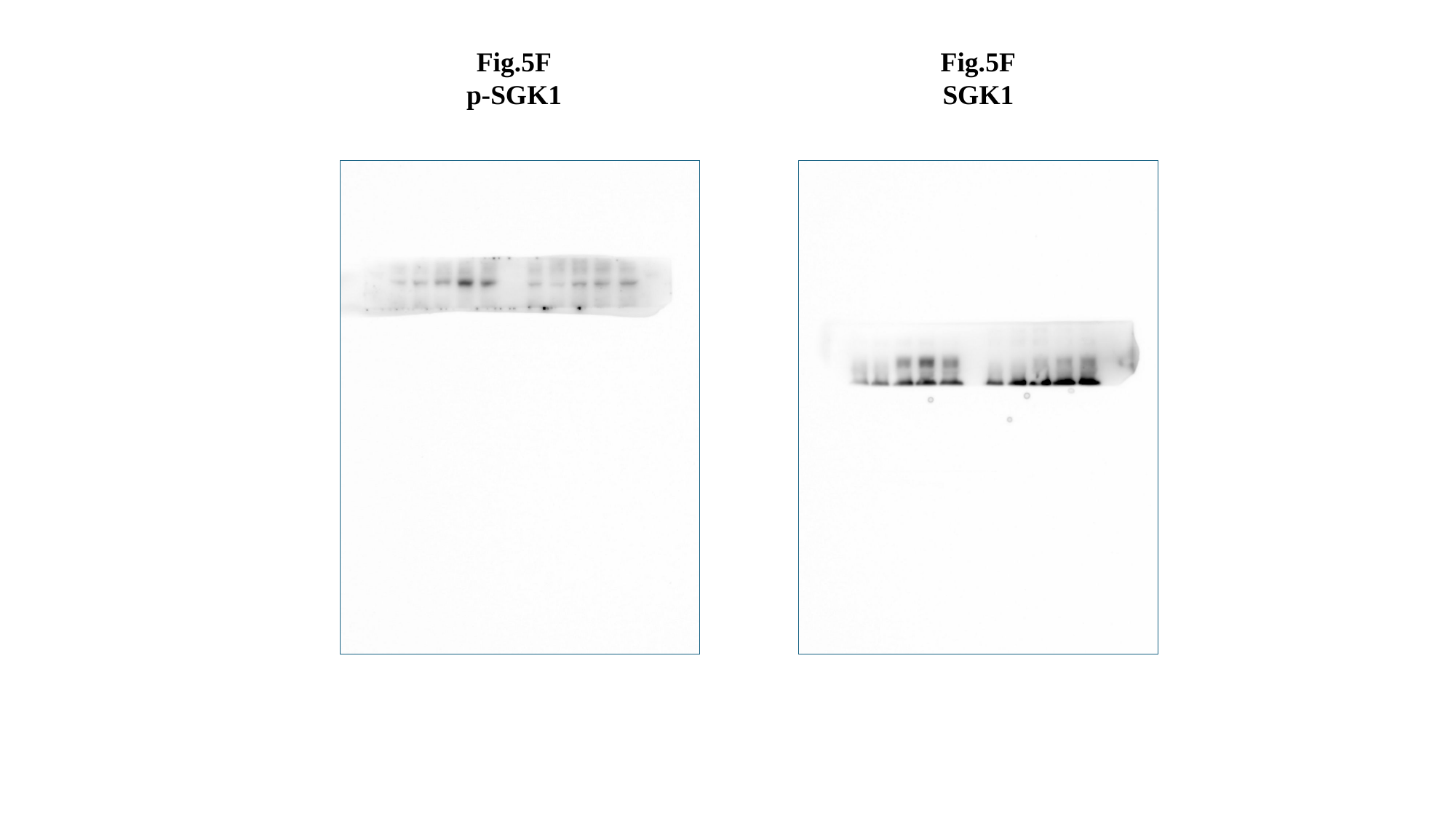

Fig.5F
p-SGK1
Fig.5F
SGK1

## Slide 19
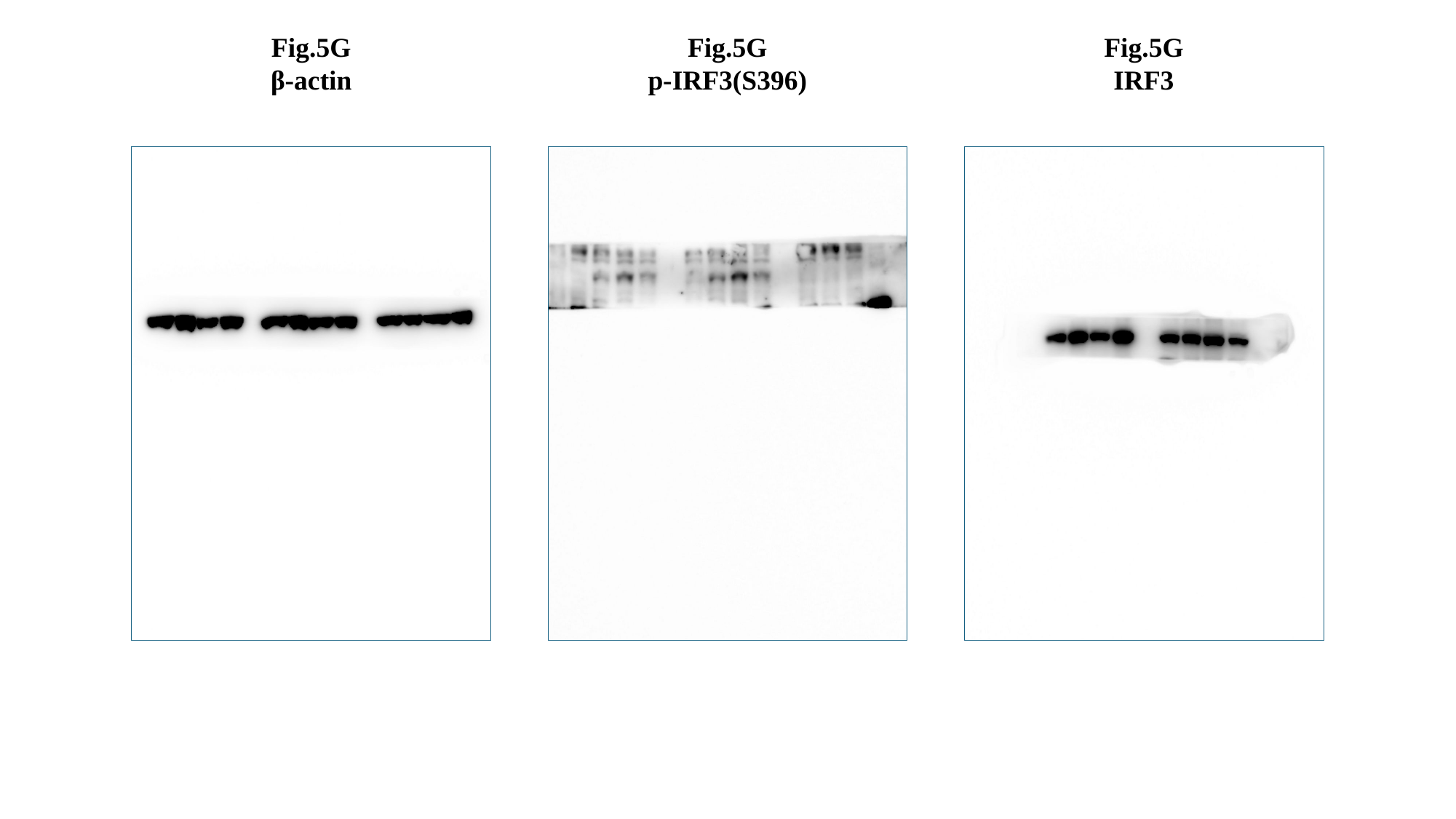

Fig.5G
β-actin
Fig.5G
p-IRF3(S396)
Fig.5G
IRF3

## Slide 20
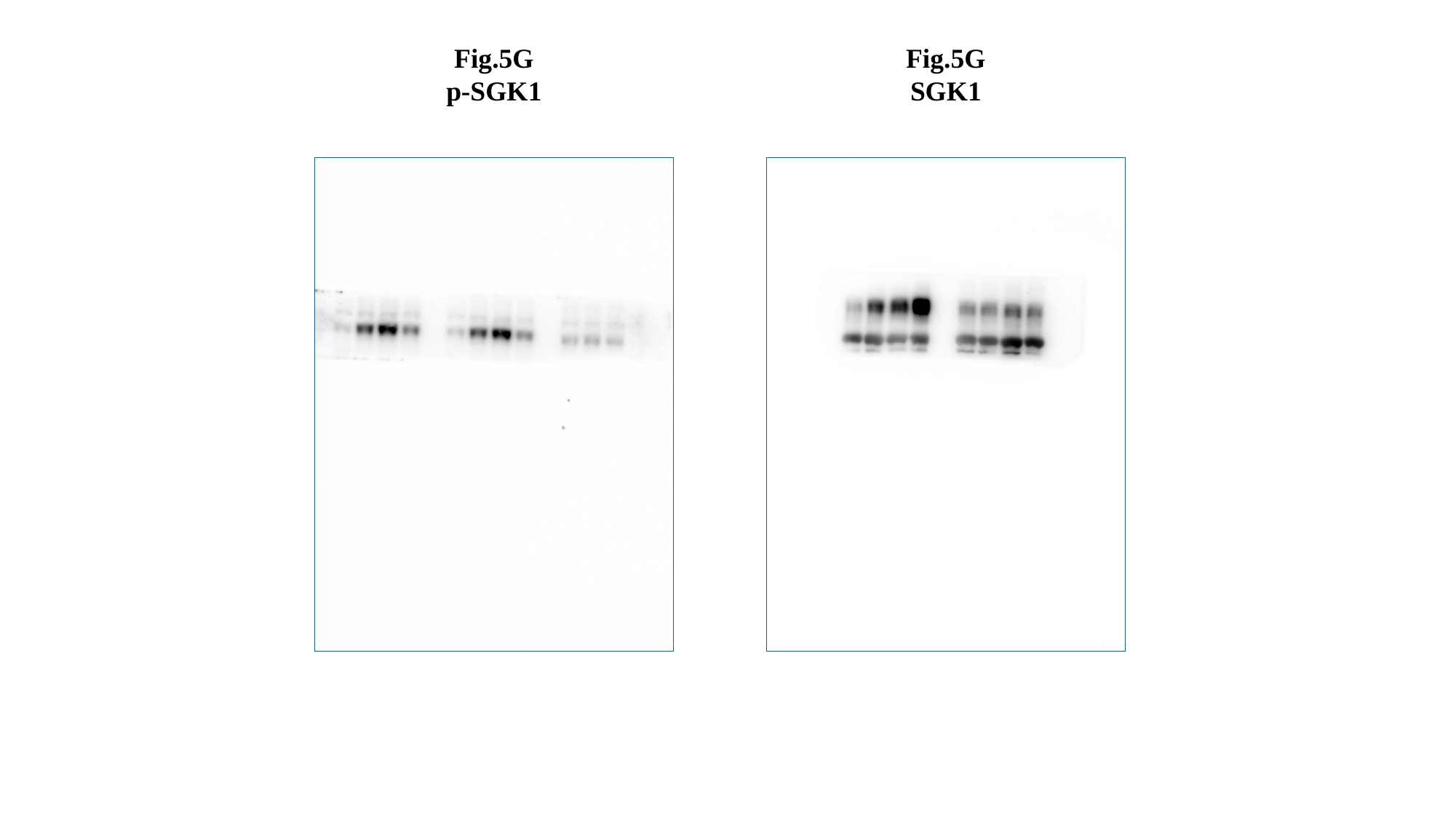

Fig.5G
p-SGK1
Fig.5G
SGK1

## Slide 21
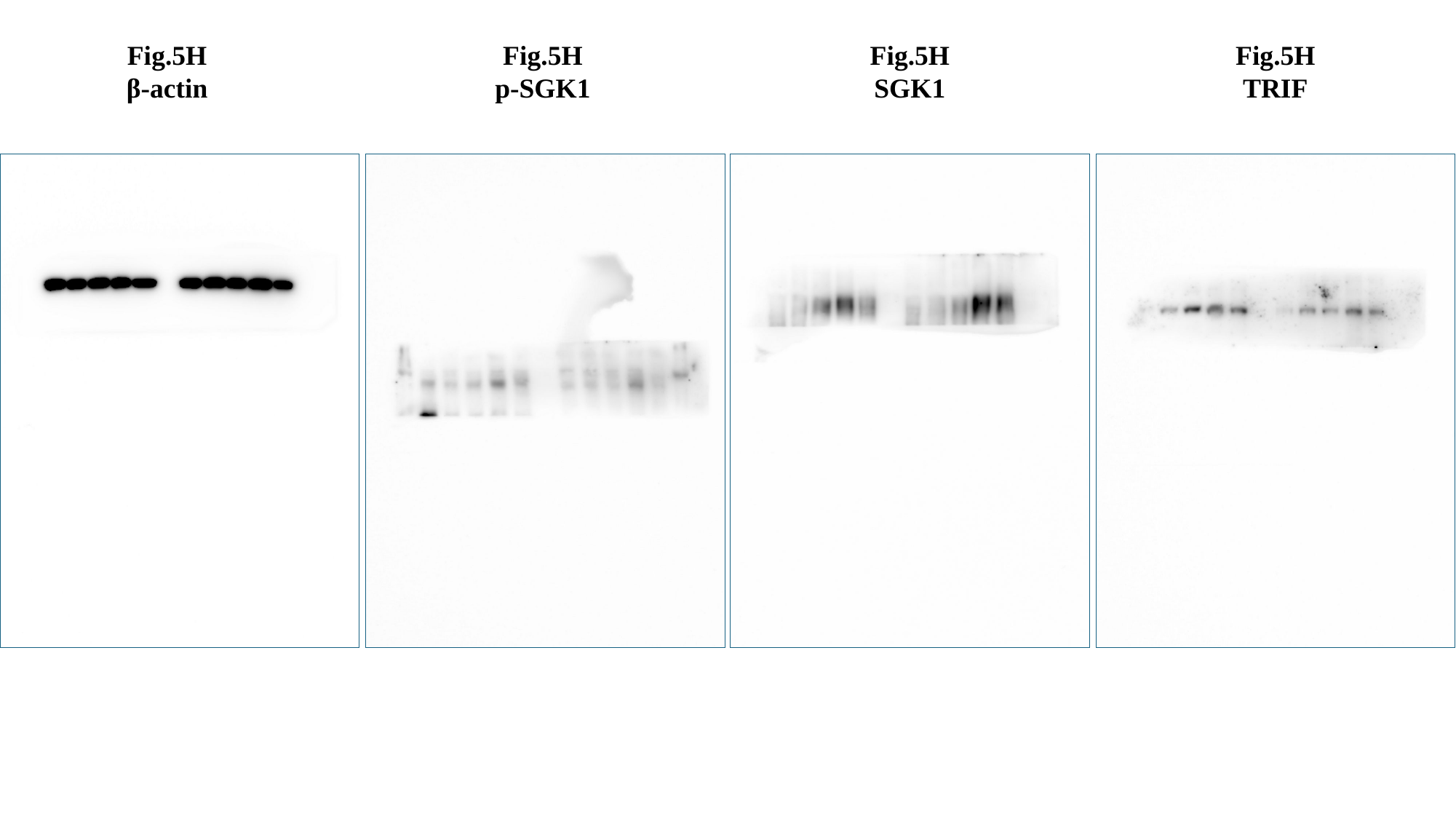

Fig.5H
β-actin
Fig.5H
p-SGK1
Fig.5H
SGK1
Fig.5H
TRIF

## Slide 22
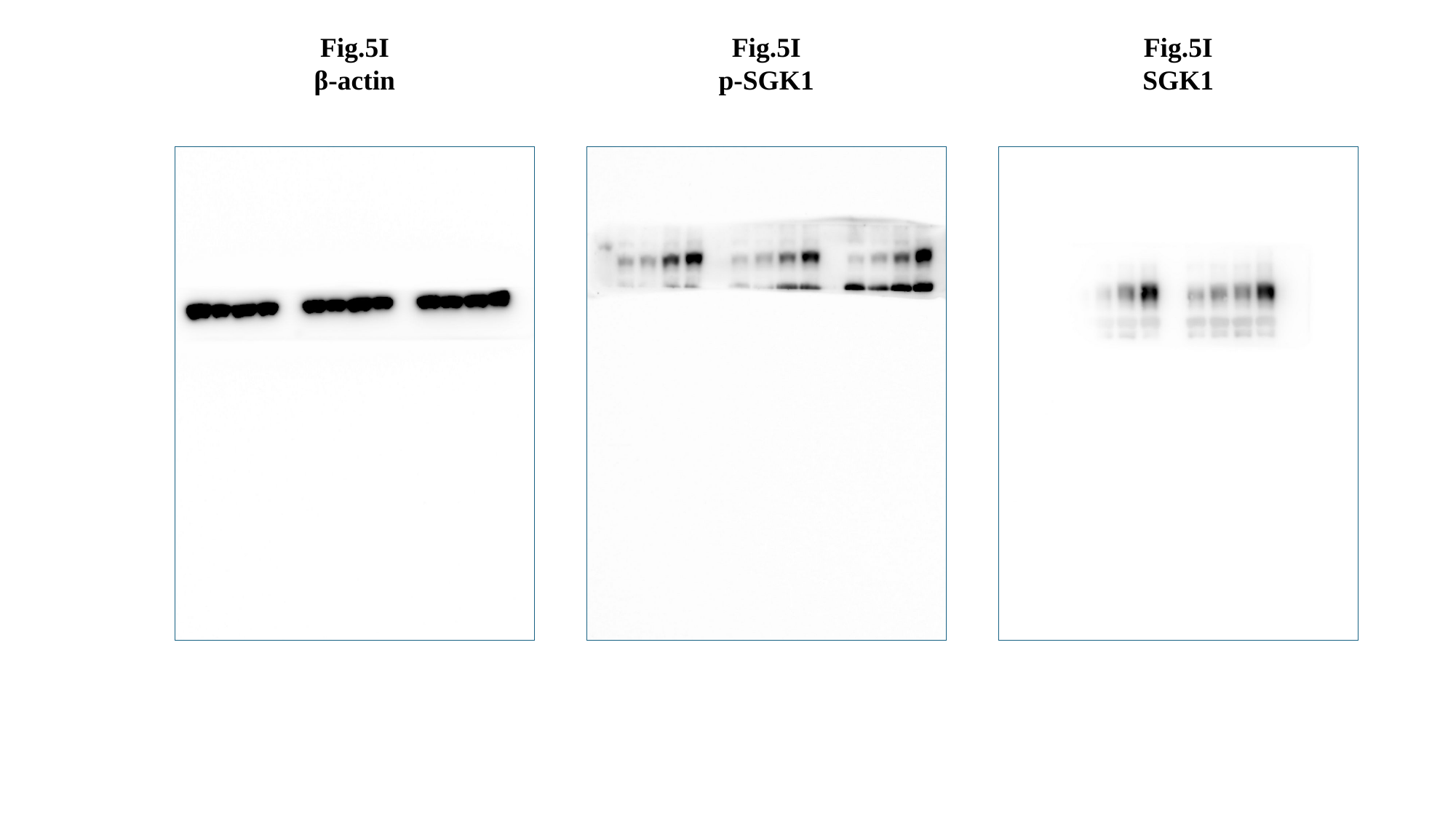

Fig.5I
β-actin
Fig.5I
p-SGK1
Fig.5I
SGK1

## Slide 23
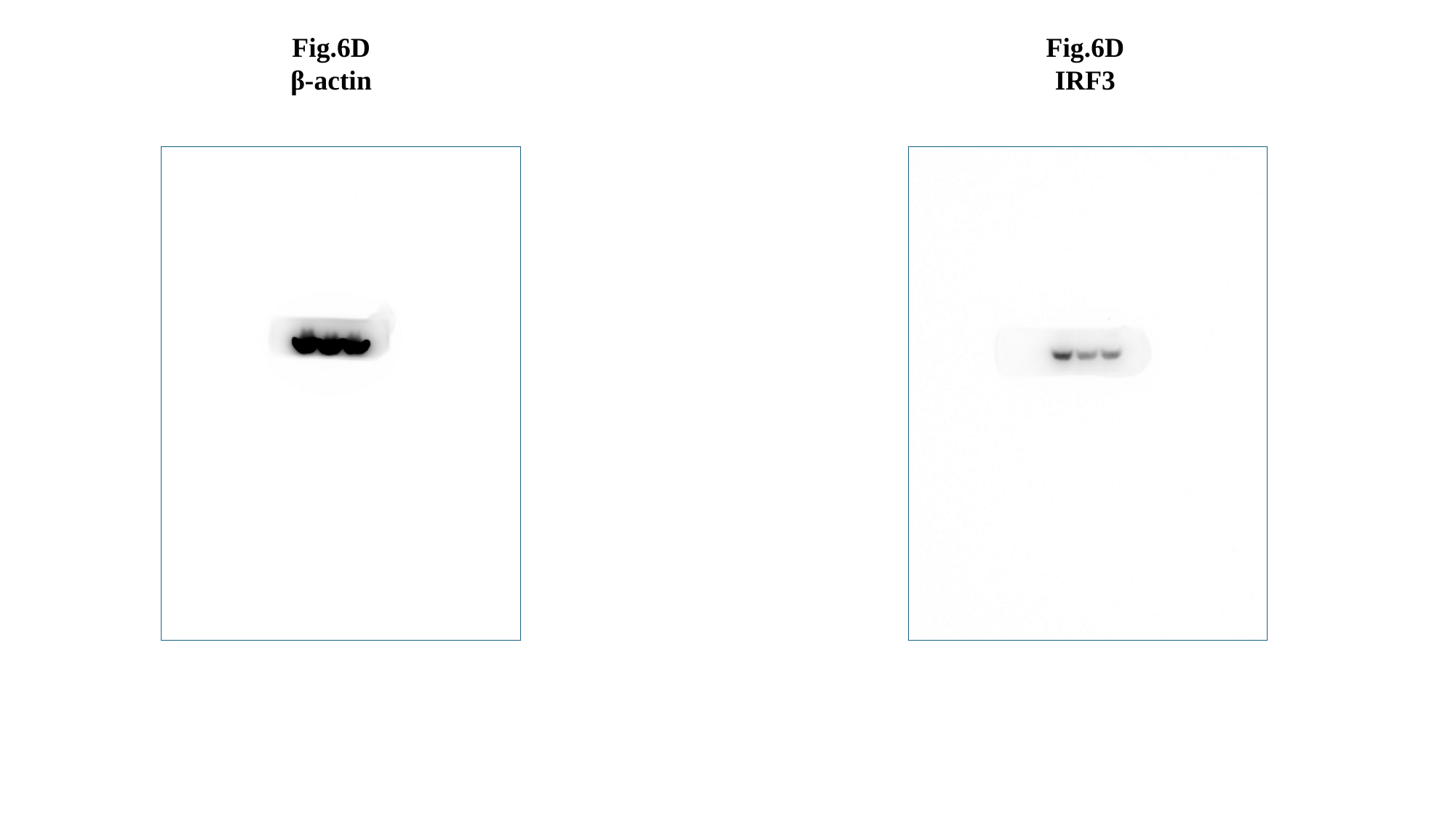

Fig.6D
β-actin
Fig.6D
IRF3

## Slide 24
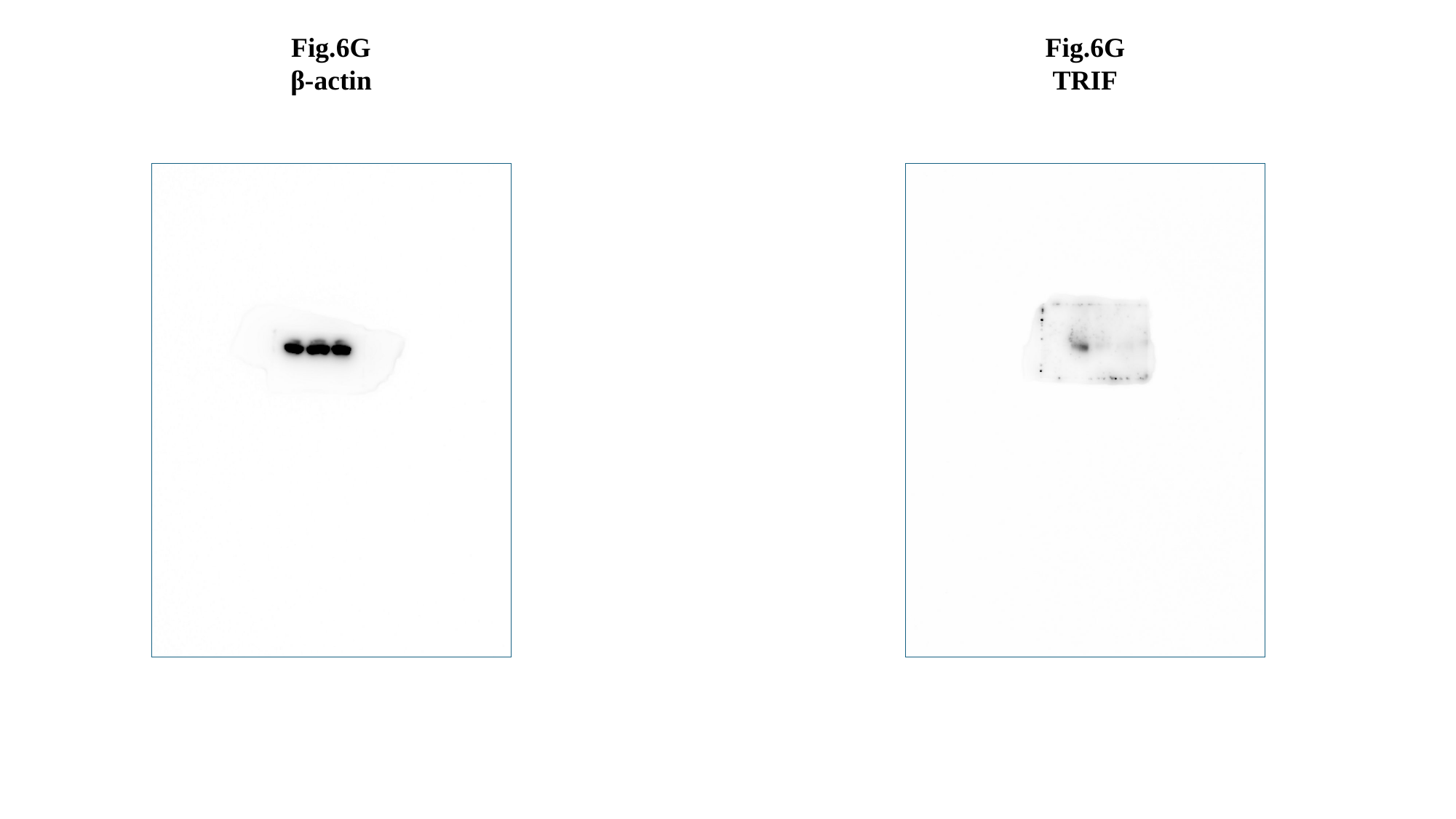

Fig.6G
β-actin
Fig.6G
TRIF

## Slide 25
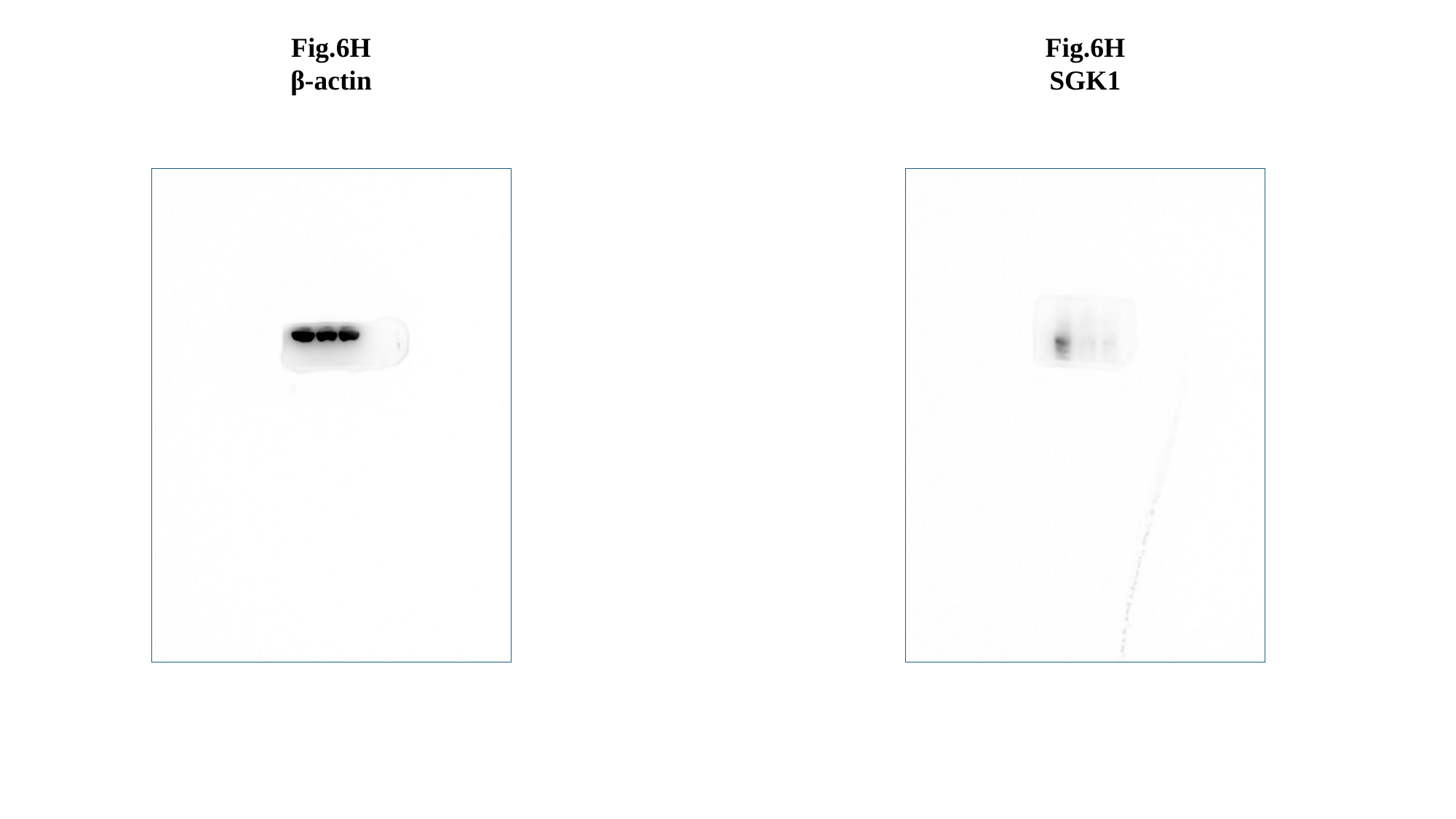

Fig.6H
β-actin
Fig.6H
SGK1
